# Supplementary material for: Multidimensional evaluation of the early emergence of executive function and development in Bangladeshi children using nutritional and psychosocial intervention: A randomized controlled trial protocol
Source: PLoS One. 2024 Mar 15;19(3):e0296529. doi: 10.1371/journal.pone.0296529 (PMC10942035; doi:10.1371/journal.pone.0296529)
Supplement: S1 File — (PDF) [file pone.0296529.s002.pdf]

# Information Sheet for mother with her 1-year-old child

| Protocol No. PR-21084 | Version No. 3 .00 | Date: '12th June'2022 |
|---|---|---|

**Protocol Title: Multidimensional evaluation of the early emergence of executive function and emotional regulation in young children in Bangladesh using nutritional and psychosocial intervention: A Pilot study**

**Investigator's name: Dr. Rashidul Haque**

**Organization:** International Centre for Diarrhoeal Disease Research, Bangladesh (icddr,b)

**Purpose of the research:** To determine the effect of nutritional intervention for improvement of cognition and emotional regulation among the children in Bangladesh where malnutrition and social adversities are common

**Background** (brief introduction of the issue and the need for/ importance of the research)
We are conducting a study to understand the problem of malnutrition and poor cognitive outcomes in children of Bangladesh.Malnutrition affects around 47 million children under 5 years of age in low- and middle-income countries annually and among then more than 20 million death occurs, others suffer long term cognitive and behavioural impairment. Malnutrition causing significant number of deaths of under 5 years child globally. It is a large problem in Bangladesh where 40% of under-fives have moderately acute malnutrition. Malnutrition is further worsened by poor diet. Inadequate feeding practices leading to deficiencies in vitamin and minerals. Studies show that, during infancy and early childhood, nutrition is essential as these are the crucial period for the formation of the brain, building the foundation for the development of cognitive, neurological and socio-emotional skills throughout childhood and adulthood.

**Why invited to participate in the study?**
Researchers from icddr,b, the University of Auckland, New Zealand, Boston Children Hospital, USA and Tropical Medicine Research Institute, Jamaica are jointly conducting this research study in your community to understand the effect of nutritional intervention on the cognition of moderate acute malnourished children . Total 210 children and 210 mother will be enrolled from this community. We invite you to help us in our efforts through your child's participation in this study, because you have 1 year±1m(11m-13m) of old child and you live in this community where malnutrition and social adversities are common. Research ethics committees at icddr,b and IRBs of University of Auckland, New Zealand, Boston Children Hospital, USA and Tropical Medicine Research Institute, Jamaica have approved this research study.

**Methods and procedures [What is expected from the participants of the research study?]**
You and your child will be requested to participate in the study. If you agree to participate; you are agreeing to provide information about you and your child's medical and personal information as well as, to allow the collection of a stool, blood sample, buccal scrab and body measurement such as height, weight from your child and blood, stool, buccal swab and body measurement such as height, weight from you as per protocol. Your child is also invited to participate Executive function, Emotional regulation, fNIRS, BAYLEY 4, LENA, PCI, Behavioural assessment questionnaires and EEG. In addition, study will provide nutritional supplementation based on randomization scheme, your child will receive RUSF followed by SQLNS or E- RUTF followed by E- SQLNS and Psychosocial stimulation to your child if your child is enrolled with WLZ/WHZ <-2 and ≥-3 z-score, and/or MUAC <12.5 and ≥11.5 cm having moderate acute malnutrition. After obtaining the signed consent from you we will start the study activities. However, no nutritional supplementation or psychosocial stimulation will be given to your child if your child is well nourished i.e WLZ/WHZ score >-1.

We will follow up your child up to 3yrs of age and perform the above-mentioned tests according to protocol.

**Screening and enrolment:**

Screening and enrolment will occur at the home / clinic by our trained study team. The team will review the eligibility criteria to confirm your child is eligible. If your child is eligible then team will explain the study to you and request to participate. If you sign this consent form for you and for your child then both of you will be enrolled in the study and we will collect details such as birth date, sex, information about your family (occupation, income, education, family size, length of time of breast feeding etc). We will also collect medical information, if it is needed. This enrolment procedure will take approximately 1 hour. Your child will be randomly assigned any of nutritional intervention if your child is enrolled with WLZ/WHZ <-2 and ≥-3 z-score, and/or MUAC <12.5 and ≥11.5 cm having moderate acute malnutrition. Randomization and interventions are not applicable who are enrolled with WLZ/WHZ score > -1 SD

We will provide you the contact information and location of local clinic to visit our clinic and our staff will start the surveillance for intervention.

**Procedures and sample collection :**

- Stool collection for all child: At enrolment at 1year±1m(11m-13m), , and then 2years±2m(22m-26m) and 3years±2m(34m-38m) of age (+/-7 days window)
- Additional stool collection only for MAM child: At the time of weight for length/height returns to normal (>-1SD) or at the end of 3 months of E-RUTF/RUSF intervention (+/-7 days window)
- Blood (2-3 ml) collection: At enrolment at 1year±1m(11m-13m), , and then 2years±2m(22m-26m) and 3years±2m(34m-38m), of age (+/-7 days window)
- Additional blood collection only for MAM child: At the time of weight for length/height returns to normal (>-1SD) or at the end of 3 months of E-RUTF/RUSF intervention (+/-7 days window)
- Anthropometry (height/length, weight, MUAC )):

  For MAM child: At enrolment 1year±1m(11m-13m),, then weekly until weight for height returns to normal (>-1SD) and then every 3 months interval for entire duration of the study (+/-7 days window).

  For well-nourished child: at enrolment 1year±1m(11m-13m),, then monthly for 3 months, then every 3 months interval for entire duration of the study (+/- 7 days window).

  Head circumference for all children will be measured at enrolment, at 2years±2m(22m-26m) and 3years±2m(34m-38m) of age
- Buccal Scrab: At the time of enrolment 1year±1m(11m-13m),,, 2years±2m(22m-26m) and 3years±2m(34m-38m), of age (+7 days window)
- Neuro development assessment (EF, ER, fNIRS, EEG, BAYLEY 4,,LENA, PCI, Behavioural assessment questionnaires): At enrollment 1year±1m(11m-13m),, then 2years±2m(22m-26m) and 3years±2m(34m-38m) of age (2 months window)

**Nutritional intervention:**

After randomization of 1:1 who are enrolled with WHZ <-2 and ≥-3 z-score, and/or MUAC <12.5 and ≥11.5 cm having MAM: One group of 70 children will receive locally produced Ready to use supplementary food (RUSF, 50 g/packet contains 204 kcal energy): Two packets of RUSF daily till the child's weight for length/height returns to normal (>-1SD) or for maximum 3 months and then be provided with 20 g daily SQLNS (contain 118 kcal energy) till end of the study.

And 70 children of other group will receive Enhanced Ready to use therapeutic feeds (E-RUTF), 50-100 kcal/kg/d daily until the child's weight for length/height returns to normal (>-1SD) or for maximum 3 months and then to be given 26g/day E-SQLNS (contain 130 k cal energy) till the end of the study.

**Psychosocial Stimulation**:

During the clinic and home visits health-workers will show the mothers how to play with home-made toys and books and interact with their children in a way to promote their development. Toys will be given to the child to play and learn until next visits, when a new set of developmentally appropriate toys will be provided. All activities will be conducted in a playful manner and not as a work-oriented activity. Bi weekly our staffs will be visit to household for this intervention.

**Behavioural Measures (Executive Functions/Emotional regulation):**

Executive functions are the processes involved in conscious control of thought and action including inhibitory control, planning and cognitive flexibility. Emotional Regulation is an integral part of self-regulation, which is a complex concept that measure cognitive flexibility, working memory and inhibition control skills of executive function.

This study involves two 2-3-hour sessions at the icddr,b Mirpur clinic for Neuro developmental assessment (EF, ER, fNIRS, EEG, BAYLEY 4, LENA, PCI, Behavioural assessment questionnaire) . The sessions will be scheduled on separate days within two weeks of each other at a time that is convenient for you and your child. Most of tasks of executive function and emotional regulation are direct interactions between the experimenter and the child, which will be recorded in video camera for video-scoring. These activities will be conducted in a playful manner and not as a work-oriented activity at 1year±1m(11m-13m),at 2 years ±2m(22m-26m and 3 years±2m (34m-38m time points .

Bayley-4

Bayley Scales of Infant and Toddler Development is an extensive formal developmental assessment tool for diagnosing developmental delays in early childhood. BAYLEY 4 assess development in children of 1-42 months old in 5 domains: cognition, motor, language, socio-emotional, and adaptive behavior. Baley-4 will be administered to all children. at 1year±1m(11m-13m), at 2 years±2m (22m-26m and 3 years ±2m (34m-38m) time points.

LENA:

Language Environment Analysis (LENA) LENA recorder and software measure the frequency of vocalization/verbalizations and conversational turns in children to see the influence of language input at different crucial points in early dev

elopment. . Recording data at multiple time points also gives us a measure of the consistency of the language environment. . at 1year±1m(11m-13m) ,at 2 years±2m (22m-26m and 3 years ±2m (34m-38m) time points .

**Parent-Child Interaction**

A 10-15-minute interaction between parent and child will be collected at 1year±1m(11m-13m), at 2 years±2m (22m-26m and 3 years ±2m (34m-38m) time points.

You (parents) will be asked to engage your child in play or conversations in the presence and absence of toys and books. The child will also play with the toys/books while the parent completes short surveys or watches a short video. This session will be recorded through video recording.

**NIRS Procedure:**

During one of sessions, we will record your child's brain activity using functional near-infrared spectroscopy (fNIRS). fNIRS measures and records the changes in the levels of oxygen in the blood by shining a near-infrared light into your child's head. We will be able to identify which areas of your child's brain are actively responding to the changes in images shown on the computer monitor.

For recording these changes, we use a computer which is attached to sensors and probes. Prior to placing the probes, we will measure your child's head with a measuring tape for proper fitting of the fNIRS sensors. These sensors rest on a headband, which is then placed around your child's head and adjusted for a tight fit.

While your child is wearing the headband, we will your child to watch videos of Resting state, Habituation & Novelty Detection and working memory tasks aim to assess child's Executive Function. There will also be sounds playing during some of the images/videos.

Finally, we will ask many different questions about your child's development (motor skills, speech, vision, etc.) in order to test for developmental disabilities. We will also ask you some questions from some questionnaires for behavioral assessment of your child and you which will take approximately 1.30 hours .We will also ask you some questions regarding impact of COVID 19 All information provided will be confidential, and if anything is concerning, further testing will be conducted, and referrals for care will be made free of charge. All of these procedures are completely safe and will not hurt your child. You will be with your child at all times.

**EEG Procedure:**

During the other visit to the clinic to perform EEG, we will record your child's brain activity using a small cap that is made of stretchy material. Each cap has many sponges on it and inside each sponge is a small recording sensor. We soak the caps in a warm salt water solution so the sponges get soft before we put the cap on the child's head. In this task, also before starting the session we need to measure the head circumference by measuring tape to get the appropriate cap size. As your brain is working, it is constantly giving off small electrical signals, which travel out to the scalp where we can pick them up with the special sensors. We will show a series of faces and patterns and record your child's brain activity during these tasks. Then, we will do the eye tracking measures. Your child will sit on your lap and watch a video of bright looming circles while we set up the eye-tracking equipment. The eye tracker is made up of a special computer monitor that has a set of infrared cameras built into the edges of the screen. Once calibrated, these cameras will follow eye movements and tell us exactly where on the screen your child is looking as he/she is watching the pictures. At your first visit we will have your child look at videos auditory, baseline, working memory, disengagement, flanker and VEP tasks. All these tasks will be used to assess Executive Function of a child by measuring emotional control, recognition, working memory, inhibitory control, attention, anticipation and cortical processing of vision. while we record your child's brain activity. Next, we will record your child's eye movements in response to a series of pictures on a computer screen. In addition, a digital video will be recorded to help the experimenter know when to present pictures to your child and aid data analysis. Your child's name will not be associated with the video recording and the file will be accessible only to the investigators of this study.

**Home visit**: For 1st one month Our study staff will visit your home daily to collect the data of complementary food consumption, review empty packets of complementary food and also collect the history of febrile illness, vomiting and diarrhoea. Then they will visit your home twice weekly and do the aforementioned procedures. They will also refer the child to the field clinic for attending clinic visit as well as primary care support by the study physician

**None of neuro developmental tasks are invasive or harmful**

**Procedures and sample collection for you (mother):**

- Stool collection: 10 gm once within 2 weeks of enrolment
- Blood collection: 5 ml once within 2 weeks of enrolment
- Buccal swab sample: Once within 2 weeks of enrolment
- Anthropometry (height and weight): Once within 2 weeks of enrolment

**Risk and benefits**

What are the risks from participating in the study?

Sometimes things happen to the research participants in research studies that may hurt them or make them feel bad. These are called risks. The risks of participating in this study include risks caused by intervention, neuro cognitive assessments and sample collection.

Intervention: There was no major adverse effects reported in earlier study, however we will collect all event related to the intervention.

Blood collection: Mild pain, discomfort, bleeding or bruising, or get an infection (which is extremely rare) where the needle or lancet is inserted. To minimize these risks, only trained, experienced staff will draw blood, and disposable materials will be used. if an infection results from a blood draw, we will provide any necessary treatment at no cost to you

Stool collection: We do not anticipate any risks from collecting stool.

Buccal swab: We do not anticipate any risks from collecting buccal swab

Neuro cognitive assessment: There are no major risks involved with those assessments. The number of tests during each day may cause some tiredness, but you and your children are allowed to take as many breaks as you need. All tests and procedures remain outside the child's body, pain free, and contain no bad effects.

If anything from the tests seems unusual or concerning (like abnormal seizure activity on EEG), child will be referred to the appropriate specialists in Bangladesh, and you will be contacted with information on what to do next. The study will cover the cost of transportation to the specialist, assistance with the consultation and referral process, but we will not cover the cost of medical care beyond primary medical care.

**Benefit**

You and Your child will receive free, high-quality primary care, and referrals for any illness through our study for the duration of participation. Additionally, there may be some benefit from taking study food.

Children who will fail to reach desired anthropometric levels even after completing the nutrition intervention package for 3 months, will be referred to nutritional rehabilitation-based facility for further evaluation and management to exclude any secondary cause of malnutrition specially Tuberculosis. But the child will remain in the study and we will continue to follow up to closely

**Principle of compensation**

There is no direct compensation for participation in this study, but in the case of any concerning information discovered, referrals to professionals will be provided free of charge. In addition, transportation to the clinic and food at the clinic will be provided.

**Privacy, anonymity and confidentiality**

We will keep all information collected from you and your child confidential and locked in a secure place under the responsibility of the study investigators. Data will be saved in secure servers at icddr, b, Boston Children's Hospital and .  Biological samples will be stored securely without identifying information at icddr, b. Neuro imaging testing will be done in private rooms, and subjects will be coded by a de-identified number. In addition, staff and researchers have completed the Course in The Protection of Human Research Subjects.

In general, anyone who is involved in this research, including those funding and regulating the study, may see the data, including information about you and your child, photos and videos.  For example, the following people might see information about you and your child:

a) Research staff at icddr,b
b) Research staff at Boston Children's Hospital
c) Research staff at Auckland University
d) Research staff at Tropical Medicine Research Institute, Jamaica

Your/your child's name and identity will not be disclosed in the process of analyzing, presenting or publishing the results of these procedures.

If you sign this form, you have given us permission to release information to authorized researchers and the safety committees, icddr,b Ethical Review Committee, regulatory authorities (both in Bangladesh and the United States), the study sponsor , Synopse ( a research data sharing and collaboration platform) and designees, and other research organizations. There is no expiration date to this permission. If you decide to withdraw your permission and end this agreement, please contact Dr. Rashidul Haque at the address/number listed below.  He or his staff will help you document in writing your decision to withdraw this permission.  Please note that any study information already obtained will continue to be used.

Your participation in this research is voluntary.  However, you will not be able to participate in this study if you do not sign this form.

**Future use of samples:**

At the end of the study, all of the specimens will be stored at icddr,b for 5 years . If you agree, we will store these samples to be used in the future for other research purposes. If such research is conducted by us or by our collaborators, appropriate approvals from respective authorities will be secured at that time.  If samples be used in the future, your and your child's privacy and anonymity will be maintained.

If you consent to having your and your child's samples saved to be used for future research, but change your mind later, you may contact us and the samples will be destroyed. If not, we will store your child's specimens for 5 years.  Of note, samples may be sent to collaborators outside of Bangladesh for specialized testing.

**Future use of information**

Information about you and your child may be shared with regulatory authorities including but not limited to the Ethical Review Committee (ERC) at the icddr,b, the Internal Review Board (IRB) at University of Auckland, Boston children hospital, Tropical Medicine Research Institute, Jamaica and the study sponsor and designees.  Investigators may choose to share information and data with other researchers at their discretion for the purpose of future research. Our study data will be shared with collaborators BCH is the data core, and we will be sending BCH our research data, including identifiers, which they will host in BCH REDCap database. An agreement with the Wellcome Leap foundation to receive funding is that our data, based on the data collected from all administered measures from individual participants in our study, will be shared with collaborators in the Leap consortium and will be available to Leap via the sponsor required Synapse (Maintained by Sage Bionetworks; synapse.org; WIRB#20112068). The data at BCH would be stored for at least 7 years after, but possibly even longer if needed. This is a sponsor-required data workflow. BCH will aid in processing and analysis of the data received from us.

**Right not to participate and withdraw**

Participation in this study is voluntary and you can choose to not participate, or withdraw at any point during the study without any penalty or loss of care.

# <u>Consent Sheet for mother with her 1-year-old child</u>

**SID:**

| **Protocol No. PR-21084** | **Version No. 3.00** | **Date: 12th June'2022** |
|---|---|---|

**Protocol Title: Multidimensional evaluation of the early emergence of executive function and emotional regulation in young children in Bangladesh using nutritional and psychosocial intervention: A Pilot study**

**Investigator's name: Dr. Rashidul Haque**

**Organization: International Centre for Diarrhoeal Disease Research, Bangladesh (icddr,b)**

If you agree to our proposal for enrolling you and your child in our study, please put √ mark on appropriate box(es) of the following and finally sign / left thumb print on the specified place for you:

| **Points** | **Status** |
|---|---|
| I have read out / study staff has read out the all information from this participants information sheet Version . 3 0, Dated 12th June'2022 about the study, have had the opportunity to ask questions, discuss the study, and received satisfactory answers | Yes ☐ No ☐ |
| I understood that I am free to leave the study without giving any reason | Yes ☐ No ☐ |
| I understood that the information that I gave will be confidential | Yes ☐ No ☐ |
| I agree to allow the study team to collect information from me and my child at every scheduled follow up visit and home visit | Yes ☐ No ☐ |
| I understood that the Information from this research study will be retained by icddr,b , Boston Children Hospital, USA, Auckland University, New Zealand and Tropical Medicine Research Institute, Jamaica, Synopse ( a research data sharing and collaboration platform) and in the future may be included in a de-identified public use database. De-identified means that I and my child will not be individually identified by name or other personal identifiers in the database. My full name or any address details will not be included. Information released will not identify me or my child's participation in this research study.<br>I am giving permission for those individuals to have access to my records. | Yes ☐ No ☐ |
| I agree to to allow the study team to collect all anthropometric data ( Height/length, weight, MUAC, Head Circumference) of my child | Yes ☐ No ☐ |
| I agree to the collection of blood (2-3 ml), buccal swab, stool from my child | Yes ☐ No ☐ |
| I agree to the collection of anthropometry (height, weight) ,blood (5 ml), buccal swab and stool sample from me | Yes ☐ No ☐ |
| I agree that anonymised blood, buccal swab and stool (those collected from me and my child) samples can be sent overseas for analysis | Yes ☐ No ☐ |
| I agree to feed nutritional intervention and phycological stimulation to my child (If my child is malnourished) | Yes ☐ No ☐ |
| I agree to perform neurocognitive tests (Executive Function, Emotional Regulation, fNIRS, EEG) to my child | Yes ☐ No ☐ |

| | |
|---|---|
| I agree to perform LENA recording, Bayley-4 assessment , Parent child interaction recording and questionnaires for my child developmental assessment | Yes ☐ No ☐ |
| I agree to storage and future use of me and my child's data and samples by ethically approved studies | Yes ☐ No ☐ |
| I agree to being contacted in the future for studies related to this study and I agree for future use of photos of my child for study purpose | Yes ☐ No ☐ |
| I understand that relevant sections of me and my child's medical notes and data collected during the study may be looked at by individuals from the sponsor and by regulatory authorities, where it is relevant to my taking part in this research. I give my permission for those individuals to have access to my records. | Yes ☐ No ☐ |
| I agree to participate in to this study | Yes ☐ No ☐ |

______________________________ ______________

Signature or left thumb impression of participant Date (dd/mmm/yyyy)

______________________________ ______________

Signature or left thumb impression of Parent/ Guardian/ Attendant Date (dd/mmm/yyyy)

______________________________ ______________

Signature of the witness Date ( dd/mmm/yyyy)

______________________________ ______________

Signature of the PI or his/her representative Date (dd/mmm/yyyy)

**Communication:**
If you have any question, you can ask me right now or at any time later to the below mentioned personnel:

| Purpose of contact | Name and address | Address for communication |
|---|---|---|
| For any question related to the study, or any problem | Dr. Masud Alam | Address: House 28, Avenue 1, Kalsi Road, Mirpur-12, Dhaka-1216, Mobile No. 01711570550 (to be open 7/24 hours) |
| | Name of PI: Dr. Rashidul Haque. | Address: Parasitology Laboratory, IDD, icddr,b , Mohakhali, Dhaka-1212 , Mobile: 01713093859 (9:00 am to 5:00 pm) |
| To know the rights or benefits or to log any complain or dissatisfaction | M A Salam Khan (IRB Coordinator) | IRB Secretariat, Research Administration, icddr,b, Mohakhali, Dhaka-1212 Phone: (+88-02) 9827084 or Mobile: 01711428989 |

Thank you for your cooperation.
A Copy of singed consent will be given to you.

## মা ও তার ১ বছর বয়সী শিশুর জন্য গবেষণা সম্পর্কিত তথ্য

| Protocol No. PR-21084 | Version No. 3.00 | Date: 12th June'2022 |
|---|---|---|

**Protocol Title: Multidimensional evaluation of the early emergence of executive function and emotional regulation in young children in Bangladesh using nutritional and psychosocial intervention: A Pilot study**

**Investigator's name: Dr. Rashidul Haque**

**Organization: International Centre for Diarrhoeal Disease Research, Bangladesh (icddr,b)**

### গবেষণার উদ্দেশ্য

এই গবেষণার উদ্দেশ্য হলো বাংলাদেশের শিশুদের বুদ্ধির বিকাশ এবং আবেগীয় নিয়ন্ত্রণ উন্নয়নের জন্য পুষ্টি খাওয়ানোর প্রভাব নির্ধারণ করা যেখানে অপুষ্টি এবং সামাজিক প্রতিকুলতা বিদ্যমান।

### ভুমিকা (সমস্যার সংক্ষিপ্ত ভুমিকা এবং এই গবেষণার গুরুত্ব / প্রয়োজনীয়তা)

নিম্ন ও মধ্যআয়ের দেশগুলোর মধ্যে পাঁচ বৎসরের নীচে শিশুদের মধ্যে সাড়ে চার কোটিরও বেশী শিশু প্রতিবছর অপুষ্টির শিকার হয়। এদের মধ্যে ২ কোটি শিশু মারা যায় এবং অন্যরা দীর্ঘ মেয়াদী বুদ্ধির বিকাশগত সমস্যায় ভুগে। সারা বিশ্বে অপুষ্টির কারনে ৫ বৎসরের নীচে শিশুদের মধ্যে উল্লেখযোগ্য সংখ্যক শিশু মারা যায়। এই অপুষ্টি বাংলাদেশের একটি বড় সমস্যা যেখানে পাঁচ বৎসরের নীচের ৪০% শিশু মাঝারি অপুষ্টিতে ভোগে। এই অপুষ্টি আরও খারাপ হয় যখন খাবারে ঘাটতি হয়। স্বল্প খাদ্য গ্রহনের অভ্যাসের কারনে শরীরে ভিটামিন ও খনিজ লবনের ঘাটতি দেয়া হয়। গবেষণায় দেখা গেছে যে, শৈশবকাল ও শৈশবকালীন সময়ে পুষ্টি গ্রহন অপরিহার্য কারন মস্তিষ্ক গঠনের জন্য ইহা গুরুত্বপূর্ণ সময়কাল। বুদ্ধির বিকাশ, শৈশব ও কৈশোরকালে স্নায়বিক এবং সামাজিক-মানসিক দক্ষতার ভিত্তি তৈরি করার জন্য পুষ্টি অপরিহার্য।

### কেন গবেষণায় অংশগ্রহণের আমন্ত্রন জানানো হচ্ছে?

আইসিডিডিআরবি, অকল্যান্ড ইউনিভার্সিটি নিউজিল্যান্ড, বোস্টন চিলড্রেনস হসপিটাল আমেরিকা এবং ট্রপিকাল মেডিসিন রিসার্চ ইন্সটিটিউট, জ্যামাইকার গবেষকবৃন্দ আপনাদের এলাকায় মাঝারি অপুষ্টির শিশুদের বুদ্ধির বিকাশের উপর পুষ্টি খাওয়ানোর প্রভাব দেখার জন্য যৌথভাবে এই গবেষণাটি পরিচালনা করছেন। আপনাদের এলাকার সর্বমোট ২১০ জন শিশু এবং ২১০ মায়েদের এই গবেষণায় অন্তর্ভুক্ত করা হবে। আপনার শিশুকে এই গবেষণায় অংশগ্রহণের জন্য আমরা আপনাকে আমন্ত্রন জানাচ্ছি কারন আপনি ১ বছর বয়সী ±১মাস (১১ মাস-১৩ মাস) শিশুর অভিভাবক এবং এই এলাকায় বসবাস করছেন যেখানে অপুষ্টি এবং বিভিন্ন সামাজিক প্রতিকুলতা বিদ্যমান। আইসিডিডিআরবি র গবেষণা নীতি কমিটি এবং ইউনিভার্সিটি অফ অকল্যান্ড, নিউজিল্যান্ড র আইআরবি, বোস্টন চিলড্রেনস হসপিটাল আমেরিকা এবং ট্রপিকাল মেডিসিন রিসার্চ ইন্সটিটিউট, জ্যামাইকা এই গবেষণাটির অনুমোদন দিয়েছেন।

### পদ্ধতি ও প্রক্রিয়া (গবেষণায় অংশগ্রহনকারীদের কাছ থেকে কি প্রত্যাশা?)

আপনি এবং আপনার শিশুকে এই গবেষণায় অংশগ্রহনের জন্য অনুরোধ জানাচ্ছি। আপনি যদি অংশগ্রহনে রাজি থাকেন,তাহলে,আপনি এবং আপনার শিশুর স্বাস্থ্য এবং ব্যক্তিগত তথ্য দিতেও সম্মত আছেন এবং পাশাপাশি আপনার এবং আপনার শিশুর মল, রক্তের নমুনা, মুখগহবর মিউকাসের নমুনা এবং শারীরিক পরিমাপ যেমন- উচ্চতা, ওজন সংগ্রহের অনুমতি দিচ্ছেন। আপনার শিশুকে আরও আমন্ত্রন জানানো হবে *(Executive Function)* বুদ্ধি পরীক্ষা, আবেগীয় নিয়ন্ত্রন,

(NIRS এবং EEG- পরীক্ষা তে অংশগ্রহন করার জন্য। এছাড়াও, এই গবেষণায় দৈবচয়ন প্রক্রিয়ার উপর ভিত্তি করে আপনার শিশু যদি ওজন/উচ্চতায়(WHZ <-2 and ≥-3 z-score, and/or MUAC <12.5 and ≥11.5 cm) মাঝারিঅপুষ্টি নিয়ে এই গবেষণায় অন্তর্ভুক্ত হয়, তাহলে তাকে পুষ্টির পরিপূরক (RUSF followed by SQLNS or E- RUTF followed by E- SQLNS ) ২টির যে কোন একটি এবং মনো-সামাজিক উদ্দীপনা দেয়া হবে। আপনার নিকট থেকে সাক্ষরিত সম্মতিপত্র পাওয়ার পর আমরা গবেষণা কার্যক্রম শুরু করবো।

যাই হোক, আপনার শিশু যদি অপুষ্ট না হয়, যেমন- ওজন/উচ্চতা স্বাভাবিক মাত্রায় (WHZ Score >-1 SD) হয়, তবে আপনার শিশুকে কোনপ্রকার পুষ্টির পরিপূরক বা মনো-সামাজিক উদ্দীপনা দেয়া হবে না।

আপনার শিশুকে ৩ বছর বয়স পর্যন্ত ফলোআপ করা হবে এবংউপরের উল্লিখিত পরিক্ষাগুলি করা হবে।

**বাছাইকরন এবং অন্তর্ভুক্তিকরনঃ**

আমাদের প্রশিক্ষনপ্রাপ্ত আমাদের কর্মী দ্বারা বাসায়/ক্লিনিকে এই বাছাইকরন এবং অন্তর্ভুক্তিকরন করা হবে। আপনার শিশু অন্তর্ভুক্তিকরনের যোগ্য কি না নিশ্চিত করতে দলটি অন্তর্ভুক্তিকরনের যোগ্যতা পর্যালোচনা করবে। আপনার শিশু যদি যোগ্য হয় তবে দলটি আপনাকে গবেষণা সম্পর্কে ব্যাখ্যা করবে এবং এই গবেষণায় অংশগ্রহণ করার জন্য অনুরোধ করবে। যদি আপনি নিজের এবং শিশুর জন্য সম্মতিপত্রে সাক্ষর করে থাকেন, তাহলে আপনারা উভয়ে এই গবেষণায় অন্তর্ভুক্ত হবেন এবং আমরা কিছু তথ্য বিশদভাবে সংগ্রহ করবো, যেমন- জন্মতারিখ, লিঙ্গ, আপনার পারিবারিক তথ্য (পেশা, আয়, শিক্ষা, পরিবারের আকার, শিশুকে বুকের দুধ খাওয়ানোর সময়কাল, ইত্যাদি )। আমরা প্রয়োজনানুসারে স্বাস্থ্য তথ্যও সংগ্রহ করবো। এই অন্তর্ভুক্তিকরন সময়কাল আনুমানিক ১ ঘণ্টা হবে। আপনার শিশু যদি ওজন/ উচ্চতা (WHZ <-2 and ≥-3 z-score, and/or MUAC <12.5 and ≥11.5 cm) মাঝারি অপুষ্টি নিয়ে এই গবেষণায় অন্তর্ভুক্ত হয়, তাহলে আপনার শিশু দৈবচয়িতভাবে যে কোন একটি পুষ্টির পরিপূরকের জন্য নির্বাচিত হবে। দৈবচয়ন এবং পুষ্টি খাওয়ানো সেইসব শিশুর জন্য প্রযোজ্য নয় যারা ওজন/উচ্চতা স্বাভাবিক মাত্রায় (WHZScore >-1 SD) নিয়ে গবেষণায় অংশগ্রহন করেছে। আমাদের ক্লিনিকে আসার জন্য আমরা আপনাকে যোগাযোগ সংক্রান্ত তথ্য এবং স্থানীয় ক্লিনিকের ঠিকানা প্রদান করবো এবং আমাদের কর্মী পুষ্টির পরিপূরক সম্পর্কিত তথ্য সংগ্রহ শুরু করবে।

**শিশুদের নমুনা সংগ্রহ এবং প্রক্রিয়াঃ**

- মল সংগ্রহঃ অন্তর্ভুক্তির সময়, ওজন অনুযায়ী উচ্চতা স্বাভাবিক মাত্রায় ১ বছর ±১ মাস (১১মাস-১৩মাস) (>-1 SD) ফেরত আসার সময়, এবং ২ বছর± ২ মাস (২২ মাস -২৬ মাস)ও ৩ বছর±২ মাস (৩৪মাস-৩৮ মাস)মাস বয়সে (+/- ৭ দিন)।
- মাঝারি অপুস্টির শিশুদের অতিরিক্ত আরেকটি মলের নমুনা ওজন অনুযায়ী উচ্চতা স্বাভাবিক মাত্রায় (>-১ ঝড) ফেরত আসার সময় সংগ্রহ করবো অথবা ৩ মাসের সময় যখন পুস্টি পরিপূরক প্রদান করা শেষ হবে (+/- ৭ দিন) ।
- রক্ত (২-৩ মিঃলিঃ) সংগ্রহঃ অন্তর্ভুক্তির সময়, ওজন অনুযায়ী স্বাভাবিক ১ বছর ±১ মাস (১১মাস-১৩মাস) ( >-1 SD) ফেরত আসার সময়, এবং ২ বছর± ২ মাস (২২ মাস -২৬ মাস) ও ৩ বছর±২ মাস(৩৪মাস-৩৮ মাস) মাস বয়সে (+/- ৭ দিন)।
- মাঝারি অপুস্টির শিশুদের অতিরিক্ত আরেকটি রক্তর নমুনা ওজন অনুযায়ী উচ্চতা স্বাভাবিক মাত্রায় (>-1 SD) ফেরত আসার সময় সংগ্রহ করবো অথবা ৩ মাসের সময় যখন পুস্টি পরিপূরক প্রদান করা শেষ হবে (+/- ৭ দিন) ।

- শারীরিক পরিমাপ (উচ্চতা , ওজন ও উপর বাহুর মধ্যমার পরিধি):
  - অপুষ্ট শিশুর জন্যঃ অন্তর্ভুক্তির সময়, ১ বছর ±১ মাস (১১মাস- ১৩মাস)তারপর প্রতি সপ্তাহে একবার যতক্ষণ না ওজন অনুযায়ী উচ্চতা স্বাভাবিক মাত্রায় (>-1 SD) ফেরত না আসে এবং তারপর ৩ মাস অন্তর অন্তর পুরো গবেষণাকালের শেষ সময় পর্যন্ত ( +/- ৭ দিন )।
  - অপুষ্ট নয় শিশুর জন্যঃ অন্তর্ভুক্তির সময়, ১ বছর ±১ মাস (১১মাস- ১৩মাস)তারপর ৩ মাস পর্যন্ত প্রতি মাসে, তারপর ৩ মাস অন্তর অন্তর পুরো গবেষণাকালের শেষ সময় পর্যন্ত (+/- ৭ দিন)।

শিশুর মাথার পরিধির মাপ নেওয়া হবে: অন্তর্ভুক্তির সময়, ২ বছর ± ২ মাস **(**২২ মাস **-**২৬ মাস**)** ও ৩ বছর±২ মাস (৩৪মাস-৩৮ মাস) বয়সে **(+/-** ২ মাস **)**।

- মুখগহবর থেকে মিউকাসের নমুনা সংগ্রহঃ অন্তর্ভুক্তির সময়, ১ বছর ±১ মাস (১১মাস- ১৩মাস), ২ বছর± ± ২ মাস (২২ মাস -২৬ মাস) ও ৩ বছর±২ মাস (৩৪মাস-৩৮ মাস) বয়সে (+/- ৭ দিন)।
- বুদ্ধি বিকাশের পরীক্ষাঃ (*Executive Function*/বুদ্ধি পরীক্ষা, আবেগীয় নিয়ন্ত্রণ, *fNIRS* এবং *EEG* LENA রেকর্ডিং, বেইলী-৪ মূল্যায়ন, পিতামাতা ও সন্তানের পারস্পরিক মিথিস্ক্রিয়া রেকর্ডিং এবং আমার শিশুর বিকাশ্মুলক মূল্যায়নের জন্য প্রশ্নমালা): অন্তর্ভুক্তির সময় ১ বছর ±১ মাস (১১মাস- ১৩মাস), ২ বছর± ± ২ মাস (২২ মাস -২৬ মাস) ও ৩ বছর±২ মাস (৩৪মাস-৩৮ মাস) য়সে ( ২ মাস window )।

## পুষ্টির পরিপূরক প্রদানঃ

দৈবচয়ন ১:১ পরে, যে সব শিশু ওজন/ উচ্চতা (WHZ <-2 and ≥-3 z-score, and/or MUAC <12.5 and ≥11.5 cm ) মাঝারি অপুষ্টি নিয়ে এই গবেষণায় অন্তর্ভুক্ত হবেঃ ৭০ জন শিশুর একটি দল স্থানীয়ভাবে উৎপাদিত পরিপূরক খাবার গ্রহন করবে (জটঝঋ, ৫০ গ্রাম/প্যাকেট সম্বলিত ২০৪ কিঃ ক্যালোরী শক্তি): *RUSF* এর দুই প্যাকেট প্রতিদিন পাবে যতদিন উচ্চতা অনুযায়ী ওজন সাধারণ মাত্রায় ( >১- ঝউ) ফেরত না আসে অথবা সর্বোচ্চ ৩ মাস এবং তারপর প্রতিদিন ২০ গ্রাম *SQLNS* প্রদান করা হবে গবেষণার শেষদিন পর্যন্ত। আরেক দলের ৭০ জন শিশু বর্ধিতভাবে প্রস্তুতকৃত থেরাপিউটিক খাবার (*E-RUTF*),৫০-১০০ কিঃ ক্যালোরী/কেজি/ দিন হিসাবে পাবে যতদিন উচ্চতা অনুযায়ী ওজন সাধারণ মাত্রায় ( >১- ঝউ) ফেরত না আসে অথবা সর্বোচ্চ ৩ মাস এবং তারপর প্রতিদিন ২০ গ্রাম *E-SQLNS* প্রদান করা হবে গবেষণার শেষদিন পর্যন্ত।

## মনো-সামাজিক উদ্দীপনাঃ

ক্লিনিক এবং বাড়ি পরিদর্শনের সময় আমাদের স্বাস্থ্যকর্মীগন মায়েদের দেখিয়ে দিবেন যে তাদের শিশুদের সাথে বাড়িতে তৈরি খেলনা এবং বই দিয়ে কিভাবে খেলতে হয় এবং ভাবের আদান প্রদানের মাধ্যমে কিভাবে শিশুদের বিকাশ ভাল করা যায়। শিশুর বিকাশের সাথে সম্পর্কিত একটি খেলনার সেট দেয়া হবে, যাতে পরবর্তী পরিদর্শনের আগ পর্যন্ত শিশু তা দিয়ে খেলতে পারে আর শিখতে পারে ।এই সমস্তকিছুই খেলাধুলার মত করে করা হবে কোন কাজের মত নয়। এই ইন্টাভেনশন দেয়ার জন্য আমাদের একজন স্টাফ দুই সপ্তাহে একদিন বাড়ী ভিজিট করবেন।

আচরণগত পরিমাপ **(**এক্সিকিউটিভ ফাংশন**/** নির্বাহী কাজ**/** আবেগ নিয়ন্ত্রণ**):**

এক্সিকিউটিভ ফাংশন হচ্ছে এমন একটি প্রক্রিয়া যেখানে চিন্তা করা এবং কাজ করার মাঝে ব্যাক্তির সচেতন নিয়ন্ত্রণ থাকে, যার মধ্যে রয়েছে বাধানিষেধ নিয়ন্ত্রণ, পরিকল্পনা এবং জ্ঞানীয় নমনীয়তা। আবেগ নিয়ন্ত্রন হল নিজেকে নিয়ন্ত্রনের একটি অবিচ্ছেদ্য অংশ, যা আবেগ, প্রেরণা, মনোযোগ, সামাজিক মিথস্ক্রিয়া এবং শারীরিক আচরণ নিয়ন্ত্রণ করার একটি জটিল ধারণা। যেটা এক্সিকিউটিভ ফাংশনের বুদ্ধিগত

নমনীয়তা,কার্যকারী স্মৃতিএবং বাধা নিয়ন্ত্রণের দক্ষতা পরিমাপ করে।

আইসিডিডিআরবি মিরপুর ক্লিনিকে এই নিউরো কগনিটিভ মূল্যায়ন (Executive Function/বুদ্ধি পরীক্ষা, আবেগীয় নিয়ন্ত্রণ, fNIRS এবং EEG, LENA রেকর্ডিং, বেইলী-৪ মূল্যায়ন, পিতামাতা ও সন্তানের পারস্পরিক মিথিস্ক্রিয়া রেকর্ডিং এবং আমার শিশুর বিকাশ্মূলক মূল্যায়নের জন্য প্রশ্নমালা)-র কাজগুলো ২-৩ ঘণ্টায় সম্পন্ন করা হবে। সেশনগুলি আপনার এবং আপনার শিশুর জন্য সুবিধাজনক যে কোনও একটি সময়ে দুই সপ্তাহের মধ্যে পৃথক দুই দিনে করা হবে। এই এক্সিকিউটিভ ফাংশন এবং আবেগ নিয়ন্ত্রন কার্যকলাপের বেশিরভাগই পরীক্ষক ও আপনার শিশুর মধ্যে সরাসরি ভাবের আদান প্রদান হবে যা ভিডিও ক্যামেরায় রেকর্ড করে স্কোরিং করা হবে। এই সমস্তকিছু শুধুমাত্র খেলাধুলার মাধ্যমে পরিচালিত হবে কোন কাজের মত করে নয় ১ বছর ±১ মাস (১১মাস- ১৩মাস), ২ বছর± ± ২ মাস (২২ মাস -২৬ মাস)) ও ৩ বছর±২ মাস (৩৪মাস-৩৮ মাস) সময় নির্দেশক

বেইলী – ৪
বেইলী স্কেলস অফ ইনফ্যান্ট এন্ড টডলার ডেভেলপমেন্ট হল প্রাক শৈশবকালে বিকাশগত বিলম্ব নির্ণয়ের জন্য একটি ব্যাপক আনুষ্ঠানিক উন্নয়নমূলক মূল্যায়নের সরঞ্জাম। বেইলী – ৪ শিশুদের ১-৪২ মাসের মধ্যে তাদের বিকাশের ৫ টি দিক পরিমাপ করেঃ জ্ঞানীয়, পেশিসঞ্চালন,ভাষা,সামাজিক-আবেগীয় এবং অভিযোজিত আচরণ।বেইলী – ৪ সকল শিশুদের উপরে পরিচালনা করা হবে। অন্তর্ভুক্তির সময়ে ১ বছর ±১ মাস (১১মাস- ১৩মাস), ২ বছর± ± ২ মাস (২২ মাস -২৬ মাস)) ও ৩বছর বয়সে ±২ মাস(৩৪ মাস-৩৮ মাস)

LENA
ল্যাংগুয়েজ এনভায়রনমেন্ট এনালাইসিস (LENA) LENA রেকর্ডার এন্ড সফটওয়কারীশিশুদের প্রাথমিক বিকাশের বিভিন্ন গুরুত্বপূর্ণ সময়ে ভাষার ইনপুটের প্রভাব দেখতে শিশুদের কন্ঠসর/বাচ্যায়ন এবং কথোপকথনের কম্পাঙ্ক বা ফ্রিকোয়েন্সি পরিমাপ করে। একাধিক সময়ে তথ্য রেকর্ড করার ফলে তা আমাদের ভাষা পরিবেশের সামঞ্জস্যের একটি পরিমাপ দেয়। অন্তর্ভুক্তির সময়ে ১ বছর ±১ মাস (১১মাস- ১৩মাস), ২ বছর± ± ২ মাস (২২ মাস -২৬ মাস) ও ৩বছর বয়সে ±২ মাস(৩৪ মাস-৩৮ মাস)
পিতামাতা ও সন্তানের পারস্পরিক মিথস্ক্রিয়াঃ

পিতামাতা ও শিশুর মধ্যে পারস্পরিক মিথস্ক্রিয়ার ১০-১৫ মিনিটের একটি তথ্য সংগ্রহ করা হবে। অন্তর্ভুক্তির সময়ে ১ বছর ±১ মাস (১১মাস- ১৩মাস), ২ বছর± ± ২ মাস (২২ মাস -২৬ মাস)) ও ৩বছর বয়সে ±২ মাস(৩৪ মাস-৩৮ মাস)। খেলনা এবং বইয়ের উপস্থিতি বা অনুপস্থিতিতে আপনাকে (পিতামাতা) শিশুর সাথে খেলা অথবা কথাবার্তার মাধ্যমে তাকে ব্যস্ত রাখতে বলা হবে। পিতামাতা যখন কোন সংক্ষিপ্ত সার্ভে করবেন বা সংক্ষিপ্ত ভিডিও দেখতে থাকবেন তখন শিশুকে খেলনা/বই দিয়ে খেলতে দেয়া হবে। পুরো সেশনটি ভিডিও রেকর্ডারের মাধ্যমে রেকর্ড করে রাখা হবে।

***fNIRS পদ্ধতিঃ***
একটি সেশনে, fNIRS (ফাংশনাল নেয়ার ইনফ্রারেড স্পেক্ট্রস্কপি) প্রযুক্তি ব্যাবহার করে আপনার শিশুর মস্তিষ্কের সক্রিয়তা লিপিবদ্ধ করবো। ভঘওজঝ আপনার শিশুর মাথায় একটি উজ্জ্বল লেজার লাইট/ রশ্মি প্রবেশ করিয়ে তার রক্তে অক্সিজেনের পরিমানের পরিবর্তন পরিমাপ করে এবং লিপিবদ্ধ করে রাখে। এর ফলে আমরা চিহ্নিত করতে সমর্থ হব যে কম্পিউটারের মনিটরের ছবি পরিবর্তনের সাথে সাথে আপনার শিশুর মস্তিষ্কের কোন অংশটি সক্রিয়ভাবে প্রতিক্রিয়া করে। শিশুর মাথায় যে উজ্জ্বল আলো প্রবেশ করানো হবে এতে শিশুর কোন ক্ষতি হবে না এবং সে কোন ধরনের তাপ বা ব্যাথা অনুভব করবে না।

যেকোনো ধরনের পরিবর্তন লিপিবদ্ধ করে রাখার জন্য আমরা একটি কম্পিউটার ব্যাবহার করবো। কম্পিউটারের সাথে সেন্সরস বা সংবেদক এবং প্রবস বা শলাকা সংযুক্ত থাকবে। আপনার শিশুর মাথায় প্রবস/ শলাকা স্থাপন করার পূর্বে ,পরিমাপক ফিতা দিয়ে শিশুর মাথা আগে মেপে নেয়া হবে যাতে fNIRS সেন্সরস / সংবেদকগুলো সঠিক ভাবে লেগে থাকে। সেন্সর /সংবেদকগুলো একটি মাথার বন্ধনীর উপর লাগানো থাক, যা পরে শিশুর মাথার চারপাশে যথাযথ এবং শক্ত করে লাগিয়ে দেয়া হবে।

যখন শিশুর মাথায় বন্ধনী লাগানো থাকবে, তখন শিশুকে মনিটরে চলমান মহিলার ছবি দেখানো হবে বিশ্রামরত অবস্থা, অভ্যস্থতা, নূতনত্বের প্রতি প্রতিক্রিয়া এবং কার্যকরী স্মৃতির মাধ্যমে শিশুর নির্বাহী কার্যক্রম মূল্যায়ন করা হবে। , কিছু কিছু ছবি এবং ভিডিও দেখানোর সময় শব্দ হতে থাকবে।

সর্বোপরি, আমরা আপনার শিশুর বিকাশ সম্বন্ধে নানান ধরনের প্রশ্ন জিজ্ঞাসা করবো যেমন-বিভিন্ন দক্ষতা কোন কিছু স্পর্শ করা/ধরে রাখা /নাড়াচাড়া করানো,কথা বলা ,দেখা, ইত্যাদি) যাতে আমরা তার বিকাশমূলক অক্ষমতা/ ধীরতা এবং কর্মসম্পাদনমূলক দক্ষতা পরীক্ষা করে দেখতে পারি। আমরা আপনাকে শিশুর আচরণগত পরিমাপ সম্পর্কিত প্রশ্নমালা থেকে কিছু প্রশ্নও জিজ্ঞাসা করবো এবং যার জন্য আনুমানিক ১:৩০ ঘণ্টা সময় লাগবে। আমরা কোভিড-১৯ এর প্রভাব সম্পর্কেও আপনাকে কিছু প্রশ্ন জিজ্ঞাসা করবো।আমরা যেসব তথ্য সংগ্রহ করবো তা ব্যক্তিগত ও গোপনীয় থাকবে ,এবং পরীক্ষাগুলো থেকে যদি অস্বাভাবিক কিছু পাওয়া যায় তবে, আরও পরীক্ষা নিরীক্ষা করা হবে এবং তাকে যথাযথ বিশেষজ্ঞর কাছে পাঠানো হবে। সব পদ্ধতিসমূহ সম্পূর্ণ নিরাপদ এবং এতে আপনার শিশু কোন ব্যাথা পাবেনা। পুরোটা সময় আপনি আপনার শিশুর সাথেই থাকবেন।

**EEG পদ্ধতিঃ**

ক্লিনিকে অন্য আরেকটি ভিজিট বা সাক্ষাতের সময়, আমরা সম্প্রসারণশীল উপকরন দিয়ে তৈরি একটি ছোট টুপি দিয়ে আপনার শিশুর মস্তিষ্কের সক্রিয়তা লিপিবদ্ধ করবো। প্রতিটি টুপিতে অনেকগুলো স্পঞ্জ /ছিদ্র রয়েছে এবং প্রতিটি স্পঞ্জের ভিতরে একটি করে ছোট রেকর্ডিং স্পন্সর / সংবেদক রয়েছে। আপনার শিশুর মাথায় টুপিটি পরানোর পূর্বে উষ্ণ লবন পানির দ্রবনে ভিজিয়ে রাখব যাতে এর স্পঞ্জগুলো ভিজে নরম হয়ে থাকে। আমরা একটি পরিমাপক ফিতা দিয়ে আপনার শিশুর মাথার চারপাশের দূরত্ব (মাথার পরিধি) মাপব যাতে আপনার শিশুর মাথার মাপ অনুযায়ী সঠিক টুপি আমরা ব্যাবহার করতে পারি। যেহেতু আপনার শিশুর মস্তিষ্ক সক্রিয়, সেহেতু এটি ক্রমাগত ভাবে বৈদ্যুতিক সংকেত পাঠাতে থাকবে,মাথার খুলির ওপর যা ঘুরতে থাকা অবস্থায় টুপিতে লাগানো বিশেষ সেন্সর বা সংবেদক গুলো দিয়ে ধরে রাখবো। পর্দায় আমরা তাকে কিছু মুখাবয়ব এবং আকৃতির ছবি দেখাতে থাকবো এবং আপনার শিশুর প্রতিক্রিয়া লিপিবদ্ধ করবো। এরপরে চোখের নড়াচড়া পরিমাপ এর কাজ করবো। আই ট্র্যাকিং যন্ত্রপাতি স্থাপন করার সময়ে আপনার শিশু আপনার কোলে বসে থাকবে এবং উজ্জ্বল কিছু বৃত্তের একটি ভিডিও দেখতে থাকবে। আই ট্র্যাকারটি একটি বিশেষ কম্পিউটারের পর্দা দিয়ে বানানো যাতে পর্দার ধার ঘেঁষে অনেকগুলো লাল আলোর ইনফ্রারেড ক্যামেরা আছে। এই ক্যামেরাগুলো চোখের নাড়াচাড়া অনুসরন করবে এবং পর্দায় দেখানো ছবিগুলো দেখার সময় আপনার শিশু পর্দার ঠিক কোন জায়গাটি দেখছে তা বুঝতে সাহায্য করবে। আপনার প্রথম ভিজিটে আমরা আপনার শিশুকে ভিডিও দেখাবো, শব্দ শোনাবো, বেজলাইন, কার্যকরী স্মৃতি, আলাদা করতে পারা, ফ্ল্যাঙ্কার এবং ভিএপি কাজ দিব। যখন আমরা আপনার শিশুর মস্তিষ্কের কার্যকলাপ রেকর্ড করবো তখন এই সব কাজ আবেগ নিয়ন্ত্রন, কোন কিছু চিনতে পারা, কার্যকরী স্মুতি, বাঁধা নিয়ন্ত্রন,মনোযোগ, প্রত্যাশা/অপেক্ষা/পুর্বঅনুমান, কোন কিছুর দিকে তাকানো পরিমাপ করার মাধ্যমে নির্বাহী কার্যক্রম মূল্যায়নের জন্য ব্যবহার করা হবে।

তারপর,কম্পিউটার স্ক্রিনে দেখানো বিভিন্ন ছবির প্রতি আপনার শিশুর চোখের নড়াচড়া লিপিবদ্ধ করবো। একইসাথে, পুরো সেশনটিতে, কোন কিছুর দিকে শিশুর তাকানো এবং আচরন একটি ডিজিটাল ভিডিও দিয়ে রেকর্ড করে রাখা হবে। এটি গবেষকদের তথ্যসমুহ ভালভাবে বিশ্লেষণ করতে সাহায্য করবে। আপনার শিশুর গোপনীয়তা রক্ষার জন্য, তার নাম ভিডিও রেকর্ডিং এর সাথে দেয়া হবে না এবং এই ফাইল শুধুমাত্র গবেষণার প্রধান গবেষক দেখতে পারবেন।

**খানা থেকে তথ্য সংগ্রহ** :
আমাদের কর্মীরা ১ম এক মাস প্রতিদিন এবং তারপর সপ্তাহে ২ দিন আপনার বাসায় যেয়ে পুষ্টি পরিপুরক খাওয়ার তথ্য ও প্যাকেট যাচাই করবে। এর সাথে সাথে আপনার শিশুর জ্বর, বমি, ডাইরিয়াতে ভুগতেছে কিনা তার তথ্যও সংগ্রহ করবে গবেষণার শেষ পর্যন্ত। তারা আপনাকে শিশুর নির্ধারিত সাক্ষাতের কথা স্বরণ করিয়ে দিবে এবং দরকার হলে প্রাথমিক চিকিৎসার জন্য আমাদের ক্লিনিকে পাঠাবে।

বুদ্ধি পরীক্ষার মূল্যায়নের কোন কাজই ক্ষতিকারক নয়।

**আপনার (মায়ের) নমুনা সংগ্রহ এবং প্রক্রিয়াঃ**
শারীরিক পরিমাপ (উচ্চতা এবং ওজন): তালিকাভুক্তির ২ সপ্তাহের মধ্যে একবার।
রক্ত সংগ্রহঃ তালিকাভুক্তির ২ সপ্তাহের মধ্যে একবার ৫ মিলি।
মুখগহবর থেকে মিউকাসের নমুনাঃ তালিকাভুক্তির ২ সপ্তাহের মধ্যে একবার।
মল সংগ্রহঃ তালিকাভুক্তির ২ সপ্তাহের মধ্যে একবার ১০ গ্রাম।

**ঝুঁকি এবং সুবিধাদিঃ**
এই গবেষণায় অংশগ্রহণের ঝুঁকিসমূহ কি কি?
কখনো কখনো গবেষণায় অংশগ্রহণকারীদের সাথে এমন কিছু ঘটে যা তাদের কোন ক্ষতি করতে পারে বা তাদের খারাপ অনুভূতি হতে পারে। এইগুলো কে ঝুঁকি বলা হয়। এই গবেষণায় অংশগ্রহণের ঝুঁকি সমূহের মধ্যে রয়েছে পুষ্টির পরিপূরক প্রদান, বুদ্ধি পরীক্ষা এবং নমুনা সংগ্রহ করার সময় ।

**পুষ্টির পরিপূরকঃ** আগের কোন গবেষণায় কোন খারাপ প্রভাব হয়েছিলো বলে জানা নাই, তবে আমরা ইন্টারভেনশন সম্পর্কিত আরও তথ্য সংগ্রহ করবো।

**রক্ত সংগ্রহঃ** হাল্কা ব্যাথা, অস্বস্তি, রক্তপাত অথবা জখম, অথবা সুঁই বা লেন্সেট রয়েছে এমন কোন ইনজেকশন দেয়া (যা অত্যন্ত বিরল) । এই ঝুঁকি সমূহ কমানোর জন্য, শুধুমাত্র প্রশিক্ষণ প্রাপ্ত, অভিজ্ঞ কর্মী রক্ত সংগ্রহ করবে, এবং দ্বিতীয়বার ব্যাবহার অনুপযোগী উপকরণ ব্যাবহার করা হবে। যদি রক্ত সংগ্রহ করার সময় কোন সংক্রমণ হয়, প্রয়োজনীয় চিকিৎসা প্রদাণ করা হবে, যার জন্য আপনাকে কোন খরচ করতে হবে না।

**মল সংগ্রহঃ** মল সংগ্রহ করার সময় কোনরকম ঝুঁকি আছে বলে আমরা প্রত্যাশা করি না।

**মুখগহবর থেকে মিউকাসের নমুনাঃ** মুখগহবর থেকে মিউকাসের নিঃসৃত নমুনা সংগ্রহ করার সময় কোনরকম ঝুঁকি আছে বলে আমরা প্রত্যাশা করি না।

**বুদ্ধি পরীক্ষার মূল্যায়নঃ** এই মূল্যায়ন গুলোতে তেমন কোন বড় ঝুঁকি নেই। প্রতিদিনের করা পরীক্ষার সংখ্যা কিছুটা ক্লান্তির কারন হতে পারে, তবে আপনার এবং আপনার শিশুকে আপনাদের প্রয়োজনমত বিশ্রাম দেয়া হবে।সমস্ত পরীক্ষা এবং পদ্ধতি শিশুর শরীরের বাইরে করা হবে,যা ব্যাথামুক্ত এবং কোন খারাপ প্রভাব মুক্ত।

এই পরীক্ষাগুলো থেকে যদি এমন কিছু পাওয়া যায় যা অস্বাভাবিক বা উদ্বেগজনক (উউএ কার্যক্রমে অস্বাভাবিক খিঁচুনি), তবে শিশুকে এই বিষয়ে অভিজ্ঞ বাংলাদেশ এর কোন বিশেষজ্ঞের কাছে রেফার করে দেয়া হবে, এবং প্রয়োজনীয় তথ্যসহ পরবর্তীতে আপনাকে কি করতে হবে তা জানানো হবে। এই গবেষণায় আপনাকে বিশেষজ্ঞের কাছে যাবার জন্য গাড়ির ব্যবস্থা করে দেয়া হবে, পরামর্শ এবং রেফারেল প্রক্রিয়ায় সাহায্য করা হবে, তবে আমরা প্রাথমিক চিকিৎসার বাইরে অন্য কোন চিকিৎসার ব্যয়ভার বহন করবো না।

**সুবিধাঃ**

আপনি এবং আপনার শিশু বিনাখরচে ভালমানের প্রাথমিক চিকিৎসা পাবেন এবং গবেষণায় অংশগ্রহণকালীন সময়ে অন্যান্য অসুখে রেফার করে দেয়া হবে। আরও বলতে গেলে, গবেষণা থেকে প্রাপ্ত খাবার থেকেও কিছু সুবিধা পেতে পারেন। ৩ মাস পুষ্টির পরিপূরক খাওয়ানোর পর যাদের উচ্চতা অনুযায়ী ওজন স্বাভাবিক হবে না তাদেরকে বিশেষায়িত স্বাস্থ্যকেন্দ্রে পাঠানো হবে, কেন তারা স্বাভাবিক অবস্থায় আসছে না তার কারণ গুলো বের করার জন্য, বিশেষ ভাবে আমরা যক্ষ্মার জন্য পরীক্ষা করতে বলবো। গবেষণা চলাকালিন পুরো সময় শিশু গবেষণায় অন্তর্ভুক্ত থাকবে এবং আমরা তাকে নিবিড় পর্যবেক্ষণ করবো।

**আর্থিক সুবিধাঃ**

গবেষনায় অংশগ্রহণের জন্য সরাসরি কোন আর্থিক সুবিধা দেয়া হবে না, তবে যদি উদ্বেগজনক কোন তথ্য পাওয়া যায় তখন বিনা খরচে বিশেষজ্ঞের কাছে রেফার করে দেয়া হবে। এছাড়াও নির্ধারিত সাক্ষাতের জন্য যাতায়াত খরচ এবং ক্লিনিকে অবস্থানকালীন সময়ে কিছু খাবার দেয়া হবে।

**ব্যাক্তিগত, নামহীনতা এবং গোপনীয়তা**

আপনার এবং আপনার শিশুর কাছ থেকে সংগৃহীত সকল তথ্য গোপনীয় রাখা হবে এবং তা একটি সুরক্ষিত জায়গায় গবেষণার গবেষকবৃন্দের দায়িত্বে জমা রাখা হবে। তথ্যসমূহ আইসিডিডিআরবি এবং বোস্টন চিলড্রেন হসপিটালের সুরক্ষিত সার্ভারে জমা রাখা হবে। জৈবিক নমুনা কোনরকম চিহ্নিতকরণ তথ্য ছাড়া আইসিডিডিআরবি তে সুরক্ষিতভাবে রাখা হবে। নিউরো ইমেজিং পরীক্ষাগুলো পৃথক কক্ষে করানো হবে, এবং অংশগ্রহণকারীকে একটি পুনঃ চিহ্নিতকরণ নাম্বার দিয়ে কোড করা হবে। উপরন্তু , কর্মী এবং গবেষকবৃন্দের গবেষণায় অংশগ্রহণকারীদের স্বার্থ রক্ষা সম্পর্কিত কোর্স সম্পন্ন করা আছে।

সাধারনভাবে, যারা এই গবেষণার সাথে যুক্ত আছেন, এমনকি যারা আর্থিক সহায়তা দিয়েছেন এবং গবেষণার নীতিনির্ধারক তারাও তথ্যসমূহ দেখতে পাবেন, যার মধ্যে রয়েছে আপনার এবং আপনার শিশুর সম্পর্কিত তথ্য, স্থিরচিত্র এবং ভিডিও। উদাহরনস্বরূপ, নিম্নলিখিত ব্যাক্তিবর্গ আপনার এবং আপনার শিশুর সম্পর্কিত তথ্য জানতে পারে

- আইসিডিডিআর,বি র গবেষনাকর্মী
- বোস্টন চিলড্রেন হসপিটালের গবেষনাকর্মী
- অকল্যান্ড ইউনিভার্সিটির গবেষনাকর্মী
- ট্রপিকাল মেডিসিন রিসার্চ ইন্সটিটিউট, জ্যামাইকার গবেষনাকর্মী

এই পদ্ধতিগুলোর ফলাফল উপস্থাপন বা প্রকাশের সময় আপনার বা আপনার শিশুর নাম এবং পরিচয় কোথাও প্রকাশ করা হবে না। আপনি যদি এই ফর্মটিতে সাক্ষর করেন, তবে আপনি তথ্য প্রকাশের অনুমতি দিতেছেন, অনুমোদিত গবেষকবৃন্দ এবং নিরাপত্তা কমিটি, আইসিডিডিআর,বি ইথিকাল রিভিউ কমিটি, নীতিনির্ধারক কর্ত্রীপক্ষ (বাংলাদেশ এবং আমেরিকা উভয়পক্ষ), গবেষণার

অর্থ যোগানদাতা , সিনপস ( যারা গবেষনার তথ্য সংরক্ষন ও বিস্লেষনে গবেষকদের সহায়তা দিয়ে থাকে) এবং যারা দায়িত্তে আছেন, এবং অন্যান্য গবেষণা প্রতিষ্ঠান। এই অনুমতির কোন মেয়াদউত্তীর্ণ তারিখ নেই। যদি আপনি নিজের অনুমতি প্রত্যাহার করেন এবং এই চুক্তিটি শেষ করার সিদ্ধান্ত নেন, তবে ডঃ রাশিদুল হকের সাথে নিম্নউল্লেখিত ঠিকানা/নাম্বারে যোগাযোগ করুন। তিনি বা তার কর্মীরা এই অনুমতি প্রত্যাহারের সিদ্ধান্ত লিখিতভাবে রাখতে সহায়তা করবেন। দয়া করে মনে রাখবেন যে, গবেষণায় ইতোমধ্যে যেসব তথ্য নেয়া হয়েছে তা ব্যাবহার করা হতে পারে।

আপনার গবেষণায় অংশগ্রহণ করা ঐচ্ছিক। তবে, এই ফর্মটিতে সাক্ষর না করলে আপনি এই গবেষণায় অংশগ্রহণ করতে পারবেন না।

**নমুনার ভবিষ্যৎ ব্যবহারঃ**

এই গবেষনা শেষে, সব নমুনা আইসিডিডিআর,বিতে ৫ বছরের জন্য সংরক্ষন করা হবে। আপনি রাজী থাকলে, এই নমুনা আমরা ভবিষ্যতে অন্য গবেষণার কাজে ব্যাবহারের জন্য সংরক্ষন করবো। যদি আমরা বা আমাদের অন্য কোন সহযোগী প্রতিষ্ঠান গবেষণাটি করেন, সেক্ষেত্রে তখন যথাযথ কত্রিপক্ষের কাছ থেকে অনুমতি নেয়া হবে। ভবিষ্যতে এই নমুনা ব্যাবহার করা হলে, আপনার শিশুর ব্যাক্তিগত গোপনীয়তা রক্ষা করা হবে। যদি আপনি আপনার শিশুর নমুনা ভবিষ্যতে গবেষণা কাজে ব্যাবহারের জন্য সংরক্ষনের অনুমতি প্রদান করেন কিন্তু পরবর্তীতে আপনার সিদ্ধান্ত পরিবর্তন করেন, আপনি আমাদের সাথে যোগাযোগ করলে আমরা নমুনা নস্ট করে ফেলবো। যদি আপনি সিদ্ধান্ত পরিবর্তন না করেন, তাহলে আমরা আপনার শিশুর নমুনা ৫ বছর পর্যন্ত সংরক্ষন করবো। দ্রষ্টব্য, নমুনা বিশেষ পরীক্ষার জন্য বাংলাদেশের বাইরে সহযোগীদের কাছে পাঠানো হতে পারে।

**তথ্যের ভবিষ্যৎ ব্যবহারঃ**

আপনার এবং আপনার শিশুর সম্পর্কিত তথ্য শেয়ার করা হবে নীতিনির্ধারক কর্তৃপক্ষ সহ আইসিডিডিআর,বি ইথিকাল রিভিউ কমিটি (ERC), ইন্সটিটিউশনাল রিভিউ বোর্ড (ERB) অকল্যান্ড ইউনিভার্সিটি, বোস্টন চিলড্রেন হসপিটাল, ট্রপিকাল মেডিসিন রিসার্চ ইন্সটিটিউট, জ্যামাইকা, এবং গবেষণার অর্থ যোগানদাতা এবং যারা দায়িত্বে আছেন। গবেষকগণ ভবিষ্যতে গবেষণার উদ্দেশ্যে তাদের বিবেচনার ভিত্তিতে অন্যান্য গবেষকদের সাথে তথ্য এবং ডাটা ভাগ করে নিতে চাইতে পারেন। আমাদের স্টাডি ডাটা BCH এর কোর এর সহযোগীদের সাথে ভাগ করে নেওয়া হবে। আমরা আমাদের গবেষনা ডাটা সনাক্তকরনসহ BCH এ পাঠাবো, সেটা BCH REdCap ডাটাবেজ এ কাজ করবে। ফান্ড পাওয়ার জন্য Wellcome Leap এর সাথে আমাদের ডেটা হলো একটা চুক্তি, যেটা আমাদের গবেষনার প্রতিটা পুথক ব্যক্তির উপরে পরিচালিত পরিমাপের ডেটার উপরে ভিত্তি করে Leap consortium এর সাথে শেয়ার করা হবে। এবং sponsor required synapes (Maintain by sage Binetworks; synapes.org; WIRB#20112068) এর মাধ্যমে সহজে পাওয়া যাবে। BCH এ এই ডাটা আন্তত ৭ বছর সংরক্ষন করা যাবে, কিন্তু প্রয়োজন হলে বেশী সময় রাখা সম্ভব। এটা স্পন্সরদের চাহিদা অনুযায়ী ডাটার কাচের প্রবাহ। আমাদের নিকট থেকে ডেটা গ্রহনের পরে BCH সেটা এনালাইসিসের প্রক্রিয়ায় সাহায্য করবে।

**অংশগ্রহণ না করা এবং প্রত্যাহার করার অধিকারঃ**

গবেষণায় অংশগ্রহণ বিষয়টি ঐচ্ছিক এবং আপনি চাইলে অংশগ্রহণ নাও করতে পারেন অথবা যেকোন সময় নাম প্রত্যাহার করতে পারেন, যার জন্য আপনাকে কোন ক্ষতিপূরণ দিতে হবে না বা আইসিডিডিআর,বি হতে চিকিৎসা গ্রহণেও কোন সমস্যা হবে না।

# মায়ের নিজের ও তার ১ বছর বয়সী শিশুর সম্মতিপত্র

**SID:**

| | | |
|---|---|---|
| **Protocol No. PR-21084** | **Version No. .3.00** | **Date:**<br>**Date:,12th June' 2022** |

**Protocol Title: Multidimensional evaluation of the early emergence of executive function and emotional regulation in young children in Bangladesh using nutritional and psychosocial intervention: A Pilot study**

**Investigator's name: Dr. Rashidul Haque**

**Organization: International Centre for Diarrhoeal Disease Research, Bangladesh (icddr,b)**

আপনি যদি আপনার শিশুকে আমাদের গবেষণায় নাম লেখানোর জন্য আমাদের প্রস্তাবে সম্মত হন তবে দয়া করে নীচের বাক্সগুলোতে √ (চিহ্ন) দিন এবং শেষে নির্দিষ্ট স্থানে স্বাক্ষর / বাম বৃদ্ধা আঙুলের ছাপ দিন:

| **পয়েন্ট** | **স্টাটাস** |
|---|---|
| আমি পড়েছি / গবেষণা কর্মীরা এই গবেষণার সমস্ত তথ্য ভার্সন 3.0, ,12th June' ২০২২ থেকে পড়ে শোনায়, তারা আরো প্রশ্ন জিজ্ঞাসা করার ও এই গবেষণা সম্পর্কে আলোচনা করার সুযোগ দেয় এবং তাদের কাছ থেকে সন্তোষজনক উত্তর পাই। | হ্যাঁ ☐ না ☐ |
| আমি কোন কারণ ছাড়াই এই গবেষণা থেকে বের হয়ে আসতে পারি। | হ্যাঁ ☐ না ☐ |
| আমি বুঝলাম যে আমার দেওয়া সকল তথ্য গোপন থাকবে। | হ্যাঁ ☐ না ☐ |
| আমি গবেষণা কর্মীকে প্রতিটি নির্ধারিত ফলোআপ ভিজিট এবং হোম ভিজিটে আমার এবং আমার সন্তানের কাছ থেকে তথ্য সংগ্রহের অনুমতি দেওয়ার বিষয়ে সম্মত হই। | হ্যাঁ ☐ না ☐ |
| আমি সম্মতি দিচ্ছি যে, এই গবেষণা সমীক্ষা থেকে প্রাপ্ত তথ্য আইসিডিডিআর, বি, বোস্টন চিলড্রেন হাসপাতাল, মার্কিন যুক্তরাষ্ট্র, অকল্যান্ড বিশ্ববিদ্যালয়, নিউজিল্যান্ড এবং ট্রপিকাল মেডিসিন রিসার্চ ইনস্টিটিউট, জামাইকা, সিনপস ( যারা গবেষনার তথ্য সংরক্ষন ও বিস্লেষনে গবেষকদের সহায়তা দিয়ে থাকে) দ্বারা সংরক্ষণ করা হবে এবং ভবিষ্যতে অশনাক্তকারী অংশগ্রহণকারী হিসাবে পাবলিক ডেটাবেজে অন্তর্ভুক্ত থাকতে পারে । অশনাক্তকরণের অর্থ হলো আমি এবং আমার শিশুর নাম দ্বারা বা ডাটাবেসে থাকা অন্যান্য সনাক্তকারী চিহ্ন দ্বারা সনাক্ত করা যাবে না। আমার পুরো নাম বা কোনও ঠিকানার বিস্তারিত কোথাও অন্তর্ভুক্ত করা হবে না। প্রকাশিত তথ্যগুলি এই গবেষণায় আমার বা আমার সন্তানের অংশগ্রহণ সনাক্ত করবে না। আমি নির্দিষ্ট ব্যক্তিদের আমার তথ্য পাওয়ার অনুমতি দিচ্ছি। | হ্যাঁ ☐ না ☐ |
| আমি আমার সন্তানের কাছ থেকে ২-৩ মিলি রক্ত, মল, মুখগহবর থেকে মিউকাসের নমুনা দিতে সম্মত হয়েছি। | হ্যাঁ ☐ না ☐ |
| আমি আমার কাছ থেকে ৫ মিলি রক্ত, মল, মুখগহবর থেকে মিউকাসের নমুনা দিতে সম্মত হয়েছি। | হ্যাঁ ☐ না ☐ |
| আমার আর আমার সন্তানের কাছ থেকে যে বেনামে রক্ত, মল, মুখগহবর থেকে মিউকাসের নমুনা সংগ্রহ করা হয়েছিলো সেই নমুনাগুলি বিশ্লেষণের জন্য বিদেশে পাঠানোর বাপারে সম্মত হয়েছি। | হ্যাঁ ☐ না ☐ |
| আমার সন্তানকে পুষ্টির পরিপূরক খাবার এবং মানসিক উদ্দীপনা দিতে সম্মত আছি (যদি আমার শিশু অপুষ্টিতে ভুগে) | হ্যাঁ ☐ না ☐ |
| আমি আমার সন্তানের বুদ্ধি বিকাশের পরীক্ষা (এক্সিকিউটিভ ফাংশন, ইমোশনাল রেগুলেশন, এফএনআইআরএস, ইইজি) করতে সম্মত আছি | হ্যাঁ ☐ না ☐ |
| আমি আমরি শিশূর বিকাশ মূল্যায়নের জন্য LENA রেকর্ডিং, Bayley-4 assessment, পিতামাতা ও শিশুর পরস্পরিক প্রতিক্রিয়া (প্যারেন্ট চাইল্ড ইন্টার‍্যাকশন) রেকর্ডিং এবং প্রশ্নপত্রের কাজ সম্পন্ন করতে সম্মত আছি। | হ্যাঁ ☐ না ☐ |

| | |
|---|---|
| নৈতিকভাবে অনুমোদিত গবেষণা তে আমি এবং আমার সন্তানের তথ্য এবং নমুনাগুলি সংরক্ষণ এবং ভবিষ্যতে ব্যবহারে সম্মত আছি। | হ্যাঁ ☐ না ☐ |
| আমি ভবিষ্যতে গবেষণা সম্পর্কিত তথ্যের জন্য যোগাযোগ করাতে সম্মত আছি এবং আমি ভবিষ্যতে গবেষনা কাজের জন্য আমার শিশুর ছবি ব্যবহারের জন্য সম্মত আছি। | হ্যাঁ ☐ না ☐ |
| আমি অনুধাবন করতে পারলাম যে, গবেষণাতে আমার এবং আমার সন্তানের চিকিৎসার তথ্য এবং সংগ্রহ করা তথ্য পৃষ্ঠপোষক ব্যক্তি এবং নিয়ন্ত্রক কর্তৃপক্ষ দ্বারা অনুসন্ধান করা হতে পারে, যেখানে এটি আমার এবং আমার সন্তানের এই গবেষণায় অংশ নেওয়ার ক্ষেত্রে প্রাসঙ্গিক। আমি সেই নির্দিস্ট ব্যক্তিদের আমার তথ্য পাওয়ার অনুমতি দিয়েছি। | হ্যাঁ ☐ না ☐ |
| আমি এই গবেষণায় অংশ নিতে সম্মত আছি। | হ্যাঁ ☐ না ☐ |

________________________________ ______________

অংশগ্রহণকারীদের স্বাক্ষর বা বাম বৃদ্ধা আঙুলের ছাপ তারিখ (দিন/মাস/বছর)

________________________________ ______________

মাতা-পিতা / অভিভাবক / উপস্থিতির স্বাক্ষর বা
বাম বৃদ্ধা আঙুলের ছাপ তারিখ (দিন/মাস/বছর)

________________________________ ______________

সাক্ষীর স্বাক্ষর তারিখ (দিন/মাস/বছর)

________________________________ ______________

গবেষক বা তার প্রতিনিধি স্বাক্ষর তারিখ (দিন/মাস/বছর)

**যোগাযোগের জন্য**:
আপনার যদি কোনও প্রশ্ন থাকে তবে আপনি এখন বা যে কোনও সময় নীচে উল্লিখিত কর্মীদের কাছে জিজ্ঞাসা করতে পারেন:

| যোগাযোগের উদ্দেশ্য | নাম এবং ঠিকানা | যোগাযোগের ঠিকানা |
|---|---|---|
| স্টাডি সম্পর্কিত যে কোনও প্রশ্নের জন্য, বা কোনও সমস্যার জন্য | ডা: মাসুদ আলম | ঠিকানা: বাড়ি-২৮, এভিনিউ-১, কলসী রোড, মিরপুর-১২, ঢাকা-১২১৬. মোবাইল নংঃ ০১৭১১৫৭০৫৫০ (৭/২৪ ঘন্টা খোলা থাকবে) |
| | ডা: রাশিদুল হক। | ঠিকানা: প্যারাসাইটোলজি ল্যাবরেটরি, আইসিডিডিআর,বি, মহাখালী, ঢাকা-১২১২,মোবাইল: ০১৭১৩০৯৩৮৫৯ (সকাল ৯:০০ টা থেকে বিকাল ৫:০০ টা পর্যন্ত) |
| অধিকার বা সুযোগ সুবিধা জানতে বা কোনও অভিযোগ বা অসন্তুষ্টি জানাতে | এম এ সালাম খান (আইআরবি সমন্বয়কারী) | আইআরবি সচিবালয়, গবেষণা প্রশাসন, আইসিডিডিআর,বি, মহাখালী, ঢাকা-১২১২<br>ফোনঃ (+৮৮-০২) ৯৮২৭০৮৪ বা মোবাইল: ০১৭১১৪২৮৯৮৯ |

আপনার সহযোগিতার জন্য ধন্যবাদ। স্বাক্ষরকৃত সম্মতি পত্রের একটি অনুলিপি আপনাকে দেওয়া হবে।

## Information Sheet for mother with her 3-year-old child

| Protocol No. PR-21084 | Version No. 3.00 | Date: 12th June'2022 |
|---|---|---|

**Protocol Title: Multidimensional evaluation of the early emergence of executive function and emotional regulation in young children in Bangladesh using nutritional and psychosocial intervention: A Pilot study**

**Investigator's name: Dr. Rashidul Haque**

**Organization: International Centre for Diarrhoeal Disease Research, Bangladesh (icddr,b)**

**Purpose of the research:** To determine the effect of nutritional intervention for improvement of cognition and emotional regulation among the children in Bangladesh where malnutrition and social adversities are common

**Background** (brief introduction of the issue and the need for/ importance of the research)
We are conducting a study to understand the problem of malnutrition and poor cognitive outcomes in children of Bangladesh.Malnutrition affects around 47 million children under 5 years of age in low- and middle-income countries annually and among then more than 20 million death occurs, others suffer long term cognitive and behavioural impairment. Malnutrition causing significant number of deaths of under 5 years child globally. It is a large problem in Bangladesh where 40% of under-fives have moderately acute malnutrition. Malnutrition is further worsened by poor diet. Inadequate feeding practices leading to deficiencies in vitamin and minerals. Studies show that, during infancy and early childhood, nutrition is essential as these are the crucial period for the formation of the brain, building the foundation for the development of cognitive, neurological and socio-emotional skills throughout childhood and adulthood.

**Why invited to participate in the study?**
Researchers from icddr,b, the University of Auckland, New Zealand, Boston Children Hospital, USA and Tropical Medicine Research Institute, Jamaica are jointly conducting this research study in your community to understand the effect of nutritional intervention on the cognition of moderate acute malnourished chidren at 3 years ±2m(34m-38m) old . Total 70 children at 3 years of old with WLZ/WHZ <-2 and ≥-3 z-score, and/or MUAC <12.5 and ≥11.5 cm having stable moderate acute malnutrition and 70 mothers will be enrolled from this community. We invite you to help us in our efforts through your child's participation in this study, because you have 3 years ±2m(34m-38m) of old child with stable moderate or severe malnutrition and you live in this community where malnutrition and social adversities are common. Research ethics committees at icddr,b and IRBs of University of Auckland, New Zealand, Boston Children Hospital, USA and Tropical Medicine Research Institute, Jamaica have approved this research study.

**Methods and procedures [What is expected from the participants of the research study?]**
You and your child will be requested to participate in the study. If you agree to participate; you are agreeing to provide information about you and your child's medical and personal information as well as, to allow the collection of a stool, blood sample, buccal scrab and body measurement such as height, weight from your child and blood, stool and body measurement such as height, weight from you as per protocol for one time. Your child is also invited to participate Executive function, Emotional regulation, fNIRS , BAYLEY 4, LENA, PCI, Behavioural assessment questionnaires and EEG for one time after enrolment. After obtaining the signed consent from you we will start the study activities.

**Screening and enrolment:**
Screening and enrolment will occur at the home/ clinic by our trained study team. The team will review the eligibility criteria to confirm your child is eligible. If your child is eligible then team will explain the study to you and request to participate. If you sign this consent form for you and for your child then both of you will be enrolled in the study and we will collect birth date, sex, information about your family (occupation, income, education, family size, etc). We will also collect medical information, if it is needed. This enrolment procedure will take approximately 1 hour. Your participation duration will be 3 months from enrolment.
We will provide you the contact information and location of local clinic to visit our clinic.

**Procedures and sample collection for children:**

- Stool collection: Within two weeks of enrolment
- Blood collection: Within two weeks of enrolment
- Anthropometry (height, weight, MUAC, Head Circumference): Within two weeks of enrolment
- Buccal Scrab: Within two weeks of enrolment
- Neurodevelopmental assessment (ER, ER, fNIRS, EEG, BAYLEY 4, LENA, PCI, Behavioural assessment questionnaires): At enrollment (2 months window)

**Behavioural Measures (Executive Functions/Emotional regulation):**
Executive functions are the processes involved in conscious control of thought and action including inhibitory control, planning and cognitive flexibility. Emotional Regulation is an integral part of self-regulation, which is a complex concept that measure cognitive flexibility, working memory and inhibition control skills of executive function.

This study involves two 2-3-hour sessions at the icddr,b Mirpur clinic for Neuro developmental assessment (Executive function, Emotional regulation, fNIRS,BAYLEY 4, LENA, PCI, Behavioural assessment questionnaires, EEG) . The sessions will be scheduled on separate days within two weeks of each other at a time that is convenient for you and your child. Most of tasks of executive function and emotional regulation are direct interactions between the experimenter and the child, which will be recorded in video camera for video-scoring. These activities will be conducted in a playful manner and not as a work-oriented activity.

Bayley-4
Bayley Scales of Infant and Toddler Development is an extensive formal developmental assessment tool for diagnosing developmental delays in early childhood. BAYLEY 4 assess development in children of 1-42 months old in 5 domains : cognition, motor, language, socio-emotional, and adaptive behavior. Baley-4 will be administered to all children. At enrolment time point 3years±2m (34m-38m)

LENA
Language Environment Analysis (LENA) LENA recorder and software measure the frequency of vocalization/verbalizations and conversational turns in children to see the influence of language input at different crucial points in early development. . Recording data at multiple time points also gives us a measure of the consistency of the language environment. . At enrolment time point 3years±2m (34m-38m)

## Parent-Child Interaction

A 10-15-minute interaction between parent and child will be collected. at enrolment time point 3years±2m (34m-38m) You (parents) will be asked to engage your child in play or conversations in the presence and absence of toys and books. The child will also play with the toys/books while the parent completes short surveys or watches a short video. This session will be recored through video recording.

**NIRS Procedure**:

During one of sessions, we will record your child's brain activity using functional near-infrared spectroscopy (fNIRS). fNIRS measures and records the changes in the levels of oxygen in the blood by shining a near-infrared light into your child's head. We will be able to identify which areas of your child's brain are actively responding to the changes in images shown on the computer monitor.

For recording these changes, we use a computer which is attached to sensors and probes. Prior to placing the probes, we will measure your child's head with a measuring tape for proper fitting of the fNIRS sensors. These sensors rest on a headband, which is then placed around your child's head and adjusted for a tight fit.

While your child is wearing the headband, we will your child to watch videos of Resting state, Habituation & Novelty Detection and working memory tasks aim to assess child's Executive Function There will also be sounds playing during some of the images/videos.

Finally, we will ask many different questions about your child's development (motor skills, speech, vision, etc.) in order to test for developmental disabilities. We will also ask you some questions from some questionnaires for behavioral assessment of your child and you which will take approximately 1.30 hours. We will also ask you some questions regarding impact of COVID 19 All information provided will be confidential, and if anything is concerning, further testing will be conducted, and referrals for care will be made free of charge. All of these procedures are completely safe and will not hurt your child. You will be with your child at all times.

**EEG Procedure**:

During the other visit to the clinic to perform EEG, we will record your child's brain activity using a small cap that is made of stretchy material. Each cap has many sponges on it and inside each sponge is a small recording sensor. We soak the caps in a warm salt water solution so the sponges get soft before we put the cap on the child's head. In this task, also before starting the session we need to measure the head circumference by measuring tape to get the appropriate cap size. As your brain is working, it is constantly giving off small electrical signals, which travel out to the scalp where we can pick them up with the special sensors. We will show a series of faces and patterns and record your child's brain activity during these tasks. Then, we will do the eye tracking measures. Your child will sit on your lap and watch a video of bright looming circles while we set up the eye-tracking equipment. The eye tracker is made up of a special computer monitor that has a set of infrared cameras built into the edges of the screen. Once calibrated, these cameras will follow eye movements and tell us exactly where on the screen your child is looking as he/she is watching the pictures. At your first visit we will have your child look at videos auditory, baseline, working memory, disengagement, flanker and VEP tasks. All these tasks will be used to assess Executive Function of a child by measuring emotional control, recognition, working memory, inhibitory control, attention, anticipation and cortical processing of vision while we record your child's brain activity. Next, we will record your child's eye movements in response to a series of pictures on a computer screen.

In addition, a digital video will be recorded to help the experimenter know when to present pictures to your child and aid data analysis. Your child's name will not be associated with the video recording and the file will be accessible only to the investigators of this study.

**None of neuro developmental tasks are invasive or harmful**

**Feed for 3 years MAM children (outcome reference group)**

After completion of all baseline work your child will receive. We will be given Nutriset's Plumpy'Sup™, RUSF for 3 years MAM children (outcome reference group)

After completing the baseline assessment, we will provide your children weekly basis for daily consuming of 1 packet of RUSF (Nutriset's Plumpy'Sup™, RUSF) for 2 months. **Each individual will be provided 1sachet of Nutriset's Plumpy'Sup™, RUSF** which contains **100 g of feed which is about 540 kcal** and each child will be provided 1 sachet per day for 2 months. We will also collect anthropometry before and after feed and then refer to NRU at icddr,b or local facilities if needed.

As this is a crucial period of growth and cognitive development, it is necessary to correct the nutritional status of these young groups as early as possible.

**Procedures and sample collection for you (mother):**

- Stool collection: 10 gm once within 2 weeks of enrolment
- Blood collection: 5ml once within 2 weeks of enrolment
- Buccal swab sample: Once within 2 weeks of enrolment
- Anthropometry (height and weight): once within 2 weeks of enrolment

**Risk and benefits**

What are the risks from participating in the study?

Sometimes things happen to the research participants in research studies that may hurt them or make them feel bad. These are called risks. The risks of participating in this study include risks caused by intervention, neuro cognitive assessments and sample collection.

Blood collection: Mild pain, discomfort, bleeding or bruising, or get an infection (which is extremely rare) where the needle or lancet is inserted. To minimize these risks, only trained, experienced staff will draw blood, and disposable materials will be used. if an infection results from a blood draw, we will provide any necessary treatment at no cost to you

Stool collection: We do not anticipate any risks from collecting stool.

Buccal swab: We do not anticipate any risks from collecting buccal swab

Neuro cognitive assessment: There are no major risks involved with those assessments. The number of tests during each day may cause some tiredness, but you and your children are allowed to take as many breaks as you need. All tests and procedures remain outside the child's body, pain free, and contain no bad effects.

If anything from the tests seems unusual or concerning (like abnormal seizure activity on EEG), child will be referred to the appropriate specialists in Bangladesh, and you will be contacted with information on what to do next. The study will cover the cost of transportation to the specialist, assistance with the consultation and referral process, but we will not cover the cost of medical care beyond primary medical care.

**Benefit**
You and Your child will receive free, high-quality primary care, and referrals for any illness through our study for the duration of participation.

**Principle of compensation**
There is no direct compensation for participation in this study, but in the case of any concerning information discovered, referrals to professionals will be provided free of charge. In addition, transportation to the clinic and food at the clinic will be provided.

**Privacy, anonymity and confidentiality**
We will keep all information collected from you and your child confidential and locked in a secure place under the responsibility of the study investigators. Data will be saved in secure servers at icddr,b and Boston Children's Hospital. Biological samples will be stored securely without identifying information at icddr,b. Neuro imaging testing will be done in private rooms, and subjects will be coded by a de-identified number. In addition, staff and researchers have completed the Course in The Protection of Human Research Subjects.

In general, anyone who is involved in this research, including those funding and regulating the study, may see the data, including information about you and your child, photos and videos. For example, the following people might see information about you and your child:

- Research staff at icddr,b
- Research staff at Boston Children's Hospital
- Research staff at Auckland University
- Research staff at Tropical Medicine Research Institute, Jamaica

Your/your child's name and identity will not be disclosed in the process of analyzing, presenting or publishing the results of these procedures.

If you sign this form, you have given us permission to release information to authorized researchers and the safety committees, icddr,b Ethical Review Committee, regulatory authorities (both in Bangladesh and the United States), the study sponsor , Synopse ( a research data sharing and collaboration platform) and designees, and other research organizations. There is no expiration date to this permission. If you decide to withdraw your permission and end this agreement, please contact Dr. Rashidul Haque at the address/number listed below. He or his staff will help you document in writing your decision to withdraw this permission. Please note that any study information already obtained will continue to be used.

Your participation in this research is voluntary. However, you will not be able to participate in this study if you do not sign this form.

**Future use of samples:**
At the end of the study, all of the specimens will be stored at icddr,b for 5 years . If you agree, we will store these samples to be used in the future for other research purposes. If such research is conducted by us or by our collaborators, appropriate approvals from respective authorities will be secured at that time. If samples be used in the future, your and your child's privacy and anonymity will be maintained. If you consent to having your and your child's samples saved to be used for future research, but change your mind later, you may contact us and the samples will be destroyed. If not, we will store your child's specimens for 5 years. Of note, samples may be sent to collaborators outside of Bangladesh for specialized testing.

**Future use of information**

Information about you and your child may be shared with regulatory authorities including but not limited to the Ethical Review Committee (ERC) at the icddr,b, the Internal Review Board (IRB) at University of Auckland, Boston children hospital, Tropical Medicine Research Institute, Jamaica and the study sponsor and designees.  Investigators may choose to share information and data with other researchers at their discretion for the purpose of future research. Our study data will be shared with collaborators BCH is the data core, and we will be sending BCH our research data, including identifiers, which they will host in BCH REDCap database. An agreement with the Wellcome Leap foundation to receive funding is that our data, based on the data collected from all administered measures from individual participants in our study, will be shared with collaborators in the Leap consortium and will be available to Leap via the sponsor required Synapse (Maintained by Sage Bionetworks; synapse.org; WIRB#20112068). The data at BCH would be stored for at least 7 years after, but possibly even longer if needed. This is a sponsor-required data workflow. BCH will aid in processing and analysis of the data received from us.

**Right not to participate and withdraw**

Participation in this study is voluntary and you can choose to not participate, or withdraw at any point during the study without any penalty or loss of care.

# <u>Consent Sheet for mother with her 3-year-old child</u>

**SID:**

| **Protocol No. PR-21084** | **Version No. 3 .00** | **Date:12th June'2022** |
|---|---|---|

**Protocol Title: Multidimensional evaluation of the early emergence of executive function and emotional regulation in young children in Bangladesh using nutritional and psychosocial intervention: A Pilot study**

**Investigator's name: Dr. Rashidul Haque**

**Organization: International Centre for Diarrhoeal Disease Research, Bangladesh (icddr,b)**

If you agree to our proposal for enrolling you and your child in our study, please put √ mark on appropriate box(es) of the following and finally sign / left thumb print on the specified place for you:

| Points | Status |
|---|---|
| I have read out / study staff has read out the all information from this participants information sheet Version .3 0, Dated: 12th June' 2022about the study, have had the opportunity to ask questions, discuss the study, and received satisfactory answers | Yes ☐ No ☐ |
| I understood that I am free to leave the study without giving any reason | Yes ☐ No ☐ |
| I understood that the information that I gave will be confidential | Yes ☐ No ☐ |
| I agree to allow the study team to collect information from me and my child at every scheduled visit | Yes ☐ No ☐ |
| I understood that the Information from this research study will be retained by icddr,b , Boston Children Hospital, USA, Auckland University, New Zealand and Tropical Medicine Research Institute, Jamaica, Synopse ( a research data sharing and collaboration platform) and in the future may be included in a de-identified public use database. De-identified means that I and my child will not be individually identified by name or other personal identifiers in the database. My full name or any address details will not be included. Information released will not identify me or my child's participation in this research study.<br>I am giving permission for those individuals to have access to my records. | Yes ☐ No ☐ |
| I agree to to allow the study team to collect all anthropometric data (Height/length, weight, MUAC, Head Circumference) of my child | Yes ☐ No ☐ |
| I agree to the collection of blood (2-3 ml), buccal swab, and stool from my child | Yes ☐ No ☐ |
| I agree to the collection of anthropometry (height, weight) , blood (5 ml), buccal swab and stool sample from me | Yes ☐ No ☐ |
| I agree that anonymised blood, buccal swab and stool (those collected from me and my child) samples can be sent overseas for analysis | Yes ☐ No ☐ |
| I agree to perform neurocognitive tests (Executive Function, Emotional Regulation, FNIRS, EEG) to my child | Yes ☐ No ☐ |

| | |
|---|---|
| I agree to perform LENA recording, Bayley-4 assessment, parent-child interection recording and questionnaires for my child developmental assessment | Yes ☐ No ☐ |
| I agree to storage and future use of me and my child's data and samples by ethically approved studies | Yes ☐ No ☐ |
| I agree for future use of photos of my child for study purpose. | Yes ☐ No ☐ |
| I agree to being contacted in the future for studies related to this study | Yes ☐ No ☐ |
| I understand that relevant sections of my medical notes and data collected during the study may be looked at by individuals from the sponsor and by regulatory authorities, where it is relevant to my taking part in this research. I give my permission for those individuals to have access to my records. | Yes ☐ No ☐ |
| I agree to participate in to this study | Yes ☐ No ☐ |

__________________________________ ________________

Signature or left thumb impression of participant Date (dd/mmm/yyyy)

_______________________________ ___________________

Signature or left thumb impression of Parent/ Guardian/ Attendant Date (dd/mmm/yyyy)

_____________________________________ ___________________

Signature of the witness Date (dd/mmm/yyyy)

_____________________________________ __________________

Signature of the PI or his/her representative Date (dd/mmm/yyyy)

**Communication:**
If you have any question, you can ask me right now or at any time later to the below mentioned personnel:

| Purpose of contact | Name and address | Address for communication |
|---|---|---|
| For any question related to the study, or any problem | Dr. Masud Alam | Address: House no 28, Avenue 1, Kalsi Road, Mirpur-12, Dhaka-1216, Mobile No. 01711570550 (to be open 7/24 hours) |
| | Name of PI: Dr. Rashidul Haque. | Address: Parasitology Laboratory, IDD, icddr,b , Mohakhali, Dhaka-1212 , Mobile: 01713093859 (9:00 am to 5:00 pm) |
| To know the rights or benefits or to log any complain or dissatisfaction | M A Salam Khan (IRB Coordinator) | IRB Secretariat, Research Administration, icddr,b, Mohakhali, Dhaka-1212 Phone: (+88-02) 9827084 or Mobile: 01711428989 |

Thank you for your cooperation.
A Copy of singed consent will be given to you.

## Information Sheet for mother with her 3-year-old child

| Protocol No. PR-21084 | Version No. 3.00 | Date: 12th June'2022 |
|---|---|---|

**Protocol Title: Multidimensional evaluation of the early emergence of executive function and emotional regulation in young children in Bangladesh using nutritional and psychosocial intervention: A Pilot study**

**Investigator's name: Dr. Rashidul Haque**

**Organization: International Centre for Diarrhoeal Disease Research, Bangladesh (icddr,b)**

**গবেষণার উদ্দেশ্যঃ**
এই গবেষণার উদ্দেশ্য হলো বাংলাদেশের শিশুদের বুদ্ধির বিকাশ এবং আবেগীয় নিয়ন্ত্রনের উন্নয়নের জন্য পুষ্টি খাওয়ানোর প্রভাব নির্ধারণ করা যেখানে অপুষ্টি এবং সামাজিক প্রতিকুলতা বিদ্যমান।

ভূমিকা(সমস্যার সংক্ষিপ্ত ভুমিকা এবং এই গবেষণার গুরুত্ত / প্রয়োজনীয়তা)ঃ

বাংলাদেশের শিশুদের অপুষ্টিগত সমস্যা এবং দুর্বল বুদ্ধির বিকাশ অনুধাবন করার জন্য একটি গবেষণা পরিচালনা করা হচ্ছে । এখানে নিম্ন ও মধ্যবিত্ত পরিবারের পাঁচ বছরের নিচের শিশুদের মধ্যে বার্ষিকহারে আনুমানিক ৪৭ মিলিয়ন শিশুর উপর অপুষ্টিগত প্রভাব পড়ে এবং তাদের মধ্যে ২০ মিলিয়নের অধিক শিশু মারা যায় আর অন্যরা দীর্ঘমেয়াদি বুদ্ধির বিকাশ এবং আচরণগত দুর্বলতায় ভুগে।বিশ্বব্যাপী ৫ বছরের নিচের উল্লেখ্যযোগ্য সংখ্যক শিশু মারা যায় অপুষ্টির কারনে। ইহা বাংলাদেশের একটি বড় সমস্যা যেখানে ৫ বছরের ৪০% শিশুর মাঝারিভাবে তীব্র অপুষ্টি রয়েছে। দুর্বল খাদ্যাভাস দ্বারা এই অপুষ্টি পরিস্থিতির আরও অবনতি হয়।অপর্যাপ্ত খাদ্যগ্রহন ভিটামিন ও খনিজ পদার্থ ঘাটতির দিকে পরিচালিত করে।গবেষণায় দেখা গেছে যে, শৈশবকাল ও শৈশবকালীন সময়ে পুষ্টি গ্রহন অপরিহার্য কারন মস্তিষ্ক গঠনের জন্য ইহা গুরুত্বপূর্ণ সময়কাল। বুদ্ধির বিকাশ,শৈশব ও কৈশোরকালে স্নায়বিক এবং সামাজিক-মানসিক দক্ষতার ভিত্তি তৈরি করার জন্য পুষ্টি অপরিহার্য।

**কেন আপনাকে গবেষণায় অংশগ্রহণের আমন্ত্রন জানানো হচ্ছে?**
আইসিডিডিআরবি,অকল্যান্ড ইউনিভার্সিটি নিউজিল্যান্ড ,বোস্টন চিলড্রেনস হসপিটাল আমেরিকা এবং ট্রপিকাল মেডিসিন রিসার্চ ইন্সটিটিউট, জ্যামাইকার গবেষকবৃন্দ যৌথভাবে এই গবেষণাটি পরিচালনা করছেন আপনাদের এলাকায় শিশুদের বুদ্ধির বিকাশের উপর পুষ্টি খাওয়ানোর প্রভাব দেখার জন্য মাঝারি তীব্র অপুষ্টির শিকার ৩ বছর বয়সী (±২ মাস(৩৪ মাস-৩৮ মাস)) শিশুদের উপরে।আপনাদের এলাকার সর্বমোট ৭০ জন ৩ বছর বয়সী শিশু (WHZ <-2 and ≥-3 z-score, and/or MUAC <12.5 and ≥11.5 cm) মাঝারি তীব্র অপুষ্টির শিকার এবং তাদের মায়েদের এই গবেষণায় অন্তর্ভুক্ত করা হবে।আপনার শিশুকে এই গবেষণায় অংশগ্রহণের জন্য আমরা আপনাকে আমন্ত্রন জানাচ্ছি কারন আপনি ৩ বছর বয়সী (±২ মাস(৩৪ মাস-৩৮ মাস))মাঝারি বা তীব্র অপুষ্টির শিকার একজন শিশুর অভিভাবক এবং এই এলাকায় বসবাস করছেন যেখানে অপুষ্টি এবং বিভিন্ন সামাজিক প্রতিকুলতা বিদ্যমান। আইসিডিডিআরবি র গবেষণা নীতি কমিটি এবং ইউনিভার্সিটি অফ অকল্যান্ড, নিউজিল্যান্ড র আইআরবি , বোস্টন চিলড্রেনস হসপিটাল আমেরিকা এবং ট্রপিকাল মেডিসিন রিসার্চ ইন্সটিটিউট, জ্যামাইকা এই গবেষণাটির অনুমোদন দিয়েছেন।

**পদ্ধতি ও প্রক্রিয়া (গবেষণায় অংশগ্রহনকারীদের কাছ থেকে কি প্রত্যাশা?)**
আপনি এবং আপনার শিশুকে এই গবেষণায় অংশগ্রহনের জন্য অনুরোধ জানাচ্ছি। আপনি যদি অংশগ্রহনে রাজি থাকেন,তাহলে,আপনি এবং আপনার শিশুর স্বাস্থ্য এবং ব্যক্তিগত তথ্য দিতে সম্মত আছেন এবং পাশাপাশি আপনার শিশুর মল, রক্তের নমুনা, মুখগহবর মিউকাসের নমুনা এবং শারীরিক পরিমাপ যেমন- উচ্চতা, ওজন সংগ্রহের অনুমতি দিচ্ছেন। আপনার শিশুকে আরও আমন্ত্রন জানানো হবে Executive Function/বুদ্ধি পরীক্ষা, আবেগীয় নিয়ন্ত্রন, fNIRS এবং EEG-তে অংশগ্রহন করার জন্য। এছাড়াও, আপনার নিকট থেকে সাক্ষরিত সম্মতিপত্র পাওয়ার পর আমরা গবেষণা কার্যক্রম শুরু করবো।

**<u>বাছাইকরন এবং অন্তর্ভুক্তিকরনঃ</u>**
আমাদের প্রশিক্ষনপ্রাপ্ত গবেষকদল দ্বারা বাসায়/ক্লিনিকে এই বাছাইকরন এবং অন্তর্ভুক্তিকরন করা হবে। আপনার শিশু অন্তর্ভুক্তিকরনের যোগ্য কি না নিশ্চিত করার জন্য গবেষকদল আপনাকে গবেষণা সম্পর্কে ব্যাখা করবে।যদি আপনার শিশু এই গবেষণায় অংশগ্রহণের যোগ্য হয় এবং আপনি আপনার নিজের এবং শিশুর জন্য সম্মতিপত্রে সাক্ষর করে থাকেন, তাহলে আপনারা উভয়ে এই গবেষণায় অন্তর্ভুক্ত হবেন এবং আমরা কিছু তথ্য বিশদভাবে সংগ্রহ করবো, যেমন- জন্মতারিখ, লিঙ্গ, আপনার পারিবারিক তথ্য( পেশা, আয় ,শিক্ষা, পরিবারের আকার, শিশুকে বুকের দুধ খাওয়ানোর সময়কাল, ইত্যাদি )। আমরা প্রয়োজনানুসারে স্বাস্থ্য তথ্যও সংগ্রহ করবো। এই অন্তর্ভুক্তিকরন সময়কাল আনুমানিক ১ ঘণ্টা হবে । আপনার অংশগ্রহণ অন্তর্ভুক্তির সময় থেকে ৩ মাস ব্যাপী চলবে।
আমাদের ক্লিনিকে আসার জন্য আমরা আপনাকে যোগাযোগ সংক্রান্ত তথ্য এবং স্থানীয় ক্লিনিকের ঠিকানা প্রদান করবো

**<u>সকল শিশুদের নমুনা সংগ্রহের প্রক্রিয়াঃ</u>**

- মল সংগ্রহঃ অন্তর্ভুক্তির ২ সপ্তাহের মধ্যে।
- রক্ত সংগ্রহঃ অন্তর্ভুক্তির ২ সপ্তাহের মধ্যে।
- শারীরিক পরিমাপ ( উচ্চতা,ওজন,MUAC, মাথার পরিধি)ঃ অন্তর্ভুক্তির ২ সপ্তাহের মধ্যে।
- মুখগহবর থেকে মিউকাসের নিঃসৃত নমুনাঃ অন্তর্ভুক্তির ২ সপ্তাহের মধ্যে একবার।
- নিউরোডেভেলপমেন্টাল মূল্যায়ন (ER,EF,fNIRS,EEG LENA রেকর্ডিং, বেইলী-৪ মূল্যায়ন, পিতামাতা ও সন্তানের পারস্পরিক মিথিস্ক্রিয়া রেকর্ডিং এবং আমার শিশুর বিকাশ্মুলক মূল্যায়নের জন্য প্রশ্নমালা): অন্তর্ভুক্তির সময় (২ মাসের উইন্ডো)

<u>আচরণগত পরিমাপসমূহ **(**এক্সিকিউটিভ ফাংশন **/**আবেগীয় নিয়ন্ত্রণ**)**ঃ</u>

এক্সিকিউটিভ ফাংশন হলো সচেতন ভাবে চিন্তা এবং কাজের নিয়ন্ত্রণ যার মধ্যে আছে দমনমূলক নিয়ন্ত্রণ, পরিকল্পনা এবং বুদ্ধিগত নমনীয়তা। আবেগীয় নিয়ন্ত্রণ হলো স-নিয়ন্ত্রণের একটি অবিচ্ছেদ্য অংশ, যা এমন একটি জটিল ধারনা যেটা এক্সিকিউটিভ ফাংশনের বুদ্ধিগত নমনীয়তা,কার্যকারী স্মৃতিএবং বাধা নিয়ন্ত্রণের দক্ষতা পরিমাপ করে। আইসিডিডিআরবি মিরপুর ক্লিনিকে এই নিউরো কগনিটিভ মূল্যায়ন (*Executive Function*/বুদ্ধি পরীক্ষা, আবেগীয় নিয়ন্ত্রণ, *fNIRS* এবং *EEG*, LENA রেকর্ডিং, বেইলী-৪ মূল্যায়ন, পিতামাতা ও সন্তানের পারস্পরিক মিথিস্ক্রিয়া রেকর্ডিং এবং আমার শিশুর বিকাশ্মুলক মূল্যায়নের জন্য প্রশ্নমালা)-র কাজগুলো ২-৩ ঘণ্টায় সম্পন্ন করা হবে। সেশনগুলি আপনার এবং আপনার শিশুর জন্য সুবিধাজনক যে কোনও একটি সময়ে দুই সপ্তাহের মধ্যে পৃথক দুই দিনে করা হবে। এই এক্সিকিউটিভ ফাংশন এবং আবেগ নিয়ন্ত্রন কার্যকলাপের বেশিরভাগই পরীক্ষক ও আপনার শিশুর মধ্যে সরাসরি ভাবের আদান প্রদান হবে যা ভিডিও ক্যামেরায় রেকর্ড করে স্কোরিং করা হবে। এই সমস্তকিছু শুধুমাত্র খেলাধুলার মাধ্যমে পরিচালিত হবে কোন কাজের মত করে নয়

<u>বেইলী **–** ৪</u>
বেইলী স্কেলস অফ ইনফ্যান্ট এন্ড টডলার ডেভেলপমেন্ট হল প্রাক শৈশবকালে বিকাশগত বিলম্ব নির্ণয়ের জন্য একটি ব্যাপক আনুষ্ঠানিক উন্নয়নমূলক মূল্যায়নের সরঞ্জাম। বেইলী – ৪ শিশুদের ১-৪২ মাসের মধ্যে তাদের বিকাশের ৫ টি দিক পরিমাপ করেঃ জ্ঞানীয়, পেশিসঞ্চালন,ভাষা,সামাজিক-আবেগীয় এবং অভিযোজিত আচরণ।বেইলী – ৪ সকল শিশুদের উপরে পরিচালনা করা হবে। অন্তর্ভুক্তির সময়ে ৩বছর বয়সে ±২ মাস(৩৪ মাস-৩৮ মাস)

<u>LENA</u>
ল্যাংগুয়েজ এনভায়রনমেন্ট এনালাইসিস (LENA) LENA রেকর্ডার এন্ড সফটওয়কারীশিশুদের প্রাথমিক বিকাশের বিভিন্ন গুরুত্বপূর্ণ সময়ে ভাষার ইনপুটের প্রভাব দেখতে শিশুদের কন্ঠসর/বাচ্যায়ন এবং

কথোপকথনের কম্পাঙ্ক বা ফ্রিকোয়েন্সি পরিমাপ করে। একাধিক সময়ে তথ্য রেকর্ড করার ফলে তা আমাদের ভাষা পরিবেশের সামঞ্জস্যের একটি পরিমাপ দেয়। অন্তর্ভুক্তির সময়ে ৩বছর বয়সে ±২ মাস(৩৪ মাস-৩৮ মাস)

পিতামাতা ও সন্তানের পারস্পরিক মিথস্ক্রিয়া

পিতামাতা ও শিশুর মধ্যে  পারস্পরিক মিথস্ক্রিয়ার ১০-১৫ মিনিটের একটি তথ্য সংগ্রহ করা হবে। অন্তর্ভুক্তির সময়ে ৩বছর বয়সে ±২ মাস(৩৪ মাস-৩৮ মাস)। খেলনা এবং বইয়ের উপস্থিতি বা অনুপস্থিতিতে আপনাকে (পিতামাতা) শিশুর সাথে খেলা অথবা কথাবার্তার মাধ্যমে তাকে ব্যস্ত রাখতে বলা হবে। পিতামাতা যখন কোন সংক্ষিপ্ত সার্ভে করবেন বা সংক্ষিপ্ত ভিডিও দেখতে থাকবেন তখন শিশুকে খেলনা/বই দিয়ে খেলতে দেয়া হবে। পুরো সেশনটি ভিডিও রেকর্ডারের মাধ্যমে রেকর্ড করে রাখা হবে।

NIRS পদ্ধতিঃ
একটি সেশনে, fNIRS (ফাংশনাল নেয়ার ইনফ্রারেড স্পেক্ট্রস্কপি) প্রযুক্তি ব্যাবহার করে আপনার শিশুর মস্তিষ্কের সক্রিয়তা লিপিবদ্ধ করবো। fNIRS আপনার শিশুর মাথায় একটি উজ্জ্বল লেজার লাইট/ রশ্মি প্রবেশ করিয়ে তার রক্তে অক্সিজেনের পরিমানের পরিবর্তন পরিমাপ করে এবং লিপিবদ্ধ করে রাখে। এর ফলে আমরা চিহ্নিত করতে সমর্থ হব যে কম্পিউটারের মনিটরের ছবি পরিবর্তনের সাথে সাথে আপনার শিশুর মস্তিষ্কের কোন অংশটি সক্রিয়ভাবে প্রতিক্রিয়া করে।
যেকোনো ধরনের পরিবর্তন লিপিবদ্ধ করে রাখার জন্য আমরা একটি কম্পিউটার ব্যাবহার করবো যেটা সেন্সরস এবং প্রবসের সাথে সংযুক্ত থাকবে। আপনার শিশুর মাথায় প্রবস/ শলাকা স্থাপন করার পূর্বে , পরিমাপক ফিতা দিয়ে শিশুর মাথা আগে মেপে নেয়া হবে যাতে fNIRS সেন্সরস / সংবেদকগুলো  সঠিক ভাবে লেগে থাকে।  সেন্সরস  /সংবেদকগুলো একটি মাথার বন্ধনীর উপর লাগানো থাকে ,যা পরে শিশুর মাথার চারপাশে যথাযথ এবং শক্ত করে লাগিয়ে দেয়া হবে।

যখন শিশুর মাথায় বন্ধনী লাগানো থাকবে ,তখন শিশুকে মনিটরে চলমান ভিডিও দেখানো হবে বিশ্রামরত অবস্থা, অভ্যস্থতা, নৃতনত্বের প্রতি প্রতিক্রিয়া এবং কার্যকরী স্মৃতির মাধ্যমে শিশুর নির্বাহী কার্যক্রম মূল্যায়ন করা হবে। কিছু কিছু ছবি এবং ভিডিও দেখানোর সময় শব্দ হতে থাকবে।সর্বোপরি, আমরা আপনার শিশুর বিকাশ সম্বন্ধে নানান ধরনের প্রশ্ন জিজ্ঞাসা করবো যেমন-বিভিন্ন দক্ষতা কোন কিছু স্পর্শ করা/ধরে রাখা / নাড়াচাড়া করানো,কথা বলা ,দেখা, ইত্যাদি) যাতে আমরা তার বিকাশমূলক অক্ষমতা/ ধীরতা পরীক্ষা করে দেখতে পারি। আমরা আপনাকে শিশুর আচরণগত পরিমাপ সম্পর্কিত প্রশ্নমালা থেকে কিছু প্রশ্নও জিজ্ঞাসা করবো এবং যার জন্য আনুমানিক ১:৩০ ঘণ্টা সময় লাগবে। আপনাকে COVID 19   এর প্রভাব সম্পর্কিত কিছু প্রশ্ন জিজ্ঞাসা করা হবে। আমরা যেসব তথ্য সংগ্রহ করবো তা ব্যক্তিগত ও গোপনীয় থাকবে ,এবং পরীক্ষাগুলো থেকে যদি অস্বাভাবিক কিছু পাওয়া যায়  তবে  ,আরও পরীক্ষা নিরীক্ষা করা হবে ,এবং তাকে যথাযথ বিশেষজ্ঞর কাছে পাঠানো হবে। সব পদ্ধতিসমূহ সম্পূর্ণ নিরাপদ এবং এতে আপনার শিশু কোন ব্যাথা পাবেনা। পুরোটা সময় আপনি আপনার শিশুর সাথেই থাকবেন।

EEG পদ্ধতিঃ
ক্লিনিকে অন্য আরেকটি ভিজিট বা সাক্ষাতের সময় ,আমরা সম্প্রসারণশীল উপকরন দিয়ে তৈরি একটি ছোট টুপি দিয়ে আপনার শিশুর মস্তিষ্কের সক্রিয়তা লিপিবদ্ধ করবো। প্রতিটি টুপিতে অনেকগুলো স্পঞ্জ  / ছিদ্র রয়েছে এবং প্রতিটি স্পঞ্জের ভিতরে একটি করে ছোট রেকর্ডিং স্পন্সর / সংবেদক রয়েছে। আপনার শিশুর মাথায় টুপিটি পরানোর পূর্বে  উষ্ণ লবন পানির দ্রবনে ভিজিয়ে রাখব যাতে এর স্পঞ্জগুলো ভিজে নরম হয়ে থাকে। আমরা একটি পরিমাপক ফিতা দিয়ে আপনার শিশুর মাথার চারপাশের দূরত্ব ( মাথার পরিধি ) মাপব যাতে আপনার শিশুর মাথার মাপ অনুযায়ী সঠিক টুপি আমরা ব্যাবহার করতে পারি। যেহেতু আপনার শিশুর মস্তিষ্ক সক্রিয় ,সেহেতু এটি ক্রমাগত ভাবে বৈদ্যুতিক সংকেত পাঠাতে থাকবে,মাথার খুলির ওপর যা ঘুরতে থাকা অবস্থায় টুপিতে লাগানো বিশেষ সেন্সর বা সংবেদক গুলো দিয়ে ধরে রাখবো। পর্দায় আমরা তাকে কিছু মুখাবয়ব এবং আকৃতির ছবি দেখাতে থাকবো এবং আপনার শিশুর  প্রতিক্রিয়া লিপিবদ্ধ

করবো। এরপরে ,চোখের নড়াচড়া পরিমাপ  এর কাজ করবো। আই ট্র্যাকিং যন্ত্রপাতি স্থাপন করার সময়ে আপনার শিশু আপনার কোলে বসে থাকবে এবং উজ্জ্বল কিছু বৃত্তের একটি ভিডিও দেখতে থাকবে। আই ট্র্যাকারটি একটি বিশেষ কম্পিউটারের পর্দা দিয়ে বানানো যাতে পর্দার ধার ঘেঁষে অনেকগুলো লাল আলোর ইনফ্রারেড ক্যামেরা আছে। এই ক্যামেরাগুলো চোখের নাড়াচাড়া অনুসরন করবে এবং পর্দায় দেখানো ছবিগুলো দেখার সময় আপনার শিশু পর্দার ঠিক কোন জায়গাটি দেখছে তা বুঝতে সাহায্য করবে। আপনার প্রথম ভিজিটে আপনার শিশুকে শ্রবনীয়,বেজলাইন, কার্যকরী স্মৃতি, বিচ্ছিন্নতা,ফ্ল্যাঙ্কার এবং ভিইপি কাজ সম্পর্কিত ভিডিও দেখানো হবে। এই সবগুলো কাজ একটি শিশুর এক্সিকিউটিভ ফাংশন নিরূপনের জন্য ব্যবহারের জন্য করা হবে তার আবেগীয় নিয়ন্ত্রণ, স্বীকৃতি, কার্যকরী স্মৃতি, বাঁধা নিয়ন্ত্রণ, মনোযোগ, প্রত্যাশা এবং দৃষ্টির করটিকাল প্রক্রিয়াকরণ এর মাধ্যমে। এবং আমরা আপনার শিশুর মস্তিষ্কের কার্যকারিতা রেকর্ড করে রাখবো। তারপর,কম্পিউটার স্ক্রিনে দেখানো বিভিন্ন ছবির প্রতি আপনার শিশুর চোখের নড়াচড়া লিপিবদ্ধ করবো। একইসাথে, পুরো সেশনটিতে ,কোন কিছুর দিকে শিশুর তাকানো এবং আচরন একটি ডিজিটাল ভিডিও দিয়ে রেকর্ড করে রাখা হবে। এটি গবেষকদের তথ্যসমুহ ভালভাবে বিশ্লেষণ করতে সাহায্য করবে। আপনার শিশুর গোপনীয়তা রক্ষার জন্য, তার নাম ভিডিও রেকর্ডিং এর সাথে দেয়া হবে না এবং এই ফাইল শুধুমাত্র গবেষণার প্রধান গবেষক দেখতে পারবেন।

**বুদ্ধি পরীক্ষার মূল্যায়নের কোন কাজই ক্ষতিকারক নয়**

<u>৩ বছরের MAM বাচ্চাদের খাবার (Outcome reference group )</u>

আপনার শিশুকে বেজলাইনের সমস্ত কাজ সম্পন্ন করার আমরা ৩ বছরের MAM বাচ্চাদের (Outcome reference group ) Nutriset's Plumpy'Sup™, RUSF খাবার দিবো ।

প্রাথমিক মূল্যায়ন শেষ হবার পরে, আমরা আপনার বাচ্চাকে সপ্তাহ ভিত্তিতে ২ মাসের জন্য RUSF (Nutriset's Plumpy'Sup™, RUSF) খাবার বাসায় দিয়ে আসবো যা কিনা প্রতিদিন ১ প্যাকেট করে খাওয়াতে হবে। প্রতিটি বাচ্চাকে ৫৪০ কিলোক্যালোরির ১০০ গ্রাম খাবারের RUSF (Nutriset's Plumpy'Sup™, RUSF)-র ১ টি করে প্যাকেট দেয়া হবে যা ২ মাস পর্যন্ত প্রতিদিন খাবে। খাবার খাওয়ানোর আগে ও পরে একবার করে শারীরিক পরিমাপ ( ওজন , উচ্চতা ও অন্যান্য পরিমাপ) নেয়া হবে এবং প্রয়োজন হলে মহাখালী আইসিডিডিআরবি- র NRU বা স্থানীয় সেবা কেন্দ্রে রেফার করা হবে।

যেহেতু শারীরিক বৃদ্ধি ও জ্ঞানীয় বিকাশের জন্য ইহা একটি গুরুত্বপূর্ণ  সময়কাল, তাই এই বয়সের শিশুদের পুষ্টিগত অবস্থার যত দ্রুত সম্ভব সংশোধন করা জরুরি ।

আপনার (মায়ের) নমুনা সংগ্রহের পদ্ধতিঃ

- মল সংগ্রহঃ তালিকাভুক্তির ২ সপ্তাহের মধ্যে একবার ১০গ্রাম।
- রক্ত সংগ্রহঃ তালিকাভুক্তির ২ সপ্তাহের মধ্যে একবার ৫ মিলি।
- মুখগহবর থেকে মিউকাসের নিঃসৃত নমুনাঃ তালিকাভুক্তির ২ সপ্তাহের মধ্যে একবার।
- শারীরিক পরিমাপ (উচ্চতা এবং ওজন)ঃ তালিকাভুক্তির ২ সপ্তাহের মধ্যে একবার।

ঝুঁকি এবং সুবিধাদিঃ

<u>এই গবেষণায় অংশগ্রহণের ঝুঁকিসমূহ কি কি?</u>

কখনো কখনো গবেষণায় অংশগ্রহণকারীদের সাথে এমন কিছু ঘটে যা তাদের কোন ক্ষতি করতে পারে বা তাদের খারাপ অনুভূতি হতে পারে। এইগুলো কে ঝুঁকি বলা হয়। এই গবেষণায় অংশগ্রহণের ঝুঁকি সমূহের মধ্যে রয়েছে পুষ্টির পরিপূরক, বুদ্ধি পরীক্ষা এবং নমুনা সংগ্রহ।

পুষ্টির পরিপূরকঃ আগের কোন গবেষণায় কোন খারাপ প্রভাব হয়েছিলো বলে জানা নাই, তবে আমরা ইন্টারভেনশন সম্পর্কিত আরও তথ্য সংগ্রহ করবো।

রক্ত সংগ্রহঃ হাল্কা ব্যাথা, অস্বস্তি, রক্তপাত অথবা জখম, অথবা সুঁই বা লেন্সেট রয়েছে এমন কোন ইনজেকশন দেয়া (যা অত্যন্ত বিরল)। এই ঝুঁকি সমূহ কমানোর জন্য, শুধুমাত্র প্রশিক্ষণ প্রাপ্ত , এবং ,অভিজ্ঞ কর্মী রক্ত সংগ্রহ করবেদ্বিতীয়বার ব্যাবহার অনুপযোগী উপকরণ ব্যাবহার করা হবে। যদি রক্ত সংগ্রহ করার সময় কোন সংক্রমণ হয়,প্রয়োজনীয় চিকিৎসা প্রদাণ করা হবে,যার জন্য আপনাকে কোন খরচ করতে হবে না।

মল সংগ্রহঃ মল সংগ্রহ করার সময় কোনরকম ঝুঁকি আছে বলে আমরা প্রত্যাশা করি না।

মুখগহবর থেকে মিউকাসের নিঃসৃত নমুনাঃমুখগহবর থেকে মিউকাসের নিঃসৃত নমুনা সংগ্রহ করার সময় কোনরকম ঝুঁকি আছে বলে আমরা প্রত্যাশা করি না।

বুদ্ধি পরীক্ষার মূল্যায়নঃ এই মূল্যায়ন গুলোতে তেমন কোন বড় ঝুঁকি নেই। প্রতিদিনের করা পরীক্ষার সংখ্যা কিছুটা ক্লান্তির কারন হতে পারে, তবে আপনার এবং আপনার শিশুকে আপনাদের প্রয়োজনমত বিশ্রাম দেয়া হবে।সমস্ত পরীক্ষা এবং পদ্ধতি শিশুর শরীরের বাইরে করা হবে,যা ব্যাথামুক্ত এবং কোন খারাপ প্রভাব মুক্ত। ডিভাইসগুলো শিশুদের জন্য নিরাপদ এবং আরামদায়কভাবে তৈরি করা হয়েছে।

এই পরীক্ষাগুলো থেকে যদি এমন কিছু পাওয়া যায় যা অস্বাভাবিক বা উদ্বেগজনক (EEG কার্যক্রমে অস্বাভাবিক খিঁচুনি), তবে শিশুকে এই বিষয়ে অভিজ্ঞ বাংলাদেশ এর কোন বিশেষজ্ঞের কাছে রেফার করে দেয়া হবে, এবং প্রয়োজনীয় তথ্যসহ পরবর্তীতে আপনাকে কি করতে হবে তা জানানো হবে। এই গবেষণায় আপনাকে বিশেষজ্ঞের কাছে যাবার জন্য গাড়ির ব্যবস্থা করে দেয়া হবে, পরামর্শ এবং রেফারেল প্রক্রিয়ায় সাহায্য করা হবে, তবে আমরা প্রাথমিক চিকিৎসার বাইরে অন্য কোন চিকিৎসার ব্যয়ভার বহন করবো না।

<u>সুবিধাঃ</u>
আপনি এবং আপনার শিশু বিনাখরচে ভালমানের প্রাথমিক চিকিৎসা পাবেন এবং গবেষণায় অংশগ্রহণকালীন সময়ে অন্যান্য অসুখে রেফার করে দেয়া হবে।

<u>আর্থিক সুবিধাঃ</u>
গবেষনায় অংশগ্রহণের জন্য সরাসরি কোন আর্থিক সুবিধা দেয়া হবে না , তবে যদি উদ্বেগজনক কোন তথ্য পাওয়া যায় তখন বিনা খরচে বিশেষজ্ঞের কাছে রেফার করে দেয়া হবে। এছাড়াও নির্ধারিত সাক্ষাতের জন্য যাতায়াত খরচ এবং ক্লিনিকে অবস্থানকালীন সময়ে কিছু খাবার দেয়া হবে।

<u>ব্যাক্তিগত, নামহীনতা এবং গোপনীয়তা</u>
আপনার এবং আপনার শিশুর কাছ থেকে সংগৃহীত সকল তথ্য গোপনীয় রাখা হবে এবং তা একটি সুরক্ষিত জায়গায় গবেষণার গবেষকবৃন্দের দায়িত্বে জমা রাখা হবে। তথ্যসমূহ আইসিডিডিআরবি এবং বোস্টন চিলড্রেন হসপিটালের সুরক্ষিত সার্ভারে জমা রাখা হবে। জৈবিক নমুনা কোনরকম চিহ্নিতকরণ তথ্য ছাড়া আইসিডিডিআরবি তে সুরক্ষিতভাবে রাখা হবে। নিউরো ইমেজিং পরীক্ষাগুলো পৃথক কক্ষে করানো হবে, এবং অংশগ্রহণকারীকে একটি পুনঃ চিহ্নিতকরণ নাম্বার

দিয়ে কোড করা হবে। উপরন্তু , কর্মী এবং গবেষকবৃন্দের গবেষণায় অংশগ্রহণকারীদের স্বার্থ রক্ষা সম্পর্কিত কোর্স সম্পন্ন করা আছে।
সাধারনভাবে, যারা এই গবেষণার সাথে যুক্ত আছেন, এমনকি যারা আর্থিক সহায়তা দিয়েছেন এবং গবেষণার নীতিনির্ধারক তারাও তথ্যসমূহ দেখতে পাবেন, যার মধ্যে রয়েছে আপনার এবং আপনার শিশুর সম্পর্কিত তথ্য, স্থিরচিত্র এবং ভিডিও। উদাহরনস্বরূপ, নিম্নলিখিত ব্যাক্তিবর্গ আপনার এবং আপনার শিশুর সম্পর্কিত তথ্য জানতে পারেনঃ

- আইসিডিডিআর,বির গবেষনাকর্মী
- বোস্টন চিলড্রেন হসপিটালের গবেষনাকর্মী
- অকল্যান্ড ইউনিভার্সিটির গবেষনাকর্মী
- ট্রপিকাল মেডিসিন রিসার্চ ইন্সটিটিউট, জ্যামাইকার গবেষনাকর্মী

এই পদ্ধতিগুলোর ফলাফল উপস্থাপন বা প্রকাশের সময় আপনার বা আপনার শিশুর নাম এবং পরিচয় কোথাও প্রকাশ করা হবে না।

আপনি যদি এই ফর্মটিতে সাক্ষর করেন, তবে আপনি তথ্য প্রকাশের অনুমতি দিতেছেন, অনুমোদিত গবেষকবৃন্দ এবং নিরাপত্তা কমিটি, আইসিডিডিআর,বি ইথিকাল রিভিউ কমিটি, নীতিনির্ধারক কর্ত্রীপক্ষ (বাংলাদেশ এবং আমেরিকা উভয়পক্ষ), গবেষণার অর্থ যোগানদাতা এবং যারা দায়িত্বে আছেন, এবং অন্যান্য গবেষণা প্রতিষ্ঠান। এই অনুমতির কোন মেয়াদউত্তীর্ণ তারিখ নেই। যদি আপনি নিজের অনুমতি প্রত্যাহার করেন এবং এই চুক্তিটি শেষ করার সিদ্ধান্ত নেন, তবে ডঃ রাশিদুল হকের সাথে নিম্নউল্লেখিত ঠিকানা/নাম্বারে যোগাযোগ করুন। তিনি বা তার কর্মীরা এই অনুমতি প্রত্যাহারের সিদ্ধান্ত লিখিতভাবে রাখতে সহায়তা করবেন। দয়া করে মনে রাখবেন যে, গবেষণায় ইতোমধ্যে যেসব তথ্য নেয়া হয়েছে তা ব্যাবহার করা হতে পারে।

আপনার গবেষণায় অংশগ্রহণ করা ঐচ্ছিক। তবে, এই ফর্মটিতে সাক্ষর না করলে আপনি এই গবেষণায় অংশগ্রহণ করতে পারবেন না।

<u>নমুনার ভবিষ্যৎ ব্যাবহারঃ</u>
এই গবেষনা শেষে, সব নমুনা আইসিডিডিআর,বিতে ৫ বছরের জন্য সংরক্ষন করা হবে। আপনি রাজী থাকলে, এই নমুনা আমরা ভবিষ্যতে অন্য গবেষণার কাজে ব্যাবহারের জন্য সংরক্ষন করবো। যদি আমরা বা আমাদের অন্য কোন সহযোগী প্রতিষ্ঠান গবেষণাটি করেন, সেক্ষেত্রে তখন যথাযথ কত্রিপক্ষের কাছ থেকে অনুমতি নেয়া হবে। ভবিষ্যতে এই নমুনা ব্যাবহার করা হলে, আপনার শিশুর ব্যাক্তিগত গোপনীয়তা রক্ষা করা হবে। যদি আপনি আপনার শিশুর নমুনা ভবিষ্যতে গবেষণা কাজে ব্যাবহারের জন্য সংরক্ষনের অনুমতি প্রদান করেন কিন্তু পরবর্তীতে আপনার সিদ্ধান্ত পরিবর্তন করেন, আপনি আমাদের সাথে যোগাযোগ করলে আমরা নমুনা নস্ট করে ফেলবো। যদি আপনি সিদ্ধান্ত পরিবর্তন না করেন, তাহলে আমরা আপনার শিশুর নমুনা ৫ বছর পর্যন্ত সংরক্ষন করবো। দ্রষ্টব্য, নমুনা বিশেষ পরীক্ষার জন্য বাংলাদেশের বাইরে সহযোগীদের কাছে পাঠানো হতে পারে।

<u>তথ্যের ভবিষ্যৎ ব্যাবহারঃ</u>
আপনার এবং আপনার শিশুর সম্পর্কিত তথ্য শেয়ার করা হবে নীতিনির্ধারক কত্রিপক্ষ সহ আইসিডিডিআর,বি ইথিকাল রিভিউ কমিটি (ERC), ইন্সটিটিউশনাল রিভিউ বোর্ড (IRB) অকল্যান্ড ইউনিভার্সিটি, বোস্টন চিলড্রেন হসপিটাল, ট্রপিকাল মেডিসিন রিসার্চ ইন্সটিটিউট, জ্যামাইকা, এবং গবেষণার অর্থ যোগানদাতা এবং যারা দায়িত্বে আছেন। গবেষকগণ ভবিষ্যতে গবেষণার উদ্দেশ্যে

তাদের বিবেচনার ভিত্তিতে অন্যান্য গবেষকদের সাথে তথ্য এবং তথ্য ভাগ করে নিতে চাইতে পারেন। আমাদের গবেষণার তথ্যসমুহ BCH ডাটা কোরের সহযোগীগণের সাথে ভাগ করা হবে, এবং BCH কে আমরা তথ্য পাঠাবো, শনাক্তকরণ সহ, যেটা নিয়ে তারা BCH  REDCap ডেটাবেজে কাজ করবে। Wellcome Leap Foundation থেকে প্রাপ্ত অর্থ চুক্তির মধ্যে  একটি চুক্তি ছিল এই গবেষণায় অংশগ্রহণকারী প্রতিটি শিশুর উপরে বিভিন্ন পদ্ধতি পরিচালনের মাধ্যমে প্রাপ্ত তথ্য, Leap consortiam এর সহযোগীগণের সাথে ভাগ করে নেয়া হবে এবং Leap এর মাধ্যমে স্পন্সরদের চাহিদানুযায়ি Synapes এ সহজলভ্য হবে (Maintain by Sage Binetworks; synapes .org; WIRB#20112068). এই তথ্যসমূহ BCH এ কাজ শেষ হয়ে যাবার ৭ বছর পর্যন্ত সংরক্ষিত রাখা হবে, সম্ভবত প্রয়োজনে আরও বেশি সময়ও রাখা হতে পারে। এটা স্পন্সরদের চাহিদানুযায়ি  তথ্য সংরক্ষনের একটি কর্মধারা। আমাদের কাছ থেকে প্রাপ্ত তথ্য প্রক্রিয়াকরণ এবং বিশ্লেষণে BCH সহযোগীতা করবে।

<u>অংশগ্রহণ না করা এবং প্রত্যাহার করার অধিকারঃ</u>

গবেষণায় অংশগ্রহণ বিষয়টি ঐচ্ছিক এবং আপনি চাইলে অংশগ্রহণ নাও করতে পারেন অথবা যেকোন সময় নাম প্রত্যাহার করতে পারেন, যার জন্য আপনাকে কোন ক্ষতিপূরণ দিতে হবে না বা চিকিৎসা গ্রহণেও কোন সমস্যা হবে না।

# মায়ের নিজের ও তার ৩ বছর বয়সী শিশুর সম্মতিপত্র

SID:

| Protocol No. PR-21084 | Version No. 3 .00 | Date: 12th June'2022 |
|---|---|---|

**Protocol Title: Multidimensional evaluation of the early emergence of executive function and emotional regulation in young children in Bangladesh using nutritional and psychosocial intervention: A Pilot study**

**Investigator's name: Dr. Rashidul Haque**

**Organization: International Centre for Diarrhoeal Disease Research, Bangladesh (icddr,b)**

আপনি যদি আপনার শিশুকে আমাদের গবেষণায় নাম লেখানোর জন্য আমাদের প্রস্তাবে সম্মত হন তবে দয়া করে নীচের বাক্সগুলোতে √ (চিহ্ন) দিন এবং শেষে নির্দিষ্ট স্থানে স্বাক্ষর / বাম বৃদ্ধা আঙুলের ছাপ দিন:

| পয়েন্ট | স্টাটাস |
|---|---|
| আমি পড়েছি / গবেষণা কর্মীরা এই গবেষণার সমস্ত তথ্য ভার্সন 3, 12th June,২০২২ থেকে পড়ে শোনায়, তারা আরো প্রশ্ন জিজ্ঞাসা করার ও এই গবেষণা সম্পর্কে আলোচনা করার সুযোগ দেয় এবং তাদের কাছ থেকে সন্তোষজনক উত্তর পাই। | হ্যাঁ ☐ না ☐ |
| আমি কোন কারণ ছাড়াই এই গবেষণা থেকে বের হয়ে আসতে পারি। | হ্যাঁ ☐ না ☐ |
| আমি বুঝলাম যে আমার দেওয়া সকল তথ্য গোপন থাকবে। | হ্যাঁ ☐ না ☐ |
| আমি গবেষণা কর্মীকে প্রতিটি নির্ধারিত ফলোআপ ভিজিট এবং হোম ভিজিটে আমার এবং আমার সন্তানের কাছ থেকে তথ্য সংগ্রহের অনুমতি দেওয়ার বিষয়ে সম্মত হই। | হ্যাঁ ☐ না ☐ |
| আমি সম্মতি দিচ্ছি যে, এই গবেষণা সমীক্ষা থেকে প্রাপ্ত তথ্য আইসিডিডিআর, বি, বোস্টন চিলড্রেন হাসপাতাল, মার্কিন যুক্তরাষ্ট্র, অকল্যান্ড বিশ্ববিদ্যালয়, নিউজিল্যান্ড এবং ট্রপিকাল মেডিসিন রিসার্চ ইনস্টিটিউট, জামাইকা, সিনপস ( যারা গবেষনার তথ্য সংরক্ষন ও বিশ্লেষনে গবেষকদের সহায়তা দিয়ে থাকে) দ্বারা সংরক্ষণ করা হবে এবং ভবিষ্যতে অশনাক্তকারী অংশগ্রহণকারী হিসাবে পাবলিক ডেটাবেজে অন্তর্ভুক্ত থাকতে পারে । অশনাক্তকরণের অর্থ হলো আমি এবং আমার শিশুর নাম দ্বারা বা ডাটাবেসে থাকা অন্যান্য সনাক্তকারী চিহ্ন দ্বারা সনাক্ত করা যাবে না। আমার পুরো নাম বা কোনও ঠিকানার বিস্তারিত কোথাও অন্তর্ভুক্ত করা হবে না। প্রকাশিত তথ্যগুলি এই গবেষণায় আমার বা আমার সন্তানের অংশগ্রহণ সনাক্ত করবে না। আমি নির্দিষ্ট ব্যক্তিদের আমার তথ্য পাওয়ার অনুমতি দিচ্ছি। | হ্যাঁ ☐ না ☐ |
| আমি আমার সন্তানের কাছ থেকে ২-৩ মিলি রক্ত, মল, মুখগহবর থেকে মিউকাসের নমুনা দিতে সম্মত হয়েছি। | হ্যাঁ ☐ না ☐ |
| আমি আমার কাছ থেকে ৫ মিলি রক্ত, মল, মুখগহবর থেকে মিউকাসের নমুনা দিতে সম্মত হয়েছি। | হ্যাঁ ☐ না ☐ |
| আমার আর আমার সন্তানের কাছ থেকে যে বেনামে রক্ত, মল, মুখগহবর থেকে মিউকাসের নমুনা সংগ্রহ করা হয়েছিলো সেই নমুনাগুলি বিশ্লেষণের জন্য বিদেশে পাঠানোর বাপারে সম্মত হয়েছি। | হ্যাঁ ☐ না ☐ |
| আমার সন্তানকে পুষ্টির পরিপূরক খাবার এবং মানসিক উদ্দীপনা দিতে সম্মত আছি (যদি আমার শিশু অপুষ্টিতে ভুগে) | হ্যাঁ ☐ না ☐ |
| আমি আমার সন্তানের বুদ্ধি বিকাশের পরীক্ষা (এক্সিকিউটিভ ফাংশন, ইমোশনাল রেগুলেশন, এফএনআইআরএস, ইইজি) করতে সম্মত আছি | হ্যাঁ ☐ না ☐ |
| আমি এই গবেষণায় LENA রেকর্ডিং, বেইলী-৪ মূল্যায়ন, পিতামাতা ও সন্তানের পারস্পরিক মিথষ্ক্রিয়া রেকর্ডিং এবং আমার শিশুর বিকাশমূলক মূল্যায়নের জন্য প্রশ্নমালা পূরনে রাজী আছি। | হ্যাঁ ☐ না ☐ |
| নৈতিকভাবে অনুমোদিত গবেষণা তে আমি এবং আমার সন্তানের তথ্য এবং নমুনাগুলি সংরক্ষণ এবং ভবিষ্যতে ব্যবহারে সম্মত আছি। | হ্যাঁ ☐ না ☐ |

| | |
|---|---|
| আমি আমার সন্তানের ছবি গবেষণায় ভবিষ্যেতে ব্যবহারের জন্য সম্মত আছি। | হ্যাঁ ☐ না ☐ |
| আমি ভবিষ্যতে গবেষণা সম্পর্কিত তথ্যের জন্য যোগাযোগ করাতে সম্মত আছি। | হ্যাঁ ☐ না ☐ |
| আমি অনুধাবন করতে পারলাম যে, গবেষণাতে আমার এবং আমার সন্তানের চিকিৎসার তথ্য এবং সংগ্রহ করা তথ্য পৃষ্ঠপোষক ব্যক্তি এবং নিয়ন্ত্রক কর্তৃপক্ষ দ্বারা অনুসন্ধান করা হতে পারে, যেখানে এটি আমার এবং আমার সন্তানের এই গবেষণায় অংশ নেওয়ার ক্ষেত্রে প্রাসঙ্গিক। আমি সেই নির্দিস্ট ব্যক্তিদের আমার তথ্য পাওয়ার অনুমতি দিয়েছি। | হ্যাঁ ☐ না ☐ |
| আমি এই গবেষণায় অংশ নিতে সম্মত আছি। | হ্যাঁ ☐ না ☐ |

_______________________________________ _____________________

অংশগ্রহণকারীদের স্বাক্ষর বা বাম বৃদ্ধা আঙুলের ছাপ তারিখ (দিন/মাস/বছর)

__________________________________________ ___________________

মাতা-পিতা / অভিভাবক / উপস্থিতির স্বাক্ষর বা
বাম বৃদ্ধা আঙুলের ছাপ তারিখ (দিন/মাস/বছর)

____________________________________ __________________

সাক্ষীর স্বাক্ষর তারিখ (দিন/মাস/বছর)

_____________________________________ __________________

গবেষক বা তার প্রতিনিধি স্বাক্ষর তারিখ (দিন/মাস/বছর)

**যোগাযোগের জন্য**:
আপনার যদি কোনও প্রশ্ন থাকে তবে আপনি এখন বা যে কোনও সময় নীচে উল্লিখিত কর্মীদের কাছে জিজ্ঞাসা করতে পারেন:

| যোগাযোগের উদ্দেশ্য | নাম এবং ঠিকানা | যোগাযোগের ঠিকানা |
|---|---|---|
| স্টাডি সম্পর্কিত যে কোনও প্রশ্নের জন্য, বা কোনও সমস্যার জন্য | ডা: মাসুদ আলম | ঠিকানা: বাড়ি-২৮, এভিনিউ-১, কলসী রোড, মিরপুর-১২, ঢাকা-১২১৬. মোবাইল নংঃ ০১৭১১৫৭০৫৫০ (৭/২৪ ঘন্টা খোলা থাকবে) |
| | ডা: রাশিদুল হক। | ঠিকানা: প্যারাসাইটোলজি ল্যাবরেটরি, আইসিডিডিআর,বি, মহাখালী, ঢাকা-১২১২ .মোবাইল: ০১৭১৩০৯৩৮৫৯ (সকাল ৯:০০ টা থেকে বিকাল ৫:০০ টা পর্যন্ত) |
| অধিকার বা সুযোগ সুবিধা জানতে বা কোনও অভিযোগ বা অসন্তুষ্টি জানাতে | এম এ সালাম খান (আইআরবি সমন্বয়কারী) | আইআরবি সচিবালয়, গবেষণা প্রশাসন, আইসিডিডিআর,বি, মহাখালী, ঢাকা-১২১২ ফোনঃ (+৮৮-০২) ৯৮২৭০৮৪ বা মোবাইল: ০১৭১১৪২৮৯৮৯ |

আপনার সহযোগিতার জন্য ধন্যবাদ।
স্বাক্ষরকৃত সম্মতি পত্রের একটি অনুলিপি আপনাকে দেওয়া হবে।
