# Supplementary material for: Multidimensional evaluation of the early emergence of executive function and development in Bangladeshi children using nutritional and psychosocial intervention: A randomized controlled trial protocol
Source: PLoS One. 2024 Mar 15;19(3):e0296529. doi: 10.1371/journal.pone.0296529 (PMC10942035; doi:10.1371/journal.pone.0296529)
Supplement: S2 File — (PDF) [file pone.0296529.s003.pdf]

Multidimensional Evaluation of the emergence of executive function and dysfunction in young children in Bangladesh using Nutritional and Psychological Intervention: Pilot Study<br>**Standard Operating Procedure (SOP)**<br>**Anthropometric Measurement**	PR-21084<br><br>Version No 01

Date: 15 September 2021<br>Written By: Dr Shahria Hafiz Kakon		Effective Date:21 September 2021
Related Documents and Attachments: None		

Purpose

- Anthropometry needs to be conducted accurately and precisely using standardized methods to identify of wasting MAM children and healthy control and also to assess the nutritional status of children in LEAP study.

- To measure the height, weight, MUAC of enrolled participants in a uniform manner at protocol-designated time point to ensure accuracy of data collection, and to maintain protocol compliance. Mid-upper arm circumference (MUAC) is an anthropometric measure providing information on muscle mass and subcutaneous fat. It also is a key indicator of the nutritional status of children.

- Head circumference (HC) will be taken to correlate with the developmental measures of the children of both cases (children with MAM) and healthy controls at protocol-designated time point to ensure accuracy of data collected, and to maintain protocol compliance.

Time point of Anthropometric Measurement in LEAP study

- Children with MAM (intervention group) wt, ht, MUAC will be taken during screening time, at enrolment, then weekly till anthropometric recovery (WHZ>-1 z-score) then quarterly (3 monthly) until 3 years of age of children.

- Children of the healthy control group wt, ht, MUAC will be measured screening time, at enrolment, then monthly for 3 months, then quarterly (3 monthly) until 3 years of age of children.

- Maternal measurements (weight, height, MUAC) will be collected once at enrollment.

- Head circumference will be taken from children at enrollment (1 year) , at 2years and 3 years of age of children

Materials for anthropometry Measurements

- Pen
- Seca digital scales (for infant)
- Seca mother-baby scales (for older children)
- Recumbent lengt board (stadiometer) for measuring standing height
- Infant meter (Seca) for measuring supine length
- Disinfectant/alcohol surface wipes for cleaning measuring board and scale
- Mother digital scale
- Measuring tape for mother
- Follow up clinic visit case report form (CRF) for correct visit for subject (Infant or mother)
- Clinic visit source document
- WHO Weight for height z-score (WHZ) chart
- WHO MUAC chart
- UNICEF/WHO MUAC measuring tape
- White eyeliner pencil (to mark the measuring position on the infant's arm)
- Alcohol swabs to clean the tape
- Measuring tape for Head circumference
- Clinical paper roll

Methods of Child anthropometric Measurement

- All Field Research Assistants (FRAs) will be trained to perform anthropometric measurements and must demonstrate proficiency before measuring enrolled subjects.
- FRAs will take anthropometric measurements of enrolled children at the screening and Enrolment visit as well as specific time points according to protocol.
- FRAs will additionally take anthropometric measurements of enrolled mother within the first two weeks of enrolment.
- Prior to conducting the measurements, FRAs will explain to the mother what they intend to do to ensure the mother understands what is happening to her child.
- All scales will be calibrated daily. At each assessment, 3 consecutive measurements will be taken and the average value will be recorded on the visit CRF and the WHO growth chart.
- FRA may require the assistance of a second member of the study staff in obtaining accurate measurements
- All equipment should be wiped clean by disinfectant before and after measuring each subject. The equipment should be cool, clean and safely secured. Child measuring devices should be placed on the floor or another solid, stable surface while measurements are taken.
- All aseptic safety precaution and PPE will be maintained during anthropometry collection for covid 19 prevention.

Procedure for taking weight for infant

- FRAs should weight the child using a digital scale.
- Ask the mother to remove all clothing from the child (including their nappy). To avoid the child moving too much ask, the mother to help distract the child while take the weight measurement
- Prior to weighing the infant, the scale must be turned on and check there is nothing on the scales. Press the green Start key to switch on the scales. The display will read SECA and then quickly run through all elements of the display. The scales are ready for operation when the display reads 0.000.
- Place a layer of clinical paper on the tray of the scales. It is important that clinical roll does not hang over the edge of the tray. The child should be centred on the scale tray and the baby should be as still as possible. Infant should be weighed laid down in the scale but if older babies are too long or will not stay in this position and they can sit unsupported, then they can be sat on the scales.
- Read off the measured weight to 2 decimal places. Note that the scale reads to 3 decimal places, but the final number displayed is always zero. When transferring data to the CRF, ignore the final digit (e.g., 11.460 kg should be recorded as 11.46 kg on the CRF. Note that if the weight is below 10kg, you should record a zero at the front, e.g., 8.36kg is recorded on the CRF as 08.36 kg). Missing measurements should be entered as 99.99.
- The FRA will take the average of the two measured weights and will record on the associated paperwork.
- If the infant moves excessively while the scales are stabilising you may get a false reading. If you think this is the case, reweigh the infant.
- Ask the mother to lift the infant off the scales.
- Throw away the clinical paper and wipe down the scales with an antibacterial surface wipe after each use.

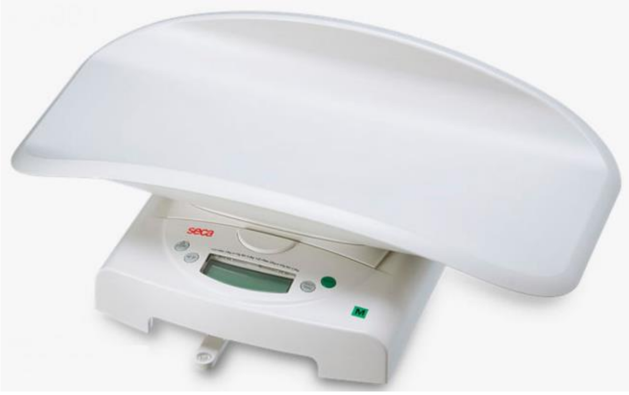

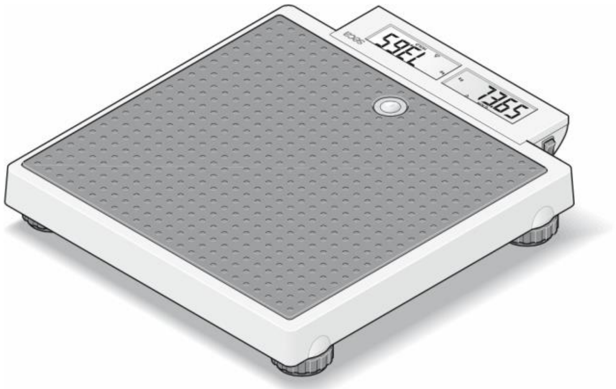

Procedure for taking weight in children above 2 years

- Press the start key with no load on the scale. When SECA will appears 0.00 then it will be ready to use.
- Ask the mother to stand the child on the scale. If necessary, study staff can pick up the child on the scale.
- The child's weight will display, after a few seconds of stabilizing the reading will be fixed.
- Transfer the reading to the CRF and repeat 2 more times.
- The scale switches itself off automatically.

Procedure for taking the infant's height.

- The length of the child will be measured two times using a measuring board.
- The measuring board should be covered with clinical paper.
- The infant's shoes and socks should be removed (if applicable).
- The FRA should place the subject in a supine position on the measuring board with the crown of the head touching the headboard. A second staff member or the child's mother will hold the child's head to maintain this position during measurement.
- The legs must be extended at the hips and knees by the measurer and held flat on the board with one hand while the measurer slides the movable board against the heels with her other hand. a gentle downward pressure over the legs during measurement. It is very important that this pressure should be GENTLE. It is very important that the measurement be taken with the child's feet flat against the slide, including the heels.
- Length will be recorded to the CRF in cm to the nearest 0.1 cm. Missing measurements

should be entered as 999.9.

- Wipe the height board with an antibacterial surface wipe after taking the measurements.

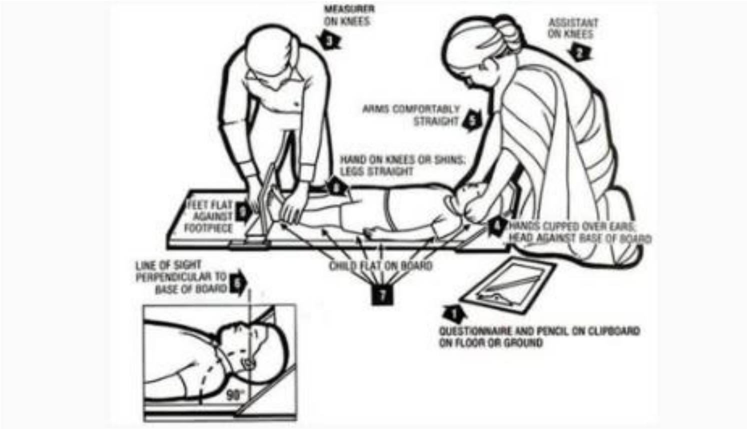

Measuring length. (Source: UNICEF, 1986, How to weigh and measure children: assessing the nutrition status of young children)

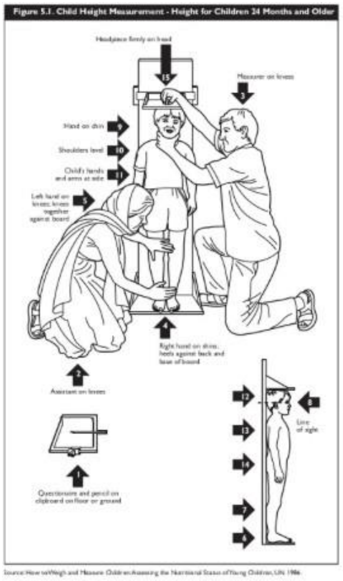

Procedure for taking the child's height (above 2years)

- The child should stand with his/her back to the device. The child should be standing with the device cantered down the middle of the body and should be standing with heels together and heels, buttocks, and shoulders touching the wall.
- The child should tuck his/her chin down into the chest and stand as tall as possible. If the measuring device has a horizontal bar to assist with the measurement, the bar should be raised above the child's head and lowered until it just touches the head (the skull; not just the hair).
- It is important to make certain that the bar is completely horizontal. If the bar is at an angle greater or less than 90° to the wall, the measurement of height will be inaccurate.
- Height will be recorded to the CRF in cm to the nearest 0.1 cm

Procedure for taking infant/ children MUAC

- Explain the procedure to the mother.
- The baby must have a bare arm and shoulder for this measurement. Position the baby in a sitting position on the mother's lap so that it can easily access the left arm. The baby will need to have their elbow bent at a right angle. The mother should hold the left hand to prevent the baby from pulling their arm away.

- Identify the process of the Acromion (this is the end of the shoulder bone,

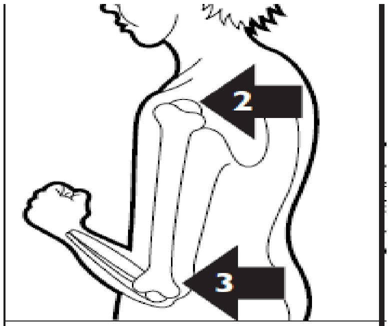

2. Tip of shoulder
3. Tip of elbow

Arrow 2) and the tip of the elbow (olecranon process, **Arrow 3**).

- Using the measuring tape, measure the distance between these points. Divide this measurement in half, this is the mid-point of the upper arm; mark this mid-point with the white eyeliner pen.
- Pass the tape over the baby's hand and slip it up the baby's arm, to the mid-point that has marked. The mother should hold the baby's arm loosely, and the arm should be straight.
- The tape should lie on top of the mark, covering it. Ensure the tape is passing horizontally around the arm, not sloping, and is in contact with the skin but does not compress tissue underneath (**Arrow 7**). It should not be puckering the skin (**Arrow 8**) or too loose (**Arrow 9**).

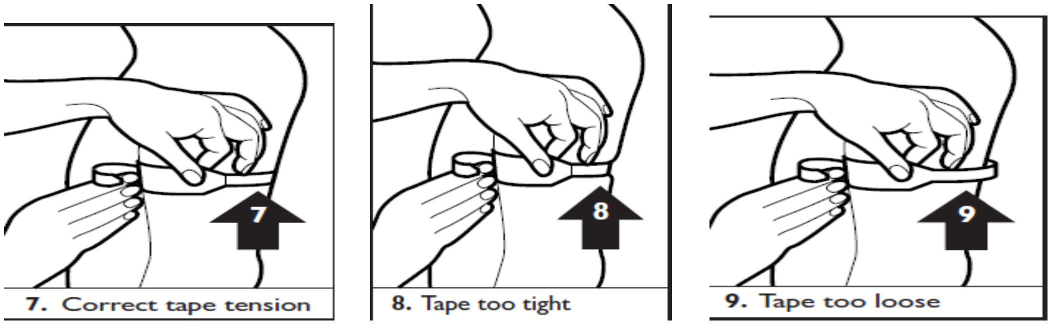

7. Correct tape tension   8. Tape too tight   9. Tape too loose

- Read the measurement to the nearest 0.1cm. If the arrow falls between millimetres always read to the nearest whole millimetre. Write this down or enter directly into CRF.
- Loosen and then reposition the tape, and repeat the measurement two more times.
- Ensure all the measurements are entered into the CRF.
- Offer a non-alcohol wipe to the mother so that they can wipe off the white eyeliner marks on the baby’s arm, if they wish to.
- Clean the MUAC tape after use with the antibacterial surface wipe.

Procedure of taking children head circumference

- Explain the procedure to the mother.
- Position the child on the mother's lap.
- Ask the mother to remove any hair clips, bobbles, hair bands or hats from the child's hair/head.
- Place the measuring tape around the child's head at its largest diameter midway between the eyebrows and the hairline at the front of the head, above the ears and around the occipital prominence at the back of the head. Your aim is to always measure the largest circumference possible.
- Ask the mother to distract the infant with toys as they may try to pull the tape off.
- Pull the tape snugly to compress the hair. Make sure that the tape passes the occipito-frontal plane (as described above) and does not slip when getting the reading. It may be helpful to ask the mother to hold the tape in place on the back of the head with a finger.
- Read the measurement to the nearest 0.1 cm.
- Write down the measurement or enter it directly into the CRF.
- Repeat the measurement two more times.
- Wipe the measuring tape with the antibacterial surface wipes after each child and store to avoid damage and creasing.

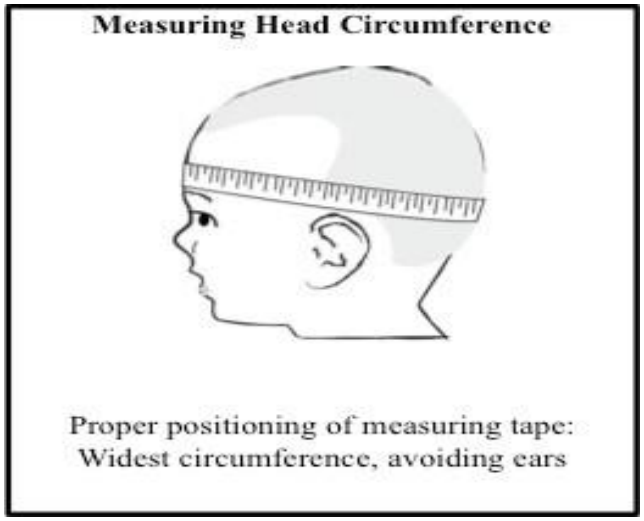

Measuring Head Circumference
Proper positioning of measuring tape:
Widest circumference, avoiding ears

Procedure of taking mother's weight

- FRAs will weigh the mother using a digital scale.
- The mother should be weighed in light clothing, the weight of which can be estimated.
- The scale should be placed on a flat surface, and the mother asked to stand on the scale without moving, so scale can accurately display her weight. The mother's weight should be taken alone, with nothing or no one else on the scale.
- The mother is weighed only one time to the nearest 10 g or 0.01 kg. Her weight should be recorded on the CRF of the study.

Procedure of taking mother's height

- FRAs will measure the height of the mother using a vertical height measuring board.
- The subject should stand in an upright position with her back against the board. The FRA should instruct the mother to place her heels together and move them all the way so they are touching the back of the board. The shoulders and buttocks should also be touching the board.
- The FRA should instruct the mother to stand up straight, keep her head up and look forward.
- The FRA will then slide the movable board down to the top or the crown of the head and read the measuring tape at the point where the board meets the crown.
- Height will be measured to the nearest 0.1 cm and recorded on the CRF of Mother of the study.

References

MAL_ED Manual of procedures.

WHO, Physical status: the use and interpretation of anthropometry report of a WHO expert committee, World Health Organization.

Brief Infant Sleep Questionnaire – Revised Short Form

Please answer a few questions about your family.

1. What is your relationship to your child?

☐ Mother ☐ Grandparent

☐ Father ☐ Other

2. What is the highest degree that you completed?

☐ Less than high school/secondary

☐ High school/secondary

☐ College/university

☐ Graduate (e.g., MS, MD, JD, Ph.D.)

☐ Prefer not to answer

3. How old is your child (in months)?

________months

4. Was your child premature (born before 37 weeks' gestation)?

☐ Yes

☐ No

☐ I don't know

5. My child's biological sex is

☐ Male

☐ Female

☐ Intersex

6. In which country/region do you currently reside?

Country/region: ___________________________

7. How many nights per week are you involved with your child at bedtime and/or overnight?

☐ 0 ☐ 1 ☐ 2 ☐ 3 ☐ 4 ☐ 5 ☐ 6 ☐ 7 nights

Please think about your child's sleep during the past <u>two weeks</u> in answering the following questions.

1. What time do you usually start your child's bedtime routine (start getting your child ready for bed)?

Example: 7:45 PM would be written as _____7_:_45_PM

I start getting my child ready for bed at _______:_______

2. In a typical week, how often does your child have the exact same bedtime routine?

☐ 0 ☐ 1 ☐ 2 ☐ 3 ☐ 4 ☐ 5 ☐ 6 ☐ 7 nights

3. Where does your child usually fall asleep at bedtime?

☐ Crib
☐ Own bed (any size)
☐ Parents' bed
☐ Co-sleeper (attached to parents' bed)
☐ Bassinet/infant seat
☐ Swing/stroller
☐ Parent's/adult's arms
☐ Other

4. How does your child usually fall asleep at bedtime?

☐ While being held or rocked
☐ With an adult in the room, but not being held or rocked
☐ On own (without an adult in the room)

5. At bedtime, does your child usually fall asleep while breastfeeding, bottle feeding, or drinking from a sippy cup?

☐ Yes
☐ No

6. What time do you usually put your child to bed at night (lights out)?

Example: 8:30 PM would be written as ____8__:_30_PM

I put my child to bed at _______:_______

7. Typically, how difficult is bedtime?

☐ Very easy
☐ Somewhat easy
☐ Neither easy nor difficult
☐ Somewhat difficult
☐ Very difficult

8. How long does it usually take your child to fall asleep?

Example: If you put your child to bed at 6:30 pm and your child falls asleep at 8:00 pm, it takes 1 hour and 30 minutes for your child to fall asleep.

_________ hours
_________ minutes

9. In what room does your child sleep for most of the night?

☐ In his/her own room
☐ In parents' room
☐ In sibling's or other bedroom
☐ In another room of the house

10. Where does your child sleep for most of the night?

☐ Crib
☐ Own bed (any size)
☐ Parents' bed
☐ Co-sleeper (attached to parents' bed)
☐ Bassinet/infant seat
☐ Swing/stroller
☐ Other

11. How many times does your child usually wake during the night?

_______ times per night

12. When your child wakes up during the night, what do you usually do? (check all that apply)

☐ Pick up my child and put him/her back down while still awake
☐ Bottle feed or give a sippy cup to put my child back to sleep
☐ Breastfeed/nurse my child back to sleep
☐ Play with my child, watch TV, or use/show smartphone/tablet
☐ None of these

13. How much total time during the NIGHT is your child usually awake (between when your child goes to bed and wakes for the day)?

Example: If your child wakes up 2 times and is awake for about 15 minutes each time, your child's total time spent awake is 30 minutes. In that case, write "0 hours, 30 minutes."

________ hours
________ minutes

14. What is the <u>longest stretch</u> of time that your child is asleep during the NIGHT without waking up?

________ hours
________ minutes

15. What time does your child wake up in the morning?

My child wakes up at _______:_______

16. How much total time does your child spend sleeping during the NIGHT (between when your child goes to bed and wakes for the day)?

Example: If your child sleeps for 3 hours, wakes up, then sleeps for 5 hours and 30 minutes more, your child sleeps for 8 hours and 30 minutes total.

________ hours
________ minutes

17. How well does your child usually sleep at night?

☐ Very well
☐ Well
☐ Fairly well
☐ Poorly
☐ Very poorly

18. On a typical DAY, how many naps does your child take (between when your child wakes for the day and goes to bed at night)?

________ naps

19. How much total time does your child spend sleeping during the DAY (between when your child wakes for the day and goes to bed at night)?

Example: If your child took 2 naps and slept 1 hour each time, your child's total time spent sleeping during the day is 2 hours.

________ hours
________ minutes

20. Do you consider your child's sleep a problem?

☐ Not a problem at all
☐ A very small problem
☐ A small problem
☐ A moderate problem
☐ A serious problem

If you have any concerns about the responses that you provided about your child please speak with your child's pediatrician.

[redacted] ____________________ Gender ________ Age ______ [redacted] ___/___/___

[redacted] ____________________ Today's Date ___/___/___

Relationship to Child: ☐ Mother ☐ Father ☐ Teacher* ☐ Other* ____________________

How well do you know the child? ☐ Not Well ☐ Moderately Well ☐ Very Well *Have known the child for______ ☐ months ☐ years

During the past 6 months, how often has each of the following behaviors been a *problem*?

	Behavior	Never	Sometimes	Often
1.	Overreacts to small problems	N	S	O
2.	When given two things to do, remembers only the first or last	N	S	O
3.	Is unaware of how his/her behavior affects or bothers others	N	S	O
4.	When instructed to clean up, puts things away in a disorganized, random way	N	S	O
5.	Becomes upset with new situations	N	S	O
6.	Has explosive, angry outbursts	N	S	O
7.	Has trouble carrying out the actions needed to complete tasks (such as trying one puzzle piece at a time, cleaning up to earn a reward)	N	S	O
8.	Does not stop laughing at funny things or events when others stop	N	S	O
9.	Needs to be told to begin a task even when willing to do it	N	S	O
10.	Has trouble adjusting to new people (such as babysitter, teacher, friend, or day care worker)	N	S	O

11.	Becomes upset too easily	N	S	O
12.	Has trouble concentrating on games, puzzles, or play activities	N	S	O
13.	Has to be more closely supervised than similar playmates	N	S	O
14.	When sent to get something, forgets what he/she is supposed to get	N	S	O
15.	Is upset by a change in plans or routine (for example, order of daily activities, adding last minute errands to schedule, change in driving route to store)	N	S	O
16.	Has outbursts for little reason	N	S	O
17.	Repeats the same mistakes over and over even after help is given	N	S	O
18.	Acts wilder or sillier than others in groups (such as birthday parties, play group)	N	S	O
19.	Cannot find clothes, shoes, toys, or books even when he/she has been given specific instructions	N	S	O
20.	Takes a long time to feel comfortable in new places or situations (such as visiting distant relatives or new friends)	N	S	O

21.	Mood changes frequently	N	S	O
22.	Makes silly mistakes on things he/she can do	N	S	O
23.	Is fidgety, restless, or squirmy	N	S	O
24.	Has trouble following established routines for sleeping, eating, or play activities	N	S	O
25.	Is bothered by loud noises, bright lights, or certain smells	N	S	O
26.	Small events trigger big reactions	N	S	O
27.	Has trouble with activities or tasks that have more than one step	N	S	O
28.	Is impulsive	N	S	O
29.	Has trouble thinking of a different way to solve a problem or complete an activity when stuck	N	S	O
30.	Is disturbed by changes in the environment (such as new furniture, things in room moved around, or new clothes)	N	S	O

If you have any concerns about the responses that you provided about your child please speak with your child's pediatrician.

During the past 6 months, how often has each of the following behaviors been a *problem*?

	Item	Never	Sometimes	Often
31.	Angry or tearful outbursts are intense but end suddenly	N	S	O
32.	Needs help from adult to stay on task	N	S	O
33.	Does not notice when his/her behavior causes negative reactions	N	S	O
34.	Leaves messes that others have to clean up even after instruction	N	S	O
35.	Has trouble changing activities	N	S	O
36.	Reacts more strongly to situations than other children	N	S	O
37.	Forgets what he/she is doing in the middle of an activity	N	S	O
38.	Does not realize that certain actions bother others	N	S	O
39.	Gets caught up in the small details of a task or situation and misses the main idea	N	S	O
40.	Has trouble "joining in" at unfamiliar social events (such as birthday parties, picnics, holiday gatherings)	N	S	O
41.	Is easily overwhelmed or overstimulated by typical daily activities	N	S	O

42.	Has trouble finishing tasks (such as games, puzzles, pretend play activities)	N	S	O
43.	Gets out of control more than playmates	N	S	O
44.	Cannot find things in room or play area even when given specific instructions	N	S	O
45.	Resists change of routine, foods, places, etc.	N	S	O
46.	After having a problem, will stay disappointed for a long time	N	S	O
47.	Cannot stay on the same topic when talking	N	S	O
48.	Talks or plays too loudly	N	S	O
49.	Does not complete tasks even after given directions	N	S	O
50.	Acts overwhelmed or overstimulated in crowded, busy situations (such as lots of noise, activity, or people)	N	S	O
51.	Has trouble getting started on activities or tasks even after instructed	N	S	O
52.	Acts too wild or out of control	N	S	O

53.	Does not try as hard as his/her ability on activities	N	S	O
54.	Has trouble putting the brakes on his/her actions even after being asked	N	S	O
55.	Unable to finish describing an event, person, or story	N	S	O
56.	Completes tasks or activities too quickly	N	S	O
57.	Is unaware when he/she does well and not well	N	S	O
58.	Gets easily sidetracked during activities	N	S	O
59.	Has trouble remembering something, even after a brief period of time	N	S	O
60.	Becomes too silly	N	S	O
61.	Has a short attention span	N	S	O
62.	Plays carelessly or recklessly in situations where he/she could be hurt (such as playground, swimming pool)	N	S	O
63.	Is unaware when he/she performs a task right or wrong	N	S	O

CPAS Selected

4.	Correcting Your Child (In General/Past Month)		
**Interviewer:** "In addition to showing affection, parents also need to correct their children. While young babies cannot learn lessons in this way, all children reach an age when parents must begin giving lessons.			
	NOTE: Q4a is deleted in version 4		
**Interviewer: "**We know that there are some common ways that parents in the community teach lessons to children when they do wrong, such as given one or two slaps, explaining, making afraid, or beating more harshly. We know that many parents think that one or two slaps and sometimes severe hitting are needed to keep the child on right path or to keep them safe. I would like to ask about things that [child]'s caregivers do to teach lessons. If you feel your child is too young to be disciplined, you can just tell me that." [*interviewer select "Not applicable"*]		5= Scolds/ বকা দেয়া<br>4= Beats lightly (e.g.one or two slaps)/<br>3= Beats more harshly (e.g. hitting hard)/ 2= Explains:<br>1= Explains first, then beats if child doesn't listen/ 0= Other (anything else)<br>9= Not applicable (*For coding only:*<br>7= Does not know<br>8= Declined to answer	
4b.	How do [child]'s mother and father usually correct when he/she does wrong? (choose up to 2 most common; choose "not applicable" if less than 2 are used commonly)	Mother /মা<br>4b1. |___| 4b2. |___|	Father /বাবা<br>4b3. |___| 4b4. |___|
	NOTE: Q4c is deleted in version 4		
**Interviewer: "**Approximately how often did [child]'s different caregivers do these things in the **past one month** to teach or correct [child]? I would like to know about things done by the mother, father (if present), or any other caregiver over 10 years of age who help correct [child] (e.g. any grandparent, aunty, uncle). You don't have to tell us which caregiver did these things, we would just like to know how often [child] experienced them from any caregiver, for instance for correction and improving of character or behavior."<br>[*If parent states that child is too young to be disciplined by any of the methods below, interviewer selects "**never**" for each.*]<br>"Did it happen in the past 1 month that [child]'s [mother/father/other caregiver]:" *[Read statement]*<br>"If yes, how often did it happen?" *[Read response options; If no, choose 'never']*		4= Most days or every day/<br>3= Once or twice per week/<br>2= Once or twice per month/<br>1= Less than once per month<br>0= Never<br>9= Not applicable /<br>(*For coding only:* 7= Does not know;<br>8= Declined to answer)	
	**Note:** ***In version 4, option to select responses individualy from mother, father and other caregiver is collapsed to one.***		
4d.	Beat lightly, e.g. one or two slaps?	4d1. |___|	
4e.	Explained what he/she did wrong?	4e1. |___|	
4f.	Beat him/her more harshly, e.g. hitting more than once or twice, causing pain or leaving red marks?	4f1. |___|	
	NOTE: Q4g is deleted in version 4		
4h.	Shut out of the house, e.g. out in the street?	4h1.|___|	
4i.	Beat with an object, e.g. stick, daa, stool?	4i1. |___|	
4j.	Beat so that he/she was cut, bleeding, part of the body was swollen, or there were blackish marks over the body?	4j1. |___|	
	NOTE: Q4k and Q4l are deleted in version 4		
4m.	Shook him/her?	4m1. |___|	
4n.	Criticized him/her, for instance calling names like lazy, dumb?	4n1. |___|	
4o.	"This is something that we know happens in families. It is ok to share. You don't have to be ashamed."<br>Cursed [child] with slang, e.g. son of a dog, son of a bitch?	4o1. |___|	

	NOTE: Q4p is deleted in version 4

4q.	Threatened to hit or beat child, for instance by raising hand, but did not hit?	4q1. |___|
4r.	Frightened [child] for disciplining, e.g. by talking about kidnapper, ghost, cat, cockroach etc?	4r1. |___|
4s.	"Sometimes we have to try to teach, and when we are very much angry we say so many things that are ugly and maybe we regret later. For instance, did you ever say you regret [child]'s life, things like saying it was a mistake to have given birth, telling to "just go and die," or telling to jump under a vehicle, or anything else like that?	4s1. |___|
	NOTE: Q4t is deleted in version 4	

➡	All participants continue here
**Interviewer:** "We also sometimes all become tired or frustrated. Parents might do things they later regret when they are in such a state, for instance, if their child is crying for a long time or refusing to sleep. They may do things like shaking or hitting their child, not for correction, but because of losing patience. Many parents have this experience, and you do not need to feel embarrassed about sharing your experience. I would like to know how often these things happened in the **past one month**."<br>"Did it happen in the past 1 month that:" *[Read statement]*<br>"If yes, how often did it happen?" *[Read response options; If no, choose 'never']*	4= Most days or every day<br>3= Once or twice per week<br>2= Once or twice per month<br>1= Less than once per month<br>0= Never<br>9= Not applicable<br>(*For coding only:*<br>7= Does not know<br>8= Declined to answer)

4u.	[Child]'s caregivers shook or hit [child] for a reason other than for correction (e.g. because tired, frustrated)?	|___|
4v.	[Child]'s caregivers burned or scalded [child] intentionally (not an accident) for a reason other than for correction?	|___|
4w.	[Child]'s caregivers scolded or spoke with an angry voice to [child] for reasons other than correction?	|___|

**5.**	**Home life, Part 1—Challenges in families**
	NOTE: Q5a, Q5b, Q5c and Q5d are deleted in version **4**
**Interviewer:** "I would like to know if these things happened in your marriage or family in the **past 6 months** or during your **pregnancy with [child]**. For these questions, I will ask again if these things happened always, frequently, sometimes, or rarely, or never during these time periods. If you were not married during these times, you can tell me that and we will skip some questions." [*interviewer enter "not applicable]*<br>"Did it happen [in the past 6 months]/[during pregnancy] that:" *[Read statement]*<br>If yes, did it happen always, frequently, sometimes, or rarely?" *[If no, choose 'never']*	4=Always<br>3= Frequently<br>2= Sometimes<br>1= Rarely<br>0= Never<br>9= Not applicable<br>(*For coding only:*<br>7=Does not remember<br>8= Declined to answer)

5e.	Your husband neglected giving money to support your family?	5e1. Past 6m |___|	5e2.Pregnancy |___|
5f.	Your husband and you lived separately but remained married, or your husband abandoned your family, because of arguments or marital trouble?	5f1. Past 6m / |___|	5f2.Pregnancy |___|
	NOTE: Q5g is deleted in version 4		

5h.	Your husband had relations with another woman (or women)?	5h1. Past 6m / |___|	5h2.Pregnancy |___|
5i.	Your husband criticized you for many things, for instance about cooking, care of children, housework, etc.?	5i1. Past 6m |___|	5i2.Pregnancy |___|
5j.	You had quarrels with your husband?	5j1. Past 6m / গত ছয়মাসে |___|	5j2.Pregnancy/গর্ভাবস্থায় |___|
5k.	You were unhappy because your husband did not go for earning money?	5k1. Past 6m / গত ছয়মাসে |___|	5k2.Pregnancy/গর্ভাবস্থায় |___|
5l.	Your husband threatened to send you back to your village permanently because he was unhappy with you?	5l1. Past 6m / গত ছয়মাসে |___|	5l2.Pregnancy/গর্ভাবস্থায় |___|
5m.	Your husband put pressure to get money from you or your family?	5m1. Past 6m / গত ছয়মাসে |___|	5m2.Pregnancy/গর্ভাবস্থায় |___|
5n.	Your husband denied you food?	5n1. Past 6m / গত ছয়মাসে |___|	5n2.Pregnancy/গর্ভাবস্থায় |___|
	NOTE: Q5o is deleted in version 4		

**6.**	**Home life, Part 2—Family Conflict & Social Support**		
	NOTE: Q6a is deleted in version 4		
**Interviewer:** "I would like to know if the following things happened always, frequently, sometimes, rarely, or never in the **past 6 months**, and during our **pregnancy with [child]**. If you did not have a husband during those times, or if you did not see your husband's family (e.g. because you were back at your mother's house), just tell me that." *[Interviewer select 'not applicable']*<br>"Did it happen [in the past 6 months]/[during pregnancy] that:" *[Read statement]*<br>"If yes, did it happen always, frequently, sometimes, or rarely?" *[If no, choose 'never']*			4= Most days or every day<br>3= Once or twice per week<br>2= Once or twice per month<br>1= Less than once per month<br>0= Never<br>9= Not applicable<br>(*For coding only:*<br>7= Does not know<br>8= Declined to answer)
6b.	Your husband's family criticized you, for instance about cooking, care of children, housework?	6b1. Past 6m / গত ছয়মাসে |___|	6b2.Pregnancy/গর্ভাবস্থায় |___|
	NOTE: Q6c deleted in version 4		
6d.	You quarreled with your husband's family?	6d1. Past 6m / গত ছয়মাসে |___|	6d2.Pregnancy/গর্ভাবস্থায় |___|
6e.	Your husband's family threatened to send you back to your village or mother/father's house permanently because they were unhappy with you?	6e1. Past 6m / গত ছয়মাসে |___|	6e2.Pregnancy/গর্ভাবস্থায় |___|
	NOTE: Q6f, Q6g and Q6h are deleted in version 4		

➡	**All participants continue here**	
**Interviewer:** "Now I'd like to know about some other things that members of the community sometimes experience. I would like to know if the following things happened in the **past 6 months** and during your **pregnancy with [child]**. I would like to know if they happened always, frequently, sometimes, rarely, or never."		4= Most days or every day<br>3= Once or twice per week<br>2= Once or twice per month<br>1= Less than once per month

"Did it happen [in the past 6 months]/[during pregnancy] that:" *[Read statement]*<br>"If yes, did it happen always, frequently, sometimes, or rarely?" *[If no, choose 'never']*			0= Never<br>9= Not applicable<br>(*For coding only:*<br>7= Does not know<br>8= Declined to answer)
6i.	You felt like home was like Hell or you had no peace at home?	6i1. Past 6m / গত ছয়মাসে |___|	6i2.Pregnancy/গর্ভাবস্থায় |___|
6j.	You felt all alone, like you had no one in your daily life to share your sorrows and troubles with (in person, by phone, etc.)?	6j1. Past 6m / গত ছয়মাসে |___|	6j2.Pregnancy/গর্ভাবস্থায় |___|
6k.	You felt like you had no one in your daily life who could make you laugh and cheer you up?	6k1. Past 6m / |___|	6k2.Pregnancy |___|
	NOTE: Q6l is deleted in version 4		
6m.	You felt like there was no one in your daily life who gave you praise, kindness, or encouragement?	6m1. Past 6m / |___|	6m2.Pregnancy |___|
6n.	You felt like you had no one in your daily life who could give you advice about how to solve problems or to do new/difficult things?	6n1. Past 6m / ছয়মাসে |___|	6n2.Pregnancy/গর্ভাবস্থায় |___|

**7.**	**Home life, Part 3—Intimate Partner Violence**		
**Interviewer:** "We also know sometimes husbands, or other men in women's lives (*e.g. boyfriend, father-in-law, brother-in-law*), can use violence towards wives or partners. Many women say that their husbands or other men beat or curse at them sometimes. There is no need to be ashamed of this. You are safe to share these things here, and they are confidential."			
7a.	Has husband (or other man close to you) ever beat you or threatened to beat or abuse you physically?	1= Yes/ হ্যাঁ<br>0= No/ না<br>(*For coding only:*<br>8= Declined to answer/	|__|
➡	**<u>If yes:</u>** Go to 7b<br><u>উত্তর "হ্যা"</u> n‡j: 7b প্রশ্ন করতে হবে।	**<u>If no:</u>** Skip to 7l<br><u>উত্তর "না"</u> n‡j: স্কিপ করে 7l প্রশ্ন করতে হবে।	

**Interviewer:** "Please tell me if your husband *or any other man close to you (e.g. boyfriend, father-in-law, brother-in-law)* did these things to you in the **past 6 months** or during your **pregnancy with [child]**. I would like to know if they did them always, frequently, sometimes, rarely, or never."<br>"Did it happen [in the past 6 months]/[during pregnancy] that your husband or any other many close to you:" *[Read statement]*<br>If yes, how often did it happen?" *[Read response options; If no, choose 'never']*			4= Most days or every day<br>3= Once or twice per week<br>2= Once or twice per month<br>1= Less than once per month<br>0= Never<br>9= Not applicable<br>(*For coding only:*<br>7= Does not know<br>8= Declined to answer)
7b.	Beat you a little, for instance one or two slaps?	7b1. Past 6m / গত ছয়মাসে |___|	7b2.Pregnancy/গর্ভাবস্থায় |___|
7c.	Beat you harshly, e.g. causing a lot of pain or leaving swelling, bruises, or injuries?	7c1. Past 6m / গত ছয়মাসে |___|	7c2.Pregnancy/গর্ভাবস্থায় |___|
7d.	Beat you with an object, e.g. stick, daa, tool?	7d1. Past 6m / গত ছয়মাসে |___|	7d2.Pregnancy/গর্ভাবস্থায় |___|
7e.	Pushed you, shook you, or threw something at you?	7e1. Past 6m / গত ছয়মাসে |___|	7e2.Pregnancy/গর্ভাবস্থায় |___|
7f.	Kicked you, dragged you, or beat you up (beat continuously)?	7f1. Past 6m / গত ছয়মাসে |___|	7f2.Pregnancy/গর্ভাবস্থায় |___|

	NOTE: Q7g is deleted in version 4		
7h.	Threatened to beat you but did not beat you, or did other things to make you afraid?	7h1. Past 6m / গত ছয়মাসে |___|	7h2.Pregnancy/গর্ভাবস্থায় |___|
7i.	Attacked you with a weapon, e.g. knife, gun, machete, scythe?	7i1. Past 6m / গত ছয়মাসে |___|	7i2.Pregnancy/গর্ভাবস্থায় |___|
7j.	You were afraid he would kill you by beating or abuse?	7j1. Past 6m / গত ছয়মাসে |___|	7j2.Pregnancy/গর্ভাবস্থায় |___|
	NOTE: Q7k is deleted in version 4		
➡	*Proceed directly to 7l after answering 7j WITHOUT reading following interviewer paragraph 7j*		

*Read interviewer paragraph <u>only if participant</u> **<u>skipped 7b-7j.</u>***<br>➡ **Interviewer:** "Please tell me if your husband *or any other man close to you* (e.g. boyfriend, uncle) did these things to you in the **past 6 months** or during your **pregnancy with [child]**."<br>"Did it happen [in the past 6 months]/[during pregnancy] that your husband or any other many close to you:" *[Read statement]*<br>"If yes, how often did it happen?" *[Read response options; If no, choose 'never']*	4= Most days or every day<br>3= Once or twice per week<br>2= Once or twice per month<br>1= Less than once per month<br>0= Never<br>9= Not applicable<br>(*For coding only:*<br>7= Does not know<br>8= Declined to answer)

7l.	Insulted you with slang or insults, e.g. calling you dumb or lazy, or cursing you?	7l1. Past 6m / |___|	7l2. Pregnancy |___|
7m.	Made you feel ashamed or humiliated in front of others?	7m1. Past 6m / গত ছয়মাসে |___|	7m2.Pregnancy/গর্ভাবস্থায় |___|
	NOTE: Q7n and Q7o is deleted in version 4		

**Interviewer:** "Did it happen in the **past 6 months** that: *[Read statement]*<br>"If yes, how often did it happen?" *[Read response options; If no, choose 'never']*	4= Most days or every day<br>3= Once or twice per week<br>2= Once or twice per month<br>1= Less than once per month<br>0= Never<br>9= Not applicable<br>(*For coding only:*<br>7= Does not know<br>8= Declined to answer)

7p.	[Child] saw/heard you being beaten by anyone close to you (e.g. by husband, mother-in-law, other)?	Past 6m / গত ছয় মাসে |___|
7q.	[Child] saw/heard you being yelled at or humiliated by anyone close to you (e.g. by husband, mother-in-law, other)?	Past 6m / গত ছয় মাসে |___|

Sdq

Study ID ______________________________

Strengths and Difficulties Questionnaire

For each item, please choose Not True, Somewhat True or Certainly True. It would help us if you answered all items as best you can even if you are not absolutely certain. Please give your answers on the basis of your child's behavior over the last six months.

1. Considerate of other people's feelings
   - ◯ Not True
   - ◯ Somewhat True
   - ◯ Certainly True
   - ◯ I do not wish to respond

2. Restless, overactive, cannot stay still for long
   - ◯ Not True
   - ◯ Somewhat True
   - ◯ Certainly True
   - ◯ I do not wish to respond

3. Often complains of headaches, stomach-aches or sickness
   - ◯ Not True
   - ◯ Somewhat True
   - ◯ Certainly True
   - ◯ I do not wish to respond

4. Shares readily with other children, for example toys, treats, pencils
   - ◯ Not True
   - ◯ Somewhat True
   - ◯ Certainly True
   - ◯ I do not wish to respond

5. Often loses temper
   - ◯ Not True
   - ◯ Somewhat True
   - ◯ Certainly True
   - ◯ I do not wish to respond

6. Rather solitary, prefers to play alone
   - ◯ Not True
   - ◯ Somewhat True
   - ◯ Certainly True
   - ◯ I do not wish to respond

7. Generally well behaved, usually does what adults request
   - ◯ Not True
   - ◯ Somewhat True
   - ◯ Certainly True
   - ◯ I do not wish to respond

8. Many worries or often seems worried
   - ◯ Not True
   - ◯ Somewhat True
   - ◯ Certainly True
   - ◯ I do not wish to respond

9. Helpful if someone is hurt, upset or feeling ill
   - ◯ Not True
   - ◯ Somewhat True
   - ◯ Certainly True
   - ◯ I do not wish to respond

10. Constantly fidgeting or squirming	◯ Not True<br>◯ Somewhat True<br>◯ Certainly True<br>◯ I do not wish to respond
11. Has at least one good friend	◯ Not True<br>◯ Somewhat True<br>◯ Certainly True<br>◯ I do not wish to respond
12. Often fights with other children or bullies them	◯ Not True<br>◯ Somewhat True<br>◯ Certainly True<br>◯ I do not wish to respond
13. Often unhappy, depressed or tearful	◯ Not True<br>◯ Somewhat True<br>◯ Certainly True<br>◯ I do not wish to respond
14. Generally liked by other children	◯ Not True<br>◯ Somewhat True<br>◯ Certainly True<br>◯ I do not wish to respond
15. Easily distracted, concentration wanders	◯ Not True<br>◯ Somewhat True<br>◯ Certainly True<br>◯ I do not wish to respond
16. Nervous or clingy in new situations, easily loses confidence	◯ Not True<br>◯ Somewhat True<br>◯ Certainly True<br>◯ I do not wish to respond
17. Kind to younger children	◯ Not True<br>◯ Somewhat True<br>◯ Certainly True<br>◯ I do not wish to respond
18. Often lies or cheats	◯ Not True<br>◯ Somewhat True<br>◯ Certainly True<br>◯ I do not wish to respond
19. Picked on or bullied by other children	◯ Not True<br>◯ Somewhat True<br>◯ Certainly True<br>◯ I do not wish to respond
20. Often offers to help others (parents, teachers, other children)	◯ Not True<br>◯ Somewhat True<br>◯ Certainly True<br>◯ I do not wish to respond

21. Thinks things out before acting	◯ Not True<br>◯ Somewhat True<br>◯ Certainly True<br>◯ I do not wish to respond
22. Steals from home, school or elsewhere	◯ Not True<br>◯ Somewhat True<br>◯ Certainly True<br>◯ I do not wish to respond
23. Gets along better with adults than with other children	◯ Not True<br>◯ Somewhat True<br>◯ Certainly True<br>◯ I do not wish to respond
24. Many fears, easily scared	◯ Not True<br>◯ Somewhat True<br>◯ Certainly True<br>◯ I do not wish to respond
25. Good attention span, sees chores or homework through to the end	◯ Not True<br>◯ Somewhat True<br>◯ Certainly True<br>◯ I do not wish to respond
26. Overall, do you think that your child has difficulties in one or more of the following areas: emotions, concentration, behavior, or being able to get on with other people?	◯ No<br>◯ Yes - minor difficulties<br>◯ Yes - definite difficulties<br>◯ Yes - severe difficulties<br>◯ I do not wish to respond
27. How long have these difficulties been present?	◯ Less than a month<br>◯ 1-5 months<br>◯ 6-12 months<br>◯ Over a year<br>◯ I do not wish to respond
28. Do the difficulties upset or distress your child?	◯ Not at all<br>◯ Only a little<br>◯ A medium amount<br>◯ A great deal<br>◯ I do not wish to respond
29. Do the difficulties interfere with your child's everyday life in the following areas?	
HOME LIFE	◯ Not at all<br>◯ Only a little<br>◯ A medium amount<br>◯ A great deal<br>◯ I do not wish to respond
FRIENDSHIPS	◯ Not at all<br>◯ Only a little<br>◯ A medium amount<br>◯ A great deal<br>◯ I do not wish to respond

CLASSROOM LEARNING	◯ Not at all<br>◯ Only a little<br>◯ A medium amount<br>◯ A great deal<br>◯ I do not wish to respond
LEISURE ACTIVITIES	◯ Not at all<br>◯ Only a little<br>◯ A medium amount<br>◯ A great deal<br>◯ I do not wish to respond
30. Do the difficulties put a burden on you or the family as a whole?	◯ Not at all<br>◯ Only a little<br>◯ A medium amount<br>◯ A great deal<br>◯ I do not wish to respond

Epidemic – Pandemic Impacts Inventory – Brief Form (EPII-B)

Damion J. Grasso, Ph.D.[1]
Margaret J. Briggs-Gowan, Ph.D.[1]
Julian D. Ford, Ph.D., ABPP[1]
Alice S. Carter, Ph.D.[2]

[1]*University of Connecticut School of Medicine*
[2]*University of Massachusetts*

PURPOSE: The EPII is a tool designed to assess tangible impacts of epidemics and pandemics across personal and social life domains.

DEVELOPMENT: Candidate items were constructed by a team of clinical and developmental psychologists with expertise in assessment of stress, trauma, resilience, and coping. Feedback from professionals across multiple disciplines (e.g., social work, pediatrics, medicine, anthropology) was incorporated in selecting and refining final items for of the measure, which was accomplished via expert consensus.

PSYCHOMETRICS: Because the EPII is newly developed, psychometric properties are still being established. Use of the EPII in research studies will help to establish psychometric properties and will likely result in refinement of the tool. Please see the following for initial results from a survey of individuals residing in the Northeast region of the United States: https://psyarxiv.com/v36hj/

SCORING. Optimal scoring procedures are not yet determined and will be informed by future research.

PERMISSIONS. Researchers are welcome and encouraged to use the EPII in their research studies. Researchers may disseminate the survey using a paper format or may convert items to an online survey format so long as the integrity of the instructions and items is maintained. Users shall not modify items without permission from the developers. Please inform us of your intention to use the instrument by sending an Email to Dr. Damion Grasso at dgrasso@uchc.edu with the following information: (1) Principal Investigator(s), (2) Purpose of research study, (3) Population(s) studied, and (4) Study location(s).

Suggested Citation

Grasso, D.J., Briggs-Gowan, M.J., Ford, J.D., & Carter, A.S. (2020). *The Epidemic – Pandemic Impacts Inventory – Brief Form (EPII-B).* University of Connecticut School of Medicine.

 All Rights Reserved

Epidemic-Pandemic Impacts Inventory (EPII) – Brief Form

INSTRUCTIONS

We would like to learn how COVID-19 has changed people's lives. For each statement below, please indicate whether the pandemic has impacted you or your family in the way described.

**Since the COVID-19 pandemic began, what has changed for you or your family?**					
**1. I (or someone in my home) was laid off, furloughed, had to close a business, or had reduced work hours**	**0** Did not happen	**1** Happened but no impact on me or my family	**2** Some impact on me or my family	**3** A lot of impact on me or my family	**4** Extreme impact on me or my family
**2. I (or someone in my home) had to work in close contact with people who might be infected**	**0** Did not happen	**1** Happened but no impact on me or my family	**2** Some impact on me or my family	**3** A lot of impact on me or my family	**4** Extreme impact on me or my family
**3. I (or someone in my home) had an increase in workload or work responsibilities**	**0** Did not happen	**1** Happened but no impact on me or my family	**2** Some impact on me or my family	**3** A lot of impact on me or my family	**4** Extreme impact on me or my family
**4. I (or someone in my home) provided direct care or services to people who had the disease**	**0** Did not happen	**1** Happened but no impact on me or my family	**2** Some impact on me or my family	**3** A lot of impact on me or my family	**4** Extreme impact on me or my family
**5. A child, teenager, or young adult I care for could not go to school or needed home instruction**	**0** Did not happen	**1** Happened but no impact on me or my family	**2** Some impact on me or my family	**3** A lot of impact on me or my family	**4** Extreme impact on me or my family
**6. Childcare or babysitting was unavailable to me (or someone in my home) when needed**	**0** Did not happen	**1** Happened but no impact on me or my family	**2** Some impact on me or my family	**3** A lot of impact on me or my family	**4** Extreme impact on me or my family

7.	**I had more conflict with my child(ren) or I was harsher in disciplining them.**	**0** Did not happen	**1** Happened but no impact on me or my family	**2** Some impact on me or my family	**3** A lot of impact on me or my family	**4** Extreme impact on me or my family
8.	**Another caregiver in my home had more conflict with my child(ren) or was harsher in disciplining them.**	**0** Did not happen	**1** Happened but no impact on me or my family	**2** Some impact on me or my family	**3** A lot of impact on me or my family	**4** Extreme impact on me or my family
9.	**I spent a lot more time taking care of a family member**	**0** Did not happen	**1** Happened but no impact on me or my family	**2** Some impact on me or my family	**3** A lot of impact on me or my family	**4** Extreme impact on me or my family
10.	**My family had to move, relocate, was evicted, or became homeless**	**0** Did not happen	**1** Happened but no impact on me or my family	**2** Some impact on me or my family	**3** A lot of impact on me or my family	**4** Extreme impact on me or my family
11.	**There was an increase in verbal or physical conflict with a partner or spouse**	**0** Did not happen	**1** Happened but no impact on me or my family	**2** Some impact on me or my family	**3** A lot of impact on me or my family	**4** Extreme impact on me or my family
12.	**There was an increase in verbal or physical conflict among other family in my home**	**0** Did not happen	**1** Happened but no impact on me or my family	**2** Some impact on me or my family	**3** A lot of impact on me or my family	**4** Extreme impact on me or my family
13.	**My family was unable to pay for or get enough food or clean water**	**0** Did not happen	**1** Happened but no impact on me or my family	**2** Some impact on me or my family	**3** A lot of impact on me or my family	**4** Extreme impact on me or my family
14.	**My family was unable to pay important large bills, like rent or utilities**	**0** Did not happen	**1** Happened but no impact on me or my family	**2** Some impact on me or my family	**3** A lot of impact on me or my family	**4** Extreme impact on me or my family

 All Rights Reserved

15.	**My family had trouble getting places due to less access to public transportation or concerns about safety**	**0** Did not happen	**1** Happened but no impact on me or my family	**2** Some impact on me or my family	**3** A lot of impact on me or my family	**4** Extreme impact on me or my family
16.	**I (or someone in my home) did not have the ability to talk to or see family or friends while separated**	**0** Did not happen	**1** Happened but no impact on me or my family	**2** Some impact on me or my family	**3** A lot of impact on me or my family	**4** Extreme impact on me or my family
17.	**My family had to cancel or could not attend important celebrations (such as weddings) or religious ceremonies or funerals**	**0** Did not happen	**1** Happened but no impact on me or my family	**2** Some impact on me or my family	**3** A lot of impact on me or my family	**4** Extreme impact on me or my family
18.	**I (or someone in my home) was unable to be with a close family member who was hospitalized, in a nursing home, or in critical condition**	**0** Did not happen	**1** Happened but no impact on me or my family	**2** Some impact on me or my family	**3** A lot of impact on me or my family	**4** Extreme impact on me or my family
19.	**I (or someone in my home) was isolated or quarantined due to possible exposure to the disease, symptoms, or increased risk**	**0** Did not happen	**1** Happened but no impact on me or my family	**2** Some impact on me or my family	**3** A lot of impact on me or my family	**4** Extreme impact on me or my family
20.	**I had limited physical closeness with my child or loved one due to concerns of infection**	**0** Did not happen	**1** Happened but no impact on me or my family	**2** Some impact on me or my family	**3** A lot of impact on me or my family	**4** Extreme impact on me or my family
21.	**My child[ren] had more frequent or severe behavioral or emotional problems (for example, mood, anxiety, sleep, nightmares)**	**0** Did not happen	**1** Happened but no impact on me or my family	**2** Some impact on me or my family	**3** A lot of impact on me or my family	**4** Extreme impact on me or my family
22.	**I (or someone in my home) had more frequent or severe mental health problems, sleep problems, or use of alcohol or substances**	**0** Did not happen	**1** Happened but no impact on me or my family	**2** Some impact on me or my family	**3** A lot of impact on me or my family	**4** Extreme impact on me or my family

23.	**I (or someone in my home) was unable to access or was less satisfied with mental health treatment or therapy**	**0** Did not happen	**1** Happened but no impact on me or my family	**2** Some impact on me or my family	**3** A lot of impact on me or my family	**4** Extreme impact on me or my family
24.	**I (or someone in my home) could not get enough medication or medical treatment for a chronic illness or pain**	**0** Did not happen	**1** Happened but no impact on me or my family	**2** Some impact on me or my family	**3** A lot of impact on me or my family	**4** Extreme impact on me or my family
25.	**I (or someone in my home) got less exercise, spent more time sitting down, or ate more junk food**	**0** Did not happen	**1** Happened but no impact on me or my family	**2** Some impact on me or my family	**3** A lot of impact on me or my family	**4** Extreme impact on me or my family
26.	**I (or someone in my home) had important medical procedures cancelled or was unable to access medical care for a serious condition**	**0** Did not happen	**1** Happened but no impact on me or my family	**2** Some impact on me or my family	**3** A lot of impact on me or my family	**4** Extreme impact on me or my family
27.	**I (or someone in my home) tested positive for COVID-19 and had severe symptoms**	**0** Did not happen	**1** Happened but no impact on me or my family	**2** Some impact on me or my family	**3** A lot of impact on me or my family	**4** Extreme impact on me or my family
28.	**A close friend or family member died from COVID-19 or related complications**	**0** Did not happen	**1** Happened but no impact on me or my family	**2** Some impact on me or my family	**3** A lot of impact on me or my family	**4** Extreme impact on me or my family
29.	**My family enjoyed more quality time together, paid more attention to personal health, or made new connections with one another or with friends**	**0** Did not happen	**1** Happened but no impact on me or my family	**2** Some impact on me or my family	**3** A lot of impact on me or my family	**4** Extreme impact on me or my family
30.	**I (or someone in my home) was harassed or blamed for causing or spreading COVID-19 because of my (or their) race/ethnicity**	**0** Did not happen	**1** Happened but no impact on me or my family	**2** Some impact on me or my family	**3** A lot of impact on me or my family	**4** Extreme impact on me or my family

31.	**I (or someone in my home) was denied or unable to access services or treatment for COVID-19 because of my (or their) race/ethnicity**	**0** Did not happen	**1** Happened but no impact on me or my family	**2** Some impact on me or my family	**3** A lot of impact on me or my family	**4** Extreme impact on me or my family
32.	**I found greater meaning and was more effective in my work, school, or friendships than before COVID-19**	**0** Did not happen	**1** Happened but no impact on me or my family	**2** Some impact on me or my family	**3** A lot of impact on me or my family	**4** Extreme impact on me or my family

33. Overall, how upset have you been by how COVID-19 has affected you and your family?

**0**	**1**	**2**	**3**	**4**
Not at all upset	A little upset	Somewhat upset	Very upset	Extremely upset

 All Rights Reserved

PR#21084		SID:------------------------------

Multidimensional evaluation of the early emergence of executive function and dysfunction in young children in Bangladesh using nutritional and psychosocial intervention: A Pilot study

Family Care Indicators(FCI)

ASSESSMENT INFORMATION TO BE DATA ENTERED

1	Visit month	○ 12<br>○ 24<br>○ 36
1a	Date of visit<br>**(If not assessed, enter 09/09/99)**	|__||__| / |__||__| / |__||__| *D D M M Y Y*

(This questionnaire to be asked to mother and caretaker)

Interviewer: "I am interested in learning about the things that [CHILD] plays with when he is at home". "Can you please bring me all toys that your child plays with?"

Instructions: The toys may be home-made (like clay toys, dolls made of cloths, etc.), household materials (like pots and pans, crockery, pillow, school bag, mobile phone etc.), bought toys, children books/ picture books (can be bought/ received from school or someone free of charge) and the child should have access to play with at home during the last month.
DO NOT include play at playgroup

সাক্ষা□কার গ্রহনকারী মা/ পরচিরি্যাকারীকে বলুন যে " শশিু বাড়তিে যা যা দয়িে খলেে, আমি সে সমস্ত জনিসি সম্পর্কে জানতে আগ্রহী"। খলেনাগুলো বাড়তিে তরৈি হতে পারে (যমেনঃ ঘররে তরৈি মাটরি খলেনা, কাপড়রে তরৈি পুতুল, ইত্যাদি), ঘরে ব্যাবহাররে জনিসিপত্র হতে পারে (যমেনঃ হারপাতিলি, বালসি, স্কুলব্যাগ, মোবাইল, ইত্যাদি), কনো খলেনা হতে পারে, শশিুর উপযোগী বই/ ছবরি বই হতে পারে (স্কুলরে বই বা কার কাছ থকেে ফ্রি পাওয়া বই নয়) ইত্যাদি।

2	**Play Materials:**		
	2a. In the past 30 days, has [CHILD] played with toys that make or play music (e.g. Instrument, stuffed animals that play melodies or any other toys that make noise, given to the child to play)?<br>গত ৩০ দিনে [শিশু] এমন কোন খেলনা দিয়ে খেলেছে বা বাজনা বাজিয়েছে, শুধুমাত্র শব্দ নয় (যেমন যন্ত্র, খেলনা পশু যা শব্দ তৈরী করে বা এমন যেকোনো খেলনা যা শব্দ তৈরী করে, তবে এটা শিশুকে খেলতে দিতে হবে)?	0 = No 1 = Yes	|___|

Interviewer: These toys must make sound/music, not just noise (e.g. instruments such as drum, piano, harmonica flute, harmonium, jory, etc.
যেমন শুধু শব্দ বা আওয়াজ হয় এমন খেলনা নয়, যা দিয়ে সুর তোলা যায়। যেমন-,ঢোল, পিয়ানো,মাউথ অর্গান, হারমোনিয়াম, জুরি, বাঁশের বাঁশি ইত্যাদি।
Instructions: Instruments can be real instruments or toy instruments. Only include things that are played at home.

	2b. In the past 30 days, has the (CHILD) played with materials for drawing and writing (e.g. coloring picture books, crayons, pencils, pens etc.)?<br>গত ৩০ দিনে [শিশু] কি আঁকা বা লেখার উপকরণ দিয়ে খেলেছে? (যেমন: রং করার জন্যে ছবির বই, পেন্সিল, কলম ইত্যাদি) যা বাচ্চা আঁকাআঁকি করে।	0 = No 1 = Yes	|___|

Instructions: If child cannot draw but mother gives items to the child, score 1= Yes
(বাচ্চা আঁকা আঁকি না পারলেও মা যদি এগুলো বাচ্চার হাতে দেয় তাহলে স্কোর হ্যাঁ হবে।
DO NOT include play at playgroup

	2c. In the past 30 days, has [CHILD] played using toys or objects as, something or someone else, such as a Mommy, doctor, teacher, hero using toys or objects (e.g. dolls, tea-set/ cups, toy kitchen set and plates for eating)?<br>গত ৩০ দিনে শিশু কি এমন কোনো খেলনা বা জিনিস দিয়ে কোন কিছু সেজে যেমন:মা, ডাক্তার, শিক্ষক, নায়ক খেলনা বা জিনিস দিয়ে খেলেছে? (যেমন পুতুল, চায়ের সেট/ কাপ, খেলনা হাড়িপাতিল, খাওয়ার জন্য পেট ইত্যাদি)	0 = No 1 = Yes	|___|

	2d. In the past 30 days, has [CHILD] played with toys that (Gross Motor) encourage movement (e.g. balls, small car, skipping rope, bats, rope for swinging, pull-along, push alone etc.) ?<br>গত ৩০ দিনে [শিশু] কি ছুটাছুটি করে খেলতে পারে এমন কোনো খেলনা দিয়ে খেলেছে?(যেমন বল, ছোট গাড়ি, দড়ি লাফানো, ব্যাট, দড়ি দিয়ে বানানো দোলনা, টানা বা ঠেলা দেওয়া গাড়ী ইত্যাদি)।	0 = No 1 = Yes	|___|
	2e. Have you any toys to teach shapes and colors (triangle, square,round)?<br>বাচ্চার এমন কোন খেলনা আছে যা দিয়ে বিভিন্ন আকৃতি (তিনকোণা, চারকোণা,গোল) এবং রং শেখা যায়।	0 = No 1 = Yes	|___|
3	**Household books, magazines and newspapers**		

Interviewer: "Now I will ask you some questions about books"

Instructions: If there are more than 10 books or magazines, record 11 in Q3a &3b.

	3a.How many books for children including picture books are there in the household?<br>**(Please do not include school books, but include other books meant for children, such as picture books)**<br>বাচ্চাদের উপযোগী কয়টি বই, ছবির বই আছে (স্কুল বই ছাড়া)	|___||___|
	3b. How many magazines and newspaper are in the house?<br>বাড়িতে কয়টি পত্রিকা / পেপার এবং ম্যাগাজিন আছে?	|___||___|
4	**Activities in the past three days**	

Interviewer: "In the past 3 days, did you or any household member (over 15 years of age) engage in any of the following actives with the child (Name)?" "If yes, who engaged this activity with child? (Mother, Father, or any others adult family members of the household including caregiver)".
সাক্ষাৎকার গ্রহণকারী বলবে: গত তিন (3) দিনে আপনি বা বাড়ীর বড় কেউ (১৫ বৎসরের উপরে) বাচ্চার সাথেনিম্নলিখিত কাজগুলো করেছেন? হ্যাঁ হলে কে করেছেন? মা, বাবা বা পরিবারের অন্য বড় কেউ পরিচর্যাকারী

	4a.Read books, poems or look at picture books, to with [Child's name]?<br>বাচ্চাকে (নাম) বই পড়ে / কবিতার বই পড়ে শুনিয়েছেন বা ছবির বই দেখিয়েছেন? যদি হ্যাঁ হয়,		0=No 1=Yes	|___|
	Who engaged in this activity with [Child's name]?<br>কে এই কাজে যুক্ত ছিল?	4a.1. Mother মা	0=No 1=Yes	|___|
		4a.2. Father বাবা	0=No 1=Yes	|___|
		4a.3. Other অন্যান্য	0=No 1=Yes	|___|
	4b.Tell stories, nursery rhythms to with [Child's name]?<br>বাচ্চাকে (নাম) গল্প, কবিতা বা ছড়া বলেছেন?		0=No 1=Yes	|___|
	Who engaged in this activity with [Child's name]?<br>কে এই কাজে যুক্ত ছিল?	4b.1. Mother মা	0=No 1=Yes	|___|
		4b.2. Father বাবা	0=No 1=Yes	|___|
		4b.3. Other অন্যান্য	0=No 1=Yes	|___|
	4c.Sing songs / including lullabies with [Child's name]? বাচ্চার (নাম) সাথে গান / গজল গেয়েছেন?		0=No 1=Yes	|___|
	Who engaged in this activity with [Child's name]?<br>কে এই কাজে যুক্ত ছিল?	4c.1. Mother মা	0=No 1=Yes	|___|
		4c.2. Father বাবা	0=No 1=Yes	|___|
		4c.3. Other অন্যান্য	0=No 1=Yes	|___|
	4d.Play with [Child's name] with toys? বাচ্চাকে (নাম) সাথে খেলনা দিয়ে খেলেছেন?		0=No 1=Yes	|___|
	Who engaged in this activity with [Child's name]?<br>কে এই কাজে যুক্ত ছিল?	4d.1. Mother মা	0=No 1=Yes	|___|
		4d.2. Father বাবা	0=No 1=Yes	|___|
		4d.3. Other অন্যান্য	0=No 1=Yes	|___|
	4e.Spend time with [Child's name] naming, counting, and/or drawing things? বাচ্চাকে (নাম) সময় দিয়ে কোনো কিছুর নাম, গণণা এবং আঁকাআঁকি শিখিয়েছেন?		0=No 1=Yes	|___|

	Who engaged in this activity with [Child’s name]? কে এই কাজে যুক্ত ছিল?	4e.1. Mother মা	0=No 1=Yes	|___|
		4e.2. Father বাবা	0=No 1=Yes	|___|
		4e.3. Other অন্যান্য	0=No 1=Yes	|___|
	4f.Spend time with [Child’s name]; did you or any other adult play with the child using hand and leg fingers? (peek-a-boo, hide & seek, clap hand etc.) বাচ্চাকে (নাম) সময় দিয়ে হাতে পায়ের আঙুল দিয়ে লুকোচুরি, উকিটুক্কা, তাইতাই,হাতের তালুতে দুধ দিযে ভাত দিয়ে এমন কোন খেলা খেলেছেন?		0=No 1=Yes	|___|
	Who engaged in this activity with [Child’s name]? কে এই কাজে যুক্ত ছিল?	4f.1. Mother মা	0=No 1=Yes	|___|
		4f.2. Father বাবা	0=No 1=Yes	|___|
		4f.3. Other অন্যান্য	0=No 1=Yes	|___|
	4g.Spend time with [Child’s name]; chatting, talking (conversation). বাচ্চাকে (নাম) সময় দিয়ে গল্পের ছলে কথা বলেছেন?		0=No 1=Yes	|___|
	Who engaged in this activity with [Child’s name]? কে এই কাজে যুক্ত ছিল?	4g.1. Mother মা	0=No 1=Yes	|___|
		4g.2. Father বাবা	0=No 1=Yes	|___|
		4g.3. Other অন্যান্য	0=No 1=Yes	|___|
5	TOTAL SCORE<br>(Add all numbers in right column, including questions 3a and 3b)			|___||___|

NOT DATA ENTERED

	Interviewer Name and Code	________________________________	|___||___||___|

**PR#21084**		SID:------------------------------

Multidimensional evaluation of the early emergence of executive function and dysfunction in young children in Bangladesh using nutritional and psychosocial intervention: A Pilot study

Home observed for measurement of environment

ASSESSMENT INFORMATION TO BE DATA ENTERED

1	Visit month	|__||__|	
2	Date of assessment<br>(If not assessed, enter 09/09/99)	|__||__| / |__||__| / |__||__| D D M M Y Y	
	(O) = Observed; (E)= Environment; (I)=Interview		
3	Relation to the child (শিশুর সাথে উত্তরদাতার সম্পর্ক)	1=Mother /মা; 2=Father /বাবা; 3=other/অন্যান্য	|___|
4	Test completed (টেস্ট সম্পন্ন)	1=Complete/ সম্পূর্ণ; 2=Refusal/প্রত্যাখ্যান; 3=Out-migrated/অন্য খানে চলে গেছে; 4= Incomplete/অসম্পূণ	|___|
	4a. Reason for refusal (*if response to Q4=2*)<br>প্রত্যাখ্যানের কারন (যদি Q4=2 হয়)	___________________	
	4b. Reason for incomplete form (*if response to Q4=4*) ফরম অসম্পূর্ণ রাখার কারন (যদি Q4=4 হয়)	___________________	
5. Emotional and Verbal Responsibility of Caregiver			
5a.	Caregiver spontaneously vocalizes to the child at least twice during the visit (exclude scolding). (O)<br>পরিদর্শন চলাকালীন সময়ে পরিচর্যাকারী বাচ্চার সাথে কমপক্ষে ২ বার স্বতর্ঃস্ফুত ভাবে কথা বলেন (বকা ছাড়া) ? (O)	1=Yes; 0=No; 9=NA	|___|
5b.	Caregiver responds to child's vocalizations with a verbal response. (O)<br>বাচ্চা মুখে কথা বললে / শব্দ করলে পরিচর্যাকারী মৌখিকভাবে সাড়া দেন।(O)	1=Yes; 0=No; 9=NA	|___|
5c.	Caregiver tells the child the name of some object or says the name of a person and object in a teaching style during the visit (O)<br>পরিদর্শন চলাকালীন সময়ে পরিচর্যাকারী বাচ্চাকে শেখানোর উদ্দেশ্যে কোন জিনিসের নাম বা ব্যক্তির নাম বলেছে। (O)	1=Yes; 0=No; 9=NA	|___|
5d.	Caregiver's speech is distinct, clear and audible. (O)<br>বাবা মার কথা স্পষ্ট, পরিষ্কার এবং সহজে শোনা যাচ্ছিল ।(O)	1=Yes; 0=No; 9=NA	|___|
5e.	Caregiver initiates verbal exchanges with the observer-ask questions, makes spontaneous comments. (O)<br>পরিচর্যাকারী নিজ থেকেই পরিদর্শনকারীর সাথে কথোপকথন শুরু করে, প্রশ্ন করছিল, স্বতঃস্ফুর্ত মতামত দিচ্ছিল? (O)	1=Yes; 0=No; 9=NA	|___|
5f.	Caregiver express ideas freely and easily, and uses statements of appropriate length for conversation (i.e., gives more than brief answers) (O)<br>পরিচর্যাকারী কোন সংকোচ ছাড়াই সহজভাবে কথাবার্তা বলছিল এবং কথোপকথোন চালিয়ে যাওয়ার জন্য সঠিকভাবে মতামত দিচ্ছিল (সংক্ষিপ্ত উত্তরের চেয়ে বেশী কথা বলছিল)। (O)	1=Yes; 0=No; 9=NA	|___|

5g.	Caregiver spontaneously praises child's qualities or behavior twice during visit. (O)<br>পরিদর্শন চলাকালীন সময়ে পরিচর্যাকারী স্বতঃস্ফূর্তভাবে বাচ্চার গুনাবলী বা আচরণ নিয়ে কমপক্ষে দুই বার প্রশংসা করেছিল?	1=Yes; 0=No; 9=NA	|___|
5h.	When speaking of or to the child, caregiver's voice conveys positive feeling. (O)<br>পরিচর্যাকারীর কথায় বা বাচ্চার সাথে কথা বলার সময় ইতিবাচক অনুভূতি প্রকাশ পাচ্ছিল। (O)	1=Yes; 0=No; 9=NA	|___|
5i.	Caregiver does something affectionate with the child (hugs, caresses, kisses, pats, etc.) at least once during the visit. (O)<br>পরিদর্শনকালীন সময়ে পরিচর্যাকারী কমপক্ষে একবার বাচ্চাকে আদর করেছিল (চুমা দেওয়া, হাত বুলানো, জড়ায় ধরা বা হাল্কা চাপড়)। (O)	1=Yes; 0=No; 9=NA	|___|
5j.	Caregiver shows some positive emotional response or praise to the child offered by the observer. (O)<br>পরিদর্শন চলাকালীন সময়ে পরিদর্শনকারী বাচ্চার কোন প্রশংসা করলে পরিচর্যাকরী খুশীর ভাব বা ইতিবাচক প্রতিক্রিয়া প্রকাশ করেছিল। (O)	1=Yes; 0=No; 9=NA	|___|
5k.	Caregiver smiles at the child or laughs with the child. (O)<br>পরিদর্শনকালীন সময়ে পরিচর্যাকারী বাচ্চার সাথে হেসেছিল বা বাচ্চার দিকে তাকিয়ে হেসেছিল। (O)	1=Yes; 0=No; 9=NA	|___|
6. Avoidance of Restriction and Punishment			
6a.	Caregiver does not shout at the child during the visit. (O)<br>পরিচর্যাকারী বাচ্চার সাথে কোন চিৎকার করে নাই? (O)	1=Yes; 0=No; 9=NA	|___|
6b.	Caregiver does not express over annoyance or hostility towards the child. (O)<br>পরিচর্যাকারী বাচ্চার প্রতি খুব বেশী বিরক্তি বা কোন আক্রমনাত্বক আচরন দেখায় নাই। (O)	1=Yes; 0=No; 9=NA	|___|
6c.	Caregiver does not slap or spank the child during the visit. (O)<br>পরিদর্শন চলাকালীন সময়ে পরিচর্যাকারী শিশুকে চড়/ পাছায় থাপ্পর কোনটাই দেয় নাই। (O)	1=Yes; 0=No; 9=NA	|___|
6d.	Caregiver reports no instances of physical punishment during the past week. (I)<br>আপনি (পরিচর্যাকারী) কি গত সপ্তাহে বাচ্চাকে কোনও শারীরিক শাস্তি (আস্তে বা হাল্কা) দেন নাই? (I)	1=Yes; 0=No; 9=NA	|___|
6e.	Caregiver does not scold or criticize the child during the visit. (O)<br>পরিদর্শন চলাকালীন সময়ে পরিচর্যাকারী শিশুকে কোন বকা দেয় নাই / শিশুর সমালোচনা করে নাই। (O)	1=Yes; 0=No; 9=NA	|___|
7. Caregiver Promotes Child Development			
7a.	Caregiver tends to keep the child within visual range and looks at the child quite often. (O)<br>পরিদর্শন চলাকালীন সময়ে পরিচর্যাকারী বাচ্চার প্রতি খেয়াল রেখেছিলো এবং তার দিকে বার বার তাকাচ্ছিল? (O)	1=Yes; 0=No; 9=NA	|___|
7b.	Caregiver talks to the child while doing her housework. (E)<br>আপনি কি (পরিচর্যাকারী) ঘরের কাজ করার সময় বাচ্চার (নাম) সাথে কথা বলেন ? (E)	1=Yes; 0=No; 9=NA	|___|
7c.	Caregiver consciously encourages developmental advance. (E)<br>আপনি কি (পরিচর্যাকারী) সচেতনভাবে বা বুঝেশুনে বাচ্চাকে কিছু শেখানোর (সঠিক বিকাশের) জন্য উৎসাহ দেন ? (E)	1=Yes; 0=No; 9=NA	|___|

7d.	Caregiver structures the child's day. (I)<br>আপনি (পরিচর্যাকারী) কি বাচ্চার দৈনিক কাজগুলো নির্দিষ্ট সময় অনুযায়ী করেন ? (I)	1=Yes; 0=No; 9=NA	|___|
7e.	Caregiver believes the child's behavior can be changed or modified and is influenced by the parent's behavior. (I)<br>আপনি (পরিচর্যাকারী) কি মনে করেন বাচ্চার আচরন পরিবর্তন বা উন্নত করা যায় এবং বাবা-মার আচরন দ্বারা বাচ্চার আচরন প্রভাবিত হয়? (I)	1=Yes; 0=No; 9=NA	|___|
8. Organization of Physical and Temporal Environment			
8a.	When the primary caregiver is away, care is provided by one of the three regular substitutes. (I)<br>আপনি (পরিচর্যাকারী) যখন বাচ্চাকে রেখে বাড়ীর বাইরে যান তখন কি কমপক্ষে তিনজন বিকল্প পরিচর্যাকারীর মধ্যে একজন বাচ্চাকে দেখাশুনা করে? (I)	1=Yes; 0=No; 9=NA	|___|
8b.	Child is not cared for by another child (under 12 years of age). (I)<br>এই বাচ্চাকে (নাম) অন্য কোন বাচ্চা দেখাশুনা করে না (১২ বছরের নীচে)? (I)	1=Yes; 0=No; 9=NA	|___|
8c.	There is no evidence that older children or adults handle the child inappropriately. (E)<br>বড় বাচ্চা বা বাড়ীর অন্য কেউ কি বাচ্চাকেঅস্বাভাবিকভাবে(এলোমেলোভাবে) ধরে না বা নাড়াচাড়া করে না? (E)	1=Yes; 0=No; 9=NA	|___|
8d.	Someone takes the child to a shop or market at least once a week. (I)<br>কেউ কি বাচ্চাকে সপ্তাহে কমপক্ষে ১ বার দোকানে বা বাজারে সাথে করে নিয়ে যায়? (I)	1=Yes; 0=No; 9=NA	|___|
8e.	The child is taken to the health clinic regularly (to be weighted or to get immunizations). (I)<br>আপনি কি নিয়মিতভাবে বাচ্চাকে স্বাস্থ্যকেন্দ্রে নিয়ে যান (টীকা দিতে বা ওজন নিতে)? (I)	1=Yes; 0=No; 9=NA	|___|
8f.	The child has a special place to keep his toys and "treasures". (E)<br>বাচ্চার খেলনা এবং জিনিসপত্র রাখার জন্য নির্দিষ্ট কোন জায়গা আছে কি?(E)	1=Yes; 0=No; 9=NA	|___|
8g.	The child's play area is relatively safe and free for hazards. (O)<br>বাচ্চার খেলার পরিবেশ অপেক্ষাকৃত নিরাপদ এবং ঝামেলামুক্ত? (O)	1=Yes; 0=No; 9=NA	|___|
8h.	The stove is located in a relatively safe area. (O)<br>চুলা অপেক্ষাকৃত নিরাপদ স্থানে (উচু) আছে? (O)	1=Yes; 0=No; 9=NA	|___|
8i.	The house is relatively light. (O)<br>ঘরে পর্যাপ্ত আলো আছে? (O)	1=Yes; 0=No; 9=NA	|___|
8j.	The house is relatively ventilated. (O)<br>ঘরে পর্যাপ্ত বাতাস চলাচল করে? (O)	1=Yes; 0=No; 9=NA	|___|
8k.	The house is relatively clean. (O)<br>বাড়ীঘর অপেক্ষাকৃত পরিষ্কার? (O)	1=Yes; 0=No; 9=NA	|___|
8l.	The house is relatively neat and orderly. (O)<br>বাড়ীঘর অপেক্ষাকৃত গোছানো? (O)	1=Yes; 0=No; 9=NA	|___|
9. Provision of Appropriate Play Material			
9a.	There are some toys, tins, balls, dolls, slates, or materials in the house that are appropriate play materials for the child. (E)<br>বাড়ীতে কিছু খেলনা, টিন,বল, পুতুল, স্লেট, বা জিনিসপত্র আছে যা বাচ্চার উপযোগি এবং যা দিয়ে বাচ্চা খেলতে পারে? (E)	1=Yes; 0=No; 9=NA	|___|
9b.	The child has a riding toys or some toy that provides gross motor stimulation. (E)	1=Yes; 0=No; 9=NA	|___|

	বাচ্চার কোন চড়ার খেলনা আছে বা এমন কোন খেলনা আছে যা দিয়ে স্থুল পেশী সঞ্চালন হয়? (E)		
9c.	The caregiver provides toys or interesting activities for the child during the visit. (O)<br>পরিদর্শন চলাকালীন সময়ে পরিচর্যাকারী কি বাচ্চাকে কোন মজার কাজ করতে দিয়েছে বা কোন খেলনা দিয়েছে? (O)	1=Yes; 0=No; 9=NA	|___|
	10. Opportunities of Variety of Daily Stimulation		
10a.	There are some magazines, newspapers, or books visible in the house. (E)<br>বাড়ীতে চোখে পড়ে এমন কোন ম্যাগজিন, বই বা পত্রিকা আছে? (E)	1=Yes; 0=No; 9=NA	|___|
10b.	The family has a pet (The pet which does not belong to the family, is given food regularly). (E)<br>বাড়ীতে কি কোন পোষা প্রানী আছে?(প্রতিবেশির প্রানীকে যদি একদিন পরপর খাবার দেয়) ? (E)	1=Yes; 0=No; 9=NA	|___|
10c.	The father (or a father figure) plays with the children or provides some caregiving every day. (E)<br>বাচ্চার বাবা (অথবা বাবার মত) যখন বাড়ীতে থাকে তখন কি প্রতিদিনই বাচ্চার সাথে খেলে বা যত্ন নেয়? (E)	1=Yes; 0=No; 9=NA	|___|
10d.	The caregiver tells the child stories or nursery rhythms at least once a week. (I)<br>আপনি (বা পরিচর্যাকারী) কি বাচ্চাকে (নাম) সপ্তাহে কমপক্ষে একবার গল্প বা ছড়া বলে শোনান? (I)	1=Yes; 0=No; 9=NA	|___|
10e.	The caregiver sings to the child every day. (I)<br>আপনি (বা পরিচর্যাকারী) কি প্রতিদিন বাচ্চাকে গান শোনান? (I)	1=Yes; 0=No; 9=NA	|___|
10f.	The child eats at least one meal per day with the caregiver and father (or a father figure) on days that the father (or father figure) is present in the home. (I)<br>যে দিন গুলোতে বাবা (অথবা বাবার মত) বাসায় থাকে তখন বাচ্চা, আপনি (বা পরিচর্যাকারী) এবং বাবা প্রতিদিন কমপক্ষে একবেলা একসাথে বসে খায় কিনা ;? (I)	1=Yes; 0=No; 9=NA	|___|
10g.	The family has not moved more than once in the past year. (I)<br>গত বছরে একবারের বেশি বাসা পরিবর্তন করেননি? (I)	1=Yes; 0=No; 9=NA	|___|
10h.	The family visits or receives visits from relatives at least once per month. (I)<br>বাচ্চাকে সাথে নিয়ে কোন আত্নীয়ের বাড়ীতে মাসে একবার বেড়াতে গিয়েছেন বা কোন আত্নীয় আপনার বাসায় বেড়াতে এসেছে? (I)	1=Yes; 0=No; 9=NA	|___|
10i.	The family visits or receives visits from close friends at least once per month. (I)<br>বাচ্চাকে সাথে নিয়ে কোন বন্ধু-বান্ধবেরর বাড়ীতে মাসে একবার বেড়াতে গিয়েছেন বা কোন বন্ধু-বান্ধব আপনার বাসায় বেড়াতে এসেছে? (I)	1=Yes; 0=No; 9=NA	|___|
11. Cleanliness of Child			
11a.	The child is relatively clean, with no offensive odor. (O)<br>বাচ্চা অপেক্ষাকৃত পরিস্কার-পরিচ্ছন্ন এবং গায়ে কোন বাজে গন্ধ নাই? (O)	1=Yes; 0=No; 9=NA	|___|
11b.	The child's hair is relatively clean. (O)<br>বাচ্চার চুল অপেক্ষাকৃত পরিস্কার ? (O)	1=Yes; 0=No; 9=NA	|___|
11c.	The child's clothes are relatively clean. (O)<br>বাচ্চার পরনের কাপড় অপেক্ষাকৃত পরিস্কার পরিচ্ছন্ন? (O)	1=Yes; 0=No; 9=NA	|___|
	Interviewer Name and Code	__________________________________	|___||___||___|

**Multidimensional Evaluation of the emergence of executive function and dysfunction in young children in Bangladesh: Pilot Study**<br>Standard Operating Procedure:<br><u>Buccal scrape Collection</u>	Version No : 01<br>PR-21084

**Date:**<br>**Written By: Dr.Talat Shama**		**Effective Date:** 21 September 2021
**Related Documents and Attachments: None**		

Background:

Buccal swabs collect the cells on the inside of the mouth. The DNA inside these cells will be used for analysis.

Purpose :

The purpose of this Standard Operating Procedure (SOP) is to standardise the process for buccal swab sample collection from participants. Buccal swabs are collected from mother and children whose parents/guardians have agreed and consented to their participation in the study.

Scope :

This Standard Operating Procedure (SOP) describes how buccal swabs should be collected, processed and stored from children.

List of abbreviations/ Definitions :

Term/Acronym	Description
Buccal Swab	A buccal swab is an easy, non-invasive way of collecting cheek cells from subjects for DNA analysis.
Buccal mucosa	The inside of the cheek
Isohelix Swab	A swab used for epigenetic measurements of potential biomarkers.
Barcoded Cryovials	Small vials in which the sample will be stored.
Aliquot	When the barcoded cryovials are filled with sample, they are referred to as an aliquot.

Roles & Responsibilities

Role	Responsibilities
Field Assistants	- Safe handling and collection of buccal swabs according to this SOP<br>- Transport of samples to Laboratory<br>- Storage of buccal samples

Materials & Equipment

Materials	Specifics	Supplier
Isohelix Swab Collection Kit	Buccal swab with 2ml tube and release cap. Matrix swab head, ethylene oxide treated and individually wrapped	Isohelix MS-01
BuccalFix tube	Sarstedt 2ml screw cap tubes pre-filled with 0.5ml BuccalFix stabilisation buffer	BFX/S1/05/50
Gloves		
Patient ID label		

Procedure

The research and field assistants should be trained in the procedure of buccal swab collection as per following steps:

1. Confirm the participant's name and date of birth.

2. Explain the buccal swab collection procedure to the mother. Be clear that this will not cause the child any pain.

3. Best results are obtained if the child has not eaten, drunk or cleaned their teeth within the hour immediately before sample collection. If the infant is nursing, wait 30 mins after feeding before collecting a buccal swab.

4. Buccal swabs should be collected during visits at enrolment, at 24 months of age, and at 36 months of age during visits to the clinic for Neuropsychological assessments with a window of +7 days.

5. Put on clean examination gloves - these should be worn at all times when handling the swabs, storage tubes and performing the swabbing procedure.

6. Check the packaging has not been damaged. Check the expiry date. Do not use if the package is damaged or if the collection kit has expired.

7. Remove a sterile swab from the packaging with gloved hands, opening at one end. Remove the swab from the tube by gently easing the cap hub. Take care not to touch the white swab head with your fingers. Do not placing the white swab head on any surfaces to avoid contamination.

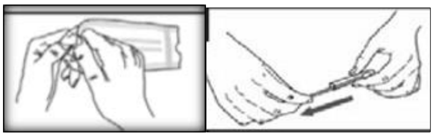

Ask the parent to sit the child on their lap and keep their mouth open. Insert the swab into the *right* side of the infant's mouth.

Use a stopwatch or the second hand of a watch/clock to time the collection.

Insert the swab into the gutter between the inside of the cheek and outer side of the lower gum.

Rub forwards and backwards for 30 seconds, pressing firmly while applying gentle pressure on the outside of the cheek with the other hand.

This will ensure maximal contact of the entire swab surface with the mucosa so that sufficient cells are collected.

This sampling technique should collect buccal cells without drawing blood.

8. After 30 seconds, allow the participant to become comfortable then re-insert the swab on the *same (right)* side of the participant's mouth but this time using the other surface of swab head to make contact with the buccal mucosa. Repeat the steps above as stated in point 7.

9. After collection of the cheek cells allow the swab to air dry for 30 seconds, then place the swab into the BuccalFix collection tube. Hold the release cap whilst pulling the swab handle outwards to release the swab material into the tube. Take care not to touch the swab head.

10. Allow the participant to become comfortable before proceeding with the next swab. Repeat step 5 to 9 for collection of a 2nd swab on the *left* side of the mouth.

11. Insert the second swab into the same BuccalFix collection tube as the first swab. In total there would be 2 swabs in the collection vial.

12. Label the BuccalFix collection tube with the participant's 11 digit ID number.

13. Remove gloves and decontaminate hands (Wash and dry hands OR use a gel hand sanitiser).

14. Transport samples to the laboratory at in a cooler box .

15. Place samples for storage in the -80°C freezer as soon as possible. Record storage in clinical trials management software.

<table>
<tr><td>Multidimensional Evaluation of the emergence of executive function and dysfunction in young children in Bangladesh: Pilot Study
Standard Operating Procedure:
Blood Collection, Processing and Storage</td><td>Version No : 01
PR-21084</td></tr>
</table>

Written By: Dr.Talat Shama and Md. Mamun Kabir		Effective Date: 21 September 2021
Related Documents and Attachments: None		

Background:

Establishing a biobank of high-quality samples is critical for the M4EFaD studies objectives and on-going studies. Collection and storage of biological samples is central to the quality of the biobank.

Purpose:

The purpose of this Standard Operating Procedure (SOP) is to standardize the process by which blood samples from M4EFaD participants are processed and stored.

Child blood collection-Child blood will be collected at three time points of the study period (at enrollment, 24th and 36th month) and one additional sample for MAM child at the time point of anthropometric recovery i.e when WLZ/WHZ >-1 SD will be achieved.

Mother blood collection-The mother will have one blood draw performed within 2 weeks of enrollment.

Scope / Applicability:

The scope of this Standard Operating Procedure covers all blood samples collected from M4EFaD participants in Bangladesh.

Roles / Responsibilities: Protocol specific

Specimen:

Blood received in the field site or home visits in EDTA containing vacutainer tube.

Materials:

1. Alcohol
2. EDTA containing vacutainer tube for plasma
3. Cotton wool/gauze
4. Syringes and needles/ butterfly needle
5. Sterile gloves
6. Tourniquet
7. Pipette
8. Tips
9. Refrigerated centrifuge
10. -70°C freezer
11. Screw cap tubes
12. Cool box with ice

Safety/ Risk Assessment:

Infection can be transmitted from patient to staff and from staff to patient during the blood-taking procedure. Viral agents pose the greatest hazard and in some instances are potentially lethal. Of particular importance are the COVID-19 viruses. To decrease the risk of transmission of these viral agents, the following recommendations should be practiced:

- ✓ Wear latex or vinyl gloves impermeable to liquids
- ✓ Wear Personal Protective Equipment at all times when processing blood collection
- ✓ Change gloves between patients.
- ✓ Syringes and needles should be disposed of in a puncture-resistant, autoclavable container. No attempt should be made to recap the needle. A new syringe and needle must be used for each patient.
- ✓ Wipe the surface of the collection desk and the gloves with a disinfectant.
- ✓ Label the EDTA containing vacutainer.
- ✓ For the transport of the sample to the laboratory, place the tube in a container that can be securely sealed.
- ✓ Remove gloves and discard in an autoclavable container.
- ✓ Wash hands with soap and water immediately after removing gloves.
- ✓ Waste disposal: Discard all sharps in sharp boxes. Autoclave all other clinical wastes before taking them for incineration

Venipuncture:

1. Gather everything needed to complete the blood collection process: Gloves, syringe, needle, tourniquet, gauze squares, cotton balls, adhesive bandage, puncture resistant container, EDTA containing vacutainer tube and antiseptic (70% alcohol).
2. Ensure vacutainers are labelled with the participant’s 11 digit ID number
3. The size of the needle will depend on the collection site and the size of the vein. A 23-gauge needle that is 20 – 25 mm in length or a butterfly needle is generally used for children. Collecting a large amount of blood from a child can be difficult: 2 – 3 ml is usually sufficient. For mother blood collection 21g needle is used and draw 5ml blood.
4. Select an arm and apply a tourniquet to restrict the flow of venous blood. The most prominent vein is usually chosen for venipuncture.
5. Vigorously wipe the skin with the 70% alcohol, and swab. Rub over the selected area. Allow to dry. If the vein is palpated again, repeat the skin disinfection.
6. After the disinfectant has dried, insert the needle into the vein with the bevel of the needle face-up. Once the vein is entered, withdraw the blood by pulling back the barrel of the syringe in a slow, steady manner. Air must not be pumped into a vein. After the desired amount of blood is obtained, release the tourniquet and place a sterile cotton ball over the insertion site while holding the needle in place. Withdraw the needle and have the patient hold the cotton ball firmly in place until the wound has stopped bleeding. Put the adhesive bandage on the wound.
7. Transfer venous blood from the syringe to the EDTA containing vacutainer and let the tube fill to the appropriate level.
8. After having drawn the specified amount of blood the phlebotomist will assure completion of all associated documentation, including the lab processing form, and the blood specimen transport log. The specimen should then be stored in a cool box or refrigerator until transport to the parasitology lab of icddr,b.

Transportation:

1. Specimens will be sent from Mirpur clinic on the day of collection via study field assistant to the icddr,b parasitology lab within 4-6 hours after blood collection.

2. Upon transport of specimens to icddr,b the Field Research Assistant will complete the Sending / transport Log, noting the specimen ID, date of collection and transport. Staff from icddr,b parasitology lab will also document receipt of sample on the Specimen Receipt Log.
3. Check they have been labelled correctly and enter the time of arrival into specimen log on the MedSciNet database.
4. All processing of biospecimens is to be performed in the class II biosafety cabinet.
5. As much as possible, keep specimens out of the light

Centrifugation and allocation:

1. EDTA blood specimens will be centrifuged at 1600 G for 10 minutes at **4˚C**.
2. After centrifugation transfer plasma into screw cap tubes labeled with 11 digit ID number, Date and participant details.
3. Aliquot plasma sample as per flow chart:

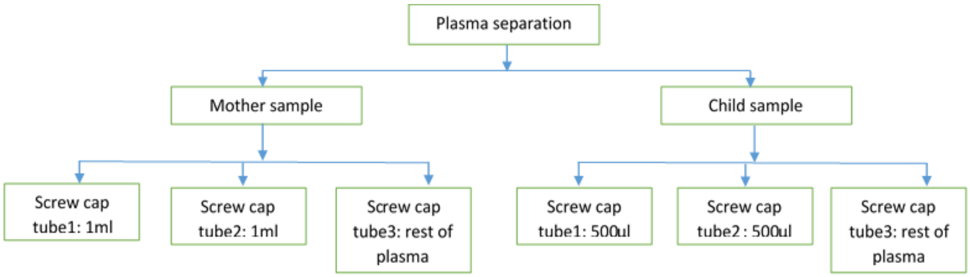

4. Following removal of the EDTA plasma, transfer the white cell layer/buffy coat (interface between plasma and red blood cells) from the EDTA vacutainer with a sterile pipette 0.250ml into a screw cap tube.

Storage:

1. Check that all samples have been processed for the participant
2. Transfer the tubes as quickly as possible to the -70°C freezer immediately.
3. Also the cell pellet will be stored and kept in freezer as well after centrifugation .
4. Ensure that the participant’s sample storage locations are correctly recorded in the database.

I. Purpose

To offer guidance to study staff regarding assessment and documentation of adverse events and serious adverse events.

II. Materials

1. Adverse event (AE) CRF
2. Serious Adverse Event (SAE) CRF from ICDDRB

III. Definitions

1) Adverse event (AE): An adverse event can be any unfavorable and unintended sign, symptom, or disease temporarily associated with the use of a therapeutic good, without any judgment about causality or relationship to the therapeutic product.

2) Serious adverse event (SAE): Any untoward medical occurrence that at any dose results in -

- Death
- Life threatening event
- Hospitalization
- Disability or permanent damage
- Required intervention to prevent permanent impairment or damage
- Other (deemed serious by study staff or patient's physician)

IV. Methods

1) It is the responsibility of all study staffs who come into contact with study subjects to be on the look out for adverse events and notify a medical officer or their supervisor when they occur.

2) FRAs will ask about potential adverse events during daily home visits for surveillance. Medical officers will assess for AE's during each encounter during clinic visits if the child seeks for primary care.

3) If a potential AE is suspected and is temporarily related to the intervention (or control) then an adverse event CRF should be completed. If a potential SAE occurs, then the ICDDR,B SAE form should be completed and the RRC notified within 24 hours. Both the site PI and the project PI must be notified immediately.

4) If either an AE or SAE occurs, the child should be brought to the study clinic for a full medical evaluation by medical officer. If the event was thought to be due to participation in the study, the child will be discontinued from the study. The site PI and project PI should also be notified.

Multidimensional Evaluation of the emergence of executive function and dysfunction in young children in Bangladesh: Pilot Study<br>Standard Operating Procedure:<br>**E-RUSF Feeding Procedure**	Version No : 01<br>PR-21084

Written by: Dr. Talat Shama		Effective Date: 21 September 2021
Related Documents and Attachments: None		

Background : A total 140 malnourished children aged 11 to 13 months with the criteria of WLZ/WHZ <-2 and ≥-3 z-score and/or MUAC <12.5 and ≥11.5 cm (moderate acute malnutrition) from Mirpur study area will be enrolled and randomized in 1:1 ratio to receive complementary foods either RUSF 500 kcal/d daily followed by SQLNS or E RUSF 500 kcal/d daily followed by E-SQLNS till the end of follow-up for 2 years.

Table 1: A typical recipe for Enhanced Ready to Use Supplementary Food (92g serving)

	**E_RUSF** (amount per 92g)
Total energy (kcal)	500
Protein kcal (% of total energy)	10.3
Proteins (g)	12.8
Dairy proteins (% of total proteins)	50
PDCAAS (Protein Digestibility Corrected Amino Acid Score)	1
Lipid kcal (% of total energy)	55
Lipids (g)	30.3
ALA[a] energy (% of total energy)	1.7
ALA[a] (g)	0.94
LA[b] energy (% of total energy)	6.3
LA[b] (g)	3.40
Ratio LA/ALA	3.6
DHA (mg)	80
EPA (mg)	135
Total fibers (g)	5.5
Prebiotics (combined Inulin & FOS)	6
Calcium (mg)	302
Phosphorus (mg)	343
Potassium (mg)	1171
Magnesium (mg)	80
Zinc (mg)	11.8
Copper (mg)	1.5
Iron (mg)	10.3
Iodine (µg)	98
Selenium (µg)	28
Sodium (mg)	165
Vitamin A / Retinol (µg)	790
Lutein (mg)	0.944

Zeaxanthin (mg)	0.044- 0.1
Beta – Cryptoxanthin (mg)	1-2
Vitamin D (μg)	14
Vitamin E (mg)	18.4
Vitamin C (mg)	46
Vitamin B1 (mg)	0.50
Vitamin B2 (mg)	1.5
Vitamin B6 (mg)	0.55
Vitamin B12 (μg)	1.5
Vitamin K (μg)	14.4
Biotin (μg)	56
Folic acid (μg)	184
Pantothenic acid (mg)	2.8
Niacin (mg)	4.6

[a]Alpha- Linolenic Acid; [b]Linoleic Acid; [c]CMAM Statement, UN agencies, 2007: CMAM (Community-based Management of Acute Malnutrition), a Joint Statement by the World Health Organization, the World Food Programme, the United Nations System Standing Committee on Nutrition and the United Nations Children's Fund, 2007

70 MAM children will receive daily 92gm weighed E-RUSF packet (500 Kcal total energy) until anthropometric recovery (WLZ > - 1SD) is achieved or for maximum 3 months then E-SQLNS will be given till the end of 2 years follow-up.

Purpose :

To describe the procedure for feeding E-RUSF nutritional supplement for MAM children in the study. Also for standardization of feeding procedure, storage and quality control.

Materials :

1. E-RUSF packet
2. Fish oil capsule
3. Scissors and safety pin
4. Spoon
5. Checklist for daily feeding record / Compliance forms

Methods :

Mother will learn from our study staff how to feed this complementary food to her child properly. Mother will receive 7 days feed/supplement for her child at a time. Initially our study staff will ensure daily feeding by visiting the household daily, interview and counsel the mother, fill up the compliance forms and collect the empty sachets and empty shells of the capsules daily for first 1 month. Then our study staff

will visit the household twice per week to ensure the feeding by interviewing mother and collecting the empty packets of intervention. Study staff will capture the data on case record form (CRF) and also document any event associated with the intervention. Study staff will also measure weight, length (until 24 months of child's age) /height (when measuring > 24 months old child) and MUAC weekly to track recovery from wasting. Our study staff will ensure the availability of feeds every week before finishing existing packets. This process will continue till end of the study.

Feeding Procedure :

The feed will come in a paste form in a sterile packet/ sachet which is a ready to eat formulation. Mother will need to cut the sterile packet with scissors, open the seal and pour/spoon the paste in a spoon and feed the child in a small amount at intervals. With these sachets a gel capsule will also be supplied one for each day which will contain DHA and EPA oil. These gel capsules will be pricked by the mother or the care giver and the contents will be squirted in to the child's mouth. The capsules will be pricked with the help of the sharp end of a safety pin after cleaning the safety pin properly. Start with capsule following 3 to 5 gm ERUSF to improve the taste of the child and also to mask the fishy taste. Then, wait for 30 minutes and after that, start feeding the remaining of ERUSF to the child. During the feeding time, the mothers will be asked to spoon feed their children the assigned diet until the child refuses to eat. After a two-minute pause, the same food will be offered a second time until s/he refuses again. After a second two-minute pause, the food will be offered a third time until refused again. After this third refusal, the feeding episode will be kept withheld. Children are considered as refusing intake if they move their head away from the food, cry, clamp the mouth shut or clench the teeth, or become agitated, spits out the food or refuses to swallow. Then mother can take a break and feed the child any time for rest of the day as the food can be used for day long but cannot be fed after 24 hours. Next day, a new sachet will be opened by mother and mother will keep the previous empty or the leftover packet in a separate box. Mother will also be strictly instructed to refrain from sharing the feed with other siblings in the family. Our Surveillance workers will later collect the sachets to keep records and fill up the checklist every week including history of morbidities associated with consumption of the feed in their surveillance forms.

Storage :

The E-RUSF sachets and the oil capsules should be kept in open air at room temperature. Highest shelf life of one packet of E-RUTF at room temperature is six months. Mother will be strongly instructed to refrain from putting the sachets and the capsules in the refrigerator.

Quality control :

Surveillance workers will supply 7 days' feed in one visit and at the same time collect empty or opened sachets and also the empty shells of the capsules from previous 7-day visits and ask mother questions regarding feeding the child regularly and fill up compliance and surveillance forms twice in a week . In the forms they will collect following data :

1. Morbidities associated with feeding of the child : event of diarrhoea (duration, frequency, severity), vomiting (duration, frequency, severity), fever (temperature will be recorded), rash or any other adverse events.
2. Number of empty sachets – whether child has been fed full packet, partial packet or not fed at all in a day
3. Reason for failure of feeding : Child refused to feed, parents refused to feed, child or family was not at home, vomiting, child was sick, other reason.

Multidimensional Evaluation of the emergence of executive function and dysfunction in young children in Bangladesh: Pilot Study<br>Standard Operating Procedure:<br>**E- SQLNS Feeding Procedure**	Version No : 01<br>PR-21084

Written by: Dr. Talat Shama	Approval Signature:	Effective Date: 21 September 2021
Related Documents and Attachments: None		

Background : A total 140 malnourished children aged 11-13 months with the criteria of WLZ <-2 and ≥-3 z-score and/or MUAC <12.5 and ≥11.5 cm (moderate acute malnutrition) from Mirpur study area will be enrolled and randomized in 1:1 to receive complementary foods either RUSF 500kcal/d daily followed by SQLNS or E RUSF 500 kcal/d daily followed by E-SQLNS till the end of follow-up for 2 years.

Table : Mean nutritional value for 1 packet E-SQLNS (26gm)

	eSQ-LNS (amount per 26g)
Total energy (kcal)	130
Proteins (g)	2,6
Lipids (g)	9,6
ALA[a] (g)	0,58
LA[b] (g)	2,81
DHA (mg)	80
EPA (mg)	135
Prebiotics (combined Inulin & FOS)	6
Calcium (mg)	280
Phosphorus (mg)	190
Potassium (mg)	200
Magnesium (mg)	40
Zinc (mg)	8
Copper (mg)	0,34
Iron (mg)	6
Iodine (µg)	90
Manganese (mg)	1,2
Selenium (µg)	20
Vitamin A / Retinol (µg)	400
Lutein (mg)	0,944
Zeaxanthin (mg)	0,044- 0,1
Beta – Cryptoxanthin (mg)	1-2
Vitamin D (µg)	10
Vitamin E (mg)	6
Vitamin C (mg)	30
Vitamin B1 (mg)	0,3
Vitamin B2 (mg)	0,4
Vitamin B6 (mg)	0,3
Vitamin B12 (µg)	0,5
Vitamin K (µg)	30
Folic acid (µg)	80
Pantothenic acid (mg)	1,8
Niacin (mg)	4

[a]Alpha- Linolenic Acid; [b]Linoleic Acid, Ref: Arimond et al, 2013, Wilson et al. Prebiotic inulin-type fructans and galactooligosaccharides: definition, specificity, function, and application in gastrointestinal disorders. J Gastroenterol Hepatol. Healey G, Murphy R, Butts C, Brough L, Whelan K, Coad J. Habitual dietary fibre intake influences gut microbiota response to an inulintype fructan prebiotic: a randomised, doubleblind, placebo-controlled, crossover, human intervention study. Br J Nutr, Bohm et al. Nutrition Reviews,2020.

70 MAM children will receive daily 1 packet 92gm weighed produced E-RUSF packets (500 Kcal total energy) until anthropometric recovery (WLZ > - 1SD) is achieved or for maximum 3 months then E-SQLNS 1 packet (26gm) daily will be given till the end of 2 years follow-up.

Purpose :

To describe the procedure for feeding E-SQLNS nutritional supplement for MAM children (intervention group) in the study. Also, for standardization of feeding procedure, storage and quality control.

Materials :

1. E-SQLNS packet
2. Scissors
3. Spoon
4. Complementary food (to be prepared by mother/ care giver at home prior to mixing with E-SQLNS)
5. Bowl
6. Checklist for daily feeding record / Compliance forms

Methods :

Mother will learn from our study staff how to feed this complementary food to her child properly. Mother will receive 7 days feed/supplement for her child at a time. As by this time mother will get used to the feeding process for last 3 months, our study staff will visit the household twice per week and will ensure daily feeding by visiting the household , interview and counsel the mother, fill up the compliance forms and collect the empty sachets. Study staff will capture the data on e-CRF and also document any event associated with the intervention. Study staff will also measure weight, length (until 24 months of child's age) /height (when measuring > 24 months old child), and MUAC in every 3 months to track maintenance of anthropometric recovery. Our study staff will ensure the availability of feeds every week before finishing existing packets. This process will continue till end of the study.

Feeding Procedure :

The feed will come in a sterile packet/ sachet which is a ready to eat formulation. Mother will need to first wash her hands properly and then prepare the usual Complementary food or khichuri or other family food. Then mother will cut the sterile sachet, open the seal of E-SQLNS and with scissors mix the E-SQLNS (either half a sachet i.e 13 gm in the morning and half a sachet in the evening or full sachet i.e. 26 gm at the same time) with a portion of complementary food with a clean spoon in a clean way in a clean bowl . Then mother will feed the fortified complementary food in a small amount at intervals.

During the feeding time, the mothers will be asked to spoon feed their children the assigned diet until the child refuses to eat. After a two-minute pause, the same food will be offered a second time until s/he refuses again. After a second two-minute pause, the food will be offered a third time until refused again. After this third refusal, the feeding episode will be kept withheld. Children are considered as refusing intake if they move their head away from the food, cry, clamp the mouth shut or clench the teeth, or become agitated, spits out the food or refuses to swallow. Then mother can take a break and feed the child any time or she can divide the sachet and mix the other half at evening with the complementary food or other family food and feed the child again as the feed can be used or kept for rest of the day in room temperature but cannot be fed after 24 hours. Next day, a new sachet will be opened by mother and mother will keep the previous empty or the leftover packet in a separate box. Mother will also be strictly instructed to refrain from sharing the feed with other siblings in the family. Our Surveillance workers will later collect the sachets to keep records and fill up the checklist twice every week including history of morbidities associated with consumption of the feed in their surveillance forms.

Storage :

The E-SQLNS sachets should be kept in open air at room temperature <30^0 C. Maximum shelf life of one packet of E-SQLNS at room temperature is six months. Mother will be strongly instructed to refrain from putting the sachets in the refrigerator.

Quality control :

Surveillance workers will supply 7 days' feed in one visit and at the same time collect empty or opened sachets from previous 7-day visits and ask mother questions regarding feeding the child regularly and fill up compliance and surveillance forms twice in a week . In the forms they will collect following data :

1. Morbidities associated with feeding of the child : event of Diarrhoea (duration, frequency, severity), vomiting (duration, frequency, severity), fever (temperature will be recorded), rash or any other adverse events.
2. Number of empty sachets – whether child has been fed full packet, partial packet or not fed at all in a day

3. Reason for failure of feeding : Child refused to feed, parents refused to feed, child or family was not at home, vomiting, child was sick, other reason.

Multidimensional Evaluation of the emergence of executive function and dysfunction in young children in Bangladesh: Pilot Study<br>Standard Operating Procedure:<br>**SQLNS Feeding Procedure**	Version No :01<br>PR-21084

Written by: Dr. Talat Shama	Approval Signature:	Effective Date: 21 September 2021
Related Documents and Attachments: None		

Background : A total 140 malnourished children aged 11-13 months with the criteria of WLZ <-2 and ≥-3 z-score and/or MUAC <12.5 and ≥11.5 cm (moderate acute malnutrition) from Mirpur study area will be enrolled and randomized in 1:1 to receive complementary foods either RUSF ~100 kcal/kg/d daily followed by SQLNS or E RUTF ~100 kcal/kg/d daily followed by E-SQLNS till the end of follow-up for 2 years.

Table : Mean nutritional value for 1 packet SQLNS (20gm)

	**SQLNS** (amount per 20g)
Total energy (kcal)	118
Proteins (g)	2,6
Lipids (g)	9,6
ALA[a] (g)	0,58
LA[b] (g)	2,81
Calcium (mg)	280
Phosphorus (mg)	190
Potassium (mg)	200
Magnesium (mg)	40
Zinc (mg)	8
Copper (mg)	0,34
Iron (mg)	6
Iodine (µg)	90
Manganese (mg)	1,2
Selenium (µg)	20
Vitamin A / Retinol (µg)	400
Vitamin D (µg)	5
Vitamin E (mg)	6
Vitamin C (mg)	30
Vitamin B1 (mg)	0,3
Vitamin B2 (mg)	0,4
Vitamin B6 (mg)	0,3
Vitamin B12 (µg)	0,5
Vitamin K (µg)	30
Folic acid (µg)	80
Pantothenic acid (mg)	1,8
Niacin (mg)	4

[a]Alpha- Linolenic Acid; [b]Linoleic Acid, Ref: Arimond et al, 2013

70 MAM children will -receive daily 2 packets of 50gm weighed locally produced Rice-Lentil based RUSF packets (500 Kcal total energy) until anthropometric recovery (WLZ > - 1SD) is

achieved or for maximum 2 months then SQLNS 1 packet (20gm) daily will be given till the end of 2 years follow-up.

Purpose :

To describe the procedure for feeding SQLNS nutritional supplement for MAM children (intervention group) in the study. Also, for standardization of feeding procedure, storage and quality control.

Materials :

1. SQLNS packet
2. Scissors
3. Spoon
4. Complementary food (to be prepared by mother/ care giver at home prior to mixing with SQLNS)
5. Bowl
6. Checklist for daily feeding record / Compliance forms

Methods :

Mother will learn from our study staff how to feed this complementary food to her child properly. Mother will receive 7 days feed/supplement for her child at a time. As by this time mother will get used to the feeding process for last 3 months, our study staff will visit the household twice per week and will ensure daily feeding by visiting the household, interview and counsel the mother, fill up the compliance forms and collect the empty sachets. Study staff will capture the data on e-CRF and also document any event associated with the intervention. Study staff will also measure weight, length (until 24 months of child's age) /height (when measuring > 24 months old child) and MUAC in every 3 months to track maintenance of anthropometric recovery. Our study staff will ensure the availability of feeds every week before finishing existing packets. This process will continue till end of the study.

Feeding Procedure :

The feed will come in a sterile packet/ sachet which is a ready to eat formulation. Mother will need to first wash her hands properly and then prepare the usual complementary food or khichuri or other family food. Then mother will cut the sterile sachet, open the seal of SQLNS and with scissors mix the SQLNS (either half a sachet i.e 10 gm in the morning and half a sachet in the evening or full sachet i.e. 20 gm at the same time) with a portion of complementary food with a clean spoon in a clean way in a clean bowl . Then mother will feed the fortified complementary food in a small amount at intervals.

During the feeding time, the mothers will be asked to spoon feed their children the assigned diet until the child refuses to eat. After a two-minute pause, the same food will be offered a second time until s/he refuses again. After a second two-minute pause, the food will be offered a third

time until refused again. After this third refusal, the feeding episode will be kept withheld. Children are considered as refusing intake if they move their head away from the food, cry, clamp the mouth shut or clench the teeth, or become agitated, spits out the food or refuses to swallow. Then mother can take a break and feed the child any time or she can divide the sachet and mix the other half at evening with the complementary food or khichuri or other family food and feed the child again as the feed can be used or kept for rest of the day in room temperature but cannot be fed after 24 hours. Next day, a new sachet will be opened by mother and mother will keep the previous empty or the leftover packet in a separate box. Mother will also be strictly instructed to refrain from sharing the feed with other siblings in the family. Our Surveillance workers will later collect the sachets to keep records and fill up the checklist twice every week including history of morbidities associated with consumption of the feed in their surveillance forms.

Storage :

The SQLNS sachets should be kept in open air at room temperature <30^0 C. Maximum shelf life of one packet of SQLNS at room temperature is six months. Mother will be strongly instructed to refrain from putting the sachets in the refrigerator.

Quality control :

Surveillance workers will supply 7 days' feed in one visit and at the same time collect empty or opened sachets from previous 7-day visits and ask mother questions regarding feeding the child regularly and fill up compliance and surveillance forms twice in a week . In the forms they will collect following data :

1. Morbidities associated with feeding of the child : event of Diarrhoea (duration, frequency, severity), vomiting (duration, frequency, severity), fever (temperature will be recorded), rash or any other adverse events.
2. Number of empty sachets – whether child has been fed full packet, partial packet or not fed at all in a day
3. Reason for failure of feeding : Child refused to feed, parents refused to feed, child or family was not at home, vomiting, child was sick, other reason.

Multidimensional Evaluation of the emergence of executive function and dysfunction in young children in Bangladesh: Pilot Study<br>Standard Operating Procedure:<br>Stool Collection	Version No.01<br>PR-21084

Written By: Dr. Talat Shama		Effective Date: 21September 2021
Related Documents and Attachments: 2		

I. Purpose

To describe the procedure for stool specimen collection from participants in the study at protocol-specified intervals. Also for standardization of stool collection, storage and processing.

II. Materials

A. Pen
B. Stool collection equipments :
  I. Zymo feces Catcher
  II. Zymo DNA/RNA shield Fecal Collection tube (ZR1101)
  III. OMNImet.GUT ME-200
  IV. Disposable Gloves
  V. Cryo-Collection tubes
C. Masking tape
D. Small transfer cold boxes with cold packs within for specimen collection
E. Larger cold box for specimen storage
F. Child Specimen Requisitions Sheet source document
G. Stool Collection Case Report Form (CRF)
H. Stool Specimen Log
I. Specimen Sending Log and Specimen Receipt Log

III. Methods

A.The type of stool specimens to be collected from infants will be non diarrheal stool throughout in the study. Mother will be provided stool collection pot prior collection and should be reminded upcoming sample collection schedule by our staff.
i.Non diarrheal stool: Stools that are scheduled to collect yearly from study child without having diarrhea except for 1 year old MAM children an additional sample will be collected at the time point when anthropometric recovery i.e WLZ/WHZ >-1 SD will be achieved. Each time window period will be (+/- 7days).
B. Non diarrheal stool collection from mother: One mother stool sample (10 gm) will be collected within 2 weeks of enrollment.

C. Requirements for stool collection from children

(1) Ideally samples should be collected during the clinic visits, if necessary the sample can be collected during home visits during the window period of each time point specified by the protocol.

(2) Check and record whether the participant has taken antibiotics in the last 4 weeks or has had diarrhea in the last 3 days (defined as unusually loose bowel motion, more than twice as many times as usual in a day). Do not collect from individuals who have had diarrhea in the last 3 days. Proceed with collection if any recent diarrhea has ended 3 or more days ago.

D. <u>Research/Field assistant instructions for collecting stool sample in the clinic</u> :

1. Do not collect a sample if the participant has had Diarrhea in the last 3 days.
2. Disposable gloves are provided for you to use.
3. If you can, ask the participant or take help from their mother to empty their bladder, so that urine and stool are not mixed together.
4. Place the Zymo feces Catcher (on the floor or stool pot).
5. Ask the participant to pass stool on the feces catcher.
6. The stool should land in the catcher.
7. Collect the stool immediately after it is passed.
8. Use the scoop attached to the brown lid of the Zymo DNA/RNA shield Fecal Collection tube containers to place a heaped scoop of stool into the container. The tube has a liquid solution in it. Please don't tip this liquid out! Once the stool is in this container and the lid is on tightly, please shake this container with stool and liquid in it 10 times vigorously, so that the stool is well mixed with the liquid and no large stool pieces remain visible.

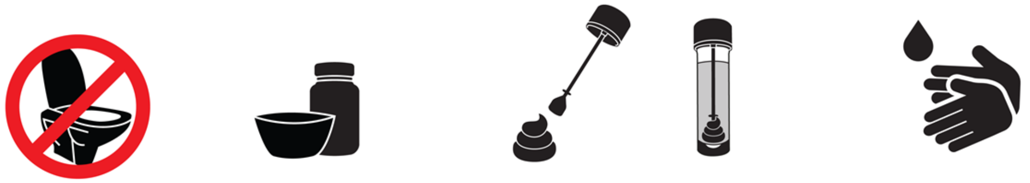

Don't let the sample go into the toilet — Collect stool into a clean container — Scoop a portion of the stool sample into the DNA/RNA Shield™ Fecal Collection Tube — Wash hands well

9. Place a scoop of stool, using the spoon provided, into the OMNImet.GUT ME-200 tube. Once the stool is in this container and the lid is on tightly, please shake this container with stool and liquid in it 10 times vigorously, so that the stool is well mixed with the liquid and no large stool pieces remain visible.

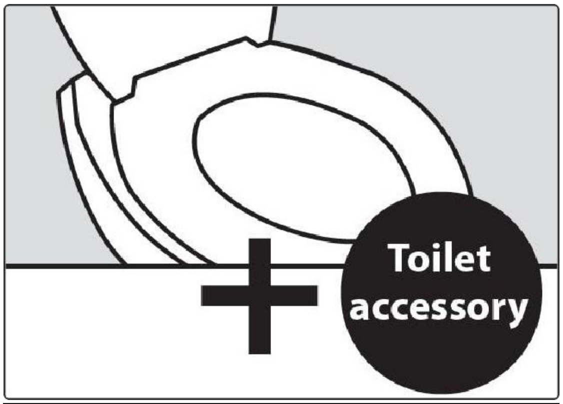

IMPORTANT PREPARATIONS

- Empty your bladder before beginning the collection.
- Collect fecal sample free of urine or toilet water.
- Utilize any toilet accessory which may be supplied with the collection device.
- Toilet paper or tissues may be required.

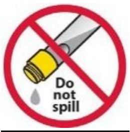

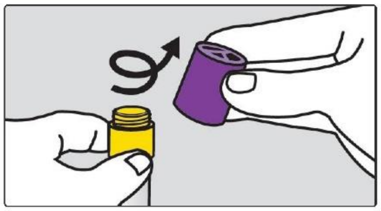

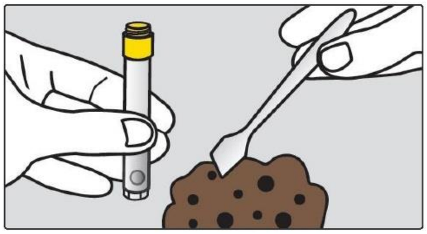

Use the spatula to collect a small amount of fecal sample .

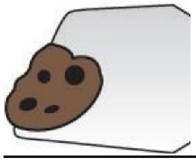

Actual size of fecal sample.

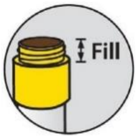

1

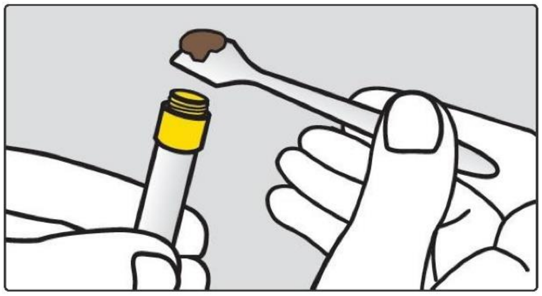

Transfer the fecal sample into the yellow tube top. Repeat until the sample fills the yellow tube top Repeat until the sample fills the yellow tube top.

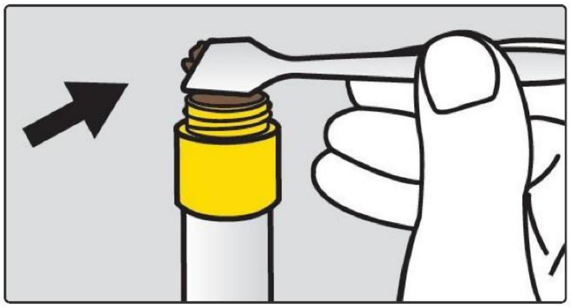

Scrape horizontally across the tube top to level the sample and remove any excess. Wipe exterior of tube top with toilet paper or tissue as needed .

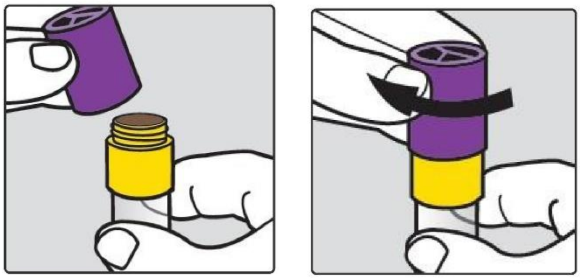

2

Pick up the purple cap and screw onto the yellow tube top until tightly closed.

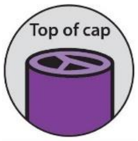

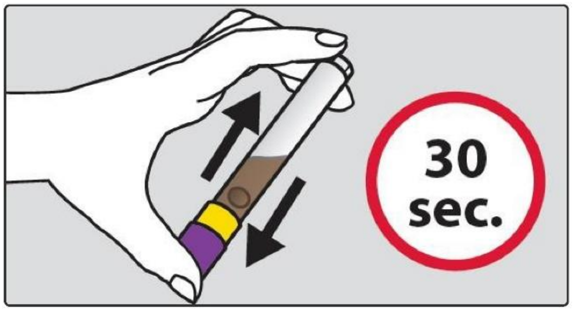

For a **minimum** of 30 seconds, shake the sealed tube as hard and fast as possible in a back and forth mo The fecal sample will be mixed with the stabilizing liquid in the tube; not all particles will dissolve.

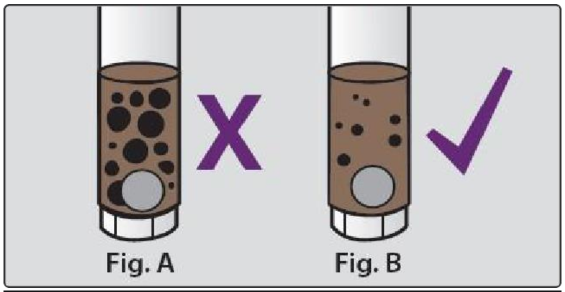

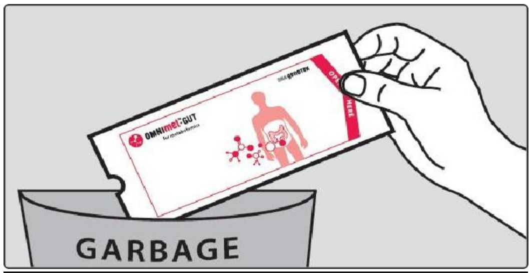

Place spatula in original packaging or wrap in toilet paper and discard in garbage.

IMPORTANT:
Send sample for processing following the delivery instructions supplied separately by the kit provider.

10. Another one scoop of stool will be collected directly from the stool container in a Cryo-Collection tube at the baseline and 36months visits, not to be collected at 24 months visit.
11. Once collected, ensure all three containers are labelled with the participant's 11 digit ID number with collection time and date. Samples can be held (≤24 hours at room temperature), before being aliquoted into labelled cryovials, and stored at -70°C.

E. Research/Field assistant instructions for collecting stool samples from the child at home

1. Do not collect a sample if the participant has had Diarrhea in the last 3 days.
2. Disposable gloves are provided for you to use.
3. We need to receive stool samples as fresh as possible. They should have been passed less than 1 hour before collection if at the home.
4. If the sample is being passed while you are at the home, ask the participant or take help from their mother to empty their bladder, so that urine and stool are not mixed together.

5. Place the Zymo feces Catcher (on the floor or stool pot). So that the child can pass their stool directly onto the catcher. The stool should land in the catcher.
6. If the stool was passed onto a cloth or into a diaper (≤1 hour) prior to you arriving, please collect from this.
7. Use the scoop attached to the brown lid of the Zymo DNA/RNA shield Fecal Collection tube containers to place a heaped scoop of stool into the container. The tube has a liquid solution in it. Please don't tip this liquid out! Once the stool is in this container and the lid is on tightly, please shake this container with stool and liquid in it 10 times vigorously, so that the stool is well mixed with the liquid and no large stool pieces remain visible.
8. Place a scoop of stool, using the spoon provided, into the OMNImet.GUT ME-200 tube. Once the stool is in this container and the lid is on tightly, please shake this container with stool and liquid in it 10 times vigorously, so that the stool is well mixed with the liquid and no large stool pieces remain visible.
9. Another one scoop of stool will be collected directly from the stool container in a Cryo-Collection tube at the baseline and 36months visits, not to be collected at 24 months visit.
10. Ensure all three containers are labelled with the participant's 11 digit ID number with collection time and date. Place the containers with the stool sample into the cooler box. Transport to the laboratory within 2 hours (or at a maximum of 24 hours).
11. Once in the laboratory, samples should be aliquoted into labelled cryovials, and stored at -80°C.

F. Throughout the study, every effort should be made to collect stool specimens within the protocol-specified window for each time point. It is the responsibility of the Field Research Officers (FROs) to track all scheduled stools and their respective collection windows. They should be in communication with FRAs and reference the Specimen Log(s) to monitor that all stools are collected within protocol-specified time points.

G. In order to assure data accuracy, Non diarrheal stool specimens may not be collected outside of the window. If stool specimen collection does not occur during the window specified for the time point, then a missing specimen sheet must be completed to document the missed specimen and send to data management. Additionally, the specimen should be recorded as 'Not Collected' on the relevant CRF.

H. Upon arriving at clinic with gathered stool specimens, a FRA must reconcile any differences in scheduled date of specimen collection from actual date of specimen collection. The FRA will be responsible for correcting the pre-labeled stool pot to

reflect the actual specimen collection if it differs from the scheduled date of specimen collection.
If during the collection time window period any diarrhoea episode is noticed or reported (passage of loose stool for 3 or more times), then recovery from diarrhoea will be allowed. Recovery time from diarrheoa will regarded atleast 72 hours or 3 days.
If diarrhea does not end within the window period of non diarrheal stool or child suffers from any other acute illness , then we will wait for the child's recovery and after completion of 72 hours of recovery from diarrhoea we will collect the non diarrhoeal stool sample irrespective of the window time point . The ICDDR,B lab will be responsible for aliquoting the specimen as necessary.

I. ICDDRB Laboratory Processing of stool samples

Ensure that the 11 digit participant ID is annotated on all tubes and all data is accurately recorded in the clinical management system. A FRA will complete a Child Specimen Requisition Sheet to document the specimen collected. Additionally, the FRA should enter the specimen in the associated Specimen
Log.
Specimens will be sent daily to the ICDDR,B labs. Upon transfer of specimens to ICDDR,B labs, the FRA should complete the Sending Log, noting the specimen ID, date of collection and date of sending. ICDDR,B labs will maintain their own Specimen Receipt Log.

1. Record the date and time the samples were received by the lab.
2. Place Zymo DNA/RNA shield Fecal Collection tube (ZR1101) directly in storage racks and store at -70°C immediately upon completion of processing.
3. Transfer the vortexed homogenized OMNImet.GUT to 3 cryovials that are labelled with 11 digit participant ID. Store at -70°C.

J. Temperature control and storage principles

(1) Samples should be collected during visits (at enrolment [C1]), at time of recovery (~3 months; [R2]), at one year post recovery [Y3], and at 2 years post recovery [F4] during visits to the clinic or at home.

(2) If collected at home, the stool should have been passed within a maximum of one hour before collection by the field assistant. Ideally, it would be passed while the field assistant is present.

(3) Upon collection by the field assistant, samples should be immediately mixed within the Zymo DNA/RNA shield Fecal and OMNImet.GUT ME-

200 collection tubes. Mix until no large particles remain in the solution. Once mixed, place the collection tubes in the cool box.

(4) During transport from the participant’s home to the lab, stool sample tubes should be carried in a cool box with ice packs.

(5) The maximum time between passing a stool and its arrival at the lab for processing is 24 hours.

(6) Upon arrival at the laboratory, stool samples should be aliquoted into labelled cryotubes and stored at -70°C immediately, or at least within 75 minutes of having arrived.

(7) The date of -70 freezer storage should be recorded.

Multidimensional Evaluation of the emergence of executive function and dysfunction in young children in Bangladesh: Pilot Study<br>Study Specific Procedure<br>Staff Training with Quality Control	PR-21084<br><br>Version No:1

Written by : Dr. Talat Shama		Effective date: 21 September 2021

I. Purpose

To outline the plans for training clinic staff and the procedures for documenting staff training to ensure compliance with Good Clinical Practice (GCP) in order to retain and protect rights of human subjects as well as with the study protocol.

II. Methods

A. Pre-employment

It is the responsibility of the site Principal Investigator and the Clinic Manager to ensure that all clinic staff members meet the basic education and training qualifications for their positions prior to an offer of employment or that appropriate training will be provided by the study and documented in cases where key qualifications are lacking.

Every clinic staff should have a record of education and training on file with ICDDR,B Human Resources Department.

B. Initial Training

All hired staff will participate in training provided by the study as appropriate for their roles in the clinic:

• Medical Officers -Specific training.
• Field Research Officers – Study-specific training.
• Phlebotomist/Nurse – Study-specific training.
• Field Research Assistants – Study-specific training.
• Field Assistants – Study-specific training.
• Clinic Assistants – on-the-job training.

Study-specific training will include:

• Study overview
• Data management overview
• Study documentation
• QA/QC and monitoring
• Subject recruitment and screening, consenting and enrolment
• Daily work flow, including relevant procedures (anthropometry, blood and stool collection, providing interventions)
•Adverse events and Serious adverse events
• Protocol deviations

C. Ongoing/Anticipated Training Needs

Clinic management staff will continually evaluate the performance of individual’s and clinic systems through quality control measures and regular staff meetings. Ongoing training needs will

be identified and addressed in consultation with the Clinic Manager, and clinic systems may be modified where necessary at the discretion of the Clinic Manager.
Anticipated training needs include 1) refresher sessions prior to the first occurrence of study procedures; and 2) whenever amendments to the study protocol or consent form are approved by the involved regulatory authorities.
Clinic staff will be trained appropriately whenever protocol or consent form changes are approved. It is important to note that no changes should be made to study procedures prior to approval by regulatory authorities, and training on protocol and/or consent form changes should occur ***after*** amendments receive approval.
All training should be documented according to the training documentation procedure below.

D. Quality Assurance
i. Anthropometry
All Field Research Assistants (FRAs) will receive training on the proper procedures for measuring infants' anthropometry. After training, and prior to conducting anthropometry for study purposes, every FRA must demonstrate proficiency in taking anthropometric measurements to within + 5% of a standard measurement. Standard measurements (weight and height) will be taken on a volunteer infant by a senior FRA with extensive experience in anthropometry.
The same infant will then be measured by other FRAs and results will be documented. Any FRA who does not meet the expected + 5% variation in measurements will not take measurements for study purposes until she has received further training and can meet the target reliably.
Training and testing for anthropometry will be repeated and documented annually for every FRA.

ii. Biological Sample (Blood, Stool, Buccal scrap) :
Field Assistants will be specially trained about the procedure of collection and transportation of stool samples from HH to clinic according to the supplied SOP. Medical technologists and FRAs will be also trained on collecting and preserving all the biological samples (Blood, stool, buccal scrap) according to the SOPs.

iii. Intervention :
Thorough training will be given to FRAs and Health Workers on making and preserving the nutritional supplementation products. A checklist will be made in according to ensure study participant's compliance. Locally produced chick-pea based RUSFs will be produced at a Food laboratory to be set at Mirpur clinic. Whole production procedure will be supervised by an experienced Clinical Nutritionist. The nutritional supplements whicl will be imported (E-RUTF,

SQLNS and E-SQLNS) will also be preserved and administered according to SOPs and staffs will be trained accordingly.
Training on psychosocial stimulation will also be given to all FRAs and psychologists as there will be two groups (intervention and evaluation).
All trainings will be given according to verified valid SOPs.

iv. Neuro-psychological assessment : (Executive Function/ Emotional Reguation, fNIRS,EEG)
Medical Officers, psychologists and FRAs will receive training on Neuropsychosocial assessment according to their designated responsibilities once the SOPs are finalized and verified. Before starting the actual study sessions, few pretests will be run to check the feasibility of the proposed SOPs in Bangladesh context.

v. Monthly in-service trainings
Once per month the clinic management team will provide a half-day in-service training to clinic staff covering a topic(s) identified by the clinic management team. This may include refresher training on study procedures, surveillance, data management, and clinic flow; and/or new training in areas identified for improvement or additions to the clinic work plan.

vi. Monthly shadowing of FRA by FRO
One FRA will be shadowed 20% of her own HH in the community by a Field Research Officer one time per month to ensure continuous quality to GCP and protocol standards in surveillance; and to identify ongoing training needs. Shadowing will be documented and any issues addressed in consultation with the Clinic Manager.

E. Training Documentation
Documentation of training for clinic staff will be maintained by the Clinic Manager in the study file. The following documents should be updated as appropriate:
• LEAP Study Training Log – can be used to document any type of training.
A description of the training material should be completed at the top of the document, and for each staff member attending the training the following information should be recorded:

- o Date of training
- o Staff name
- o Staff ID
- o Staff signature confirming receipt of training

• Authorized Representative Signature Sheet and Delegation Log – This document indicates the tasks each individual staff member is authorized to perform by the site Principal Investigator. Every staff member should be appropriately trained to perform the tasks listed on this form.

• Anthropometry Test Data spreadsheet – This should be used to document initial and annual results of anthropometry training for FRAs.

• Field Shadow Log – Should be maintained by FROs to document monthly shadowing of FRAs.

PR#21084		SID:------------------------

Multidimensional evaluation of the early emergence of executive function and dysfunction in young children in Bangladesh using nutritional and psychosocial intervention: A Pilot study

Perceived Stress Scale Form

ASSESSMENT INFORMATION TO BE DATA ENTERED

	Interviewer: “The questions in this scale ask you about your feelings and thoughts **during the last month**. In each case, you will be asked to indicate by circling *how often* you felt or thought a certain way”.		
	Visit month	○ 12<br>○ 24<br>○ 36	
	Date of visit<br>**(if not administered, enter 09/09/99)**	|__||__| / |__||__| / |__||__| *D D M M Y Y*	
1	In the past month, how often have you been upset because of something that happened unexpectedly?<br>অনাকাংখিত বা অপ্রত্যাশিতভাবে কোন কিছু ঘটে যাওয়ার কারণে গত মাসে আপনার কতবার মন খারাপ হয়েছে?	0 = Never 3 = Quite often<br>1 = Rarely 4 = Frequently<br>2 = Occasionally	|___|
2	In the last month, how often have you felt that you were unable to control the important things in your life?<br>জীবনের গুরুত্বপূর্ণ ব্যাপারগুলো আপনি নিয়ন্ত্রণ করতে পারছেন না-এ বিষয়টি গত মাসে কতবার আপনার মনে হয়েছে?	0 = Never 3 = Quite often<br>1 = Rarely 4 = Frequently<br>2 = Occasionally	|___|
3	In the last month, how often have you felt nervous and stressed?<br>গতমাসে কতবার আপনি মানসিক অস্থিরতা ও চাপ অনুভব করেছেন?	0 = Never 3 = Quite often<br>1 = Rarely 4 = Frequently<br>2 = Occasionally	|___|
4	In the last month, how often have you felt confident about your ability to handle your personal problems?<br>আপনার ব্যক্তিগত সমস্যা সমাধান/নিয়ন্ত্রণ করার সক্ষমতা আপনার রয়েছে গতমাসে কতবার এটি ভেবে আপনি আত্মবিশ্বাসী ছিলেন?	0 = Never 3 = Quite often<br>1 = Rarely 4 = Frequently<br>2 = Occasionally	|___|
5	In the past month, how often have you felt that things were going your way?<br>গতমাসে কতবার আপনার মনে হয়েছে যে-সবকিছু নিজের মন মতো চলছে?	0 = Never 3 = Quite often<br>1 = Rarely 4 = Frequently<br>2 = Occasionally	|___|
6	In the last month, how often have you found that you could not cope with all the things you had to do?<br>গতমাসে কতবার আপনার এমন হয়েছে যে, যতটুকু প্রয়োজন ছিল সে অনুযায়ী সবকিছুর সঙ্গে আপনি খাপ খাওয়াতে পারছেন না?	0 = Never 3 = Quite often<br>1 = Rarely 4 = Frequently<br>2 = Occasionally	|___|
7	In the last month, how often have you been able to control irritations in your life?<br>গত মাসের ঘটে যাওয়া ঘটনার মধ্য থেকে কতবার মনে হয়েছে, বিরক্তিকর বিষয়গুলোকে সামলাতে পেরেছেন?	0 = Never 3 = Quite often<br>1 = Rarely 4 = Frequently<br>2 = Occasionally	|___|
8	In the last month, how often have you felt that you were on top of things?<br>গত মাসে কতবার মনে হয়েছে আপনি সবকিছু সফলভাবে সামলাতে পেরেছেন।	0 = Never 3 = Quite often<br>1 = Rarely 4 = Frequently<br>2 = Occasionally	|___|

9	In the past month, how often have you been angry because of things that happened that was outside of your control?<br>গত মাসে বিভিন্ন ঘটনা আপনার মন মতো না হওয়ার কারণে কতবার রেগে গিয়েছিলেন?	0 = Never 3 = Quite often<br>1 = Rarely 4 = Frequently<br>2 = Occasionally	|___|
10	In the past month, how often have you felt that difficulties were piling up so high that you could not overcome them?<br>গতমাসে কতবার আপনার মনে হয়েছে যে, আপনার সমস্যার সংখ্যা এতটাই বেশি যে সেগুলো তা মোকাবেলা/দূর করা পারবেন না?	0 = Never 3 = Quite often<br>1 = Rarely 4 = Frequently<br>2 = Occasionally	|___|

NOT DATA ENTERED

	Interviewer Name and Code	________________________________	|___||___||___|

International Centre for Diarrhoeal Disease Research, Bangladesh (icddr,b)

Project Title: Multidimensional evaluation of the early emergence of executive function and dysfunction in young children in Bangladesh_using nutritional and psychosocial intervention: A Pilot study

Supervisor Monitoring Form

General Information			
The name of the supervisor:		The name of the stimulation provider:	
The name of the Child:		The name of the mother:	
ID:		The date of the visit:	
Session no:		Address:	
Start time of the visit:		End time of the visit:	
Child's Primary Caregiver:			
**Visit record**		**Answers**	**Code**
1	Dose the child participate in the session?	Yes= 1<br>No= 0	
1.a	If "No," write down the reason.		
2.	With whom the session was conducted?	1. Mother<br>2. Father<br>3. Grand Parent<br>4. Others	
3.	Was there any other person present with the mother?(Most of the time)	Yes =1<br>No = 0	

3(a).	If yes, please write down who was present there.		
4.	Did the stimulation provider discuss any introduction before the stimulation session started?	Yes=1<br>No=0	
5.	Did the stimulation provider properly use the session curriculum?	Yes=1<br>No=0	
৬.	Did the stimulation provider take feedback from previous session?	Yes=1 ,<br>No=0 ,<br>Not applicable= 99<br>(If the answer is 0 or 99, then have to skip the 7 numbers section)	
7. Feedback on the previous session			
7(a)	The mother sang the previous session's rhyme song with the child	Yes= 2<br>Only mother Sang = 1<br>No =0	
7(b)	The mother demonstrated talking about the book with the child from the previous session.	Yes= 2<br>Need more practice = 1<br>No =0	
7(c)	The child played the game from the previous sessions.	Yes= 2<br>Need more practice = 1<br>No =0	
7(d).	The mother demonstrated the language development of the previous session with the child.	Yes= 2<br>Need more practice = 1<br>No =0	
**8. Today's session feedback**			
8(a)	In your opinion, did the stimulation provider prepare in advance for the session?	Yes= 2<br>Need more practice = 1<br>No =0	
8(b).	Did the stimulation provider come up with everything necessary (e.g., guidelines, toys) along with her for the session?	Yes, everything was fine=1<br>Most were right=2<br>Neither was right =0	
8(c).	Did the stimulation provider discuss all the topics of the session?	Yes=1<br>No=0	
8(d)	The mother has sung the song with the child	Yes=3<br>Only the mother sang=2<br>Only the stimulation provider sang=1	

		No one sang=0	
8 (e).	The mother demonstrated talking about the book with the child.	Yes=2<br>Did not show, only the mother talked =1<br>No=0	
8(f).	The mother played with the child	Yes= 2<br>Did not show, only the mother talked = 1<br>No= 0	
8(g)	The child played the game	Yes= 2<br>Need more practice=1<br>Did not/ could not =0	
9.	The stimulation provider explained language development with a real example.	Yes=1<br>No=0	
10.	The stimulation provider took mother opinion on language development.	Yes=1<br>No=0	
11.	How did the stimulation provider explain the sessions to the mother?	Excellent = 3<br>Fairly good = 2<br>Not much better =1<br>very poor = 0	
12.	Has the stimulation provider briefly described the main points of the session to the mother at the end of the session?	Yes=1<br>No=0	
12(a).	Mention if any parts are left.		
13.	How do you feel about the relationship between the mother and Stimulation provider?	Sympathetic=2<br>Formal=1<br>Not caring= 0	
14.	How acceptable is the stimulation provider to the mother?	Mother waited with interest =3<br>Mother was less interested =2<br>Mother was irritated =1	
15.	How did the other members of the family accept the stimulation provider?	Everybody was very interested =3<br>Felt a bit annoyed =2<br>was very upset =1	

16.	Do you think the session was interesting to the mother?	Very much=4<br>Fair to good =3<br>Much less=2<br>Not at all=0	
17.	If the answer to the question of 26 is 3, 2, 1 mention the reason.	Monotonous voice of the stimulation provider=1<br>The provider tried to impose her opinion=2<br>The mother was busy=2<br>Mother was upset because of others =4	
18.	How did the child feel about the session?	Very good=5<br>Fair to good=4<br>The mind was not good=3<br>Cried=2<br>Bored was = 0	

19.	**General observation**	No	1-2 Times	3-5 Times	6 or more Times
19(a).	The stimulation provider praised the mother.				
19(b).	The stimulation provider praised the child.				
19(c).	The mother praised the child				
19(d).	The stimulation provider was attentive to the child (looking into his eyes/smiling, talking, listening to him, pointing).				
20.	If any opinion about the observation and visit				

	Project Title: Multidimensional evaluation of the early emergence of executive function and dysfunction in young children in Bangladesh using nutritional and psychosocial intervention: A Pilot study<br><br>**BAYLEY-4 Qualitative Checking Form**<br><br>**Date:** **QC assessor's Name:**<br>**Testers Name:**<br>**Child Name :** **Child ID:**	Not present	Needs work	Achieved
	**General skills:**			
	**Rapport Build up while entering the household**			
		0	1	2
01	Introduce herself properly.<br>নিজের পরিচয় ভালভাবে দিয়েছে।			
02	Take consent of the caregiver.<br>অভিভাবকের অনুমতি নিয়েছে।			
03	Ensured the child's wellbeing and readiness for test (not sleepy, not hungry, no need for toilet etc.)<br>শিশুর শারীরিক সুস্থ্যতা নিশ্চিত করেছে এবং শিশু টেষ্টের জন্য তৈরী কিনা (ঘুমিয়েছে,খেয়েছে, প্রশাব পায়খানা করেছে কিনা) জেনে নিয়েছে।			
04	Established and maintained rapport with the child.<br>শিশুর সাথে সর্ম্পক স্থাপন ও বজায় রেখেছে।			
05	Clearly explained to the mother that the goal was to gauge the child's aptitude based on age rather to assign a pass or fail grade.<br>যত্নকারী/ মাকে ভালভাবে বুঝিয়ে বলেছে যে, শিশু ও তার বয়স অনুযায়ী কি কি পারে সেটাই/ তাই দেখা হবে, এখানে কোন পাশ ফেল নাই।			
06	Requested mother/others not to interfere during test.<br>মা/ অন্যদেরকে অনুরোধ করেছে যেন শিশুকে খেলার সময় কোন নির্দেশনা না দেয়/ দেখিয়ে না দেয়।			
	**During Test**			
07	Respect and decency were shown towards the child's caregiver/mother and child.<br>যত্নকারী/ মা ও শিশুকে মর্যাদা ও সম্মান দেখিয়েছে।			
8	Explain things in easier terms to caregiver as needed by communicating at their level.<br>যত্নকারী/ মা এর প্রয়োজন অনুযায়ী, ওনাদের বোঝার মত করে সহজভাবে ব্যাখ্যা দিয়েছে।			
9	Responded to the caregiver's questions with patience and sensitivity while showing supportive listening skills.<br>যত্নকারী/ মা এর কথা ভালভাবে ধৈর্য্য ও অনুভুতিশীলভাবে শুনেছে ও সাড়া দিয়েছে।			
10	Calculate the age appropriately.<br>সঠিকভাবে বয়স গণণা করেছে			

11	Fixed the start point accordingly to the child age.<br>বয়স অনুয়ায়ী শুরুর পয়েন্ট নির্ধারিন করেছে।			
12	Maintained reverse rules.<br>পিছনে যাওয়ার নিয়ম অনুসরন করেছে।			
13	Maintained discontinue rules.<br>শেষ করার নিয়ম অনুসরন করেছে।			
14	Administered all test items in order.<br>পরীক্ষার সব আইটেম ক্রমানুসারে পরিচালনা করেছে।			
15	Most of the materials presentation was correct.<br>পরীক্ষার বেশিরভাগ খেলনা/উপকরণ সঠিকভাবে উপস্থাপন করেছে।			
16	All items of the test were conducted accurately and with understanding by the tester.<br>পরীক্ষক পরীক্ষনের সমস্ত আইটেম বুঝে এবং সঠিকভাবে পরিচালনা করেছে।			
17	During test time tester scored all incidental observation items.<br>পরীক্ষার সময় ঘটনাক্রমে পর্যবেক্ষণের মাধ্যমে পরীক্ষক আইটেমে স্কোর করেছে।			
18	The test was administered using items with a specific timeframe.<br>যেসব আইটেমে নির্দিষ্ট সময় আছে সেসব আইটেমে সময় মেনে পরীক্ষাটি পরিচালনা করেছে।			
19	The item is scored by the tester correctly after understanding it.<br>বৃঝতে পারার পরে পরীক্ষক সঠিকভাবে স্কোর করেছেন।			
20	To understand all of the subtests' series and related items and to score each subtest.<br>সিরিজ এবং রিলেটেড আইটেম বুঝতে পেরেছে এবং প্রতিটি সাবটেষ্টে স্কোর করেছে।			
21	Asked the caregiver question where needed according to record form.<br>রেকর্ড ফর্ম অনুসারে যেখানে প্রয়োজন, সেখানে পরিচর্যাকারীর প্রশ্ন জিজ্ঞাসা করেছে।			
22	Engaged the child quickly to the next task before getting exhausted.<br>শিশুটিকে অধৈর্য হবার আগেই দ্রুত পরের খেলায় নিয়ে গেছে।			
23	Didn't forget to give the kid/child a toy.<br>শিশুকে খেলনা দিতে ভুলে যায় নাই।			
24	In between the Item administration the tester offered toys smoothly to the child.<br>দুটি আইটেম পরিচালনার মধ্যবর্তী সময়ে শিশুকে সুন্দরভাবে খেলনা সরবরাহ করেছিলেন।			

25	Efficiently brought the mother on point/or child on track.<br>দক্ষতার সাথে মাকে মুল কথায় ও শিশুকে নির্দিষ্ট খেলার মাঝে ফিরিয়ে এনেছে।			
26	Interacted with child in friendly and entertaining way.<br>শিশুর সাথে হাসি-খুশীভাবে এবং মজা করে খেলেছে।			
27	Encouraged the child to move forward and praise.<br>শিশুকে খেলার জন্য উৎসাহ দিয়েছে এবং প্রশংসা করেছে।			
28	Efficiently and quickly administered tests & scored in record form in time.<br>দক্ষতা ও দ্রুততার সাথে নির্বিঘ্নে টেষ্টগুলো করেছে, খেলনা দিয়েছে এবং রেকর্ড ফর্মে স্কোর করেছে।			
29	Use language that kids can understand. (Bangla, Barmise, Rohingiya)<br>শিশুরা বুঝতে পারে এমন (বাংলা, বার্মিজ, রোহিঙ্গা) ভাষা ব্যবহার করেছে।			
**After test**				
30	Thanked the mother/caregiver for his/her time.<br>মাকে সময় দেবার জন্য ধন্যবাদ দিয়েছে।			
31	After the test, wrap things up after counting the tiny pieces of toy materials.<br>পরীক্ষা/ টেষ্ট শেষে ছোট ছোট খেলনার টুকরাগুলো গুনে গুনে রেখেছে।			
32	After the test, clean the toys using a disinfectant spray.<br>টেষ্ট শেষে জীবানুনাশক স্প্রে ব্যবহার করে খেলনা পরিষ্কার করেছে।			

PR-21084	Form: SES<br>Version 1.0; 22 OCT 2021	**SID: LCC |___|___|___|___|**
**Multidimensional evaluation of the early emergence of executive function and emotional regulation in young children in Bangladesh using nutritional and psychosocial intervention: A Pilot study**		
**SES, Water and Sanitation Case Report Form**		

**Instructions:**	**Complete this form after confirming eligibility and obtaining signed consent.**	
1	Date of Interview	|___|___| |___|___|___| |___|___|___|___| dd mmm yyyy	
2	2a. How many members are in your household?	Please enter exact number: 01, 02, 03, etc.	|___||___|
	2b. Number of living children (less than or equal to 18 years considered as children)		|___||___|
	2c. Birth order of enrolled child among live births		|___||___|
	2d. Number of siblings under 5 years, excluding enrolled child		|___||___|
	2e. How many members are using mobile phone in your household?		|___||___|
3	Ethnicity	1=Bengali 2=Bihari 3=Others	|___|
4	How long have you lived in current household in years? *	00=< 1 year<br>01=1 Year<br>02=2 Years<br>03=3 Years<br>04=4 Years<br>Etc.	|___||___|
5	Number of rooms in current household	Please enter exact number, 01, 02, 03 …	|___||___|
6	Number of people usually sleeping in household		|___||___|
7	Family type	1 = Nuclear 2 = Joint	|___|
8	Language	1=Bangla 2=Urdu 3=Others	|___|
9	Household domestic workers (maid)	1 = Yes 2 = No	|___|
10	Education (please see code list)		
	10a. Father education		|___||___|
	10b. Mother education		|___||___|
11	Occupation (please see code list)		
	11a. Father occupation		|___||___|

Multidimensional evaluation of the early emergence of executive function and emotional regulation in young children in Bangladesh using nutritional and psychosocial intervention: A Pilot study

SES, Water and Sanitation Case Report Form

	11b. Mother occupation		|___||___|
12	Do you own the house you live in?	1 = Yes 2 = No	|___|

*For question 4, half years should be rounded up or down as follows: between 1 and less than 1.5 year = 1 year; between 1.5 and 2 years = 2 years; etc.

13	Monthly income and expenditure		
	**13a. Total monthly Income** (in taka)<br>|___|___|___|___|___|	**13b. Monthly Total expenditure** (in taka):<br>|___|___|___|___|___|	
	**Worksheet for calculating answer to 13a. Add all incomes together and enter total above.**<br>13a1. Household (HH) head's income:<br>|___|___|___|___|___| 13a2. Mother's income: ***If HH head, enter zeros***<br>|___|___|___|___|___| 13a3. Other member's income:<br>|___|___|___|___|___| 13a4. Other sources income:<br>|___|___|___|___|___|	**Worksheet for calculating answer to 13b. Add all expenditures together and enter total above.**<br>13b1. House rent: Enter zeros if own the house<br>|___|___|___|___|___| 13b2. Family expenditure: (Food, Clothes, Utility bills i.e. electricity, Gas, water…)<br>|___|___|___|___|___| 13b3. Other expenditure: (Festival, Medical, Education, gift etc.)<br>|___|___|___|___|___|	
14	What is the principal type of **flooring** in your dwelling?	1 = Earth<br>2 = Bamboo/ Wood<br>3 = Cement	|___|
15	What is the principal **wall material** in your house?	1 = Bamboo/cane/straw<br>2 = Rudimentary walls (mud) or mixed with mud<br>3 = Tin wall<br>4 = Bricks, cement blocks, concrete walls	|___|
16	What is the principal **roofing** material in your house?	1 = Natural material (straw)<br>2 = Rudimentary roofing (polythene or mixed with mud)<br>3 = Finished roof (concrete)<br>4 = Tin roof	|___|

Multidimensional evaluation of the early emergence of executive function and emotional regulation in young children in Bangladesh using nutritional and psychosocial intervention: A Pilot study

SES, Water and Sanitation Case Report Form

**17**	**In your dwelling is there:**		
	17a. Facilities		
	17a1. Electricity	1 = Yes 2 = No	|___|
	17a2. Cooking Gas	1 = Yes 2 = No	|___|
	17a3. Telephone/Mobile	1 = Yes 2 = No	|___|
	17b. Furniture Facilities		
	17b1. Almeria	1 = Yes 2 = No	|___|
	17b2. Table	1 = Yes 2 = No	|___|
	17b3. Chair	1 = Yes 2 = No	|___|
	17b4. Bench	1 = Yes 2 = No	|___|
	17b5. Watch or clock	1 = Yes 2 = No	|___|
	17b6. Cot or bed	1 = Yes 2 = No	|___|
	17b7. Working Radio	1 = Yes 2 = No	|___|
	17b8. Working TV	1 = Yes 2 = No	|___|
	17b9. Bicycle	1 = Yes 2 = No	|___|
	17b10. Motorcycle	1 = Yes 2 = No	|___|
	17b11. Sewing machine	1 = Yes 2 = No	|___|
	17b12. Fan	1 = Yes 2 = No	|___|
18	Exposure to mass media		
	18a. Do you read newspaper?	1 = Regular 2 = Irregularly 3 = No	|___|
	18b. Do you listen/watch Radio/TV?	1 = Regular 2 = Irregularly 3 = No	|___|
	18c. Do you use social media?	1 = Regular 2 = Irregularly 3 = No	|___|
19	What is the principal source of household **DRINKING WATER**?	1 = Municipality supply/piped water<br>2 = Own arrangement by pump<br>3 = Tube well<br>4 = Well/Pond/Canal	|___|

Multidimensional evaluation of the early emergence of executive function and emotional regulation in young children in Bangladesh using nutritional and psychosocial intervention: A Pilot study

SES, Water and Sanitation Case Report Form

20	What is the principal type of **TOILET** facility used by members of your household?	1 = Septic tank or toilet<br>2 = Water-sealed or slab latrine<br>3 = Pit latrine<br>4 = Open latrine<br>5 = Hanging latrine<br>6 = Bush, field as latrine	|___|
21	In terms of household food availability, how do you classify your household?	1 = Deficit in whole year<br>2 = Sometimes deficit<br>3 = Neither deficit nor surplus<br>4 = Surplus	|___|

22	Hand Washing Practice (use the following codes to complete questions 20a1 – 20e1)			
	<u>**Type of Agent**</u><br>1 = Water<br>2 = Mud<br>3 = Ash<br>4 = Soap<br>5 = Other<br>9 = None	<u>**How**</u><br>1 = Left hand<br>2 = Right hand<br>3 = Both hands<br>9 = None	<u>**Source of Water**</u><br>1 = Municipality supply/piped water<br>2 = Own arrangement by pump<br>3 = Tube well<br>4 = Well/Pond/Canal<br>9 = None	
	**Purpose**	**Type of Agent**	**How**	**Source of Water**
	22a1. Before feeding child	|___|	|___|	|___|
	22b1. Before eating (feeding self)	|___|	|___|	|___|
	22c1. After defecating (self)	|___|	|___|	|___|
	22d1. Before cleaning child's dishes	|___|	|___|	|___|
	22e1. After cleaning child's anus	|___|	|___|	|___|
23	Water treatment methods used	1=None<br>2=Let it stand & settle<br>3=Water filter<br>4=Solar disinfection<br>5=Boil<br>6=Strain through cloth<br>7=Add bleach/Chlorine		|___|
24	Toilet facility shared with other households	1 = Yes	2 = No	|___|
25	Frequency of nail cutting of mother	1=Once in a week<br>2=Twice a month<br>3=Once a month<br>4=Once in quarter		|___|

Multidimensional evaluation of the early emergence of executive function and emotional regulation in young children in Bangladesh using nutritional and psychosocial intervention: A Pilot study

SES, Water and Sanitation Case Report Form

26	Place for cooking for household	1=Inside house<br>2=Separate building<br>3=Outdoors<br>4=Other	|___|
27	Type of cooking fuel	1=Gas<br>2=Electric stove<br>3=Wood<br>4=Kerosene oil stove<br>5=Animal dung<br>6=Garments products	|___|
28	Open drain beside your house	1 = Yes 2 = No	|___|

NOT DATA ENTERED

	Interviewer Name and Code	______________________________	|___||___||___|

WOLKE’S Behaviour Ratings

Approach

Initial response to the examiner. The examiner addresses a few introduction remarks to the child and then talks with the mother after giving the child a toy.

Response in the first 5 to 10 minutes is rated. It should be rated immediately, not at the end of the test.

1. Avoiding: shows stray signs of fear - clinging onto the mother/fussing /looking away, withdrawing.

2. Between 1and 3

3. Hesitant: some fear/obviously worried/ wary and watchful/not happy /not smiling/ not fussing/ not readily playing but may be slight touching of toy. May look fleetingly at examiner.
4. Between 3 and 5

5. Accepting: No sign of fear but aware of examiner /not offering/vocalising or smiling at examiner / but looking at her from time to time without fear. Plays with toy but not with vigour.

6. Between 5 and 7

7. Friendly: Not afraid. May smile or vocalise or offer toy to examiner after a few minutes, plays with toy or readily .

8. Between 7 and 9

9. Inviting: Fully accepts examiner, happily. Interacts with her smiling, vocalising and/ or approaching. Obviously enjoys toy, may show enthusiasm in playing.

General Emotional Tone

This scale refers to how unhappy and fussy or cheerful and happy the infant appeared during the examination.

1. Child seems unhappy throughout assessment, gets very upset, cries and fusses for long periods or frequently may protest and wail.

2. Between 1and 3

3. At times rather unhappy begins to fuss with cries. Short verbal protest but may respond happily to some procedures.

4. Between 3 and 5

5. Moderately happy or contented (may smiles once or twice and positively vocalises occasionally in response to some tasks), may become upset occasionally but recovers fairly easily .

6. Between 5 and 7

7. Generally appears to be in a happy state of well being. Smiles often with some excitement. Only becomes briefly unhappy once or twice during the whole assessment.

8. Between 7 and 9

9. Radiates happiness, highly excited, nothing is upsetting (never becomes upset), animated , expressive, smiling and gleeful.

Activity

This scale refers to how physically active the infant was during the testing (gross motor activity).

1. Very still, little gross motor movement. Stays quietly in one place, with practically no self –initiated movement, never wiggles around.

2. Between 1 and 3

3. Usually quiet and inactive, rarely wiggles but responds appropriately in situations calling for some gross motor activities (motor task)

4. Between 3 and 5

5. Moderate activity, wiggles occasionally and may get up or change position a number of times, can be quieted for sedentary tests without much difficulty .

6. Between 5 and 7

7. In action during much of the assessment period, gets up frequently, moves around the room, wiggles, movements are consolable and can be quieted for sedentary tests, however with difficulties sometimes .

8. Between 7 and 9

9. Overactive, on the move all the time, wiggles a lot, cannot be quieted for most of the sedentary tests .

Co-operation

This is a measure of how well the infant co-operates with the examiner and complies with her requests.

1. Resists all suggestions or requests, which are assessment related, very resisting and uncooperative.

2. Between 1 and 3

3. Refuses or resists several specific examinations initially or refuses to co-operate during part of the session (e.g. initially or towards the end).

4. Between 3 and 5.

5. Accepts the assessment or situation , neither cooperative nor resistant in relation to examiner, may occasionally say" No" but will conform .

6. Between 5 and 7.

7. Seems to enjoy the interaction with the examiner, is happy to participate most of the time.

8. Between 7 and 9.

9. Enjoys the session and always complies, readily accepts the examiner's manipulation.

Vocalisation

Vocalisations refer to non- crying utterances or to recognisable utterances embedded in crying. These may be cooing, babbling, consonant sounds or words. Crying per se, no matter how varied, does not qualify.

1. Definitely quiet, 1 or 2 vocalisations.

2. Between 1 and 3.

3. Few vocalisations and of short duration.

4. Between 3 and 5.

5. Vocalisations occur as part of the activities but too intermittent to constitute vocal excitement, chatter or the like .

6. Between 5 and 7.

7. Vocalisations constitute an obvious part of the infant's activity: infant vocalises for the sake of vocalising.

8. Between 7 and 9.

9. Excessive vocalisations, high vocal excitement.
