# Supplementary material for: Multidimensional evaluation of the early emergence of executive function and development in Bangladeshi children using nutritional and psychosocial intervention: A randomized controlled trial protocol
Source: PLoS One. 2024 Mar 15;19(3):e0296529. doi: 10.1371/journal.pone.0296529 (PMC10942035; doi:10.1371/journal.pone.0296529)
Supplement: S3 File — (PDF) [file pone.0296529.s004.pdf]

	**RRC APPLICATION FORM**

RESEARCH PROTOCOL
Number: PR-21084
Version No. 1.00
Version date: 18 July 2021

FOR OFFICE USE ONLY

RRC Approval:	☒ Yes	☐ No	Date:21 Aug 2021
ERC Approval:	☒ Yes	☐ No	Date:21 Sept 2021
AEEC Approval:	☐ Yes	☐ No	Date:
External IRB Approval	☐ Yes	☐ No	Date:
Name of External IRB: ______			

Will the protocol be submitted for expedited review? ☐ Yes ☒ No

If yes, please check all that apply:

- ☐ Outbreak investigattion
- ☐ Secondary Analysis
- ☐ Formative research
- ☐ Short surveys
- ☐ Approved by external IRB (local or abroad) except Randomized Clinical Trial
- ☐ Others (explain the justifications):______
- ☐ Pilot Study
- ☐ Student protocol
- ☐ Observational study

*** Cover Letter to RRC/ERC Chairperson through SDD must be attached.*

Protocol Title:* (maximum 250 characters including space): **Multidimensional evaluation of the early emergence of executive function and emotional regulation in young children in Bangladesh using nutritional and psychosocial intervention: A Pilot study**

Short Title: (maximum **100** characters including space) Evaluation of executive function and emotional regulation in children in Bangladesh

Key Words:* Executive function, Emotional regulation, EEG, fNIRS, Malnutrition

Name of the Research Division Hosting the Protocol:*

- ☐ Health Systems and Population Studies Division (HSPSD)
- ☐ Nutrition and Clinical Services Division (NCSD)
- ☒ Infectious Diseases Division (IDD)
- ☐ Maternal and Child Health Division (MCHD)
- ☐ Laboratory Sciences and Services Division (LSSD)
- ☐ Other (specify) ______

Has the Protocol been Derived from an Activity:* ☒ No ☐ Yes (please provide following information):

Activity No. :
Activity Title:
PI:
Grant No.: Budget Code: Start Date: End Date:

icddr,b Strategic Priority/ Initiative (SP 2015-8):* (check all that apply)

- ☐ Reducing maternal and neonatal mortality
- ☐ Controlling enteric and respiratory infections
- ☒ Preventing and treating maternal and childhood malnutrition
- ☐ Detecting and controlling emerging and re-emerging infections
- ☐ Achieving universal health coverage
- ☐ Examining the health consequences of climate change
- ☒ Preventing and treating non-communicable diseases
- ☐ Others (specify) ______

Research Phase (4 Ds):* (check all that apply)

- ☒ Discovery
- ☒ Development
- ☐ Delivery
- ☐ Evaluation of Delivery

Anticipated Impact of Research:* (check all that apply and please provide details below)

- ☒ Knowledge Production
- ☒ Capacity Building
- ☐ Informing Policy
- ☒ Health and Health Sector Benefits
- ☐ Economic Benefits

**Please provie details here:** This study is proposing nutritional intervention for malnurished chlidren to see whether nutritional interventions along with psychosocial stimulation have any role to improve Executive function and Emotional regulation in comparison with control group. To achieve the objectives, all children will be tested to determine the changes of Executive Function (EF) and Emotional Regulation (ER) in two years follow up period. This study results will give us important information to improve EF and ER in malnourished children and the effect of nutritional intervention on EF and ER.
**Which of the Sustainable Development Goal This Protocol Relates to?:* (**check all that apply**)**<br>☐ 1. End poverty in all its forms everywhere<br>☐ 2. End hunger, achieve food security and improved nutrition and promote sustainable agriculture<br>☒ 3. Ensure healthy lives and promote well-being for all at all ages<br>☐ 4. Ensure inclusive and equitable quality education and promote lifelong learning opportunities for all<br>☐ 5. Achieve gender equality and empower all women and girls<br>☐ 6. Ensure availability and sustainable management of water and sanitation for all<br>☐ 7. Ensure access to affordable, reliable, sustainable and modern energy for all<br>☐ 8. Promote sustained, inclusive and sustainable economic growth, full and productive employment and decent work for all<br>☐ 9. Build resilient infrastructure, promote inclusive and sustainable industrialization and foster innovation<br>☐ 10. Reduce inequality within and among countries<br>☐ 11. Make cities and human settlements inclusive, safe, resilient and sustainable<br>☐ 12. Ensure sustainable consumption and production patterns<br>☐ 13. Take urgent action to combat climate change and its impacts<br>☐ 14. Conserve and sustainably use the oceans, seas and marine resources for sustainable development<br>☐ 15. Protect, restore and promote sustainable use of terrestrial ecosystems, sustainably manage forests, combat desertification, and halt and reverse land degradation and halt biodiversity loss<br>☐ 16. Promote peaceful and inclusive societies for sustainable development, provide access to justice for all and build effective, accountable and inclusive institutions at all levels<br>☐ 17. Strengthen the means of implementation and revitalize the global partnership for sustainable development

**Does this Protocol Use the Gender Framework:***<br>(Please visit: http://shetu.icddrb.org/index.php?option=com_content&view=article&id=265&Itemid=677 for Gender Alanysis Tool with instructions)	☐ Yes (please complete Gender Analysis Tool)<br>☒ No
If 'no' is the response, its reason(s) in brief: There is no enough data on multidementional evaluation on executive function (EF) in Bangladesh, and since executive functions are at the heart of children making the successful transition to school and later, to society, it is essential to develop a normative template for EF and to determine how malnutrition impacts the development of EF	
**Will this Research Specifically Benefit the Disadvantaged (**economically, socially and/or otherwise**):**	☒ Yes<br>☐ No
**Does this Protocol use Behaviour Change Communication:**	☐ Yes<br>☒ No

Contribution by the Members of the Scientific Team:

Members' Name	Contribution								
	Research idea/ concept	Study design	Protocol writing	Respond to external reviewers' comments	Defending at IRB	Developing data collection Tool(s)	Data Collection	Data analysis/ interpretation of results	Manuscript writing
Dr. Rashidul Haque	☒	☒	☒	☒	☒	☐	☐	☒	☒
Dr. Masud Alam	☐	☒	☒	☒	☒	☒	☐	☐	☐
Dr. Sharia Hafiz	☐	☐	☒	☐	☒	☒	☒	☐	☐
Dr. Talat Shama	☐	☐	☒	☐	☒	☒	☒	☐	☐
Prof. Charles Nelson	☒	☒	☐	☒	☐	☐	☐	☒	☒
Prof. Terrence Forrester	☒	☒	☐	☒	☐	☐	☐	☒	☒
Sir Peter Gluckman	☒	☒	☐	☐	☐	☐	☐	☒	☒
Dr. Fahmida Tofail	☐	☒	☒	☐	☐	☒	☒	☒	☐
Kirk Ericksion	☐	☐	☐	☐	☐	☐	☐	☒	☐
Cameron-Smith	☒	☐	☐	☐	☐	☐	☐	☐	☐
Mr Mamane Zeilani	☐	☐	☒	☐	☐	☐	☐	☐	☒
Jack A Gilbert	☒	☐	☒	☐	☐	☐	☐	☒	☐
Jukka Matias	☐	☒	☐	☐	☐	☐	☐	☒	☒
Dr. Justin Martin	☐	☒	☐	☐	☐	☐	☐	☒	☒
	☐	☐	☐	☐	☐	☐	☐	☐	☐

Study Population: Sex, Age, Special Group and Ethnicity

Research Subject:
☒ Human
☐ Animal
☐ Microorganism
☐ Other (specify): ______________

Sex:
☒ Male
☒ Female
☐ Transgender

Age:
☒ 0 – 4 years
☐ 5 – 10 years
☐ 11 – 17 years
☐ 18 – 64 years
☐ 65 +

Special Group:
☐ Pregnant Women
☐ Fetuses
☐ Prisoners
☐ Destitutes
☐ Service Providers
☐ Cognitively Impaired
☐ CSW
☐ Expatriates
☐ Immigrants
☐ Refugee
☐ Others (specify): _________________

Ethnicity:
☒ No ethnic selection (Bangladeshi)
☐ Bangalee
☐ Tribal group
☐ Other (specify): _________________

NOTE: It is icddr.b's policy to include men, women, children and transgender in its research projects involving participation of humans, unless there is strong justification(s) for their exclusion.

Consent Process: (Check all that apply)
☒ Written
☐ Oral
☐ Audio
☐ Video
☐ None
*(**if anyone of the above is checked except 'None' consent forms must be attached).*

Language:
☒ Bangla
☐ English
☐ Other (specify: ________________

a) Will study tools/questionnaire be used for this protocol?	☒ Yes ☐ No ☐ Not applicable<br>(If yes, tools/questionnaire must be attached). Attached

Project/Study Site: (Check all that apply)

- ☐ Chakaria
- ☐ Bandarban
- ☐ Dhaka Hospital
- ☐ Kamalapur Field Site/HDSS
- ☒ Mirpur (Dhaka)
- ☐ Matlab DSS Area
- ☐ Matlab non-DSS Area
- ☐ Matlab Hospital
- ☐ Mirzapur
- ☐ Bianibazar (Sylhet)
- ☐ Kanaighat (Sylhet)
- ☐ Jakigonj (Sylhet)
- ☐ Other community in Dhaka
  Name: ____________________________
- ☐ Other sites in Bangladesh
  Name: ____________________________
- ☐ Multi-national Study
  Name of the country___________________

Project/Study Type: (Check all that apply)

- ☐ Case Control Study
- ☐ Clinical Trial (Hospital/Clinic/Field)*
- ☒ Community-based Trial/Intervention
- ☐ Cross Sectional Survey
- ☐ Family Follow-up Study
- ☐ Longitudinal Study (cohort or follow-up)
- ☐ Meta-analysis
- ☐ Programme Evaluation
- ☐ Programme (Umbrella Project)
- ☐ Prophylactic Trial
- ☐ Record Review
- ☐ Secondary Data Analysis
  Protocol No. of Data Source: ___________
- ☐ Surveillance/Monitoring
- ☐ Systematic Review
- ☐ Other (specify): ____________________

***Note:** International Committee of Medical Journal Editors (ICMJE) defines Clinical Trial as "*Any research project that prospectively assigns human participants to intervention and comparison groups to study the cause-and-effect relationship between a medical intervention and a health outcome*".

PI of the RRC- and ERC-approved Clinical Trials should provide necessary information to IRB Secretariat (Research Administration) for registration and uploading into relevant websites (usually at the https://register.clinicaltrials.gov/). They should also provide relevant information to the IRB Secretariat in the event of amendment/modification after their approval by RRC and ERC.

In case of a multi-country study and if a study is registered elsewhere by the prime recipient or others; it does not need to be re-registered under icddr,b's account; provided evidence of NCT registration number is submitted to the IRB.

Biological Specimen:

a) Will the biological specimen be stored for future use?	☒ Yes ☐ No ☐ Not applicable
b) If the response is 'yes', how long the specimens will be preserved?	5 years
c) What types of tests will be carried out with the preserved specimens?	Microbiome, metagenomics, epigenomics
d) Will the consent be obtained from the study participants for use of the preserved specimen for other initiative(s) unrelated to this study, without their re-consent?	☒ Yes ☐ No ☐ Not applicable
e) Will the specimens be shipped to other country/ countries? If yes, name of institution(s) and country/countries.	☒ Yes ☐ No ☐ Not applicable<br>Name: University of Auckland, New Zealand
f) If shipped to another country, will the surplus/unused specimen be returned to icddr,b? If the response is 'no', then the surplus/unused specimen must be destroyed.	☐ Yes ☒ No ☐ Not applicable<br>Unused specimen will be destroyed
g) Who will be the custodian of the specimen at icddr,b?	
h) Who will be the custodian of the specimen when shipped outside Bangladesh?	
i) Who will be the owner(s) of the specimens?	icddr,b
j) Has a MoU been signed with regards to collection, storage, use and ownership of specimen? If the response is 'yes', please attach a copy of the MoU.. If the response is 'no', appropriate justification should be provided for not signing a MoU.	☐ Yes ☒ No ☐ Not applicable<br>MoU will be prepared

Proposed Sample Size:
Sub-group (Name of subgroup e.g. Men, Women) and Number

Name	Number	Name	Number
(1) 1-Year old normal with WHZ score>-1SD children with mother	70+70	(3) 3-year-old children with WHZ <-2 and ≥-3 z-score, and/or MUAC <12.5 and ≥11.5 cm stable with moderate acute malnutrition (MAM/ with mother	70+70
(2) 1-year-old children with WHZ <-2 and ≥-3 z-score, and/or MUAC <12.5 and ≥11.5 cm having clinical moderate acute malnutrition (MAM) with mother	140+140	(4)	
		**Total sample size: (Child+ Mother)**	280+280= 560

Determination of Risk: Does the Research Involve

(Check all that apply)

☐ Human exposure to radioactive agents?
☐ Foetal tissue or abortus?
☐ Investigational new device?
  Specify: ___________________
☐ Existing data available from Co-investigator?
☐ Human exposure to infectious agents?
☐ Investigational new drug?
☐ Existing data available via public archives/sources?
☒ Pathological or diagnostic clinical specimen only?
☐ Observation of public behaviour?
☐ New treatment regime?

	Yes	No
Will the information be recorded in such a manner that study participants can be identified from the information directly or through identifiers linked to the study participants?	☒	☐
Does the research deal with sensitive aspects of the study participants' sexual behaviour, alcohol use or illegal conduct such as drug use?	☐	☒
**Could information on study participants, if available to people outside of the research team:**		
a) Place them at risk of criminal or civil liability?	☐	☒
b) Damage their financial standing, reputation or employability, or social rejection, or lead to stigma, divorce etc.?	☐	☒

Do you consider this research: (check one)

☐ Greater than minimal risk	☒ No more than minimal risk	☐ Only part of the diagnostic test

Note: Minimal Risk: The probability and the magnitude of the anticipated harm or discomfort to participants is not greater than those ordinarily encountered in daily life or during the performance of routine physical, psychological examinations or tests, e.g. the risk of drawing a small amount of blood from a healthy individual for research purposes is no greater than when the same is performed for routine management of patients.

**Risk Group of Infectious Agent and Use of Recombinant DNA**	
a) Will specimens containing infectious agent be collected?	☐ Yes ☒ No ☐ Not applicable
b) Will the study involve amplification by culture of infectious agents?	☐ Yes ☒ No ☐ Not applicable
c) If response to questions (a) and/or (b) is 'yes', to which Risk Group (RG) does the agent(s) belong? (Please visit http://shetu.icddrb.org/index.php?option=com_content&view=article&id=265&Itemid=677 to review list of microorganism by Risk Group)	☐ RG1 ☐ RG2 ☐ RG3 ☐ RG4
d) Does the study involve experiments with recombinant DNA?	☐ Yes ☒ No ☐ Not applicable

Does the study involve any biohazards materials/agents or microorganisms of risk group 2, 3, or 4 (GR2, GR-3 or GR4)?

☐ Yes ☒ No

[If the response is 'yes'] I, (print name of the PI) affirm that we will use the standard icddr,b laboratory procedures for biosafety of the hazardous materials/agents or microorganisms in the conduction of the study.

Signature of the Principal Investigator **Date**

Dissemination Plan: [please explicitly describe the plans for dissemination, including how the research findings would be shared with stakeholders, identifying them if known, and the mechanism to be used; anticipated type of publication (working papers, internal (institutional) publication, international publications, international conferences/seminars/workshops/ agencies. [Check all that are applicable]

Dissemination type	Response		Description (if the response is a yes)
Seminar for icddr,b scientists/ staff	☐ No	☒ Yes	Seminars at IDD at the icddr,b
Internal publication	☒ No	☐ Yes	
Working paper	☒ No	☐ Yes	
Sharing with GoB (e.g. DGHS/ Ministry, others)	☐ No	☒ Yes	Seminar or sharing study report with GoB
Sharing with national NGOs	☒ No	☐ Yes	
Presentation at national workshop/ seminar	☐ No	☒ Yes	Through abstract submission at a relevant national workshop/seminar
Presentation at international workshop/ conference	☐ No	☒ Yes	Through abstract submission at a relevant international workshop/conference
Peer-reviewed publication	☐ No	☒ Yes	Publication in peer-reviewed journals
Sharing with international agencies	☐ No	☒ Yes	Sharing study report if necessary
Sharing with donors	☐ No	☒ Yes	Through annual and final reports
Policy brief	☒ No	☐ Yes	
Other			
Other			

Funding:

Is the protocol fully funded?	☒ Yes	☐ No
If the answer is yes, please provide sponsor(s)'s name	1. Welcome Leap	
	2.	
Is the protocol partially funded?	☐ Yes	☒ No
If the answer is yes, please provide sponsor(s)'s name	1.	
	2.	

If fund has not been identified: NA

Is the proposal being submitted for funding?	☐ Yes	☐ No
If yes, name of the funding agency	1.	
	2.	

Conflict of interest:
Do any of the participating investigators and/or member(s) of their immediate families have an equity relationship (e.g. stockholder) with the sponsor of the project or manufacturer and/or owner of the test product or device to be studied or serve as a consultant to any of the above?

☒ No | ☐ Yes (please submit a written statement of disclosure to the Executive Director, icddr,b)

Proposed Budget:

Dates of Proposed Period of Support

(Day, Month, Year - DD/MM/YY)

Beginning Date : 01/09/2021

End Date : 31/08/2024

Cost Required for the Budget Period ($)

Certification by the Principal Investigator:

I certify that the statements herein are true, complete and accurate to the best of my knowledge. I am aware that any false, fictitious, or fraudulent statements or claims may subject me to criminal, civil, or administrative penalties. I agree to accept the responsibility for the scientific conduct of the project and to provide the required progress reports including updating protocol information in the NAVISION if a grant is awarded as a result of this application.

I also certify that I have read icddr,b Data Policies and understand the PIs' responsibilities related to archival and sharing of research data, and will remain fully compliant to the Policies. (Note: The Data Policies can be found here: http://shetu.icddrb.org/index.php?option=com_content&view=article&id=273&Itemid=685)

Signature of PI **Date**

Approval of the Project by the Division Director of the Applicant:

The above-mentioned project has been discussed and reviewed at the Division level.

Name of the Division Director Signature Date of Approval

Table of Contents

RRC APPLICATION FORM 1
Project Summary 10
Hypothesis to be tested: 12
Specific Objectives: 12
Background of the Project including Preliminary Observations: 12
Research Design and Methods 17
Sample Size Calculation and Outcome (Primary and Secondary) Variable(s) 30
Data Analysis 30
Data Safety Monitoring Plan (DSMP) 31
Ethical Assurance for Protection of Human rights 32
Use of Animals 33
Collaborative Arrangements 33
Facilities Available 33
Literature Cited 34
Budget .... **Error! Bookmark not defined.**
Other Support 39
Biography of the Investigators .... **Error! Bookmark not defined.**
Format for Consent Form .... **Error! Bookmark not defined.**
Check-List .... **Error! Bookmark not defined.**

☐ Check here if appendix is included

List of Abbreviations

EF: Executive Function

EDF: Executive Dysfunction

ER: Emotional Regulation

EDR: Emotional Dysregulation

SD: Standard Deviation

MAM: Moderate acute malnutrition

RUSF: Ready to Use Supplimentary Food

E-RUTF: Enhanced Ready to Use Therapeutic food

SQLNS: Small Quantity Lipid Nutrient Supplement

E-SQLNS: Enhanced Small Quantity Lipid Nutrient Supplement

fNIRS : functional Near Red spectroscopy

EEG: Electroencephalogram

WHO: World Health Organization

MUAC: Mid Upper Arm Circumference

icddr,b: international centre for diarrheal disease and research , Bangladesh

wt: weight

ht: height

WHZ: Weight for height Z score

RRC: Research Review Committee

ERC: Ethical Review Committee

Project Summary

[The summary, within a word limit of 300, should be stand alone and be fully understandable.]

Principal Investigator: Dr. Rashidul Haque
Research Protocol Title: Multidimensional evaluation of the early emergence of executive function and emotional regulation in young children in Bangladesh using nutritional and psychosocial intervention: A Pilot Study
Proposed start date: 01 Sept 2021 Estimated end date: 31 August 2024

Background (brief):

a. Burden: Children, who have moderate malnutrition in early life, can suffer from long term adverse neuropsychological consequences, leading to impaired cognitive performance (specifically executive function) and emotional regulation. This in turn diminishes their future developmental potential and contributes to poor school performance, reduced productivity hyperactivity and reduced attention, and greater likelihood of conflict with the law, all tributaries of sub-optimal human capital accumulation.

b. Knowledge gap: Reversing malnutrition-induced impairment of cognition and emotional regulation with effective and scalable interventions is a critical global gap that we address. Doing so early in the life cycle while the brain remains plastic is hampered by inadequate tools to measure and track the aspects of cognition most related to poor outcomes, namely executive function and emotional regulation.

c. Relevance: Malnutrition affects 47 million children < 5yr annually, most in low and low middle-income countries. Half of all deaths in under-fives is related to malnutrition. Survivors of moderate and severe acute malnutrition today number around 2 billion. This is a big global burden that slows sustainable development in poor countries where human capital is the most important asset to achieve this goal

Hypothesis (if any):

Nutritional deficiencies in malnourished children in early life leads to neuropsychological sequelae in childhood causing negative impacts on the development of executive functions and emotional regulation

Primary Objective:

1. To study Executive Function (EF) and Emotional Regulation (ER) development in young children in Bangladesh where malnutrition and social adversities are common in children.

Secondary Objectives:

1. Whether nutritional interventions along with psychosocial stimulation contribute to improvement in Executive Dysfunction (EDF) and Emotional Dysregulation (EDR) among children with malnutrition
2. Whether microbiome assembly, metabolomics, child genetics and epigenetics contribute to Executive Function/Dysfunction and Emotional Regulation/Dysregulation in normal and malnourished children at baseline and after nutritional intervention.

Methods:

To attain the objectives of this study we have designed a prospective nutritional intervention study in children in Mirpur, Dhaka. We will recruit 70 adequately nourished 1-year old children (WHZ score>-1SD) and 140 stable 1-year old MAM children (WHZ <-2 and ≥-3 z-score, and/or MUAC <12.5 and ≥11.5 cm). These 140 children will be randomized to 1:1 and will receive either locally produced Ready to Use Supplementary Food (RUSF) or Enhanced Ready to Use Food (E-RUTF) until anthropometric recovery (WHZ > - 1SD) has been achieved or for maximum 3 months after enrolment and then who randomized for RUSF they will receive Small Quantity Lipid Based Nutrient

Supplement (SQLNS) and who randomized for E-RUTF , will receive Enhanced Small Quantity Lipid Based Nutrient Supplement (E-SQLNS) till the end of the 2 years of follow up period to maintain the nutritional status after recovery by RUSF or E-RUTF supplement. They will be followed up to 3 years of age with biological sample collection, anthropometry and neurocognitive assessment at years 2 and 3. Standard psychosocial stimulation will also be given to all these 140 children. As the final outcomes are to be assessed at 3 years of age, we will recruit another arm of 70, 3years-olds previously untreated MAM children (WHZ <-2 and ≥-3 z-score, and/or MUAC <12.5 and ≥11.5 cm) as a reference group for Executive Function (EF) and Emotional Regulation (ER) outcome. This MAM reference group of children will be assessed only once with our toolkits at the age of 3 years, then we will also provide them 2 months nutritional support with RUSF for their nutritional rehabilitation.

Interventions:

- Nutritional supplementation
- Psychosocial intervention

Outcome measures/variables:

1. Neuropsychological analyses:
   - Executive Function/ Emotional Regulation
   - Resting state fNIRS and EEG functional connectivity
   - Power and coherence across the EEG spectra
   - Amplitude and latency of ERP components
   - Eye tracking data

2. Anthropometry: Weight, Height. MUAC, Head circumference

3. Blood analytes: Concentration of vitamins, and micro nutrients at the time of enrolment, at the time of anthropometric recovery and then at annual evaluations.
Functional lipid concentrations in red cell membrane and serum Sialylated milk oligo saccharides Lutein/zeaxantnine/cryptoxanthine, blood metabolome

4. Stool analytes: 16S microbial sequencing, Functional pathway analysis and stool metabolome

5. Buccal scrab: Genetics and epigenetics

Description of the Research Project

Hypothesis to be tested:

In a hypothesis testing research proposal, briefly mention the hypothesis to be tested and provide the scientific basis of the hypothesis, critically examining the observations leading to the formulation of the hypothesis.

Does this research proposal involve testing of hypothesis: ☐ No ☒ Yes (describe below))

Nutritional deficiencies in malnourished children in early life leads to neuropsychological sequelae in childhood causing negative impacts on the development of executive functions and emotional regulation

Specific Objectives:

Describe the specific objectives of the proposed study. State the specific parameters, gender aspects, biological functions, rates, and processes that will be assessed by specific methods.

Primary Objective:

To study Executive Functions (EFs) and Emotional Regulation (ER) development in young children in Bangladesh where malnutrition and social adversities are common in children.

Secondary Objectives:

1. Whether nutritional interventions along with psychosocial stimulation contribute to improvement in Executive Dysfunction (EDF) and Emotional Dysregulation (EDR) among children with moderate malnutrition
2. Whether microbiome assembly, metabolomics, child genetics and epi-genetics contribute to Executive Function/Dysfunction and Emotional Regulation/Dysregulation in normal children and MAM children at baseline and after nutritional intervention.

Background of the Project including Preliminary Observations:

Provide scientific validity of the hypothesis based on background information of the proposed study and discuss previous works on the research topic, including information on sex, gender and diversity (ethnicity, SES) by citing specific references. Critically analyse available knowledge and discuss the questions and gaps in the knowledge that need to be filled to achieve the proposed aims. If there is no sufficient information on the subject, indice the need to develop new knowledge.

Malnutrition affects ~47 million children under 5 years of age annually and underlies 45% of the mortality in low- and middle-income countries (LMICs) where ~2 billion survivors suffer long term cognitive and behavioural sequelae (1). Acute malnutrition, comprising both MAM (Moderate Acute Malnutrition) and SAM (Severe Acute Malnutrition) causes 14.6% of all deaths of children under 5 years of age globally (2,3). It is a significant problem in Bangladesh where 40% of under-fives have moderately acute malnutrition. Undernutrition is further worsened by poor dietary diversity, with diet comprising 70% cereals, and inadequate protein and micronutrient intake (4). Inadequate infant and young child feeding (IYCF) practices leading to deficiencies in vitamin A, zinc, B12, and folate, as well as maternal and child anaemia, continue to be concerns (5). The long-term consequences of malnutrition should be a noteworthy concern for Bangladesh, since about 5.5 million children under 5 years (36 percent) are suffering from chronic malnutrition (stunting or low height-for-age) and 14% are acutely malnourished (wasting or low weight-for-height) (6). Studies show that, during infancy and early childhood, nutrition is essential as these are the developmental periods critical for the formation of the brain, building the foundation for the development of cognitive, motor, and socio-emotional skills throughout childhood and adulthood. Hence, nutritional deficiencies during early childhood are likely to affect cognition, behaviour, school performance and productivity in later life leading to long term sequalae (7).

There was a study conducted at Bogota, Colombia where selected malnourished children from poor families received food supplementation for the entire family, from mid-pregnancy until the target child was 3 years old (8-10). A control group received twice weekly home visits for the same period in order to provide comparable stimulation. At 3 years of age children who received the food supplementation averaged 2.6 cm and 642 grams larger than controls. The supplementation resulted in small but significant improvements in the Bayley test scores compared with the control group. Similar findings were reported from the Bacon Chow Study in Taiwan (11)**.** The long-term effects of early malnutrition on behaviour and cognition can be minimized by feeding supplementation and psychosocial stimulation (12). Another study in Cali, Colombia provided 1,2 or 3 years of nutritional supplements to slum children and also stimulation during the day for 6 days a week. The combined treatment brought about significant development in cognition test performance compared to the control children (13,14). Therefore, it is a testable hypothesis, that reversing malnutrition-induced Executive Dysfunction (EDF)and Emotional Dysregulation (EDR) can be avoided/reversed by nutritional interventions delivered during rehabilitation from acute wasting in malnourished children when brains are most plastic. It is noteworthy that current malnutrition rehabilitation best practice does not erase cognitive or EDF/EDR impairment ultimately leading to a failure to accrue human capital in countries where achievement of sustainable development is primarily related to this asset.

Malnourished children have an abnormal assembly of the early gut microbiota (15) which may impair brain function by disturbing the bidirectional neural and immune interactions between gut and brain by altered production of signal molecules by the microbiota such as short-chain fatty acids, and neurotransmitters (16). Nutritionally wasted children notably have marked brain atrophy on MRI and while re-feeding reverses brain atrophy, significant deficits remain in function and microstructure (17). It is likely that the anatomic reconstitution of brain with feeds designed principally for corporal rapid catch-up growth results in brain structure which is unable to provide substrate for normal cognitive and emotional performance. Fixing this must depend at least in part on provision of the appropriate nutrients in amounts that meet demands during rapid catch-up growth of body brain.

We think nutrient deficiencies, and gut microbiome dysbiosis both induce structural and functional abnormalities of the brain in malnutrition that lead to neuropsychological sequelae in childhood and later life (18,19). The human brain develops during intrauterine life as well as early childhood, especially in the developmental window between birth and 3 years of age. (20). Better recovery of brain architecture and function in children suffering malnutrition will result from augmenting feeds with key nutrients with targeted functionality in the brain during rapid brain regrowth. The supplements are WHO/UNICEF E-RUTF Ready to Use Therapeutic Feed-enhanced) and E-SQLNS (standard/enhanced small quantity lipid based nutrient supplements) all containing key nutrients for rehabilitating wasted brains: 24 micronutrients (vitamins and minerals) provided at RDA (Recommended Daily Allowance) levels, functional lipids (Long Chain Polyunsaturated Fatty Acids DHA and EPA), sialylated milk oligosaccharides, neural specific antioxidants (zeaxanthine, lutein; crypto-xanthine) and microbiome modulating dietary soluble fibre mix(inulin + FOS), 6 g per 26g daily dose of E-SQLNS, as well as within a daily 100g ration of E-RUTF (21-24) .

To meet Sustainable Development Goal (SDG)-3.2 which addresses reduction in child mortality, effective and beneficial nutritional interventions to improve acute malnutrition in children under 5 years of age must be implemented and preventive measures should be implied alongside (25). The risk of death associated with severity of malnutrition. Besides SAM, early diagnosis and treatment of MAM is equally essential to reduce morbidity as well as child mortality.

The diagnostic criteria for MAM in children 6 to 59 months of age are weight-for-height z-score <-2 and ≥-3 z-score of WHO child growth standards and/or MUAC <12.5 and ≥11.5 cm. This definition is also supported USAID (26). There are about 1.8 million children under 5 years of age in Bangladesh with MAM. The current WHO recommendations for management of SAM are based on facility-based treatment for initial stabilization and nutritional rehabilitation followed by continued management at home (26). It has been observed in studies that, home-based management with ready-to-use therapeutic food (RUTF) in children suffering from SAM has been associated with positive outcomes (3). But these are yet to be used in public in Bangladesh except used by some non-government organizations (27, 28).

Also, The National Nutrition Program of Bangladesh used to provide a supplementary food called Pushti packet for the management of severely underweight children. This supplementary food is a mixture of toasted rice powder, roasted lentil powder, molasses, and vegetable oil. A study from icddr,b was carried out recently where three group of children each group consisting of 30 children with severe underweight received 50 gms of Pushti packets or locally produced Ready to Use Supplementary Food (RUSF), chickpea or rice lentil based and concluded that the newly developed both rice lentil and chickpea-based RUSFs are acceptable to children and their caregivers and nutritionally more complete than Pushti packet (29). However, we are planning to provide energy dense chickpea-based RUSF targeted calorie at 250 kcal/50 g (per serving) with caloric distribution 45–50 percent from fat and 8–10 percent from protein (29)

Table 1: **A typical recipe for Chickpea based Ready to Use Supplimentary Food (50g/serving)**

	**Chickpea based RUSF 50g/serving**
Energy (Kcal)	267
Moisture (g)	1.2
Protein (g)	5.1
Total Fat (g)	15.9
Carbohydrate (g)	24.9
Dietary fibre (g)	0.6
Ash (g)	2.5
Vitamin A (microgram)	294
B carotene (microgram)	26.5
Vitamin C (mg)	20.5
Vitamin E (mg)	14
Vitamin B_1 (mg)	0.78
Vitamin B_2 (mg)	0.63
Calcium (mg)	413.3
Phosphorus (mg)	318
Sodium (mg)	37
Potassium (mg)	424.5
Magnesium (mg)	71.5
Iron (mg)	7.0
Copper (mg)	0.4
Zinc (mg)	4.9
Chloride (mg)	68.5
Aflatoxin	ND
Water activity (24.6^0C)	0.32
pH	4.1

Peroxide value	0

Source : Ahmed et al. BMC Pediatrics 2014, 14:164 Page 5 of 8 http://www.biomedcentral.com/1471-2431/14/164

A typical recipe for Ready to Use Therapeutic Food (Table 2) used by projects supported and approved by World Food Programme and UNICEF : (30) (Manary, 2005)

Table 2: A typical recipe for Enhanced Ready to Use Therapeutic Food (92g serving)

	**E_RUTF** (d dose/92g)
Total energy (kcal)	500
Protein kcal (% of total energy)	10.3
Proteins (g)	12.8
Dairy proteins (% of total proteins)	50
PDCAAS (Protein Digestibility Corrected Amino Acid Score)	1
Lipid kcal (% of total energy)	55
Lipids (g)	30.3
ALA[a] energy (% of total energy)	1.7
ALA[a] (g)	0.94
LA[b] energy (% of total energy)	6.3
LA[b] (g)	3.40
Ratio LA/ALA	3.6
DHA (mg)	80
EPA (mg)	135
Total fibers (g)	5.5
Prebiotics (combined Inulin & FOS)	6
Calcium (mg)	302
Phosphorus (mg)	343
Potassium (mg)	1171
Magnesium (mg)	80
Zinc (mg)	11.8
Copper (mg)	1.5
Iron (mg)	10.3
Iodine (µg)	98
Selenium (µg)	28
Sodium (mg)	165
Vitamin A / Retinol (µg)	790
Lutein (mg)	0.944
Zeaxanthin (mg)	0.044- 0.1
Beta – Cryptoxanthin (mg)	1-2
Vitamin D (µg)	14
Vitamin E (mg)	18.4
Vitamin C (mg)	46
Vitamin B1 (mg)	0.50
Vitamin B2 (mg)	1.5

Vitamin B6 (mg)	0.55
Vitamin B12 (µg)	1.5
Vitamin K (µg)	14.4
Biotin (µg)	56
Folic acid (µg)	184
Pantothenic acid (mg)	2.8
Niacin (mg)	4.6

aAlpha- Linolenic Acid; bLinoleic Acid; c CMAM Statement, UN agencies, 2007: CMAM (Community-based Management of Acute Malnutrition), a Joint
Statement by the World Health Organization, the World Food Programme, the United Nations System Standing Committee on Nutrition and the United
Nations Children's Fund, 2007

Though previously, as Lipid Based Nutrient Supplement , Nutri-butter has been used in several studies in countries like Zimbabwe , Niger and Malawi and showed promising results in gaining weight as well as acceptability (31-33), however, we also prefer an Enhanced SQLNS which is more nutrient rich than Nutributter as it differ in composition to Nutributter since in addition it contains vitamins and probiotics like Inulin & FOS, DHA, EPA, Lutein, Zeaxanthin, Beta Cryptoxanthin, 34-37) which we presume will be more beneficiary for the malnourished children.

According to the National Guidelines for Bangladesh, as well as WHO, the nutritional management of MAM to support catch up growth means adding at least 25kcal/kg/day over and above the energy requirements of a well-nourished child. This should be done by encouraging increased intake of home food. The staple cereal (rice) should be fortified with micronutrient powder and animal source of food (fish, egg, milk etc.) included in the diet along with 6 monthly De-worming and promotion of hygiene practice. (38, 39).

Nevertheless, malnutrition is also a critical factor affecting brain development. Numerous studies indicate that inadequate and appropriate nutrition during critical windows of brain development cannot later be addressed by provision of those nutrients that had earlier in development been missing or in suboptimal supply. This early abnormal brain development results in impaired cognitive function, school achievement, and behaviour leading to long term neuropsychological sequelae (40). In the critical window between birth to age 3 years we are however without a valid tool for objectively measuring executive function and emotional regulation, those two aspects of cognition upon which so much later development and performance depends. Recent developments have indicated that electrophysiological approaches using technologies such as EEGs, fNIRS, are able to assess brain function objectively in a clinical-research setting. and have been proven safe to use even in young children and infants providing accurate measures of brain activity, clear spatial and temporal imaging data, translation into diagnostic tools for developmental and neuro-behavioural disorders (e.g., autism) and intervention efficacy analysis. In addition, various behavioural tests to see EF/ER as a functional outcome have been created to gauge cognitive development and also play a key role in tracking of cognitive progress and symptoms of neurodevelopmental syndromes. These processes at early age are crucial to facilitate social interaction and social-emotional development.

There have been several studies in Bangladesh that have attempted to use special feeds in the rehabilitation of malnutrition. However, to date none have been able to evaluate neurocognition with high accuracy in the period one to three years. In this present proposal, members of the consortium will develop such a toolkit and validate it; in our study we will deploy the toolkit being developed to determine how weld it functions in a developing country setting and how well it measures EF and ER as well as their response to interventions. Our overall goals in Bangladesh are to explore EF and

ER development in low-income settings and see the effect of MAM on EF and ER as well as to pilot a nutritional intervention designed to improve brain health. Data obtained from this pilot study will be helpful to design a full trial of nutritional intervention and brain health in malnourished children using the final commissioned toolkit.

Research Design and Methods

Describe the research design and methods and procedures to be used in achieving the specific aims of the research project. If applicable, mention the type of personal protective equipment (PPE), use of aerosol confinement, and the need for the use BSL2 or BSL3 laboratory for different part of the intended research in the methods.. Define the study population with inclusion and exclusion criteria, the sampling design, list the important outcome and exposure variables, describe the data collection methods/tools, and include any follow-up plans if applicable. Justify the scientific validity of the methodological approach (biomedical, social, gender, or environmental).

Also, discuss the limitations and difficulties of the proposed procedures and sufficiently justify the use of them.

Study Site:

The study will be conducted in Mirpur area within the Dhaka city (DNCC wards ward 2, 3 and 5). Mirpur study/surveillance area is well known to all staffs working in our Mirpur field clinic as they have been working in this area for our existing studies for the last 10 years. We have established a field clinic/lab located within the ward 5 where the staffs have been working for the BEAN project (PR-14110 and PR-18036) for the last 7 years. Existing staffs will therefore recruit the children and mothers from the study area. The Mirpur area is densely populated area and is located around 8 km from the main campus of icddr, b at Mohakhali, Dhaka. Mirpur is selected as the study site because it is inhabited by poor and middle-class families, residential and sanitary conditions are typical of any congested urban settlements, and we have ongoing research activities in this area for the last 30 years. The site has a typical squatter settlement; the average family size of households is 4.5, with 48% females. About 20% of households have a monthly income of only US$62, 30% of mothers never attended school, and only 3% obtained secondary school education. The majority of the people are day labourers, garment workers, and transport workers. Mirpur has a population of about half a million in an area of 14.22 km^2. More than 38,000 people live in each square kilometre of the area compared with the mean of 8229/km^2 in Dhaka district and 976/km^2 in Bangladesh (41)

Study design

This will be a community-based clinical trial study (Figure 1). Survey, screening, enrolment of subjects will be conducted in Mirpur under the Dhaka North City Corporation. Eligible children and mother (pairs) will be enrolled from wards 2,3 and 5 of Mirpur area. We will recruit 70 one-year old adequately nourished children WHZ >-1 SD they will not receive any intervention, and 140 one-year old MAM children i.e. WHZ <-2 and ≥-3 z-score, and/or MUAC <12.5 and ≥11.5 cm with their mothers. These 140 children will be randomized to 1:1. One group will receive locally produced RUSF, approximate at 50-100 kcal/kg/day, two of 50g packets daily (42) until anthropometric recovery (WHZ > - 1SD) has been achieved or for maximum 3 months then immediately 1 packet / day SQLNS will be given till end of 2 years of follow up and other group will receive the E-RUTF at 50-100 kcal/kg/day which in this age group approximates one 92 g sachet daily until anthropometric recovery (WHZ > - 1SD) has been achieved or for maximum 3 months then immediately E-SQLNS 1 packet daily provided throughout the study till the end of 2 years of follow-up. We will also recruit 70 3year-olds previously untreated MAM children WHZ <-2 and ≥-3 z-score, and/or MUAC <12.5 and ≥11.5 cm as an outcome reference group for a singular assessment. All children (both case & control) will undergo a baseline nutritional, medical, biological and neuro psychological assessment (EF, ER, EEG and fNIRS). At enrolment all children will have anthropometric measurement and biological (blood and stool) sample collections - buccal sample collection for genetics and epigenetics, as well as blood for neutronics and metabolomics and stool sample for microbiome assessment. After completion of baseline sample and data collection, all children will receive RUSF, approximate at 50-100 kcal/kg/day, two of 50g packets daily for two months. However, the biological samples will be preserved for future use for the

secondary objectives and later will be sent to University of Auckland where the tests for microbiome, genetics and epigenetics will be performed. icddr,b will not perform testing of any biological samples due to constraints of funds and expertise. After enrolment for first one month our study staffs will go to the participant's household daily to ensure intake of our study food. They will teach and councel the mothers about administration and importance of our study food. They will also councel mothers not to share the food with the other children in the household and also ask questions regarding compliance of the intervention diet e.g taste of the food, any adverse event (vomiting, diarrheoa etc) related with eating the diet, total number of packets consumed each day by the child, if not consumed the whole dose they will also record the reason and collect the empty sachets each day.We will develop a set of questionnaire regaring the compliance of the intervention diet to be filled of bty the study staffs. After one month, when the mothers are well councelled with our study procedures, we will deliver complementary foods every week to the mothers. Then study staff will visit the household twice per week to closely monitor the child's feeding status and document feeds intake and morbidity events. Every week study staff will refill the intervention dietary supply and deliver to the home. Bangladesh standard of care psychosocial stimulation will be given to all 140 children who have enrolled with MAM. All 210 children will be followed up to 3 years of age with periodic neuro developmental assessment (EF, ER, EEG, FNIRS), biological samples collection as well as clinical assessment at years 2 and 3.

Fig 1: Flow Chart

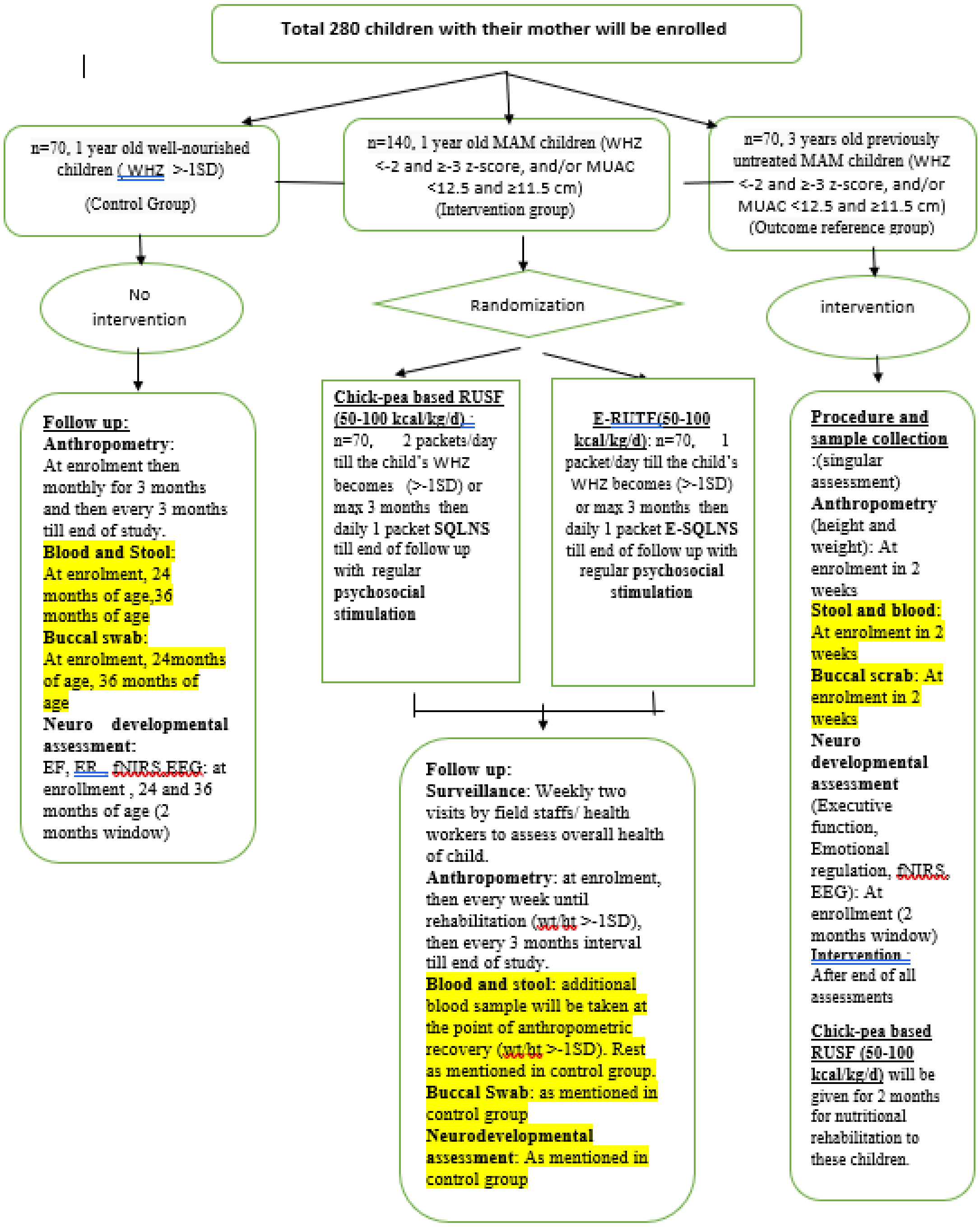

Eligibility Criteria:

- **Inclusion criteria for malnourished (MAM) children at 1 and 3 years of age:**
  All of the following criteria must be met for a subject to be eligible to participate in the study-
  - Mother willing to sign consent form
  - Child age 12-15 months or 36-39 months of age
  - WHZ <-2 and ≥-3 z-score, and/or MUAC <12.5 and ≥11.5 cm and free from any acute illness
  - Mother will agree to feed study diet to their children at home.

  - Mother willing to bring the child to the clinic for assessment (morbidity, nutrition history, adherence to treatment, anthropometry and advice to mother on parenting) and clinical examination.
  - Mother will agree to provide her biological samples
  - Mother willing to have child undergo biological sample collection and neuropsychological assessment at baseline (1-year-old) and at ages 2 years and 3 years at clinic.
  - Family has no plan to move from study area in next three years.
- **Exclusion criteria for malnourished (MAM) children:**
  Meeting any of the following criteria will exclude a subject from study participation –
  - Mother who will not willing to sign consent form
  - Congenital anomaly
  - Mother not willing to feed the rehabilitation feed or the small quantity supplement to her child
  - Family will not stay 3 years in study area.

Selection criteria for control groups:

- **Inclusion criteria for Control group with normal health (normal Z score):**
  - Mother willing to sign consent form
  - Child age 12-15 months
  - WHZ score > -1 and free from any acute illness
  - Mother willing to bring the child to the clinic for assessment (morbidity, nutrition history, adherence to treatment, anthropometry and advice to mother on parenting) and clinical examination.
  - Mother will agree to provide her biological samples (see below)
  - Mother willing to have child undergo biological sample collection and neuropsychological assessment at baseline at 1 year old and again at 2 years and 3 years at clinic.
  - Family has no plan to move from study area in next two years.

- **Exclusion criteria for Control group with normal health (normal Z score)**
  Meeting any of the following criteria will exclude a subject from study participation –
  - Mother will not willing to sign consent form
  - Child age >15 months or < 12m
  - Mother not willing to feed the rehabilitation feed or the small quantity supplement to her child
  - Family will not stay 3 years in study area.
  - Any congenital anomaly

- **Subject Withdrawal or Termination from the Study:**

  Participants may withdraw voluntarily from the study at any time without penalty or consequence.

If any of the following events occur, the child will be withdrawn from the study:

1. The research is terminated by the funding organization or the Principal Investigator(s) or by any regulatory authority.
2. The child's mother withdraws consent for the child to participate in the study.
3. The child's family moves out of the surveillance area.
4. If mother wishes to enroll her child to another clinical trial.
5. Condition of child health is not good for continuing the intervention determined by investigators or study physician

Screening, Consenting and Enrolment:

Our study staff will use a door-to-door survey to identify potential children based on the eligibility criteria. Mothers of the eligible children will be invited to be enrolled in to the study after they have had the opportunity to have a detailed review of the study objectives, procedures, and potential risks and benefits with her. Study staff will answer all of the questions related to the study from the mother. A Child is considered enrolled if mother provides her signature/ left thumb print on the IRB approved consent from. Total duration of the period of enrollment will be 24 weeks

Randomization: Total 140 malnourished children who enrolled at 1 year of age with the criteria of WHZ <-2 and ≥-3 z-score, and/or MUAC <12.5 and ≥11.5 cm (Moderate acute malnutrition) from the study area (Mirpur Ward 2, Ward 3 and Ward 5) will be randomized 1:1 to receive complementary foods either RUSF followed by SQLNS or E RUTF followed by E-SQLNS till the end of follow-up for 2 years.

Interventions:

1. Locally produced ready to use supplimentery food (RUSF), 50 g/packet contains 204 kcal energy). Two packets of RUSF provided for consumption at a rate of 50-100 kcal/kg/day till the child's weight for height returns to normal (WHZ >-1SD) or for maximum 3 months. The RUSF packets will be produced in a food processing laboratory to be established at the Mirpur field site and the food ingredients will be locally available and culturally acceptable complementary food. The overall procedure will be guided by an expert clinical nutritionist who has the experience of producing these RUSF in other studies (29). This is expected on average to take 12 weeks. Once the child's appetite returns from the high intake drive during rapid catch up to normal intake (this occurs when child reaches ideal wt/ht) children will then be provided with daily SQLNS to the end of 2 years follow-up period.
2. Enhanced Ready to use therapeutic feeds (E-RUTF), 50-100 kcal/kg/d daily until for anthropometric recovery (WHZ > - 1SD) is achieved or for maximum 3 months then E-SQLNS will be given till the end of 2 years follow-up.
   Children who will fail to reach desired anthropometric levels even after completing the nutrition intervention package for 3 months, will be refered to nutritional rehabilitation-based facility for further evaluation and management to exclude any secondary cause of malnutrition specially Tuberculosis.But the child will remain in the study and we will continue to follow up to close monitoring the child and take necessary measures with an 'Intention to Treat."

3. Psychosocial stimulation will be given to 140 children who have enrolled with MAM

Intervention scheme:

Group	Number	Intervention
1. Well-nourished children at 1 year (WLZ/WHZ score >-1 SD)	70	No nutritional and psychosocial intervention<br>Only follow-up
2. With WHZ <-2 and ≥-3 z-score, and/or MUAC <12.5 and ≥11.5 cm having moderate acute malnutrition (MAM at 1 year)	70	50-100 kcal/kg/day RUSF daily till ideal wt/ht(WHZ >-1SD) achieved or for maximum 3 months and then SQLNS will be started for up to the end of the study period<br>Psychosocial stimulation
3. With WHZ <-2 and ≥-3 z-score, and/or MUAC <12.5 and ≥11.5 cm having moderate acute malnutrition (MAM at 1 year)	70	50-100 kcal/kg/d Enhanced RUTF till ideal wt/ht(WHZ >-1SD) achieved or for maximum 3 months and then E-SQLNS will be started for up to the end of the study period.<br>Psychosocial stimulation
4. With WHZ <-2 and ≥-3 z-score, and/or MUAC <12.5 and ≥11.5 cm having stable moderate acute malnutrition (MAM) at 3 years	70	After completing the baseline assessment, we will provide them daily RUSF for 2 months and then refer to NRU at icddr,b or local facilities if needed.

Complementary food supplementation:
After randomization of 140 children at 1 year (12-15 months) of age with moderate acute malnourished child, one group of 70 children will receive RUSF 50-100 kcal/kg/d till anthropometric recovery (WHZ > - 1SD) or for maximum 3 months and then SQLNS daily till the end of 2 years of follow up. The other group of 70 children will receive Enhanced RUTF at 50-100 kcal/kg/d till anthropometric recovery (WHZ > - 1SD) or for maximum 3 months and then E-SQLNS daily till the end of 2 years of follow up. Mother will learn from our study staffs how to feed this complementary food to her child properly. Mother will receive 7 days feed/supplement for her child at a time. Initially our study staff will ensure daily feeding by visiting the household daily, interview and councel the mother, fill up the compliance forms and collect the empty sachets daily for first 1 month, then our study staff will visit the household twice per week to ensure the feeding by interviewing mother and collecting the empty packets of intervention. Study staff will capture the data on CRF and also document any event associated with the intervention. Study staff will also measure weight, height and MUAC weekly to track recovery from wasting. Our study staff will ensure the availability of feeds every week before finishing existing packets. This process will continue till end of the study

Psychosocial Stimulation:
All nutritional invention group of malnourished children will receive a set psychosocial stimulation curriculum “REACH-UP”, that has been shown to be effective on severely malnourished children

with therapeutic feedings at icddr,b nutritional rehabilitation unit. The Reach Up curriculum addresses all the key components of the "nurturing care framework" to promote optimum development (43). This psychosocial stimulation package will also follow a set of culturally appropriate, semi-structured, child's age-appropriate curriculum. During the clinic and home visits health-workers will show the mothers how to play with home-made toys and books and interact with their children in a way to promote their development (Figure 2). Toys will be given to the child to play and learn until next visits, when a new set of developmentally appropriate toys will be provided. Mothers will also be encouraged to come up with new ideas of making appropriate toys for the child from recycle materials available at home. Care will be taken to appreciate mothers for their activities and positive reinforcement will be strongly encouraged. The developmental activities will be conducted in a playful manner and not as a work-oriented activity. The activities in the curriculum will be ordered by difficulty level and the health workers will be trained to choose the level for each child according to their ability to do the activities

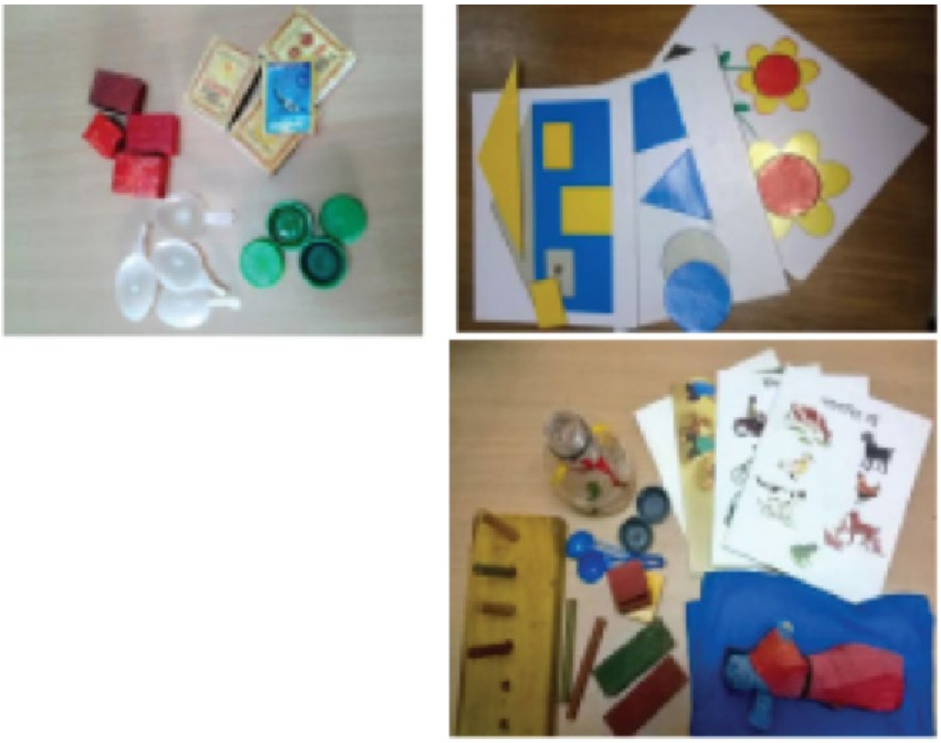

Figure 2: Homemade toys to provide psychosocial stimulation for young children

<u>STUDY PROCEDURES:</u>

<u>Enrolment visit (at 1 year of age):</u>

<u>Base line assessment for children (Plus two months window)</u>

- Obtain consent
- Anthropometry: weight, height, MUAC and head circumference
- Blood draw
- Stool collection
- Buccal scrab
- EF
- ER

- EEG
- fNIRS

<u>Baseline assessment of mother for all enrolled children (single assessment):</u>

- Anthropometry: weight and height
- One stool sample
- One buccal swab sample
- 5 ml blood

- Randomization of MAM child
- Start of intervention of malnourished child within one week of completion of baseline assessment.

Interim anthropometric measurement: Anthropometric measurement (weight, height and MUAC) will be taken by field staff weekly during the initial rehabilitation from wasting and continue till the anthropometric recovery (WHZ > - 1SD) of the child has achieved or for maximum 3 months. Thereafter children will have anthropometry in every 3 months for the remainder of the study from 140 children who have enrolled with MAM to track their nutritional status. Head circumference will be measured at enrolment, at the age of 2 and 3 years to correlate with the developmental measures
But 70 well-nourished children will have monthly anthropometry (weight, height and MUAC) for initial 3 months and then in every 3 months interval till end of the study who have been enrolled with WHZ score >-1 SD at 1 year. Head circumference will be measured at enrolment, at the age of 2 years and 3 years

Interim biological sample collection: One additional blood and stool sample will be collected at the time of anthropometric recovery and before starting SQLNS or E-SQLNS intervention in both groups.

<u>Visit at 24 months and 36 months after enrollment (Two weeks window)</u>

- Anthropometry: weight, height, MUAC and Head circumference
- Stool collection
- Blood
- EF
- ER
- EEG
- fNIRS

<u>Data collection from the children:</u>
The study will collect age, sex, DOB, demography and SES from family, medical history, feeding history at the time of enrolment.

Anthropometric data: At enrolment, weekly during rehabilitation to achieved anthropometric recovery (WHZ > - 1SD) then quarterly for the duration of the study (till 36months of age) from 140 children who have enrolled with MAM. Head circumference will be measured at enrolment, at the age of 2 years and 3 years.

Anthropometric data will also be collected from 70 normal children at enrolment, then monthly for 3 months, then quarterly for the duration of the study (till 36months of age). Head circumference will be measured at enrolment, at the age of 2 years and 3 years.
Psychosocial stimulation will be given to all 140 MAM children

Neuro developmental assessment:
EF, ER, EEG, f NIRS data will be collected at enrollment and then at 24 and 36 months of age from 210 children.

Biological sample collection from the children:

Blood: 2-3 ml of child blood will be collected at enrolment, at the time of achieved ideal wt/ht (anthropometry recovery) or at the end of 3 months of E-RUTF/RUSF intervention , 24 months and 36 months of age to perform concentration of 24 vitamins and mineral micro nutrients, functional lipid concentrations in red cell membrane, serum siallylated milk oligo saccharides, Lutein/ zeaxantnine / cryptoxanthine and Microbiome directed blood metabolome

Stool collection: Stool sample will be collected at enrolment, at the time of achieved ideal wt/ht (anthropometry recovery), or at the end of 3 months of E-RUTF/RUSF intervention, 24 months and 36 months of age to perform 16S microbial sequencing, Functional pathway analysis and stool metabolome

Buccal swab:
Buccal mucosa will be collected at enrolment, 24 and 36 months of age for genomics/epigenomics studies

Primary health care and Referral of the participants:
The study clinic at Mirpur is equipped by qualified study physicians. The Clinic is open 8.30 am to 5.00 pm 6 days a week. Study physicians will responsible for providing primary health care responding to presentations such as fever, diarrhoea, vomiting for all study participants and their mother. Drugs for this primary care will be provided by the project. However, study physicians will make appropriate referrals in case of severe illness. The study will not cover the referral cost.

Neuropsychological assessment:
Toolkit assessment of executive function and emotional regulation:
Using our toolkit and outcome measures our overall goals in these two related activities are to explore EF/ER development in low-income settings and the effect of moderate or severe acute malnutrition on short- and long-term EF/ER sequelae and pilot a nutritional intervention designed to improve brain health.

This design will adequately assess the capabilities of the EF/ER Toolkit in the control group allowing comparisons to high-income data from Boston, Singapore and New Zealand. Through the two interventional arms, and the comparison to 3y old untreated children, we will have high quality pilot data on the EF/ER response to interventions in order to power a definitive trial. We will follow up the children at 2 years and 3 years with EF/ER for developmental assessment.

Behavioural Measures (Executive Functions/Emotional regulation):
Executive functions (EF) refer to the processes involved in conscious control of thought and action (44, 45), and include inhibitory control, planning and cognitive flexibility. Emotional Regulation

(ER) is an integral part of self-regulation, which is a complex concept that regulates emotions, motivation, cognition (e.g., attention), social interactions, and physical behaviour (46).

These higher cognitive functions such as planning ability, cognitive flexibility, working memory, and cognitive and emotional control develop within the second year of life, and continue to develop through mid-late adolescence. These functions are regulated by the prefrontal cortex region of the brain, which continues to develop through mid-late adolescence, explaining why executive functions also reach maturation around that time as well. Moderate or severe acute malnutrition impair both EF and ER and here are currently no nutritional rehabilitation strategies to correct this malnutrition induced abnormalities. The provision of RUSF and SQLNS or Enhanced RUTF and Enhanced SQLNS in the project is meant to provide the preliminary data to power and refine the design of a definitive intervention trial to determine the effectiveness of this intervention in reversing the executive dysfunction and emotional dysregulation sequelae of malnutrition.

For the toolkit/test battery, we will pre-test several previously used tests on children to finalize a number of tests for the main study. EF tests are mainly based on cognitive flexibility, inhibitory control, emotional regulation and working memory of participants. Most of these are direct interactions between the experimenter and the child, which can be recorded on tablet or in video camera for video-scoring.

The neurophysiological antecedents and predictors of executive function emerging from the development of the pre-frontal cortex antedates the fill emergence for executive function at approximately 2 years of age. Our measurements are designed to capture these at 1 year of age as well as in the following 2- and 3-year assessments. The applicable tests options for this age group are –

- For Cognitive flexibility- Reverse Categorization test, Leaf/Banana, Fruit Stroop, A not B task/Hide-the-Pots,
- For Inhibition/inhibitory control: Chicken/Tiger, Tower Test, Snack delay, Gift delay
- For working memory- Picture memory, Zoo locations
- For emotional regulation: Theory of mind test

Brief description of some of the tests--

'Reverse Categorization' test, children are taught a similar sorting rule with blocks (little blocks in little bucket, big blocks in big bucket), but then asked to reverse the rule and sort the opposite way (little blocks in big bucket, etc.).
In the **'*Banana/Leaf task***, children are asked to match words to the opposite colour they represent (e.g. point to a yellow card when hearing '*Leaf* and point to a *green* card when hearing 'banana).
In ***A not B task/Hide-the-Pots*** test the tester presents the child with a board with two shallow wells, one on the right and one on the left. Then s/he hides a treat in one of the wells and covers both wells with opaque cups so that the child cannot see the treat. After distracting the child with a song for 5 seconds, the tester prompts the child to find the treat. The treat is hidden in the same place until the child searches in the correct place on two consecutive trials, after which the treat is hidden in the opposite well. Ten trials are given. The total score represents the number of trials (out of 10) in which the child searched in the correct place for the snack.

In the '***chicken/tiger task***, children will be asked to follow instructions of the 'nice chicken, but ignore instructions of the 'mean tiger.

In the ***Tower Test***, the child is invited to help the tester build a tower with wooden blocks. The child is scored in her/his ability to take turns with the tester. [For this one we need to set a constant

number of blocks and trials (make sure to record it, for example 8-10 blocks and two trials). Record the number of times the child respect turns with the tester].

In ***Snack Delay (younger children),*** the tester places a snack treat (e.g. cookie or cracker) under an inverted transparent cup in front of the child. Then s/he instructs child to wait for the bell to ring before retrieving the snack treat. S/he does these four times, with increasing time delays for each trial, s/he records the time the child waits before retrieving the snack and scores the average time the child waited across trials

In ***Gift Delay (younger children),*** the tester shows the child a large shopping bag containing a shiny wrapped gift box. She draws the child attention towards the bag and keep the child waiting with the bag to record his/her self-control about not to open the gift box. Total time is calculated.

The 2-year-olds will participate in tasks more appropriate to their age, including: categorization, A not B/hide-the-pots, snack delay, fruit strop, gift delay, and the tower test.

The 3-year-olds will participate the categorization and A-not-B task with the Banana/*Leaf*, chicken/tiger tasks, along with the theory of mind scale. These tasks are being administered in studies in our Boston lab, and are widely used and accepted for this particular age range

Note: All the above tests have been used in Bangladesh previously on different age groups of children around 3 years. The protocols for the tasks are included below, but we do plan to adapt the specific stimuli to be more culturally appropriate with the help of Dr. Fahmida Tofail at icddr,b. Previous studies have successfully used culturally adapted versions of these tasks.

NIH toolbox: Alternatively, we can explore NIH Toolbox for EF/ER tests, if that is feasible and applicable on these young (2-3 years) malnourished children

EEG

The EEG (Figure 3) will be used to calculate resting state functional connectivity, and event related potentials (ERPs) will be used to probe the neural correlates of specific cognitive functions. ERPs measure changes in electrical activity of the brain immediately in response to a direct stimulus event. In addition, sophisticated signal processing/machine learning approaches can be applied to examine frequency information (e.g., theta activity, phase amplitude coupling), which could serve as indices of local circuit connectivity.

The EEG will be recorded using a NetAmps 300 amplifier system, a 128-channel HydroCel Geodesic Sensor Net (HCGSN, Electrical Geodesics Inc., Eugene, OR) and data acquisition software (NetStation 4.5, EGI). We plan to use a system identical to what is currently being used in Dr. Nelson's laboratory in Boston. The HCGSNis an elastic net consisting of non-invasive electrodes with small plastic pedestals each containing small sponges with Ag/AgCl electrodes. When the net is donned, the pedestals are evenly distributed across the surface of the head. These nets are sized according to head circumference of the infants to avoid potential pain or discomfort. The EEG works by detecting small charges emitted by the brain during activity, amplifying the signals, and graphing signals in real time. An example of an infant being tested with this system is depicted in Figure 1.

. For the 2-year-old population, ERPs will be recorded in response to a deferred imitation task, which measures memory. Children will be shown a sequence of events using specially designed props, and asked to recognize the familiar sequences.

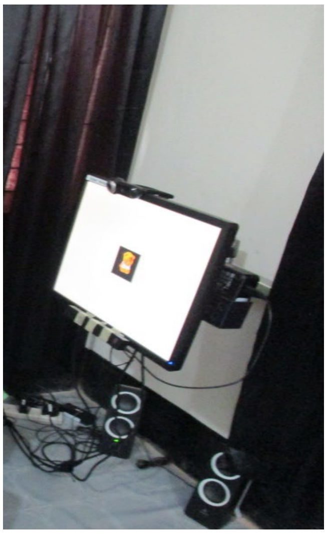

Figure 3: EEG test in Mirpur lab

Specific Cognitive Skills Assessment (Eye tracking)
A TOBII eye-tracking system will be used to track the infant's eye movements during cognitive tasks. A small infrared camera mounted to a computer monitor will track eye movements once calibrated. Eye tracking will be used to test distractibility, or disengagement. Infants will be presented with an interesting visual stimulus, and will be tested on how they disengage their attention from that stimulus. Stimuli and procedure are adapted from Elsabbagh et al (47), and have been validated in other low-income settings.

fNIRS
Brain activity will be assessed using fNIRS (Figure 4), a method that measures changes in blood oxygen concentration as a consequence of neural activity in the brain. fNIRS can be used to measure brain activity that is related to an externally presented stimulus, or it can measure the connections between brain activity in different parts of the brain with no explicit external stimulus presentation. Measures of hemodynamics reported by fNIRS include relative concentration changes in oxy-hemoglobin, deoxy-hemoglobin and total hemoglobin. The fNIRS measurement is completely non-invasive and has been used in infant studies of brain activity for over 15 years.

The fNIRS will be performed using the NTS system, designed and built at the Biomedical Optics Research Laboratory at University College London (UCL). This system has been designed to meet the two relevant international standards: IEC 60601 (for medical electrical equipment), and IEC 60825 (for safety of laser products). A series of tests performed by University College Hospital's Medical Physics department technicians further ensures compliance with international standards. Exposure to laser diode sources fall in accordance with standard IEC 60825 for both eye and skin exposure. This particular device has been used to image brain activity in over 300 healthy infants in London, as well as an additional 100 infants in the Gambia (48). The fNIRS system is being used for basic physiological research, and is not intended for use in the diagnosis of disease and other conditions, or in the cure, mitigation, treatment or prevention of disease This study is not being conducted to determine safety or effectiveness of the fNIRS system, no data will be used in the clinical care of patients. Please note that the PI of this project has been using NIRS in his BCH laboratory for the past 6+ years, in a variety of populations of infants and toddlers

The system consists of a headband with attached light optodes emitters and detectors. Images are received by shining a near- infrared laser light source through fiber optics onto the head. A photo

detector is then used to sample the variations in light reflected back from the brain. The headband will be placed around the infant's head from the forehead area to the back of the head, resting above the ear area. The headband will be secured at the back of the head with Velcro. An example of an infant being tested with this system.

The fNIRS will measure hemodynamic change in response to different stimuli conditions with reference to baseline. Procedure and stimuli are the same Lloyd Fox, et al., and have been tested in 300 healthy infants in London, as well as 100 infants in the Gambia (47). A statictical learning paradigm will also be administered in the 2-year cohort. Children will be shown a baseline image/video on the screen while listening to various auditory tones.

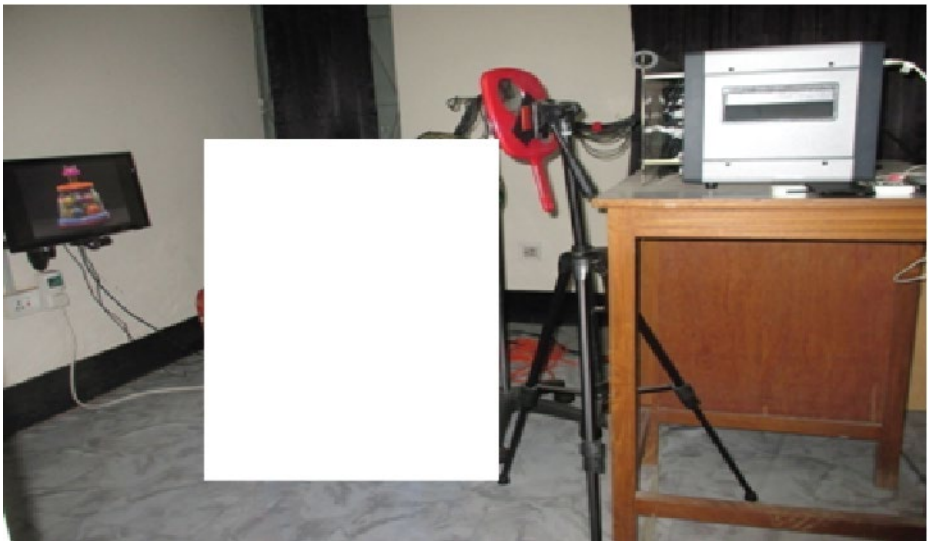

Figure 4: fNIRS test in Mirpur

COVID-19 safety concern:

Responsibilities of all staff who will directly communicate with participants during Face-to-face interviews and physical contact during test in Covid 19 situation.

This will include during field visits for census, recruitment, neuro imaging testing , behavioural assessment, bio sample collection and anthropometry.
No staff member may conduct face-to-face interviews with physical contact who has not received the appropriate bio safety training.

- The appropriate level of PPE will be worn throughout the interview and field visit.
- Participants will wear face masks.
- PPE will be appropriately disposed of in a designated place, where disposal is appropriate.
- PPE that will be re-used will be appropriately managed according to the re-use protocols.
- PPE will be changed between participants
- Where anthropometry occurs, equipment to be reused, such as stadiometers or weighing bags must either be cleaned between participants, or there must be one item available per participant (e.g., one weighing bag for each infant, which are washed at the end of the day)

- If any member of a household has COVID-19 like symptoms then we will avoid that household which will be confirmed by phone call before visit the household.
- Everyone has been trained on maintaining maximum possible distance from each other while assessing patients and sanitizing all logistics such as weight machine, measuring tape, blood pressure measurement equipment etc. after each use.
- Where biological samples are collected, single use items will be disposed of appropriately, multiple use items will only be used once until appropriate cleaning/disinfecting/autoclaving can be arranged.

Sample Size Calculation and Outcome (Primary and Secondary) Variable(s)

Clearly mention your assumptions. List the power and precision desired. Describe the optimal conditions to attain the sample size. Justify the sample size that is deemed sufficient to achieve the specific aims.

For the comparison with the well-nourished children (WHZ > -1 SD) with wasted children at 1 and 3 year (WHZ <-2 and ≥-3 z-score, and/or MUAC <12.5 and ≥11.5 cm) of the WHO child growth standards) the two propositions of sample size 70 participants in each group would suffices for conducting the two major types of analyses proposed in the present study, that is, those pertaining to between two groups differences in means using observed or latent variables. Thus, power has been estimated for Student's t-test and Structural Equation Modeling (SEM).

For a t-test, the proposed medium level of difference between two groups (i.e.,. 2 standard deviation) required 51 participants per group for recommended power equal to 80% using a level of significance equal to 5% (for a one-tailed test). For the evaluation of brain functioning using EF, EEG or fMRI, Sideridis et al. 2014 (49), using a series of montecarlo studies, reported that between 50-70 participants would suffice to evaluate functional connectivity in the brain. Their simulation study involved medium sized models of 7 latent variables but for larger models (as the ones expected in the present study) the model size is inversely related with sample size. That is, larger models require fewer participants.

The current protocol has two control groups and two intervention groups. Because we expect some data loss during the follow-up period of 3 groups if we take a sample size of 70 children in each group, we will have enough power to see within group differences. So, we will enrol 280 children total in this study.

Data Analysis

Describe plans for data analysis, including stratification by sex, gender and diversity. Indicate whether data will be analysed by the investigators themselves or by other professionals. Specify what statistical software packages will be used and if the study is blinded, when the code will be opened. For clinical trials, indicate if interim data analysis will be required to determine further course of the study.

Statistical tests will be conducted to quantify differences in various neurocognitive tests such as EF,NIRS and EEG responses between our wasted vs. well-nourished groups and within groups of children with varied levels of exposure to adversities (single adversities, composite exposure scores, and income).Two types of models will be employed, (a) a t-test for analysing univariate outcomes, and, (b) structural equation modeling to analyse multivariate outcomes. With regard to (a) the t-test will be run at an alpha level of 5% and the distributions of the two samples will be checked for normality, homogeneity of variances and autocorrelation in the residual terms. If necessary, the False Discovery Rate (FDR) method will be applied to correct for potential multiple comparisons.

For the repeated measurements of neurocognitive tests, EF, NIRS, and EEG responses at 12 ,24 and 36 months, the linear mixed effects model will be considered to characterize and evaluate their longitudinal effects with respect to the group differences and the relationship with other earlier biological and/or psychosocial risk factors, particularly focusing on the brain power and functional

change in development. Since these measurements are repeated at three time points, the mixed mdoel with either random intercept or random slope will be considered.
With regard to (b) structural equation modeling will be employed to evaluate multivariate relationships. Model fit will be evaluated using descriptive fit indices such as the Comparative Fit Index (CFI) which has been found to behave properly with relatively small sample sizes (Bentler et al,1990 (50).

Data Safety Monitoring Plan (DSMP)

All clinical investigations (research protocols testing biomedical and/or behavioural intervention(s)) should include the Data and Safety Monitoring Plan (DSMP). The purpose of DSMP is to provide a framework for appropriate oversight and monitoring of the conduct of clinical trials to ensure the safety of participants and the validity and integrity of the data. It involves involvement of all investigators in periodic assessments of data quality and timeliness, participant recruitment, accrual and retention, participant risk versus benefit, performance of trial sites, and other factors that can affect study outcome.

A formal Data and Safety Monitoring Board (DSMB) will be convened for this study at the icddr,b. The study investigators will report to the DSMB per the Board's requirements and expectations. The PIs will assume responsibility for ensuring adherence to Good Clinical Practice.

The staff collecting data will be extensively trained in the use of all recording techniques with human participants of all ages. Quality control will be ensured by regularly maintaining equipment and data will be processed as it is collected to allow the early identification of potential problems with data quality. All reportable events will be reviewed, and appropriate action will be taken immediately if needed.

Data Management and Security

Data will be stored on the Synapse Research Platform (synapse.org; WIRB#20112068), a collaborative data science environment that enables researchers to seamlessly and transparently conduct, track and share their ongoing work – building up living research projects in real time. The Synapse research environment is protected by data privacy and security controls standard in healthcare information technology, including the provisions required by HIPAA and GDPR. Synapse is designed as a collaboration tool, allowing sharing of data products, analyses, and research methods, while maintaining access control and providing tools for governance oversight. Within Synapse, data are stored in projects. Sharing settings of projects are managed separately by the owner of each project.

The Synapse Platform is developed and administered by Sage Bionetworks. The Information Security Program at Sage Bionetworks is based on the ISO 27000 series of Information Security Management System standards and is supported by NIST special publications. These frameworks establish a baseline of comprehensive security control objectives and processes for reviewing, testing, and updating them. Sage is pursuing HITRUST certification of platform systems to demonstrate assurance of the security program.

Amazon Web Services provides the base layer for Synapse Storage. AWS is the world-wide leader in cloud computing and provides security measures at the infrastructure level. Within this framework, Sage maintains compliance programs for HIPAA, GDPR, and other privacy and security standards designed to safeguard protected health information. The Synapse web service is maintained by a team of systems administrators, directly employed by Sage Bionetworks. The use

of administrator accounts are controlled and monitored but can be used to access data for maintenance of the system. Data is encrypted at the storage and network layers, and so cannot be accessed by any subcontract infrastructure provider. File and database storage supporting the application are encrypted with AES-256. Transmissions through Synapse interfaces are encrypted with SSL/TLS, enforced by technical policy.

Activity in Synapse is logged by user and time of an action. User activity is reviewed quarterly to identify risks to data privacy. Objects in Synapse can be flagged by users for review by Sage's Governance Team for consistency with the terms of use of the associated data (i.e. data user restrictions), whether due to consent, privacy, or security violation.

Ethical Assurance for Protection of Human rights

Describe the justifications for conducting this research in human participants. If the study needs observations on sick individuals, provide sufficient reasons for using them. Indicate how participants' rights will be protected, and if there would be benefit or risk to each participants of the study. Discuss the ethical issues related to biomedical and social research for employing special procedures, such as invasive procedures in sick children, use of isotopes or any other hazardous materials, or social questionnaires relating to individual privacy. Discuss procedures safeguarding participants from injuries resulting from study procedures and/or interventions, whether physical, financial or social in nature. [Please see Guidelines]

This study will be conducted in compliance with the Declaration of Helsinki and in full conformity with the principles of the Belmont Report: Ethical Principles and Guidelines for the Protection of Human Subjects of Research of the National Commission for the Protection of Human Subjects of Biomedical and Behavioral Research (April 18, 1979) and codified in 45 CFR 46, 21 CFR 312, and/or ICH E6; 62 Federal Regulations 25691 (1997). Each participating institution will hold a current FWA issued by OHRP. All key study staff will be trained in Good Clinical Practice.

Each participating institution's review board will review and approve this protocol and associated informed consent documents. Any amendments to the protocol or consent materials will also be approved before they are implemented.

Informed consent will be obtained from individuals prior to performance of study-specific procedures. Because the primary study population is not capable of assenting due to age (1 to 3 years old), parental permission will suffice as informed consent for study participants.

Participant confidentiality is strictly held in trust by the participating investigators, their staff, and the sponsor and their agents. This confidentiality includes documentation, investigation data, participant's clinical information, and all other information generated during participation in the study.

Risks to confidentiality will be minimized. Each study participant will be assigned a unique study identification number (SID) to be used on study forms and in the study database. Neither the study forms nor the study database will contain participant's names or other information that could be used to identify them. The document linking the SID to patients' name and medical record numbers will be kept in a locked office and will not be accessible to personnel not associated with the study. Study forms will be maintained in a locked office and will not be available to personnel not associated with the study. All computers containing the study database will be password-protected and the study database will not be accessible by personnel not associated with the study.

One potential risk is fatigue from the neuroimaging test. In order to minimize this risk, we have decided to split the battery of tests into two sessions, with options for breaks and snacks within sessions as needed.

All neuroimaging/behavioural procedures are non-invasive, pain free, and contain no adverse effects. Devices have been adapted to the infant population to ensure safety and comfort, and every measure proposed for use in this project is currently being used in Crypto and PROVIDE Cohort in Mirpur. All of these measures have been performed repeatedly in various studies worldwide with no adverse effects seen in participants. Infants will be constantly monitored, and as part of the protocol, if infants become fussy and fidgety, the experimenter will pause the session. If the infant is too fussy to be placated, the sensors will be removed to end the session.

Use of Animals

Describe if and the type and species of animals to be used in the study. Justify with reasons the use of particular animal species in the research and the compliance of the animal ethical guidelines for conducting the proposed procedures.

None

Collaborative Arrangements

Describe if this study involves any scientific, administrative, fiscal, or programmatic arrangements with other national or international organizations or individuals. Indicate the nature and extent of collaboration and include a letter of agreement between the applicant or his/her organization and the collaborating organization.

This study is conducted with a collaboration of icddr,b , University of Auckland, New Zealand, Boston Children's Hospital , USA and UWI Solutions fro Developing Countries, The University of the West Indies, , Jamaica. Dr. Rashidul Haque is the local principal investigator at icddr, b . This project is funded by Welcome Leap program through a subcontract from the University of Auckland and the work of the project will be carried out in close collaboration with Prof. Nelson, Boston Children Hospital and Prof. Terrence Foster, University of West Indies, Jamica.

Dr. Rashidul Haque's clinic at Mirpur will be used for screening, enrolment, sample collection, data collection, Physical examination, provide intervention and neurocognitive testing.

Professor Nelson and his team at Boston Children's Hospital will collaborate with icddr,b (Local -PI Dr. Rashidul Haque to use his Dhaka facilities to test fNIRS, EEG , EF /ER to see the neurocognitive outcome of this study.

Professor Terrence Forrester and his team at UWI Solutions for Developing Countries (UWI SODECO) will collaborate with Dr. Haque and his Teamto execute the project throughout, preliminary phases of this study at Mirpur, as well as at the baseline and intervention phases.

Facilities Available

Describe the availability of physical facilities at site of conduction of the study. If applicable, describe the use of Biosafety Level 2 and/or 3 laboratory facilities. For clinical and laboratory-based studies, indicate the provision of hospital and other types of adequate patient care and laboratory support services. Identify the laboratory facilities and major equipment that will be required for the study. For field studies, describe the field area including its size, population, and means of communications plus field management plans specifying gender considerations for community and for research team members.

The International Centre for Diarrhoeal Disease Research, Bangladesh (icddr,b) has large multi-disciplinary international and national scientific research staff. Existing field, hospital, laboratory and office facilities will be used for this study. icddr,b scientists have conducted a variety of vaccine studies (Polio, influenza, pneumococcal, cholera, Shigella, rotavirus, Japanese encephalitis etc.).

The study will be conducted in urban site of Mirpur. icddr,b . The field site clinic in Mirpur is located 8 km north west from icddr,b main campus. The clinic has approximately 2400 SFT includes office and clinic spaces, storage capacity, and appropriate facilities for conducting the study.
Two rooms already been used in Mirpur to set up the neuroimaging equipment. Major equipment to be shipped to Bangladesh are: fNIRS system, EEG system, Tobii eye tracking system, one stimulus presentation laptop, 4 computer monitors, speakers, two video cameras, one PC, one articulating arm. All Hardware/software is already provided by BCH. Another room will be used for cognitive/ executive function test

Other rooms will be used for screening, consenting, sample collection, physical exam etc.

<u>Laboratory Facilities:</u>
icddr,b: Existing laboratory facilities of icddr,b under the Infectious Disease Division will be used to process the samples. Laboratory facilities of the Boston Children Hospital and the University of AuckLand will also be used for this study.

Literature Cited

Identify all cited references to published literature in the text by number in parentheses. List all cited references sequentially as they appear in the text. For unpublished references, provide complete information in the text and do not include them in the list of Literature Cited. There is no page limit for this section, however, exercise judgment in assessing the "standard" length.

1. Bhutta, Z. A. *et al.* (2017) 'Severe childhood malnutrition', *Nature Rview Disease Primers*, 1767(3).
2. Black RE, Allen LH, Bhutta ZA, Caulfield LE, de Onis M, Ezzati M, Mathers C, Rivera J. Maternal and child undernutrition: global and regional exposures and health consequences. Lancet 2008;371:243–60.
3. Collins S, Dent N, Binns P, Bahwere P, Sadler K, Hallam A. Management of severe acute malnutrition in children.Lancet 2006;368:1992–2000.
4. Magnani, Rich; Oot, Lesley; Sethuraman, Kavita; Kabir, Golam; Rahman, Setara. 2015. USAID Office of Food for Peace Food Security Country Framework for Bangladesh FY 2015–2019. Washington, DC: FHI 360/FANTA.
5. National Institute of Population Research and Training (NIPORT), Mitra and Associates, and ICF International. 2016. Bangladesh Demographic and Health Survey 2014. Dhaka, Bangladesh, and Rockville, Maryland, USA: NIPORT, Mitra and Associates, and ICF International.
6. Bank, W., Goal, M. D. and Division, G. E. (2018) 'Bangladesh : Nutrition Profile', (February), pp. 1–8.
7. Prado, E. L. and Dewey, K. G. (2014) 'Nutrition and brain development in early life', *Nutrition Reviews*, 72(4), pp. 267–284. doi: 10.1111/nure.12102.
8. Mora JO, Herrera MG, Sellers SG, Ortiz N. Nutrition, social environment and cognitive performance of disadvantaged Colombian children at three years. Nutrition in Health and Disease and International Development. Symposia from the XII International Congress of Nutrition. New York: Alan R. Liss, 1981:403-430.
9. Super CM, Herrera MG, Mora JO. Long-term effects of food supplementation and psychosocial intervention on the physical growth of Colombian infants at risk of malnutrition. Child Development 1990;61:1-49.

10. Herrera MG, Mora JO, Christiansen N, Osriz N, Clement J, Vuori L, Waber D, De Paredes B, Wagner M. Effects of nutritional supplementation and early education on physical and cognitive development. In: Turner RR, ed. Lifespan psychology: Intervention. New York: Academic Press, 1980:149-184.
11. Adair LS, POllitt E. Outcome of maternal nutritional supplementation: a comprehensive review of the Bacon Chow study. Am J Clin Nutr 1985;41:948-978.
12. SchalschaT.Ines, Mayers, G. L. and van Oss, C. J. (1998) 'MALNUTRITION, BRAIN DEVEMPMENT, LEARNING, AND BEHAVIOR', *Encyclopedia of Immunology*, 18(1), pp. 430–439.
13. Sinisterra L, McKay A, McKay H, Gomez H, Korgi J. Response of malnourished preschool children to multidisciplinary intervention. In: Brozek J, ed. Malnutrition and human behavior. New York: Van Nostrand Reinhold, 1985:317-326.
14. McKay H, Sin&terra L, McKay A, Gomez H, Lloreda P. Improving cognitive ability in chronically deprived children. Science 1978;200:270-278.
15. Blanton, L. V. *et al.* (2016) 'Gut bacteria that prevent growth impairments transmitted by microbiota from malnourished children', *Science*, 351(6275). doi: 10.1126/science.aad3311.
16. Cerdó, T., Diéguez, E. and Campoy, C. (2019) 'Early nutrition and gut microbiome: interrelationship between bacterial metabolism, immune system, brain structure, and neurodevelopment', *American journal of physiology. Endocrinology and metabolism*, 317(4), pp. E617–E630. doi: 10.1152/ajpendo.00188.2019.
17. Gunston, G. D. *et al.* (1992) 'Reversible cerebral shrinkage in kwashiorkor: An MRI study', *Archives of Disease in Childhood*, 67(8), pp. 1030–1032. doi: 10.1136/adc.67.8.1030.

18. Gordon, J. I. *et al.* (2012) 'The human gut microbiota and undernutrition', *Science Translational Medicine*, 4(137), pp. 1–7. doi: 10.1126/scitranslmed.3004347.
19. Million, M., Diallo, A. and Raoult, D. (2017) 'Gut microbiota and malnutrition', *Microbial Pathogenesis*, 106, pp. 127–138. doi: 10.1016/j.micpath.2016.02.003.
20. Fox, S. E., Levitt, P. and Nelson, C. A. (2011) 'How the Timing and Quality of Early Experiences Influence the Development of Brain ArchitectureFox, S. E., Levitt, P., & Nelson, C. A. (2011). How the Timing and Quality of Early Experiences Influence the Development of Brain Architecture. Child Developme', *Child development*, 81(1), pp. 28–40. doi: 10.1111/j.1467-8624.2009.01380.
21. Mackie, R.I., Dodd, D., Hong, P., Zhang, M. and Cann, I.K.O., 2013. Conserved Molecular Mechanisms Used to Degrade Heteroxylans in Gut Bacteroidetes. *Microbial Ecology in Health & Disease*, *24*.
22. Charbonneau, M.R., O'Donnell, D., Blanton, L.V., Totten, S.M., Davis, J.C., Barratt, M.J., Cheng, J., Guruge, J., Talcott, M., Bain, J.R. and Muehlbauer, M.J., 2016. Sialylated milk oligosaccharides promote microbiota-dependent growth in models of infant undernutrition. *Cell*, *164*(5), pp.859-871.
23. Cusick, S.E. and Georgieff, M.K., 2016. The role of nutrition in brain development: the golden opportunity of the "first 1000 days". *The Journal of pediatrics*, *175*, pp.16-21.
24. Obelitz-Ryom, K., Bering, S.B., Overgaard, S.H., Eskildsen, S.F., Ringgaard, S., Olesen, J.L., Skovgaard, K., Pankratova, S., Wang, B., Brunse, A. and Heckmann, A.B., 2019. Bovine milk oligosaccharides with sialyllactose improves cognition in preterm pigs. *Nutrients*, *11*(6), p.1335.
25. Choudhury, N. *et al.* (2014) 'Community-based management of acute malnutrition in Bangladesh: Feasibility and constraints', *Food and Nutrition Bulletin*, 35(2), pp. 277–285. doi: 10.1177/156482651403500214.
26. World Health Organization. Management of severe malnutrition: a manual for physicians and other senior health workers. Geneva: WHO, 1999.

27. Puett C, Coates J, Alderman H, Sadler K. Quality of care for severe acute malnutrition delivered by community health workers in southern Bangladesh. Matern Child Nutr 2013;9:130–42.
28. Sadler K, Puett C, Mothabbir G, Myatt M. Community case management of severe acute malnutrition in southern Bangladesh. Boston, Mass, USA: Tufts University,2011
29. Ahmed, T. *et al.* (2014) 'Development and acceptability testing of ready-to-use supplementary food made from locally available food ingredients in Bangladesh', *BMC Pediatrics*, 14(1), pp. 1–8. doi: 10.1186/1471-2431-14-164.

30. Manary MJ. Local production and provision of ready-to-use therapeutic food (RUTF) spread for the treatment of severe childhood malnutrition. Food Nutr Bull. 2006 Sep;27(3 Suppl):S83-9. doi: 10.1177/15648265060273S305. PMID: 17076214
31. Tripp, K., Perrine, C.G., de Campos, P., Knieriemen, M., Hartz, R., Ali, F., Jefferds, M.E.D. and Kupka, R., 2011. Formative research for the development of a market-based home fortification programme for young children in Niger. *Maternal & child nutrition*, *7*, pp.82-95.
32. Paul, K.H., Muti, M., Chasekwa, B., Mbuya, M.N., Madzima, R.C., Humphrey, J.H. and Stoltzfus, R.J., 2012. Complementary feeding messages that target cultural barriers enhance both the use of lipid-based nutrient supplements and underlying feeding practices to improve infant diets in rural Zimbabwe. *Maternal & child nutrition*, *8*(2), pp.225-238.
33. Phuka, J., Ashorn, U., Ashorn, P., Zeilani, M., Cheung, Y.B., Dewey, K.G., Manary, M. and Maleta, K., 2011. Acceptability of three novel lipid-based nutrient supplements among Malawian infants and their caregivers. *Maternal & child nutrition*, *7*(4), pp.368-377.
34. Na, M., Aguayo, V.M., Arimond, M. and Stewart, C.P., 2017. Risk factors of poor complementary feeding practices in Pakistani children aged 6–23 months: A multilevel analysis of the Demographic and Health Survey 2012–2013. *Maternal & Child Nutrition*, *13*, p.e12463.
35. Wilson, B. and Whelan, K., 2017. Prebiotic inulin-type fructans and galacto-oligosaccharides: definition, specificity, function, and application in gastrointestinal disorders. *Journal of gastroenterology and hepatology*, *32*, pp.64-68.
36. Healey, G., Murphy, R., Butts, C., Brough, L., Whelan, K. and Coad, J., 2018. Habitual dietary fibre intake influences gut microbiota response to an inulin-type fructan prebiotic: a randomised, double-blind, placebo-controlled, cross-over, human intervention study. *British Journal of Nutrition*, *119*(2), pp.176-189.
37. Meléndez-Martínez, A.J., Mandić, A.I., Bantis, F., Böhm, V., Borge, G.I.A., Brnčić, M., Bysted, A., Cano, M.P., Dias, M.G., Elgersma, A. and Fikselová, M., 2020. A comprehensive review on carotenoids in foods and feeds: Status quo, applications, patents, and research needs. *Critical reviews in food science and nutrition*, pp.1-51.
38. Nasim, M. *et al.* (2017) 'National Guidelines for the Facility based Management of Children with Severe Acute Malnutrition in Bangladesh', *Institute of Public Health Nutrition Directorate General of Health Services Ministry of Health and Family Welfare Goverment of the People's Republic of Bangladesh*, (July).
39. Devi, C. D. S., Ramesan, T. and Nath, G. (1985) 'Technical note', *International Journal of Heat and Mass Transfer*, 28(10), pp. 1960–1963. doi: 10.1016/0017-9310(85)90220-0.
40. Grantham-McGregor S. A review of studies of the effect of severe malnutrition on mental development. The Journal of nutrition. 1995 Aug;125(8 Suppl):2233S-8S.
41. Ahmed T, Mahfuz M, Islam MM, Mondal D, Hossain MI, Ahmed AS, et al. The MAL-ED cohort study in Mirpur, Bangladesh. Clinical Infectious Diseases. 2014;59(4):280-S6.
42. Robert Y. Chen, Ishita Mostafa, et al. A Microbiota-Directed Food Intervention for Undernourished Children. N Engl J Med 2021;384:1517-28. DOI: 10.1056/NEJMoa2023294

43. Nahar, B., Hamadani, J., Ahmed, T. *et al.* Effects of psychosocial stimulation on growth and development of severely malnourished children in a nutrition unit in Bangladesh. *Eur J Clin Nutr* **63,** 725–731 (2009). https://doi.org/10.1038/ejcn.2008.44

44. Baddeley, A. (1996). Exploring the central executive. Quarterly Journal of Experimental Psychology: Human Experimental Psychology (SpecialIssue: Working Memory), 49A, 5–28
45. Perner, J., & Lang, B. (1999). Development of theory of mind and cognitive control. Trends in Cognitive Science, 3, 337–344.
46. Karoly, P. (1993). Mechanisms of self-regulation: A systems view. Annual Review of Psychology,44, 23–52
47. Meek JH, Firbank M, Elwell CE, Atkinson J, Braddick O, Wyatt JS. Regional hemodynamic responses to visual stimulation in awake infants. Pediatric research. 1998 Jun;43(6):840-3**.**
48. Lloyd-Fox S, Papademetriou M, Darboe MK, Everdell NL, Wegmuller R, Prentice AM, et al. Functional near infrared spectroscopy (fNIRS) to assess cognitive function in infants in rural Africa. Scientific reports. 2014;4:4740.
49. Sideridis, G. D., Simos, P., Papanicolaou, A., & Fletcher, J. (2014). On the use of SEM for evaluating functional connectivity in the brain: Sample size considerations Educational and Psychological Measurement, 74, 733-758.
50. Bentler, P M. (1990). Comparative fit indexes in structural models. *Psychological Bulletin, 107,* 238-246.

Budget [Please add]

Budget Justifications

Please provide one page statement justifying the budgeted amount for each major item, including the use of human resources, major equipment, and laboratory services.

ICDDR,B
Budget Justification

Other Support

Describe sources, amount, duration, and grant number of all other research funding currently granted to PI or under consideration.

Information Sheet for mother with her 1-year-old child

Protocol No. PR-21084	Version No. 1.00	Date: 18 July 2021

Protocol Title: Multidimensional evaluation of the early emergence of executive function and emotional regulation in young children in Bangladesh using nutritional and psychosocial intervention: A Pilot study

Investigator's name: Dr. Rashidul Haque

Organization: International Centre for Diarrhoeal Disease Research, Bangladesh (icddr,b)

Purpose of the research: To determine the effect of nutritional intervention for improvement of cognition and emotional regulation among the children in Bangladesh where malnutrition and social adversities are common

Background (brief introduction of the issue and the need for/ importance of the research)
We are conducting a study to understand the problem of malnutrition and poor cognitive outcomes in children of Bangladesh.Malnutrition affects around 47 million children under 5 years of age in low- and middle-income countries annually and among then more than 20 million death occurs, others suffer long term cognitive and behavioural impairment. Malnutrition causing significant number of deaths of under 5 years child globally. It is a large problem in Bangladesh where 40% of under-fives have moderately acute malnutrition. Malnutrition is further worsened by poor diet. Inadequate feeding practices leading to deficiencies in vitamin and minerals. Studies show that, during infancy and early childhood, nutrition is essential as these are the crucial period for the formation of the brain, building the foundation for the development of cognitive, neurological and socio-emotional skills throughout childhood and adulthood.

Why invited to participate in the study?
Researchers from icddr,b, the University of Auckland, New Zealand, Boston Children Hospital, USA and Tropical Medicine Research Institute, Jamaica are jointly conducting this research study in your community to understand the effect of nutritional intervention on the cognition of moderate acute malnourished chidren . Total 210 children and 210 mother will be enrolled from this community. We invite you to help us in our efforts through your child's participation in this study, because you have 1 year of old child and you live in this community where malnutrition and social adversities are common. Research ethics committees at icddr,b and IRBs of University of Auckland, New Zealand, Boston Children Hospital, USA and Tropical Medicine Research Institute, Jamaica have approved this research study.

Methods and procedures [What is expected from the participants of the research study?]
You and your child will be requested to participate in the study. If you agree to participate; you are agreeing to provide information about you and your child's medical and personal information as well as, to allow the collection of a stool, blood sample, buccal scrab and body measurement such as height, weight from your child and blood, stool, buccal swab and body measurement such as height, weight from you as per protocol. Your child is also invited to participate Executive function, Emotional regulation, fNIRS and EEG. In addition, study will provide nutritional supplementation based on randomization scheme, your child will receive RUSF followed by SQLNS or E- RUTF followed by E- SQLNS and Psychosocial stimulation to your child if your child is enrolled with WHZ <-2 and ≥-3 z-score, and/or MUAC <12.5 and ≥11.5 cm having moderate acute malnutrition. After obtaining the signed consent from you we will start the study activities. However, no nutritional supplementation or psychosocial stimulation will be given to your child if your child is well nourished i.e WHZ score >-1. We will follow up your child up to 3yrs of age and perform the above-mentioned tests according to protocol.

Screening and enrolment:

Screening and enrolment will occur at the home / clinic by our trained study team. The team will review the eligibility criteria to confirm your child is eligible. If your child is eligible then team will explain the study to you and request to participate. If you sign this consent form for you and for your child then both of you will be enrolled in the study and we will collect details such as birth date, sex, information about your family (occupation, income, education, family size, length of time of breast feeding etc). We will also collect medical information, if it is needed. This enrolment procedure will take approximately 1 hour. Your child will be randomly assigned any of nutritional intervention if your child is enrolled with WHZ <-2 and ≥-3 z-score, and/or MUAC <12.5 and ≥11.5 cm having moderate acute malnutrition. Randomization and interventions are not applicable who are enrolled with WHZ score > -1 SD

We will provide you the contact information and location of local clinic to visit our clinic and our staff will start the surveillance for intervention.

Procedures and sample collection :

- Stool collection for all child: At enrolment, , and then 24 and 36 months of age (+/-7 days window)
- Additional stool collection only for MAM child: At the time of weight for height returns to normal (>-1SD) or at the end of 3 months of E-RUTF/RUSF intervention (+/-7 days window)
- Blood (2-3 ml) collection: At enrolment, then 24 and 36 months of age (+/-7 days window)
- Additional blood collection only for MAM child: At the time of weight for height returns to normal (>-1SD) or at the end of 3 months of E-RUTF/RUSF intervention (+/-7 days window)

- Anthropometry (height, weight, MUAC)):

  For MAM child: At enrolment, then weekly until weight for height returns to normal (>-1SD) and then every 3 months interval for entire duration of the study (+/-7 days window).
  For well-nourished child: at enrolment, then monthly for 3 months, then every 3 months interval for entire duration of the study (+/- 7 days window).

  Head circumference for all children will be measured at enrolment, at 24 and 36 months of age
- Buccal Scrab: At the time of enrolment, 24 months and 36 months of age (+7 days window)
- Neuro development assessment (EF, ER, fNIRS, EEG): At enrollment then 24 and 36 months of age (2 months window)

Nutritional intervention:

After randomization of 1:1 who are enrolled with WHZ <-2 and ≥-3 z-score, and/or MUAC <12.5 and ≥11.5 cm having MAM: One group of 70 children will receive locally produced Ready to use supplimentery food (RUSF, 50 g/packet contains 204 kcal energy): Two packets of RUSF daily till the child's weight for height returns to normal (>-1SD) or for maximum 3 months and then be provided with 20 g daily SQLNS (contain 118 kcal energy) till end of the study.

And 70 children of other group will receive Enhanced Ready to use therapeutic feeds (E-RUTF), 50-100 kcal/kg/d daily until the child's weight for height returns to normal (>-1SD) or for maximum 3 months and then to be given 26g/day E-SQLNS (contain 130 k cal energy) till the end of the study.

Psychosocial Stimulation:

During the clinic and home visits health-workers will show the mothers how to play with home-made toys and books and interact with their children in a way to promote their development. Toys will be given to the child to play and learn until next visits, when a new set of developmentally appropriate

toys will be provided. All activities will be conducted in a playful manner and not as a work-oriented activity.

Behavioural Measures (Executive Functions/Emotional regulation):

Executive functions are the processes involved in conscious control of thought and action including inhibitory control, planning and cognitive flexibility. Emotional Regulation is an integral part of self-regulation, which is a complex concept that regulates emotions, motivation, attention, social interactions, and physical behaviour

This study involves two 2-3-hour sessions at the icddr,b Mirpur clinic for Neuro developmental assessment (EF, ER, fNIRS, EEG) . The sessions will be scheduled on separate days within two weeks of each other at a time that is convenient for you and your child . Most of tasks of executive function and emotional regulation are direct interactions between the experimenter and the child, which will be recorded in video camera for video-scoring. These activities will be conducted in a playful manner and not as a work-oriented activity.

NIRS Procedure:

During one of sessions, we will record your child's brain activity using functional near-infrared spectroscopy (fNIRS). fNIRS measures and records the changes in the levels of oxygen in the blood by shining a near-infrared light into your child's head. We will be able to identify which areas of your child's brain are actively responding to the changes in images shown on the computer monitor.

For recording these changes, we use a computer which is attached to sensors and probes. Prior to placing the probes, we will measure your child's head with a measuring tape for proper fitting of the fNIRS sensors. These sensors rest on a headband, which is then placed around your child's head and adjusted for a tight fit.

While your child is wearing the headband, we will your child to watch videos of women either moving their eyes left or right, or performing the 'peekaboo' or 'itsy bitsy spider' hand games, or images of transportation vehicles. There will also be sounds playing during some of the images/videos.

Finally, we will ask many different questions about your child's development (motor skills, speech, vision, etc.) in order to test for developmental disabilities. All information provided will be confidential, and if anything is concerning, further testing will be conducted, and referrals for care will be made free of charge. All of these procedures are completely safe and will not hurt your child. You will be with your child at all times.

EEG Procedure:

During the other visit to the clinic to perform EEG, we will record your child's brain activity using a small cap that is made of stretchy material. Each cap has many sponges on it and inside each sponge is a small recording sensor. We soak the caps in a warm salt water solution so the sponges get soft before we put the cap on the child's head. In this task, also before starting the session we need to measure the head circumference by measuring tape to get the appropriate cap size. As your brain is working, it is constantly giving off small electrical signals, which travel out to the scalp where we can pick them up with the special sensors. We will show a series of faces and patterns and record your child's brain activity during these tasks. Then, we will do the eye tracking measures. Your child will sit on your lap and watch a video of bright looming circles while we set up the eye-tracking equipment. The eye tracker is made up of a special computer monitor that has a set of infrared cameras built into the edges of the screen. Once calibrated, these cameras will follow eye movements and tell us exactly

where on the screen your child is looking as he/she is watching the pictures. At your first visit we will have your child look at videos of moving infant toys for six minutes while we record your child's brain activity. Next, we will record your child's eye movements in response to a series of pictures on a computer screen. The child will see pictures of faces and scenic backgrounds for one task, and cartoon clown/suns/balloons. In addition, a digital video will be recorded to help the experimenter know when to present pictures to your child and aid data analysis. Your child's name will not be associated with the video recording and the file will be accessible only to the investigators of this study.

Home visit: For 1st one month Our study staff will visit your home daily to collect the data of complementary food consumption, review empty packets of complementary food and also collect the history of febrile illness, vomiting and diarrhoea. Then they will visit your home twice weekly and do the aforementioned procedures. They will also refer the child to the field clinic for attending clinic visit as well as primary care support by the study physician

None of neuro developmental e tasks are invasive or harmful

Procedures and sample collection for you (mother):

- Stool collection: 10 gm once within 2 weeks of enrolment
- Blood collection: 5 ml once within 2 weeks of enrolment
- Buccal swab sample: Once within 2 weeks of enrolment
- Anthropometry (height and weight): Once within 2 weeks of enrolment

Risk and benefits

What are the risks from participating in the study?

Sometimes things happen to the research participants in research studies that may hurt them or make them feel bad. These are called risks. The risks of participating in this study include risks caused by intervention, neuro cognitive assessments and sample collection.

Intervention: There was no major adverse effects reported in earlier study, however we will collect all event related to the intervention.

Blood collection: Mild pain, discomfort, bleeding or bruising, or get an infection (which is extremely rare) where the needle or lancet is inserted. To minimize these risks, only trained, experienced staff will draw blood, and disposable materials will be used. if an infection results from a blood draw, we will provide any necessary treatment at no cost to you

Stool collection: We do not anticipate any risks from collecting stool.

Buccal swab: We do not anticipate any risks from collecting buccal swab

Neuro cognitive assessment: There are no major risks involved with those assessments. The number of tests during each day may cause some tiredness, but you and your children are allowed to take as many breaks as you need. All tests and procedures remain outside the child's body, pain free, and contain no bad effects.

If anything from the tests seems unusual or concerning (like abnormal seizure activity on EEG), child will be referred to the appropriate specialists in Bangladesh, and you will be contacted with information on what to do next. The study will cover the cost of transportation to the specialist,

assistance with the consultation and referral process, but we will not cover the cost of medical care beyond primary medical care.

Benefit

You and Your child will receive free, high-quality primary care, and referrals for any illness through our study for the duration of participation. Additionally, there may be some benefit from taking study food.

Children who will fail to reach desired anthropometric levels even after completing the nutrition intervention package for 3 months, will be referred to nutritional rehabilitation-based facility for further evaluation and management to exclude any secondary cause of malnutrition specially Tuberculosis. But the child will remain in the study and we will continue to follow up to closely

Principle of compensation

There is no direct compensation for participation in this study, but in the case of any concerning information discovered, referrals to professionals will be provided free of charge. In addition, transportation to the clinic and food at the clinic will be provided.

Privacy, anonymity and confidentiality

We will keep all information collected from you and your child confidential and locked in a secure place under the responsibility of the study investigators. Data will be saved in secure servers at icddr, b, Boston Children's Hospital and . Biological samples will be stored securely without identifying information at icddr, b. Neuro imaging testing will be done in private rooms, and subjects will be coded by a de-identified number. In addition, staff and researchers have completed the Course in The Protection of Human Research Subjects.

In general, anyone who is involved in this research, including those funding and regulating the study, may see the data, including information about you and your child, photos and videos. For example, the following people might see information about you and your child:

- Research staff at icddr,b
- Research staff at Boston Children's Hospital
- Research staff at Auckland University
- Research staff at Tropical Medicine Research Institute, Jamaica

Your/your child's name and identity will not be disclosed in the process of analyzing, presenting or publishing the results of these procedures.

If you sign this form, you have given us permission to release information to authorized researchers and the safety committees, icddr,b Ethical Review Committee, regulatory authorities (both in Bangladesh and the United States), the study sponsor , Synopse (a research data sharing and collaboration platform) and designees, and other research organizations. There is no expiration date to this permission. If you decide to withdraw your permission and end this agreement, please contact Dr. Rashidul Haque at the address/number listed below. He or his staff will help you document in writing your decision to withdraw this permission. Please note that any study information already obtained will continue to be used.

Your participation in this research is voluntary. However, you will not be able to participate in this study if you do not sign this form.

Future use of samples:

At the end of the study, all of the specimens will be stored at icddr,b for 5 years . If you agree, we will store these samples to be used in the future for other research purposes. If such research is conducted by us or by our collaborators, appropriate approvals from respective authorities will be secured at that time. If samples be used in the future, your and your child's privacy and anonymity will be maintained. If you consent to having your and your child's samples saved to be used for future research, but change your mind later, you may contact us and the samples will be destroyed. If not, we will store your child's specimens for 5 years. Of note, samples may be sent to collaborators outside of Bangladesh for specialized testing.

Future use of information

Information about you and your child may be shared with regulatory authorities including but not limited to the Ethical Review Committee (ERC) at the icddr,b, the Internal Review Board (IRB) at University of Auckland, Boston children hospital, Tropical Medicine Research Institute, Jamaica and the study sponsor and designees. Investigators may choose to share information and data with other researchers at their discretion for the purpose of future research.

Right not to participate and withdraw

Participation in this study is voluntary and you can choose to not participate, or withdraw at any point during the study without any penalty or loss of care.

Consent Sheet for mother with her 1-year-old child

SID:

**Protocol No. PR-21084**	**Version No. 1.00**	**Date: 18 July 2021**

Protocol Title: Multidimensional evaluation of the early emergence of executive function and emotional regulation in young children in Bangladesh using nutritional and psychosocial intervention: A Pilot study

Investigator's name: Dr. Rashidul Haque

Organization: International Centre for Diarrhoeal Disease Research, Bangladesh (icddr,b)

If you agree to our proposal for enrolling you and your child in our study, please put √ mark on appropriate box(es) of the following and finally sign / left thumb print on the specified place for you:

**Points**	**Status**
I have read out / study staff has read out the all information from this participants information sheet Version 1.0, Dated 18 July 2021 about the study, have had the opportunity to ask questions, discuss the study, and received satisfactory answers	Yes ☐ No ☐
I understood that I am free to leave the study without giving any reason	Yes ☐ No ☐
I understood that the information that I gave will be confidential	Yes ☐ No ☐
I agree to allow the study team to collect information from me and my child at every scheduled follow up visit and home visit	Yes ☐ No ☐
I understood that the Information from this research study will be retained by icddr,b , Boston Children Hospital, USA, Auckland University, New Zealand and Tropical Medicine Research Institute, Jamaica, Synopse (a research data sharing and collaboration platform) and in the future may be included in a de-identified public use database. De-identified means that I and my child will not be individually identified by name or other personal identifiers in the database. My full name or any address details will not be included. Information released will not identify me or my child's participation in this research study.<br>I am giving permission for those individuals to have access to my records.	Yes ☐ No ☐
I agree to the collection of blood (2-3 ml), buccal swab, stool from my child	Yes ☐ No ☐
I agree to the collection of blood (5 ml), buccal swab and stool sample from me	Yes ☐ No ☐
I agree that anonymised blood, buccal swab and stool (those collected from me and my child) samples can be sent overseas for analysis	Yes ☐ No ☐
I agree to feed nutritional intervention and phycological stimulation to my child (If my child is malnourished)	Yes ☐ No ☐
I agree to perform neurocognitive tests (Executive Function, Emotional Regulation, fNIRS, EEG) to my child	Yes ☐ No ☐
I agree to storage and future use of me and my child's data and samples by ethically approved studies	Yes ☐ No ☐
I agree to being contacted in the future for studies related to this study	Yes ☐ No ☐
I understand that relevant sections of me and my child's medical notes and data collected during the study may be looked at by individuals from the	Yes ☐ No ☐

sponsor and by regulatory authorities, where it is relevant to my taking part in this research. I give my permission for those individuals to have access to my records.	
I agree to participate in to this study	Yes ☐ No ☐

______________________________ ________________

Signature or left thumb impression of participant Date (dd/mmm/yyyy)

______________________________ ________________

Signature or left thumb impression of Date (dd/mmm/yyyy)
Parent/ Guardian/ Attendant

______________________________ ________________

Signature of the witness Date (dd/mmm/yyyy)

______________________________ ________________

Signature of the PI or his/her representative Date (dd/mmm/yyyy)

Communication:
If you have any question, you can ask me right now or at any time later to the below mentioned personnel:

Purpose of contact	Name and address	Address for communication
For any question related to the study, or any problem	Dr. Masud Alam	Address: House 28, Avenue 1, Kalsi Road, Mirpur-12, Dhaka-1216<br>Mobile No. 01711570550<br>(to be open 7/24 hours)
	Name of PI: Dr. Rashidul Haque.	Address: Parasitology Laboratory, IDD, icddr,b , Mohakhali, Dhaka-1212<br>Mobile: 01713093859<br>(9:00 am to 5:00 pm)
To know the rights or benefits or to log any complain or dissatisfaction	M A Salam Khan (IRB Coordinator)	IRB Secretariat, Research Administration, icddr,b, Mohakhali, Dhaka-1212<br>Phone: (+88-02) 9827084 or Mobile: 01711428989

Thank you for your cooperation.
A Copy of singed consent will be given to you.

মা ও তার ১ বছর বয়সী শিশুর জন্য গবেষণা সম্পর্কিত তথ্য

**Protocol No. PR-21084**	**Version No. 1.00**	**Date: 18 July 2021**

Protocol Title: Multidimensional evaluation of the early emergence of executive function and emotional regulation in young children in Bangladesh using nutritional and psychosocial intervention: A Pilot study

Investigator's name: Dr. Rashidul Haque

Organization: International Centre for Diarrhoeal Disease Research, Bangladesh (icddr,b)

গবেষণার উদ্দেশ্য

এই গবেষণার উদ্দেশ্য হলো বাংলাদেশের শিশুদের বুদ্ধির বিকাশ এবং আবেগীয় নিয়ন্ত্রণ উন্নয়নের জন্য পুষ্টি খাওয়ানোর প্রভাব নির্ধারণ করা যেখানে অপুষ্টি এবং সামাজিক প্রতিকুলতা বিদ্যমান]

ভুমিকা 'সমস্যার সংক্ষিপ্ত ভুমিকা এবং এই গবেষণার গুরুত্ব . প্রয়োজনীয়তা(

নিম্ন ও মধ্যআয়ের দেশগুলোর মধ্যে পাঁচ বৎসরের নীচে শিশুদের মধ্যে সাড়ে চার কোটিরও বেশী শিশু প্রতিবছর অপুষ্টির শিকার হয়] এদের মধ্যে ২ কোটি শিশু মারা যায় এবং অন্যরা দীর্ঘ মেয়াদী বুদ্ধির বিকাশগত সমস্যায় ভুগে] সারা বিশ্বে অপুষ্টির কারনে ৫ বৎসরের নীচে শিশুদের মধ্যে উল্লেখযোগ্য সংখ্যক শিশু মারা যায়] এই অপুষ্টি বাংলাদেশের একটি বড় সমস্যা যেখানে পাঁচ বৎসরের নীচের ৪০$ শিশু মাঝারি অপুষ্টিতে ভোগে] এই অপুষ্টি আরও খারাপ হয় যখন খাবারে ঘাটতি হয়] স্বল্প খাদ্য গ্রহনের অভ্যাসের কারনে শরীরে ভিটামিন ও খনিজ লবনের ঘাটতি দেয়া হয়] গবেষণায় দেখা গেছে যে+ শৈশবকাল ও শৈশবকালীন সময়ে পুষ্টি গ্রহন অপরিহার্য কারন মস্তিষ্ক গঠনের জন্য ইহা গুরুত্বপূর্ণ সময়কাল] বুদ্ধির বিকাশ+ শৈশব ও কৈশোরকালে স্নায়বিক এবং সামাজিক,মানসিক দক্ষতার ভিত্তি তৈরি করার জন্য পুষ্টি অপরিহার্য]

কেন গবেষণায় অংশগ্রহণের আমন্ত্রন জানানো হচ্ছে>

আইসিডিডিআরবি+ অকল্যান্ড ইউনিভার্সিটি নিউজিল্যান্ড+ বোস্টন চিলড্রেনস হসপিটাল আমেরিকা এবং ট্রপিকাল মেডিসিন রিসার্চ ইন্সটিটিউট+ জ্যামাইকার গবেষকবৃন্দ আপনাদের এলাকায় মাঝারি অপুষ্টির শিশুদের বুদ্ধির বিকাশের উপর পুষ্টি খাওয়ানোর প্রভাব দেখার জন্য যৌথভাবে এই গবেষণাটি পরিচালনা করছেন] আপনাদের এলাকার সর্বমোট ২১০ জন শিশু এবং ২১০ মায়েদের এই গবেষণায় অন্তর্ভুক্ত করা হবে] আপনার শিশুকে এই গবেষণায় অংশগ্রহণের জন্য আমরা আপনাকে আমন্ত্রন জানাচ্ছি কারন আপনি ১ বছর বয়সী শিশুর অভিভাবক এবং এই এলাকায় বসবাস করছেন যেখানে অপুষ্টি এবং বিভিন্ন সামাজিক প্রতিকুলতা বিদ্যমান] আইসিডিডিআরবি র গবেষণা নীতি কমিটি এবং ইউনিভার্সিটি অফ অকল্যান্ড+ নিউজিল্যান্ড র আইআরবি+ বোস্টন চিলড্রেনস হসপিটাল আমেরিকা এবং ট্রপিকাল মেডিসিন রিসার্চ ইন্সটিটিউট+ জ্যামাইকা এই গবেষণাটির অনুমোদন দিয়েছেন]

পদ্ধতি ও প্রক্রিয়া 'গবেষণায় অংশগ্রহনকারীদের কাছ থেকে কি প্রত্যাশা>(

আপনি এবং আপনার শিশুকে এই গবেষণায় অংশগ্রহনের জন্য অনুরোধ জানাচ্ছি] আপনি যদি অংশগ্রহনে রাজি থাকেন+তাহলে+আপনি এবং আপনার শিশুর স্বাস্থ্য এবং ব্যক্তিগত তথ্য দিতেও সম্মত আছেন এবং পাশাপাশি আপনার এবং আপনার শিশুর মল+ রক্তের নমুনা+ মুখগহবর মিউকাসের নমুনা এবং শারীরিক পরিমাপ যেমন, উচ্চতা+ ওজন সংগ্রহের অনুমতি দিচ্ছেন] আপনার শিশুকে আরও আমন্ত্রন জানানো হবে '[illegible](বুদ্ধি পরীক্ষা+ আবেগীয় নিয়ন্ত্রন+ [illegible] এবং [illegible], পরীক্ষা তে অংশগ্রহন করার জন্য] এছাড়াও+ এই গবেষণায় দৈবচয়ন প্রক্রিয়ার উপর ভিত্তি করে আপনার শিশু যদি ওজন.উচ্চতায়' WHZ <-2 and ≥-3 z-score, and/or MUAC <12.5 and ≥11.5 cm) মাঝারিঅপুষ্টি নিয়ে এই গবেষণায় অন্তর্ভুক্ত হয়+ তাহলে তাকে পুষ্টির পরিপূরক 'RUSF followed by SQLNS or E- RUTF followed by E- SQLNS (২টির যে কোন একটি এবং মনো,সামাজিক উদ্দীপনা দেয়া হবে] আপনার নিকট থেকে সাক্ষরিত সম্মতিপত্র পাওয়ার পর আমরা গবেষণা কার্যক্রম শুরু করবো]

যাই হোক+ আপনার শিশু যদি অপুষ্ট না হয়+ যেমন, ওজন.উচ্চতা স্বাভাবিক মাত্রায় (WHZ Score >-0 SD) হয়+ তবে আপনার শিশুকে কোনপ্রকার পুষ্টির পরিপূরক বা মনো,সামাজিক উদ্দীপনা দেয়া হবে না]

আপনার শিশুকে ৩ বছর বয়স পর্যন্ত ফলোআপ করা হবে এবংউপরের উল্লিখিত পরিক্ষাগুলি করা হবে]

বাছাইকরন এবং অন্তর্ভুক্তিকরনঃ

আমাদের প্রশিক্ষনপ্রাপ্ত আমাদের কর্মী দ্বারা বাসায়.ক্লিনিকে এই বাছাইকরন এবং অন্তর্ভুক্তিকরন করা হবে] আপনার শিশু অন্তর্ভুক্তিকরনের যোগ্য কি না নিশ্চিত করতে দলটি অন্তর্ভুক্তিকরনের যোগ্যতা পর্যালোচনা করবে] আপনার শিশু যদি যোগ্য হয় তবে দলটি আপনাকে গবেষণা সম্পর্কে ব্যাখ্যা করবে এবং এই গবেষণায় অংশগ্রহণ করার জন্য অনুরোধ করবে] যদি আপনি নিজের এবং শিশুর জন্য সম্মতিপত্রে সাক্ষর করে থাকেন+ তাহলে আপনারা উভয়ে এই গবেষণায় অন্তর্ভুক্ত হবেন এবং আমরা কিছু তথ্য বিশদভাবে সংগ্রহ করবো+ যেমন, জন্মতারিখ+ লিঙ্গ+ আপনার পারিবারিক তথ্য 'পেশা+ আয়+ শিক্ষা+ পরিবারের আকার+ শিশুকে বুকের দুধ খাওয়ানোর সময়কাল+ ইত্যাদি (] আমরা প্রয়োজনানুসারে স্বাস্থ্য তথ্যও সংগ্রহ করবো] এই অন্তর্ভুক্তিকরন সময়কাল আনুমানিক ১ ঘণ্টা হবে] আপনার শিশু যদি ওজন. উচ্চতা (WHZ <-2 and ≥-3 z-score, and/or MUAC <12.5 and ≥11.5 cm) মাঝারি অপুষ্টি নিয়ে এই গবেষণায় অন্তর্ভুক্ত হয়+ তাহলে আপনার শিশু দৈবচয়িতভাবে যে কোন একটি পুষ্টির পরিপূরকের জন্য নির্বাচিত হবে] দৈবচয়ন এবং পুষ্টি খাওয়ানো সেইসব শিশুর জন্য প্রযোজ্য নয় যারা ওজন.উচ্চতা স্বাভাবিক মাত্রায় 'WHZScore >-0 SD) নিয়ে গবেষণায় অংশগ্রহন করেছে] আমাদের ক্লিনিকে আসার জন্য আমরা আপনাকে যোগাযোগ সংক্রান্ত তথ্য এবং স্থানীয় ক্লিনিকের ঠিকানা প্রদান করবো এবং আমাদের কর্মী পুষ্টির পরিপূরক সম্পর্কিত তথ্য সংগ্রহ শুরু করবে]

শিশুদের নমুনা সংগ্রহ এবং প্রক্রিয়াঃ

- মল সংগ্রহঃ অন্তর্ভুক্তির সময়, ওজন অনুযায়ী উচ্চতা স্বাভাবিক মাত্রায় (>-1 SD) ফেরত আসার সময়, এবং ২৪ ও ৩৬ মাস বয়সে (+/- ৭ দিন)]
- মাঝারি অপুস্টির শিশুদের অতিরিক্ত আরেকটি মলের নমুনা ওজন অনুযায়ী উচ্চতা স্বাভাবিক মাত্রায় (>-1 SD) ফেরত আসার সময় সংগ্রহ করবো অথবা ৩ মাসের সময় যখন পুস্টি পরিপূরক প্রদান করা শেষ হবে (+/- ৭ দিন)]

- রক্ত (২-৩ মিঃলিঃ) সংগ্রহঃ অন্তর্ভুক্তির সময়, ওজন অনুযায়ী স্বাভাবিক মাত্রায় (>-1 SD) ফেরত আসার সময়, এবং ২৪ ও ৩৬ মাস বয়সে (+/- ৭ দিন)]
- মাঝারি অপুস্টির শিশুদের অতিরিক্ত আরেকটি র◌ক্তের নমুনা ওজন অনুযায়ী উচ্চতা স্বাভাবিক মাত্রায় (>-1 SD) ফেরত আসার সময় সংগ্রহ করবো অথবা ৩ মাসের সময় যখন পুস্টি পরিপূরক প্রদান করা শেষ হবে (+/- ৭ দিন)]

- শারীরিক পরিমাপ (উচ্চতা , ওজন ও উপর বাহুর মধ্যমার পরিধি):
  - অপুষ্ট শিশুর জন্যঃ অন্তর্ভুক্তির সময়, তারপর প্রতি সপ্তাহে একবার যতক্ষণ না ওজন অনুযায়ী উচ্চতা স্বাভাবিক মাত্রায় (>-1 SD) ফেরত না আসে এবং তারপর ৩ মাস অন্তর অন্তর পুরো গবেষণাকালের শেষ সময় পর্যন্ত (+/- ৭ দিন)]

  - অপুষ্ট নয় শিশুর জন্যঃ অন্তর্ভুক্তির সময়, তারপর ৩ মাস পর্যন্ত প্রতি মাসে, তারপর ৩ মাস অন্তর অন্তর পুরো গবেষণাকালের শেষ সময় পর্যন্ত (+/- ৭ দিন)]

- শিশুর মাথার পরিধির মাপ নেওয়া হবে: অন্তর্ভুক্তির সময়, ২৪ ও ৩৬ মাস বয়সে (+/- ২ মাস)]
- মুখগহবর থেকে মিউকাসের নমুনা সংগ্রহঃ অন্তর্ভুক্তির সময়, ২৪ ও ৩৬ মাস বয়সে (+/- ৭ দিন)]
- বুদ্ধি বিকাশের পরীক্ষাঃ 'Executive Function/বুদ্ধি পরীক্ষা, আবেগীয় নিয়ন্ত্রণ, fNIRS এবং EEG): অন্তর্ভুক্তির সময়, ২৪ ও ৩৬ মাস বয়সে (+/- ২ মাস)]

পুষ্টির পরিপূরক প্রদানঃ

দৈবচয়ন ১৯ পরে+ যে সব শিশু ওজন. উচ্চতা (WHZ <-2 and ≥-3 z-score, and/or MUAC <12.5 and ≥11.5 cm) মাঝারি অপুষ্টি নিয়ে এই গবেষণায় অন্তর্ভুক্ত হবেঃ ৭০ জন শিশুর একটি দল স্থানীয়ভাবে উৎপাদিত পরিপূরক খাবার গ্রহন করবে 'RUSF+ ৫০ গ্রাম.প্যাকেট সম্বলিত ২০৪ কিঃ ক্যালোরী শক্তি(9 RUSF এর দুই প্যাকেট প্রতিদিন পাবে যতদিন উচ্চতা অনুযায়ী ওজন সাধারণ মাত্রায় ' =0, [illegible] (ফেরত না আসে অথবা সর্বোচ্চ ৩ মাস এবং তারপর প্রতিদিন ২০ গ্রাম [illegible] প্রদান করা হবে গবেষণার শেষদিন পর্যন্ত] আরেক দলের ৭০ জন শিশু বর্ধিতভাবে প্রস্তুতকৃত থেরাপিউটিক খাবার '[illegible],[illegible](+৫০,১০০ কিঃ

ক্যালোরী.কেজি. দিন হিসাবে পাবে যতদিন উচ্চতা অনুযায়ী ওজন সাধারণ মাত্রায় ' =0, ৺৭ (ফেরত না আসে অথবা সর্বোচ্চ ৩ মাস এবং তারপর প্রতিদিন ২০ গ্রাম ש,שׁ? ףּ שׁ প্রদান করা হবে গবেষণার শেষদিন পর্যন্ত]

মনো,সামাজিক উদ্দীপনাঃ

ক্লিনিক এবং বাড়ি পরিদর্শনের সময় আমাদের স্বাস্থ্যকর্মীগন মায়েদের দেখিয়ে দিবেন যে তাদের শিশুদের সাথে বাড়িতে তৈরি খেলনা এবং বই দিয়ে কিভাবে খেলতে হয় এবং ভাবের আদান প্রদানের মাধ্যমে কিভাবে শিশুদের বিকাশ ভাল করা যায়] শিশুর বিকাশের সাথে সম্পর্কিত একটি খেলনার সেট দেয়া হবে+ যাতে পরবর্তী পরিদর্শনের আগ পর্যন্ত শিশু তা দিয়ে খেলতে পারে আর শিখতে পারে]এই সমস্তকিছুই খেলাধুলার মত করে করা হবে কোন কাজের মত নয়]

আচরণগত পরিমাপ (এক্সিকিউটিভ ফাংশন/ নির্বাহী কাজ/ আবেগ নিয়ন্ত্রণ):

এক্সিকিউটিভ ফাংশন হচ্ছে এমন একটি প্রক্রিয়া যেখানে চিন্তা করা এবং কাজ করার মাঝে ব্যাক্তির সচেতন নিয়ন্ত্রণ থাকে, যার মধ্যে রয়েছে বাধানিষেধ নিয়ন্ত্রণ, পরিকল্পনা এবং জ্ঞানীয় নমনীয়তা] আবেগ নিয়ন্ত্রন হল নিজেকে নিয়ন্ত্রনের একটি অবিচ্ছেদ্য অংশ, যা আবেগ, প্রেরণা, মনোযোগ, সামাজিক মিথস্ক্রিয়া এবং শারীরিক আচরণ নিয়ন্ত্রণ করার একটি জটিল ধারণা]

আইসিডিডিআরবি মিরপুর ক্লিনিকে এই নিউরো কগনিটিভ মূল্যায়ন 'שׁאטוּבּוֹט תּבּפוּוֹפּ.বুদ্ধি পরীক্ষা+ আবেগীয় নিয়ন্ত্রণ+ ׳ךּ עי שׁ এবং שׁשוו (,র কাজগুলো ২,৩ ঘণ্টায় সম্পন্ন করা হবে] সেশনগুলি আপনার এবং আপনার শিশুর জন্য সুবিধাজনক যে কোনও একটি সময়ে দুই সপ্তাহের মধ্যে পৃথক দুই দিনে করা হবে] এই এক্সিকিউটিভ ফাংশন এবং আবেগ নিয়ন্ত্রন কার্যকলাপের বেশিরভাগই পরীক্ষক ও আপনার শিশুর মধ্যে সরাসরি ভাবের আদান প্রদান হবে যা ভিডিও ক্যামেরায় রেকর্ড করে স্কোরিং করা হবে] এই সমস্তকিছু শুধুমাত্র খেলাধুলার মাধ্যমে পরিচালিত হবে কোন কাজের মত করে নয়]

׳ךּ עי שׁ পদ্ধতিঃ

একটি সেশনে, fNIRS (ফাংশনাল নেয়ার ইনফ্রারেড স্পেক্ট্রস্কপি) প্রযুক্তি ব্যাবহার করে আপনার শিশুর মস্তিষ্কের সক্রিয়তা লিপিবদ্ধ করবো] fNIRS আপনার শিশুর মাথায় একটি উজ্জ্বল লেজার লাইট. রশ্মি প্রবেশ করিয়ে তার রক্তে অক্সিজেনের পরিমানের পরিবর্তন পরিমাপ করে এবং লিপিবদ্ধ করে রাখে] এর ফলে আমরা চিহ্নিত করতে সমর্থ হব যে কম্পিউটারের মনিটরের ছবি পরিবর্তনের সাথে সাথে আপনার শিশুর মস্তিষ্কের কোন অংশটি সক্রিয়ভাবে প্রতিক্রিয়া করে] শিশুর মাথায় যে উজ্জ্বল আলো প্রবেশ করানো হবে এতে শিশুর কোন ক্ষতি হবে না এবং সে কোন ধরনের তাপ বা ব্যাথা অনুভব করবে না]

যেকোনো ধরনের পরিবর্তন লিপিবদ্ধ করে রাখার জন্য আমরা একটি কম্পিউটার ব্যাবহার করবো] কম্পিউটারের সাথে সেন্সরস বা সংবেদক এবং প্রবস বা শলাকা সংযুক্ত থাকবে] আপনার শিশুর মাথায় প্রবস. শলাকা স্থাপন করার পূর্বে +পরিমাপক ফিতা দিয়ে শিশুর মাথা আগে মেপে নেয়া হবে যাতে fNIRS সেন্সরস . সংবেদকগুলো সঠিক ভাবে লেগে থাকে] সেন্সর .সংবেদকগুলো একটি মাথার বন্ধনীর উপর লাগানো থাক+ যা পরে শিশুর মাথার চারপাশে যথাযথ এবং শক্ত করে লাগিয়ে দেয়া হবে]

যখন শিশুর মাথায় বন্ধনী লাগানো থাকবে+ তখন শিশুকে মনিটরে চলমান মহিলার ছবি দেখানো হবে +যাতে তাদের চোখ হয় বামদিকে না হয় ডানদিকে নাড়াচাড়া করবে +অথবা ‘পিকাবু(’এক ধরনের লুকোচুরি খেলা যা সাধারণত শিশুদের সাথে খেলা হয় 'অথবা‘ ইটসি বিটসি স্পাইডার(’এক ধরনের হাতের খেলা +অথবা বিভিন্ন যানবাহন চলাচলের ছবি+ যেমন গাড়ি বা ট্রাক] কিছু কিছু ছবি এবং ভিডিও দেখানোর সময় শব্দ হতে থাকবে]

সর্বোপরি+ আমরা আপনার শিশুর বিকাশ সম্বন্ধে নানান ধরনের প্রশ্ন জিজ্ঞাসা করবো যেমন, বিভিন্ন দক্ষতা কোন কিছু স্পর্শ করা.ধরে রাখা .নাড়াচাড়া করানো+কথা বলা +দেখা+ ইত্যাদি(যাতে আমরা তার বিকাশমূলক অক্ষমতা. ধীরতা এবং কর্মসম্পাদনমূলক দক্ষতা পরীক্ষা করে দেখতে পারি] আমরা যেসব তথ্য সংগ্রহ করবো তা ব্যক্তিগত ও গোপনীয় থাকবে +এবং পরীক্ষাগুলো থেকে যদি অস্বাভাবিক কিছু পাওয়া যায় তবে+ আরও পরীক্ষা নিরীক্ষা করা হবে এবং তাকে যথাযথ বিশেষজ্ঞর কাছে পাঠানো হবে] সব পদ্ধতিসমূহ সম্পূর্ণ নিরাপদ এবং এতে আপনার শিশু কোন ব্যাথা পাবেনা] পুরোটা সময় আপনি আপনার শিশুর সাথেই থাকবেন]

ww ।। পদ্ধতিঃ

ক্লিনিকে অন্য আরেকটি ভিজিট বা সাক্ষাতের সময়+ আমরা সম্প্রসারণশীল উপকরন দিয়ে তৈরি একটি ছোট টুপি দিয়ে আপনার শিশুর মস্তিষ্কের সক্রিয়তা লিপিবদ্ধ করবো] প্রতিটি টুপিতে অনেকগুলো স্পঞ্জ .ছিদ্র রয়েছে এবং প্রতিটি স্পঞ্জের ভিতরে একটি করে ছোট রেকর্ডিং স্পন্সর . সংবেদক রয়েছে] আপনার শিশুর মাথায় টুপিটি পরানোর পূর্বে উষ্ণ লবন পানির দ্রবনে ভিজিয়ে রাখব যাতে এর স্পঞ্জগুলো ভিজে নরম হয়ে থাকে] আমরা একটি পরিমাপক ফিতা দিয়ে আপনার শিশুর মাথার চারপাশের দূরত্ব 'মাথার পরিধি) মাপব যাতে আপনার শিশুর মাথার মাপ অনুযায়ী সঠিক টুপি আমরা ব্যাবহার করতে পারি] যেহেতু আপনার শিশুর মস্তিষ্ক সক্রিয়+ সেহেতু এটি ক্রমাগত ভাবে বৈদ্যুতিক সংকেত পাঠাতে থাকবে+মাথার খুলির ওপর যা ঘুরতে থাকা অবস্থায় টুপিতে লাগানো বিশেষ সেন্সর বা সংবেদক গুলো দিয়ে ধরে রাখবো] পর্দায় আমরা তাকে কিছু মুখাবয়ব এবং আকৃতির ছবি দেখাতে থাকবো এবং আপনার শিশুর প্রতিক্রিয়া লিপিবদ্ধ করবো]

এরপরে চোখের নড়াচড়া পরিমাপ এর কাজ করবো] আই ট্র্যাকিং যন্ত্রপাতি স্থাপন করার সময়ে আপনার শিশু আপনার কোলে বসে থাকবে এবং উজ্জ্বল কিছু বৃত্তের একটি ভিডিও দেখতে থাকবে] আই ট্র্যাকারটি একটি বিশেষ কম্পিউটারের পর্দা দিয়ে বানানো যাতে পর্দার

ধার ঘেঁষে অনেকগুলো লাল আলোর ইনফ্রারেড ক্যামেরা আছে] এই ক্যামেরাগুলো চোখের নাড়াচাড়া অনুসরন করবে এবং পর্দায় দেখানো ছবিগুলো দেখার সময় আপনার শিশু পর্দার ঠিক কোন জায়গাটি দেখছে তা বুঝতে সাহায্য করবে] আপনার প্রথম ভিজিটে আমরা আপনার শিশুকে ছয় মিনিটের জন্য বিভিন্ন চলমান শিশু খেলনার ভিডিও দেখাবো এবং আমরা আপনার শিশুর মস্তিষ্কের কার্যকারিতা রেকর্ড করে রাখবো] তারপর+কম্পিউটার স্ক্রিনে দেখানো বিভিন্ন ছবির প্রতি আপনার শিশুর চোখের নড়াচড়া লিপিবদ্ধ করবো]আপনার শিশু পর্দায় কিছু মুখাবয়ব এবং প্রাকৃতিক পটভূমির ছবি দেখবে এবং কার্টুন. জোকার. সূর্য.বেলুন দেখবে] একইসাথে+ পুরো সেশনটিতে+ কোন কিছুর দিকে শিশুর তাকানো এবং আচরন একটি ডিজিটাল ভিডিও দিয়ে রেকর্ড করে রাখা হবে] এটি গবেষকদের তথ্যসমুহ ভালভাবে বিশ্লেষণ করতে সাহায্য করবে] আপনার শিশুর গোপনীয়তা রক্ষার জন্য+ তার নাম ভিডিও রেকর্ডিং এর সাথে দেয়া হবে না এবং এই ফাইল শুধুমাত্র গবেষণার প্রধান গবেষক দেখতে পারবেন]

খানা থেকে তথ্য সংগ্রহ :

আমাদের কর্মীরা ১ম এক মাস প্রতিদিন এবং তারপর সপ্তাহে ২ দিন আপনার বাসায় যেয়ে পুষ্টি পরিপুরক খাওয়ার তথ্য ও প্যাকেট যাচাই করবে] এর সাথে সাথে আপনার শিশুর জ্বর+ বমি+ ডাইরিয়াতে ভুগতেছে কিনা তার তথ্যও সংগ্রহ করবে গবেষণার শেষ পর্যন্ত] তারা আপনাকে শিশুর নির্ধারিত সাক্ষাতের কথা স্বরণ করিয়ে দিবে এবং দরকার হলে প্রাথমিক চিকিৎসার জন্য আমাদের ক্লিনিকে পাঠাবে]

বুদ্ধি পরীক্ষার মূল্যায়নের কোন কাজই ক্ষতিকারক নয়]

আপনার 'মায়ের(নমুনা সংগ্রহ এবং প্রক্রিয়াঃ

শারীরিক পরিমাপ 'উচ্চতা এবং ওজন(9 তালিকাভুক্তির ২ সপ্তাহের মধ্যে একবার]

রক্ত সংগ্রহঃ তালিকাভুক্তির ২ সপ্তাহের মধ্যে একবার ৫ মিলি]

মুখগহবর থেকে মিউকাসের নমুনাঃ তালিকাভুক্তির ২ সপ্তাহের মধ্যে একবার]

মল সংগ্রহঃ তালিকাভুক্তির ২ সপ্তাহের মধ্যে একবার ১০ গ্রাম]

ঝুঁকি এবং সুবিধাদিঃ

এই গবেষণায় অংশগ্রহণের ঝুঁকিসমূহ কি কি>

কখনো কখনো গবেষণায় অংশগ্রহণকারীদের সাথে এমন কিছু ঘটে যা তাদের কোন ক্ষতি করতে পারে বা তাদের খারাপ অনুভূতি হতে পারে] এইগুলো কে ঝুঁকি বলা হয়] এই গবেষণায় অংশগ্রহণের ঝুঁকি সমূহের মধ্যে রয়েছে পুষ্টির পরিপূরক প্রদান+ বুদ্ধি পরীক্ষা এবং নমুনা সংগ্রহ করার সময়]

পুষ্টির পরিপূরকঃ আগের কোন গবেষণায় কোন খারাপ প্রভাব হয়েছিলো বলে জানা নাই+ তবে আমরা ইন্টারভেনশন সম্পর্কিত আরও তথ্য সংগ্রহ করবো]

রক্ত সংগ্রহঃ হাল্কা ব্যাথা+ অস্বস্তি+ রক্তপাত অথবা জখম+ অথবা সুঁই বা লেন্সেট রয়েছে এমন কোন ইনজেকশন দেয়া 'যা অত্যন্ত বিরল( ] এই ঝুঁকি সমূহ কমানোর জন্য+ শুধুমাত্র প্রশিক্ষণ প্রাপ্ত+ অভিজ্ঞ কর্মী রক্ত সংগ্রহ করবে+ এবং দ্বিতীয়বার ব্যাবহার অনুপযোগী উপকরণ ব্যাবহার করা হবে] যদি রক্ত সংগ্রহ করার সময় কোন সংক্রমণ হয়+ প্রয়োজনীয় চিকিৎসা প্রদাণ করা হবে, যার জন্য আপনাকে কোন খরচ করতে হবে না]

মল সংগ্রহঃ মল সংগ্রহ করার সময় কোনরকম ঝুঁকি আছে বলে আমরা প্রত্যাশা করি না]

মুখগহবর থেকে মিউকাসের নমুনাঃ মুখগহবর থেকে মিউকাসের নিঃসৃত নমুনা সংগ্রহ করার সময় কোনরকম ঝুঁকি আছে বলে আমরা প্রত্যাশা করি না]

বুদ্ধি পরীক্ষার মূল্যায়নঃ এই মূল্যায়ন গুলোতে তেমন কোন বড় ঝুঁকি নেই] প্রতিদিনের করা পরীক্ষার সংখ্যা কিছুটা ক্লান্তির কারন হতে পারে+ তবে আপনার এবং আপনার শিশুকে আপনাদের প্রয়োজনমত বিশ্রাম দেয়া হবে]সমস্ত পরীক্ষা এবং পদ্ধতি শিশুর শরীরের বাইরে করা হবে+যা ব্যাথামুক্ত এবং কোন খারাপ প্রভাব মুক্ত]
এই পরীক্ষাগুলো থেকে যদি এমন কিছু পাওয়া যায় যা অস্বাভাবিক বা উদ্বেগজনক 'ড্ড্য কার্যক্রমে অস্বাভাবিক খিঁচুনি(+ তবে শিশুকে এই বিষয়ে অভিজ্ঞ বাংলাদেশ এর কোন বিশেষজ্ঞের কাছে রেফার করে দেয়া হবে+ এবং প্রয়োজনীয় তথ্যসহ পরবর্তীতে আপনাকে কি করতে হবে তা জানানো হবে] এই গবেষণায় আপনাকে বিশেষজ্ঞের কাছে যাবার জন্য গাড়ির ব্যবস্থা করে দেয়া হবে+ পরামর্শ এবং রেফারেল প্রক্রিয়ায় সাহায্য করা হবে+ তবে আমরা প্রাথমিক চিকিৎসার বাইরে অন্য কোন চিকিৎসার ব্যয়ভার বহন করবো না]

<u>সুবিধাঃ</u>
আপনি এবং আপনার শিশু বিনাখরচে ভালমানের প্রাথমিক চিকিৎসা পাবেন এবং গবেষণায় অংশগ্রহণকালীন সময়ে অন্যান্য অসুখে রেফার করে দেয়া হবে] আরও বলতে গেলে+ গবেষণা থেকে প্রাপ্ত খাবার থেকেও কিছু সুবিধা পেতে পারেন] ৩ মাস পুষ্টির পরিপূরক খাওয়ানোর পর যাদের উচ্চতা অনুযায়ী ওজন স্বাভাবিক হবে না তাদেরকে বিশেষায়িত স্বাস্থ্যকেন্দ্রে পাঠানো হবে+ কেন তারা স্বাভাবিক অবস্থায় আসছে না তার কারণ গুলো বের করার জন্য, বিশেষ ভাবে আমরা যক্ষ্মার জন্য পরীক্ষা করতে বলবো] গবেষণা চলাকালিন পুরো সময় শিশু গবেষণায় অন্তর্ভুক্ত থাকবে এবং আমরা তাকে নিবিড় পর্যবেক্ষণ করবো]

<u>আর্থিক সুবিধাঃ</u>
গবেষনায় অংশগ্রহণের জন্য সরাসরি কোন আর্থিক সুবিধা দেয়া হবে না+ তবে যদি উদ্বেগজনক কোন তথ্য পাওয়া যায় তখন বিনা খরচে বিশেষজ্ঞের কাছে রেফার করে দেয়া হবে] এছাড়াও নির্ধারিত সাক্ষাতের জন্য যাতায়াত খরচ এবং ক্লিনিকে অবস্থানকালীন সময়ে কিছু খাবার দেয়া হবে]

<u>ব্যাক্তিগত+ নামহীনতা এবং গোপনীয়তা</u>

আপনার এবং আপনার শিশুর কাছ থেকে সংগৃহীত সকল তথ্য গোপনীয় রাখা হবে এবং তা একটি সুরক্ষিত জায়গায় গবেষণার গবেষকবৃন্দের দায়িত্বে জমা রাখা হবে] তথ্যসমূহ আইসিডিডিআরবি এবং বোস্টন চিলড্রেন হসপিটালের সুরক্ষিত সার্ভারে জমা রাখা হবে] জৈবিক নমুনা কোনরকম চিহ্নিতকরণ তথ্য ছাড়া আইসিডিডিআরবি তে সুরক্ষিতভাবে রাখা হবে] নিউরো ইমেজিং পরীক্ষাগুলো পৃথক কক্ষে করানো হবে+ এবং অংশগ্রহণকারীকে একটি পুনঃ চিহ্নিতকরণ নাম্বার দিয়ে কোড করা হবে] উপরন্তু + কর্মী এবং গবেষকবৃন্দের গবেষণায় অংশগ্রহণকারীদের স্বার্থ রক্ষা সম্পর্কিত কোর্স সম্পন্ন করা আছে]

সাধারনভাবে+ যারা এই গবেষণার সাথে যুক্ত আছেন+ এমনকি যারা আর্থিক সহায়তা দিয়েছেন এবং গবেষণার নীতিনির্ধারক তারাও তথ্যসমূহ দেখতে পাবেন+ যার মধ্যে রয়েছে আপনার এবং আপনার শিশুর সম্পর্কিত তথ্য+ স্থিরচিত্র এবং ভিডিও] উদাহরনস্বরূপ+ নিম্নলিখিত ব্যাক্তিবর্গ আপনার এবং আপনার শিশুর সম্পর্কিত তথ্য জানতে পারে

- আইসিডিডিআর,বি র গবেষনাকর্মী
- বোস্টন চিলড্রেন হসপিটালের গবেষনাকর্মী
- অকল্যান্ড ইউনিভার্সিটির গবেষনাকর্মী
- ট্রপিকাল মেডিসিন রিসার্চ ইন্সটিটিউট, জ্যামাইকার গবেষনাকর্মী

এই পদ্ধতিগুলোর ফলাফল উপস্থাপন বা প্রকাশের সময় আপনার বা আপনার শিশুর নাম এবং পরিচয় কোথাও প্রকাশ করা হবে না] আপনি যদি এই ফর্মটিতে সাক্ষর করেন+ তবে আপনি তথ্য প্রকাশের অনুমতি দিতেছেন+ অনুমোদিত গবেষকবৃন্দ এবং নিরাপত্তা কমিটি+ আইসিডিডিআর+বি ইথিকাল রিভিউ কমিটি+ নীতিনির্ধারক কর্ত্রীপক্ষ 'বাংলাদেশ এবং আমেরিকা উভয়পক্ষ(+ গবেষণার অর্থ যোগানদাতা + সিনপস ' যারা গবেষনার তথ্য সংরক্ষন ও বিস্লেষনে গবেষকদের সহায়তা দিয়ে থাকে) এবং যারা দায়িত্বে আছেন+ এবং অন্যান্য গবেষণা প্রতিষ্ঠান] এই অনুমতির কোন মেয়াদউত্তীর্ণ তারিখ নেই] যদি আপনি নিজের অনুমতি প্রত্যাহার করেন এবং এই চুক্তিটি শেষ করার সিদ্ধান্ত নেন+ তবে ডঃ রাশিদুল হকের সাথে নিম্নউল্লেখিত ঠিকানা.নাম্বারে যোগাযোগ করুন] তিনি বা তার কর্মীরা এই অনুমতি প্রত্যাহারের সিদ্ধান্ত লিখিতভাবে রাখতে সহায়তা করবেন] দয়া করে মনে রাখবেন যে+ গবেষণায় ইতোমধ্যে যেসব তথ্য নেয়া হয়েছে তা ব্যাবহার করা হতে পারে]

আপনার গবেষণায় অংশগ্রহণ করা ঐচ্ছিক] তবে+ এই ফর্মটিতে সাক্ষর না করলে আপনি এই গবেষণায় অংশগ্রহণ করতে পারবেন না]

নমুনার ভবিষ্যৎ ব্যবহারঃ

এই গবেষনা শেষে+ সব নমুনা আইসিডিডিআর+বিতে ৫ বছরের জন্য সংরক্ষন করা হবে] আপনি রাজী থাকলে+ এই নমুনা আমরা ভবিষ্যতে অন্য গবেষণার কাজে ব্যাবহারের জন্য সংরক্ষন করবো] যদি আমরা বা আমাদের অন্য কোন সহযোগী প্রতিষ্ঠান গবেষণাটি করেন+ সেক্ষেত্রে তখন যথাযথ কত্রিপক্ষের কাছ থেকে অনুমতি নেয়া হবে] ভবিষ্যতে এই নমুনা

ব্যাবহার করা হলে+ আপনার শিশুর ব্যাক্তিগত গোপনীয়তা রক্ষা করা হবে] যদি আপনি আপনার শিশুর নমুনা ভবিষ্যতে গবেষণা কাজে ব্যাবহারের জন্য সংরক্ষনের অনুমতি প্রদান করেন কিন্তু পরবর্তীতে আপনার সিদ্ধান্ত পরিবর্তন করেন+ আপনি আমাদের সাথে যোগাযোগ করলে আমরা নমুনা নস্ট করে ফেলবো] যদি আপনি সিদ্ধান্ত পরিবর্তন না করেন+ তাহলে আমরা আপনার শিশুর নমুনা ৫ বছর পর্যন্ত সংরক্ষন করবো] দ্রষ্টব্য+ নমুনা বিশেষ পরীক্ষার জন্য বাংলাদেশের বাইরে সহযোগীদের কাছে পাঠানো হতে পারে]

<u>তথ্যের ভবিষ্যৎ ব্যবহারঃ</u>

আপনার এবং আপনার শিশুর সম্পর্কিত তথ্য শেয়ার করা হবে নীতিনির্ধারক কত্রিপক্ষ সহ আইসিডিডিআর+বি ইথিকাল রিভিউ কমিটি 'שע ק (+ ইন্সটিটিউশনাল রিভিউ বোর্ড 'עי צ (অকল্যান্ড ইউনিভার্সিটি+ বোস্টন চিলড্রেন হসপিটাল+ ট্রপিকাল মেডিসিন রিসার্চ ইন্সটিটিউট+ জ্যামাইকা+ এবং গবেষণার অর্থ যোগানদাতা এবং যারা দায়িত্বে আছেন] গবেষকগণ ভবিষ্যতে গবেষণার উদ্দেশ্যে তাদের বিবেচনার ভিত্তিতে অন্যান্য গবেষকদের সাথে তথ্য এবং ডাটা ভাগ করে নিতে চাইতে পারেন]

অংশগ্রহণ না করা এবং প্রত্যাহার করার অধিকারঃ

গবেষণায় অংশগ্রহণ বিষয়টি ঐচ্ছিক এবং আপনি চাইলে অংশগ্রহণ নাও করতে পারেন অথবা যেকোন সময় নাম প্রত্যাহার করতে পারেন+ যার জন্য আপনাকে কোন ক্ষতিপূরণ দিতে হবে না বা আইসিডিডিআর+বি হতে চিকিৎসা গ্রহণেও কোন সমস্যা হবে না]

মায়ের নিজের ও তার ১ বছর বয়সী শিশুর সম্মতিপত্র

SID:

Protocol No. PR-21084	Version No. 1.00	Date: 18 July 2021	

Protocol Title: Multidimensional evaluation of the early emergence of executive function and emotional regulation in young children in Bangladesh using nutritional and psychosocial intervention: A Pilot study

Investigator's name: Dr. Rashidul Haque

Organization: International Centre for Diarrhoeal Disease Research, Bangladesh (icddr,b)

আপনি যদি আপনার শিশুকে আমাদের গবেষণায় নাম লেখানোর জন্য আমাদের প্রস্তাবে সম্মত হন তবে দয়া করে নীচের বাক্সগুলোতে √ 'চিহ্ন(দিন এবং শেষে নির্দিষ্ট স্থানে স্বাক্ষর . বাম বৃদ্ধা আঙুলের ছাপ দিন:

পয়েন্ট	স্টাটাস
আমি পড়েছি / গবেষণা কর্মীরা এই গবেষণার সমস্ত তথ্য ভার্সন ১.০, ১৮ জুলাই ২০২১ থেকে পড়ে শোনায়, তারা আরো প্রশ্ন জিজ্ঞাসা করার ও এই গবেষণা সম্পর্কে আলোচনা করার সুযোগ দেয় এবং তাদের কাছ থেকে সন্তোষজনক উত্তর পাই]	হ্যাঁ ☐ না ☐
আমি কোন কারণ ছাড়াই এই গবেষণা থেকে বের হয়ে আসতে পারি]	হ্যাঁ ☐ না ☐
আমি বুঝলাম যে আমার দেওয়া সকল তথ্য গোপন থাকবে]	হ্যাঁ ☐ না ☐
আমি গবেষণা কর্মীকে প্রতিটি নির্ধারিত ফলোআপ ভিজিট এবং হোম ভিজিটে আমার এবং আমার সন্তানের কাছ থেকে তথ্য সংগ্রহের অনুমতি দেওয়ার বিষয়ে সম্মত হই]	হ্যাঁ ☐ না ☐
আমি সম্মতি দিচ্ছি যে, এই গবেষণা সমীক্ষা থেকে প্রাপ্ত তথ্য আইসিডিডিআর, বি, বোস্টন চিলড্রেন হাসপাতাল, মার্কিন যুক্তরাষ্ট্র, অকল্যান্ড বিশ্ববিদ্যালয়, নিউজিল্যান্ড এবং ট্রপিকাল মেডিসিন রিসার্চ ইনস্টিটিউট, জামাইকা, সিনপস ' যারা গবেষনার তথ্য সংরক্ষন ও বিস্লেষনে গবেষকদের সহায়তা দিয়ে থাকে(দ্বারা সংরক্ষণ করা হবে এবং ভবিষ্যতে অশনাক্তকারী অংশগ্রহণকারী হিসাবে পাবলিক ডেটাবেজে অন্তর্ভুক্ত থাকতে পারে] অশনাক্তকরণের অর্থ হলো আমি এবং আমার শিশুর নাম দ্বারা বা ডাটাবেসে থাকা অন্যান্য সনাক্তকারী চিহ্ন দ্বারা সনাক্ত করা যাবে না] আমার পুরো নাম বা কোনও ঠিকানার বিস্তারিত কোথাও অন্তর্ভুক্ত করা হবে না] প্রকাশিত তথ্যগুলি এই গবেষণায় আমার বা আমার সন্তানের অংশগ্রহণ সনাক্ত করবে না] আমি নির্দিষ্ট ব্যক্তিদের আমার তথ্য পাওয়ার অনুমতি দিচ্ছি]	হ্যাঁ ☐ না ☐
আমি আমার সন্তানের কাছ থেকে ২,৩ মিলি রক্ত+ মল+ মুখগহবর থেকে মিউকাসের নমুনা দিতে সম্মত হয়েছি]	হ্যাঁ ☐ না ☐
আমি আমার কাছ থেকে ৫ মিলি রক্ত+ মল+ মুখগহবর থেকে মিউকাসের নমুনা দিতে সম্মত হয়েছি]	হ্যাঁ ☐ না ☐
আমার আর আমার সন্তানের কাছ থেকে যে বেনামে রক্ত+ মল+ মুখগহবর থেকে মিউকাসের নমুনা সংগ্রহ করা হয়েছিলো সেই নমুনাগুলি বিশ্লেষণের জন্য বিদেশে পাঠানোর বাপারে সম্মত হয়েছি]	হ্যাঁ ☐ না ☐

আমার সন্তানকে পুষ্টির পরিপূরক খাবার এবং মানসিক উদ্দীপনা দিতে সম্মত আছি 'যদি আমার শিশু অপুষ্টিতে ভুগে)	হ্যাঁ ☐ না ☐
আমি আমার সন্তানের বুদ্ধি বিকাশের পরীক্ষা 'এক্সিকিউটিভ ফাংশন+ইমোশনাল রেগুলেশন+ এফএনআইআরএস+ ইইজি(করতে সম্মত আছি	হ্যাঁ ☐ না ☐
নৈতিকভাবে অনুমোদিত গবেষণা তে আমি এবং আমার সন্তানের তথ্য এবং নমুনাগুলি সংরক্ষণ এবং ভবিষ্যতে ব্যবহারে সম্মত আছি]	হ্যাঁ ☐ না ☐
আমি ভবিষ্যতে গবেষণা সম্পর্কিত তথ্যের জন্য যোগাযোগ করাতে সম্মত আছি]	হ্যাঁ ☐ না ☐
আমি অনুধাবন করতে পারলাম যে+ গবেষণাতে আমার এবং আমার সন্তানের চিকিৎসার তথ্য এবং সংগ্রহ করা তথ্য পৃষ্ঠপোষক ব্যক্তি এবং নিয়ন্ত্রক কর্তৃপক্ষ দ্বারা অনুসন্ধান করা হতে পারে+ যেখানে এটি আমার এবং আমার সন্তানের এই গবেষণায় অংশ নেওয়ার ক্ষেত্রে প্রাসঙ্গিক] আমি সেই নির্দিস্ট ব্যক্তিদের আমার তথ্য পাওয়ার অনুমতি দিয়েছি]	হ্যাঁ ☐ না ☐
আমি এই গবেষণায় অংশ নিতে সম্মত আছি]	হ্যাঁ ☐ না ☐

____

____

অংশগ্রহণকারীদের স্বাক্ষর বা বাম বৃদ্ধা আঙুলের ছাপ তারিখ 'দিন.মাস.বছর(

____ ____________________

মাতা,পিতা . অভিভাবক . উপস্থিতির স্বাক্ষর বা
বাম বৃদ্ধা আঙুলের ছাপ তারিখ 'দিন.মাস.বছর(

____________________________________ ___________________

সাক্ষীর স্বাক্ষর তারিখ 'দিন.মাস.বছর(

_____________________________________ ___________________

গবেষক বা তার প্রতিনিধি স্বাক্ষর তারিখ 'দিন.মাস.বছর(

<u>যোগাযোগের জন্য</u>:
আপনার যদি কোনও প্রশ্ন থাকে তবে আপনি এখন বা যে কোনও সময় নীচে উল্লিখিত কর্মীদের কাছে জিজ্ঞাসা করতে পারেন9

যোগাযোগের উদ্দেশ্য	নাম এবং ঠিকানা	যোগাযোগের ঠিকানা
স্টাডি সম্পর্কিত যে কোনও প্রশ্নের জন্য+ বা কোনও সমস্যার জন্য	ডা: মাসুদ আলম	ঠিকানা9 বাড়ি,২৮+ এভিনিউ,১+ কলসী রোড+ মিরপুর, ১২+ ঢাকা,১২১৬- মোবাইল নংঃ ০১৭১১৫৭০৫৫০ '৭.২৪ ঘন্টা খোলা থাকবে(
	ডা: রাশিদুল হক]	ঠিকানা9 প্যারাসাইটোলজি ল্যাবরেটরি+ আইসিডিডিআর+বি+ মহাখালী+ ঢাকা,১২১২ মোবাইল9 ০১৭১৩০৯৩৮৫৯ 'সকাল ৯9০০ টা থেকে বিকাল ৫9০০ টা পর্যন্ত(
অধিকার বা সুযোগ সুবিধা জানতে বা কোনও অভিযোগ বা অসন্তুষ্টি জানাতে	এম এ সালাম খান 'আইআরবি সমন্বয়কারী(	আইআরবি সচিবালয়+ গবেষণা প্রশাসন+ আইসিডিডিআর+বি+ মহাখালী+ ঢাকা,১২১২ ফোনঃ '*৮৮,০২(৯৮২৭০৮৪ বা মোবাইল9 ০১৭১১৪২৮৯৮৯

আপনার সহযোগিতার জন্য ধন্যবাদ]
স্বাক্ষরকৃত সম্মতি পত্রের একটি অনুলিপি আপনাকে দেওয়া হবে]

Information Sheet for mother with her 3-year-old child

Protocol No. PR-21084	Version No. 1.00	Date: 18 July 2021

Protocol Title: Multidimensional evaluation of the early emergence of executive function and emotional regulation in young children in Bangladesh using nutritional and psychosocial intervention: A Pilot study

Investigator's name: Dr. Rashidul Haque

Organization: International Centre for Diarrhoeal Disease Research, Bangladesh (icddr,b)

Purpose of the research: To determine the effect of nutritional intervention for improvement of cognition and emotional regulation among the children in Bangladesh where malnutrition and social adversities are common

Background (brief introduction of the issue and the need for/ importance of the research)
We are conducting a study to understand the problem of malnutrition and poor cognitive outcomes in children of Bangladesh.Malnutrition affects around 47 million children under 5 years of age in low- and middle-income countries annually and among then more than 20 million death occurs, others suffer long term cognitive and behavioural impairment. Malnutrition causing significant number of deaths of under 5 years child globally. It is a large problem in Bangladesh where 40% of under-fives have moderately acute malnutrition. Malnutrition is further worsened by poor diet. Inadequate feeding practices leading to deficiencies in vitamin and minerals. Studies show that, during infancy and early childhood, nutrition is essential as these are the crucial period for the formation of the brain, building the foundation for the development of cognitive, neurological and socio-emotional skills throughout childhood and adulthood.

Why invited to participate in the study?
Researchers from icddr,b, the University of Auckland, New Zealand, Boston Children Hospital, USA and Tropical Medicine Research Institute, Jamaica are jointly conducting this research study in your community to understand the effect of nutritional intervention on the cognition of moderate acute malnourished chidren at 3 years old . Total 70 children at 3 years of old with WHZ <-2 and ≥-3 z-score, and/or MUAC <12.5 and ≥11.5 cm having stable moderate acute malnutrition and 70 mother will be enrolled from this community. We invite you to help us in our efforts through your child's participation in this study, because you have 3 years of old child with stable moderate or severe malnutrition and you live in this community where malnutrition and social adversities are common. Research ethics committees at icddr,b and IRBs of University of Auckland, New Zealand, Boston Children Hospital, USA and Tropical Medicine Research Institute, Jamaica have approved this research study.

Methods and procedures [What is expected from the participants of the research study?]
You and your child will be requested to participate in the study. If you agree to participate; you are agreeing to provide information about you and your child's medical and personal information as well as, to allow the collection of a stool, blood sample, buccal scrab and body measurement such as height, weight from your child and blood, stool and body measurement such as height, weight from you as per protocol for one time. Your child is also invited to participate Executive function, Emotional regulation, fNIRS and EEG for one time after enrolment. After obtaining the signed consent from you we will start the study activities.

Screening and enrolment:

Screening and enrolment will occur at the home/ clinic by our trained study team. The team will review the eligibility criteria to confirm your child is eligible. If your child is eligible then team will explain the study to you and request to participate. If you sign this consent form for you and for your child then both of you will be enrolled in the study and we will collect birth date, sex, information about your family (occupation, income, education, family size, etc). We will also collect medical information, if it is needed. This enrolment procedure will take approximately 1 hour. Your participation duration will be 3 months from enrolment.
We will provide you the contact information and location of local clinic to visit our clinic.

Procedures and sample collection for children:

- Stool collection: Within two weeks of enrolment
- Blood collection: Within two weeks of enrolment
- Anthropometry (height, weigh, MUAC, Head Circumgerence): Within two weeks of enrolment
- Buccal Scrab: Within two weeks of enrolment
- Neurodevelopmental assessment (ER, ER, fNIRS, EEG): At enrollment (2 months window)

:

<u>Behavioural Measures (Executive Functions/Emotional regulation):</u>
Executive functions are the processes involved in conscious control of thought and action including inhibitory control, planning and cognitive flexibility. Emotional Regulation is an integral part of self-regulation, which is a complex concept that regulates emotions, motivation, attention, social interactions, and physical behaviour.

This study involves two 2-3-hour sessions at the icddr,b Mirpur clinic for Neuro developmental assessment (Executive function, Emotional regulation, fNIRS, EEG) . The sessions will be scheduled on separate days within two weeks of each other at a time that is convenient for you and your child. Most of tasks of executive function and emotional regulation are direct interactions between the experimenter and the child, which will be recorded in video camera for video-scoring. These activities will be conducted in a playful manner and not as a work-oriented activity.

<u>NIRS Procedure</u>:
During one of sessions, we will record your child's brain activity using functional near-infrared spectroscopy (fNIRS). fNIRS measures and records the changes in the levels of oxygen in the blood by shining a near-infrared light into your child's head. We will be able to identify which areas of your child's brain are actively responding to the changes in images shown on the computer monitor.

For recording these changes, we use a computer which is attached to sensors and probes. Prior to placing the probes, we will measure your child's head with a measuring tape for proper fitting of the fNIRS sensors. These sensors rest on a headband, which is then placed around your child's head and adjusted for a tight fit.

While your child is wearing the headband, we will your child to watch videos of women either moving their eyes left or right, or performing the 'peekaboo' or 'itsy bitsy spider' hand games, or images of transportation vehicles. There will also be sounds playing during some of the images/videos.

Finally, we will ask many different questions about your child's development (motor skills, speech, vision, etc.) in order to test for developmental disabilities. All information provided will be confidential, and if anything is concerning, further testing will be conducted, and referrals for care

will be made free of charge. All of these procedures are completely safe and will not hurt your child. You will be with your child at all times.

EEG Procedure:
During the other visit to the clinic to perform EEG, we will record your child's brain activity using a small cap that is made of stretchy material. Each cap has many sponges on it and inside each sponge is a small recording sensor. We soak the caps in a warm salt water solution so the sponges get soft before we put the cap on the child's head. In this task, also before starting the session we need to measure the head circumference by measuring tape to get the appropriate cap size. As your brain is working, it is constantly giving off small electrical signals, which travel out to the scalp where we can pick them up with the special sensors. We will show a series of faces and patterns and record your child's brain activity during these tasks. Then, we will do the eye tracking measures. Your child will sit on your lap and watch a video of bright looming circles while we set up the eye-tracking equipment. The eye tracker is made up of a special computer monitor that has a set of infrared cameras built into the edges of the screen. Once calibrated, these cameras will follow eye movements and tell us exactly where on the screen your child is looking as he/she is watching the pictures. At your first visit we will have your child look at videos of moving infant toys for six minutes while we record your child's brain activity. Next, we will record your child's eye movements in response to a series of pictures on a computer screen. The child will see pictures of faces and scenic backgrounds for one task, and cartoon clown/suns/balloons. In addition, a digital video will be recorded to help the experimenter know when to present pictures to your child and aid data analysis. Your child's name will not be associated with the video recording and the file will be accessible only to the investigators of this study.

None of neuro developmental tasks are invasive or harmful

After completion of all baseline work your child will receive locally produced Ready ti use supplimentery food (RUSF, 50 g/packet contains 204 kcal energy): Two packets of RUSF daily for two months.

Procedures and sample collection for you (mother):

- Stool collection: 10 gm once within 2 weeks of enrolment
- Blood collection: 5ml once within 2 weeks of enrolment
- Buccal swab sample: Once within 2 weeks of enrolment
- Anthropometry (height and weight): once within 2 weeks of enrolment

Risk and benefits
What are the risks from participating in the study?
Sometimes things happen to the research participants in research studies that may hurt them or make them feel bad. These are called risks. The risks of participating in this study include risks caused by intervention, neuro cognitive assessments and sample collection.

Blood collection: Mild pain, discomfort, bleeding or bruising, or get an infection (which is extremely rare) where the needle or lancet is inserted. To minimize these risks, only trained, experienced staff will draw blood, and disposable materials will be used. if an infection results from a blood draw, we will provide any necessary treatment at no cost to you

Stool collection: We do not anticipate any risks from collecting stool.

Buccal swab: We do not anticipate any risks from collecting buccal swab

Neuro cognitive assessment: There are no major risks involved with those assessments. The number of tests during each day may cause some tiredness, but you and your children are allowed to take as many breaks as you need. All tests and procedures remain outside the child's body, pain free, and contain no bad effects.

If anything from the tests seems unusual or concerning (like abnormal seizure activity on EEG), child will be referred to the appropriate specialists in Bangladesh, and you will be contacted with information on what to do next. The study will cover the cost of transportation to the specialist, assistance with the consultation and referral process, but we will not cover the cost of medical care beyond primary medical care.

Benefit

You and Your child will receive free, high-quality primary care, and referrals for any illness through our study for the duration of participation.

Principle of compensation

There is no direct compensation for participation in this study, but in the case of any concerning information discovered, referrals to professionals will be provided free of charge. In addition, transportation to the clinic and food at the clinic will be provided.

Privacy, anonymity and confidentiality

We will keep all information collected from you and your child confidential and locked in a secure place under the responsibility of the study investigators. Data will be saved in secure servers at icddr,b and Boston Children's Hospital. Biological samples will be stored securely without identifying information at icddr,b. Neuro imaging testing will be done in private rooms, and subjects will be coded by a de-identified number. In addition, staff and researchers have completed the Course in The Protection of Human Research Subjects.

In general, anyone who is involved in this research, including those funding and regulating the study, may see the data, including information about you and your child, photos and videos. For example, the following people might see information about you and your child:

- Research staff at icddr,b
- Research staff at Boston Children's Hospital
- Research staff at Auckland University
- Research staff at Tropical Medicine Research Institute, Jamaica

Your/your child's name and identity will not be disclosed in the process of analyzing, presenting or publishing the results of these procedures.

If you sign this form, you have given us permission to release information to authorized researchers and the safety committees, icddr,b Ethical Review Committee, regulatory authorities (both in Bangladesh and the United States), the study sponsor , Synopse (a research data sharing and collaboration platform) and designees, and other research organizations. There is no expiration date to this permission. If you decide to withdraw your permission and end this agreement, please contact Dr. Rashidul Haque at the address/number listed below. He or his staff will help you document in writing your decision to withdraw this permission. Please note that any study information already obtained will continue to be used.

Your participation in this research is voluntary. However, you will not be able to participate in this study if you do not sign this form.

Future use of samples:

At the end of the study, all of the specimens will be stored at icddr,b for 5 years . If you agree, we will store these samples to be used in the future for other research purposes. If such research is conducted by us or by our collaborators, appropriate approvals from respective authorities will be secured at that time. If samples be used in the future, your and your child's privacy and anonymity will be maintained. If you consent to having your and your child's samples saved to be used for future research, but change your mind later, you may contact us and the samples will be destroyed. If not, we will store your child's specimens for 5 years. Of note, samples may be sent to collaborators outside of Bangladesh for specialized testing.

Future use of information

Information about you and your child may be shared with regulatory authorities including but not limited to the Ethical Review Committee (ERC) at the icddr,b, the Internal Review Board (IRB) at University of Auckland, Boston children hospital, Tropical Medicine Research Institute, Jamaica and the study sponsor and designees. Investigators may choose to share information and data with other researchers at their discretion for the purpose of future research.

Right not to participate and withdraw

Participation in this study is voluntary and you can choose to not participate, or withdraw at any point during the study without any penalty or loss of care.

Consent Sheet for mother with her 3-year-old child

SID:

Protocol No. PR-21084	Version No. 1.00	Date: 18 July 2021

Protocol Title: Multidimensional evaluation of the early emergence of executive function and emotional regulation in young children in Bangladesh using nutritional and psychosocial intervention: A Pilot study

Investigator's name: Dr. Rashidul Haque

Organization: International Centre for Diarrhoeal Disease Research, Bangladesh (icddr,b)

If you agree to our proposal for enrolling you and your child in our study, please put √ mark on appropriate box(es) of the following and finally sign / left thumb print on the specified place for you:

Points	Status
I have read out / study staff has read out the all information from this participants information sheet Version 1.0, Dated:18 July 2021 about the study, have had the opportunity to ask questions, discuss the study, and received satisfactory answers	Yes ☐ No ☐
I understood that I am free to leave the study without giving any reason	Yes ☐ No ☐
I understood that the information that I gave will be confidential	Yes ☐ No ☐
I agree to allow the study team to collect information from me and my child at every scheduled visit	Yes ☐ No ☐
I understood that the Information from this research study will be retained by icddr,b , Boston Children Hospital, USA, Auckland University, New Zealand and Tropical Medicine Research Institute, Jamaica, Synopse (a research data sharing and collaboration platform) and in the future may be included in a de-identified public use database. De-identified means that I and my child will not be individually identified by name or other personal identifiers in the database. My full name or any address details will not be included. Information released will not identify me or my child's participation in this research study.<br>I am giving permission for those individuals to have access to my records.	Yes ☐ No ☐
I agree to the collection of blood (2-3 ml), buccal swab, and stool from my child	Yes ☐ No ☐
I agree to the collection of blood (5 ml), buccal swab and stool sample from me	Yes ☐ No ☐
I agree that anonymised blood, buccal swab and stool (those collected from me and my child) samples can be sent overseas for analysis	Yes ☐ No ☐
I agree to perform neurocognitive tests (Executive Function, Emotional Regulation, FNIRS, EEG) to my child	Yes ☐ No ☐
I agree to storage and future use of me and my child's data and samples by ethically approved studies	Yes ☐ No ☐

I agree to feed nutritional intervention to my child	Yes ☐ No ☐
I agree to being contacted in the future for studies related to this study	Yes ☐ No ☐
I understand that relevant sections of my medical notes and data collected during the study may be looked at by individuals from the sponsor and by regulatory authorities, where it is relevant to my taking part in this research. I give my permission for those individuals to have access to my records.	Yes ☐ No ☐
I agree to participate in to this study	Yes ☐ No ☐

______________________________ _______________

Signature or left thumb impression of participant Date (dd/mmm/yyyy)

______________________________ _______________

Signature or left thumb impression of Parent/ Guardian/ Attendant Date (dd/mmm/yyyy)

______________________________ _______________

Signature of the witness Date (dd/mmm/yyyy)

______________________________ _______________

Signature of the PI or his/her representative Date (dd/mmm/yyyy)

Communication:
If you have any question, you can ask me right now or at any time later to the below mentioned personnel:

Purpose of contact	Name and address	Address for communication
For any question related to the study, or any problem	Dr. Masud Alam	Address: House no 28, Avenue 1, Kalsi Road, Mirpur-12, Dhaka-1216<br>Mobile No. 01711570550<br>(to be open 7/24 hours)
	Name of PI: Dr. Rashidul Haque.	Address: Parasitology Laboratory, IDD, icddr,b , Mohakhali, Dhaka-1212<br>Mobile: 01713093859<br>(9:00 am to 5:00 pm)
To know the rights or benefits or to log any complain or dissatisfaction	M A Salam Khan (IRB Coordinator)	IRB Secretariat, Research Administration, icddr,b, Mohakhali, Dhaka-1212<br>Phone: (+88-02) 9827084 or Mobile: 01711428989

Thank you for your cooperation.
A Copy of singed consent will be given to you.

মা ও তার ৩ বছর বয়সী শিশুর জন্য গবেষণা সম্পর্কিত তথ্য

**Protocol No. PR-21084**	**Version No. 1.00**	**Date: 18 July 2021**

Protocol Title: Multidimensional evaluation of the early emergence of executive function and emotional regulation in young children in Bangladesh using nutritional and psychosocial intervention: A Pilot study

Investigator's name: Dr. Rashidul Haque

Organization: International Centre for Diarrhoeal Disease Research, Bangladesh (icddr,b)

গবেষণার উদ্দেশ্য

এই গবেষণার উদ্দেশ্য হলো বাংলাদেশের শিশুদের বুদ্ধির বিকাশ এবং আবেগীয় নিয়ন্ত্রন উন্নয়নের জন্য পুষ্টি খাওয়ানোর প্রভাব নির্ধারণ করা যেখানে অপুষ্টি এবং সামাজিক প্রতিকুলতা বিদ্যমান]

ভুমিকা 'সমস্যার সংক্ষিপ্ত ভুমিকা এবং এই গবেষণার গুরুত্ত . প্রয়োজনীয়তা(

নিম্ন ও মধ্যআয়ের দেশগুলোর মধ্যে পাঁচ বৎসরের নীচে শিশুদের মধ্যে সাড়ে চার কোটিরও বেশী শিশু প্রতিবছর অপুষ্টির শিকার হয়] এদের মধ্যে ২ কোটি শিশু মারা যায় এবং অন্যরা দীর্ঘ মেয়াদী বুদ্ধির বিকাশগত সমস্যায় ভুগে] সারা বিশ্বে অপুষ্টির কারনে ৫ বৎসরের নীচে শিশুদের মধ্যে উল্লেখযোগ্য সংখ্যক শিশু মারা যায়] এই অপুষ্টি বাংলাদেশের একটি বড় সমস্যা যেখানে পাঁচ বৎসরের নীচের ৪০$ শিশু মাঝারি অপুষ্টিতে ভোগে] এই অপুষ্টি আরও খারাপ হয় যখন খাবারে ঘাটতি হয়] স্বল্প খাদ্য গ্রহনের অভ্যাসের কারনে শরীরে ভিটামিন ও খনিজ লবনের ঘাটতি দেয়া হয়] গবেষণায় দেখা গেছে যে+ শৈশবকাল ও শৈশবকালীন সময়ে পুষ্টি গ্রহন অপরিহার্য কারন মস্তিষ্ক গঠনের জন্য ইহা গুরুত্বপূর্ণ সময়কাল] বুদ্ধির বিকাশ+ শৈশব ও কৈশোরকালে স্নায়বিক এবং সামাজিক,মানসিক দক্ষতার ভিত্তি তৈরি করার জন্য পুষ্টি অপরিহার্য]

কেন গবেষণায় অংশগ্রহণের আমন্ত্রন জানানো হচ্ছে>

আইসিডিডিআরবি+অকল্যান্ড ইউনিভার্সিটি নিউজিল্যান্ড +বোস্টন চিলড্রেনস হসপিটাল আমেরিকা এবং ট্রপিকাল মেডিসিন রিসার্চ ইন্সটিটিউট+ জ্যামাইকার গবেষকবৃন্দ আপনাদের এলাকায় ৩ বছর বয়সী মাঝারি অপুষ্টির শিশুদের বুদ্ধির বিকাশের উপর পুষ্টি পরিপূরক খাওয়ানোর প্রভাব তুলনা করার জন্য যৌথভাবে এই গবেষণাটি পরিচালনা করছেন] আপনাদের এলাকার সর্বমোট ৭০ জন শিশু যারা ওজন.উচ্চতার (WHZ <-2 and ≥-3 z-score, and/or MUAC <12.5 and ≥11.5 cm) মাঝারি অপুষ্টি আছেএবং ৭০ জন মায়েদের এই গবেষণায় অন্তর্ভুক্ত করা হবে]আপনার শিশুকে এই গবেষণায় অংশগ্রহণের জন্য আমরা আপনাকে আমন্ত্রন

জানাচ্ছি কারন আপনি ৩ বছর বয়সী শিশুর অভিভাবক এবং যারা ওজন.উচ্চতার (WHZ <-2 and ≥-3 z-score, and/or MUAC <12.5 and ≥11.5 cm)মাঝারি অপুষ্টি আছে এবং এই এলাকায় বসবাস করছেন যেখানে অপুষ্টি এবং বিভিন্ন সামাজিক প্রতিকুলতা বিদ্যমান] আইসিডিডিআরবির গবেষণা নীতি কমিটি এবং ইউনিভার্সিটি অফ অকল্যান্ড+ নিউজিল্যান্ড র আইআরবি+ বোস্টন চিলড্রেনস হসপিটাল আমেরিকা এবং ট্রপিকাল মেডিসিন রিসার্চ ইন্সটিটিউট+ জ্যামাইকা এই গবেষণাটির অনুমোদন দিয়েছেন]

<u>পদ্ধতি ও প্রক্রিয়া 'গবেষণায় অংশগ্রহনকারীদের কাছ থেকে কি প্রত্যাশা</u>>)

আপনি এবং আপনার শিশুকে এই গবেষণায় অংশগ্রহনের জন্য অনুরোধ জানাচ্ছি] আপনি যদি অংশগ্রহনে রাজি থাকেন+তাহলে+আপনি এবং আপনার শিশুর স্বাস্থ্য এবং ব্যক্তিগত তথ্য দিতেও সম্মত আছেন এবং পাশাপাশি আপনার এবং আপনার শিশুর মল+ রক্তের নমুনা+ মুখগহবর মিউকাসের নমুনা এবং শারীরিক পরিমাপ যেমন, উচ্চতা+ ওজন সংগ্রহের অনুমতি দিচ্ছেন] আপনার শিশুকে আরও আমন্ত্রন জানানো হবে '[illegible](বুদ্ধি পরীক্ষা+ আবেগীয় নিয়ন্ত্রন+[illegible] এবং [illegible], পরীক্ষাগুলোতে একবার অংশগ্রহন করার জন্য] আপনার নিকট থেকে সাক্ষরিত সম্মতিপত্র পাওয়ার পর আমরা গবেষণা কার্যক্রম শুরু করবো]

<u>বাছাইকরন এবং অন্তর্ভুক্তিকরনঃ</u>

আমাদের প্রশিক্ষনপ্রাপ্ত আমাদের কর্মী দ্বারা বাসায়.ক্লিনিকে এই বাছাইকরন এবং অন্তর্ভুক্তিকরন করা হবে] আপনার শিশু অন্তর্ভুক্তিকরনের যোগ্য কি না নিশ্চিত করতে দলটি অন্তর্ভুক্তিকরনের যোগ্যতা পর্যালোচনা করবে] আপনার শিশু যদি যোগ্য হয় তবে দলটি আপনাকে গবেষণা সম্পর্কে ব্যাখ্যা করবে এবং এই গবেষণায় অংশগ্রহণ করার জন্য অনুরোধ করবে]

যদি আপনি নিজের এবং শিশুর জন্য সম্মতিপত্রে সাক্ষর করে থাকেন+তাহলে আপনারা উভয়ে এই গবেষণায় অন্তর্ভুক্ত হবেন এবং আমরা কিছু তথ্য বিশদভাবে সংগ্রহ করবো+ যেমন, জন্মতারিখ+ লিঙ্গ+ আপনার পারিবারিক তথ্য 'পেশা+ আয়+ শিক্ষা+ পরিবারের আকার+ ইত্যাদি( ] আমরা প্রয়োজনানুসারে স্বাস্থ্য তথ্যও সংগ্রহ করবো] এই অন্তর্ভুক্তিকরন সময়কাল আনুমানিক ১ ঘণ্টা হবে] গবেষণার সময়কাল হবে ৩ মাস]

আমাদের ক্লিনিকে আসার জন্য আমরা আপনাকে যোগাযোগ সংক্রান্ত তথ্য এবং স্থানীয় ক্লিনিকের ঠিকানা প্রদান করবো]

<u>সকল শিশুদের নমুনা সংগ্রহ এবং প্রক্রিয়াঃ</u>

- মল সংগ্রহঃ তালিকাভুক্তির ২ সপ্তাহের মধ্যে একবার]
- রক্ত (২-৩ মিঃলিঃ) সংগ্রহঃ তালিকাভুক্তির ২ সপ্তাহের মধ্যে একবার]
- শারীরিক পরিমাপ (উচ্চতা, ওজন, উপর বাহুর মধ্যমার পরিধি ও মাথার পরিধির মাপ): তালিকাভুক্তির ২ সপ্তাহের মধ্যে একবার]
- মুখগহবর থেকে মিউকাসের নমুনা সংগ্রহঃ তালিকাভুক্তির ২ সপ্তাহের মধ্যে একবার]

- বুদ্ধি বিকাশের পরীক্ষাঃ 'Executive Function/বুদ্ধি পরীক্ষা, আবেগীয় নিয়ন্ত্রণ, fNIRS এবং EEG(ঃ তালিকাভুক্তির ২ মাসের মধ্যে একবার]

<u>שאטוׂבוׂט טלוׂבתבבוׂפבת פצוׂ זפז זפז טה צוׂצפטדפש וּבקבּטוׂפּ .</u>**<u>বুদ্ধি পরীক্ষা এবং আবেগীয় নিয়ন্ত্রণঃ</u>**
এক্সিকিউটিভ ফাংশন হচ্ছে এমন একটি প্রক্রিয়া যেখানে চিন্তা করা এবং কাজ করার মাঝে ব্যাক্তির সচেতন নিয়ন্ত্রণ থাকে, যার মধ্যে রয়েছে বাধানিষেধ নিয়ন্ত্রণ, পরিকল্পনা এবং জ্ঞানীয় নমনীয়তা। আবেগ নিয়ন্ত্রন হল নিজেকে নিয়ন্ত্রনের একটি অবিচ্ছেদ্য অংশ, যা আবেগ, প্রেরণা, মনোযোগ, সামাজিক মিথস্ক্রিয়া এবং শারীরিক আচরণ নিয়ন্ত্রণ করার একটি জটিল ধারণা]

আইসিডিডিআরবি মিরপুর ক্লিনিকে এই নিউরো কগনিটিভ মূল্যায়ন 'שאטוׂבוׂט טלוׂבתבבוׂפבת פצוׂ.বুদ্ধি পরীক্ষা+ আবেগীয় নিয়ন্ত্রণ+ שעיךי এবং ששוו(,র কাজগুলো ২,৩ ঘণ্টায় সম্পন্ন করা হবে] সেশনগুলি আপনার এবং আপনার শিশুর জন্য সুবিধাজনক যে কোনও একটি সময়ে দুই সপ্তাহের মধ্যে পৃথক দুই দিনে করা হবে] এই এক্সিকিউটিভ ফাংশন এবং আবেগ নিয়ন্ত্রন কার্যকলাপের বেশিরভাগেই পরীক্ষক ও আপনার শিশুর মধ্যে সরাসরি ভাবের আদান প্রদান হবে যা ভিডিও ক্যামেরায় রেকর্ড করে স্কোরিং করা হবে] এই সমস্তকিছু শুধুমাত্র খেলাধুলার মাধ্যমে পরিচালিত হবে কোন কাজের মত করে নয়]

<u>שעיךי</u> **<u>পদ্ধতিঃ</u>**
একটি সেশনে, fNIRS (ফাংশনাল নেয়ার ইনফ্রারেড স্পেক্ট্রস্কপি) প্রযুক্তি ব্যাবহার করে আপনার শিশুর মস্তিষ্কের সক্রিয়তা লিপিবদ্ধ করবো] fNIRS আপনার শিশুর মাথায় একটি উজ্জ্বল লেজার লাইট. রশ্মি প্রবেশ করিয়ে তার রক্তে অক্সিজেনের পরিমানের পরিবর্তন পরিমাপ করে এবং লিপিবদ্ধ করে রাখে] এর ফলে আমরা চিহ্নিত করতে সমর্থ হব যে কম্পিউটারের মনিটরের ছবি পরিবর্তনের সাথে সাথে আপনার শিশুর মস্তিষ্কের কোন অংশটি সক্রিয়ভাবে প্রতিক্রিয়া করে] শিশুর মাথায় যে উজ্জ্বল আলো প্রবেশ করানো হবে এতে শিশুর কোন ক্ষতি হবে না এবং সে কোন ধরনের তাপ বা ব্যাথা অনুভব করবে না]

যেকোনো ধরনের পরিবর্তন লিপিবদ্ধ করে রাখার জন্য আমরা একটি কম্পিউটার ব্যাবহার করবো] কম্পিউটারের সাথে সেন্সরস বা সংবেদক এবং প্রবস বা শলাকা সংযুক্ত থাকবে] আপনার শিশুর মাথায় প্রবস. শলাকা স্থাপন করার পূর্বে+ পরিমাপক ফিতা দিয়ে শিশুর মাথা আগে মেপে নেয়া হবে যাতে fNIRS সেন্সরস . সংবেদকগুলো সঠিক ভাবে লেগে থাকে] সেন্সরস . সংবেদকগুলো একটি মাথার বন্ধনীর উপর লাগানো থাকে+ যা পরে শিশুর মাথার চারপাশে যথাযথ এবং শক্ত করে লাগিয়ে দেয়া হবে] যখন শিশুর মাথায় বন্ধনী লাগানো থাকবে+ তখন শিশুকে মনিটরে চলমান মহিলার ছবি দেখানো হবে+ যাতে তাদের চোখ হয় বামদিকে না হয় ডানদিকে নাড়াচাড়া করবে +অথবা' পিকাবু('এক ধরনের লুকোচুরি খেলা যা সাধারণত শিশুদের সাথে খেলা হয় 'অথবা' ইটসি বিটসি স্পাইডার('এক ধরনের হাতের খেলা+ 'অথবা বিভিন্ন যানবাহন চলাচলের ছবি+ যেমন গাড়ি বা ট্রাক] কিছু কিছু ছবি এবং ভিডিও দেখানোর সময় শব্দ হতে থাকবে]

সর্বোপরি+ আমরা আপনার শিশুর বিকাশ সম্বন্ধে নানান ধরনের প্রশ্ন জিজ্ঞাসা করবো যেমন, বিভিন্ন দক্ষতা কোন কিছু স্পর্শ করা.ধরে রাখা. নাড়াচাড়া করানো+ কথা বলা+ দেখা ইত্যাদি(যাতে আমরা তার বিকাশমূলক অক্ষমতা. ধীরতা এবং কর্মসম্পাদনমূলক দক্ষতা পরীক্ষা করে দেখতে পারি] আমরা যেসব তথ্য সংগ্রহ করবো তা ব্যক্তিগত ও গোপনীয় থাকবে+ এবং পরীক্ষাগুলো থেকে যদি অস্বাভাবিক কিছু পাওয়া যায় তবে+ আরও পরীক্ষা নিরীক্ষা করা হবে+ এবং তাকে যথাযথ বিশেষজ্ঞর কাছে পাঠানো হবে] সব পদ্ধতিসমূহ সম্পূর্ণ নিরাপদ এবং এতে আপনার শিশু কোন ব্যাথা পাবেনা] পুরোটা সময় আপনি আপনার শিশুর সাথেই থাকবেন]

ששוו পদ্ধতিঃ

ক্লিনিকে অন্য আরেকটি ভিজিট বা সাক্ষাতের সময়+ আমরা সম্প্রসারণশীল উপকরন দিয়ে তৈরি একটি ছোট টুপি দিয়ে আপনার শিশুর মস্তিষ্কের সক্রিয়তা লিপিবদ্ধ করবো] প্রতিটি টুপিতে অনেকগুলো স্পঞ্জ . ছিদ্র রয়েছে এবং প্রতিটি স্পঞ্জের ভিতরে একটি করে ছোট রেকর্ডিং স্পন্সর . সংবেদক রয়েছে] আপনার শিশুর মাথায় টুপিটি পরানোর পূর্বে উষ্ণ লবন পানির দ্রবনে ভিজিয়ে রাখব যাতে এর স্পঞ্জগুলো ভিজে নরম হয়ে থাকে] আমরা একটি পরিমাপক ফিতা দিয়ে আপনার শিশুর মাথার চারপাশের দূরত্ব ' মাথার পরিধি) মাপব যাতে আপনার শিশুর মাথার মাপ অনুযায়ী সঠিক টুপি আমরা ব্যবহার করতে পারি] যেহেতু আপনার শিশুর মস্তিষ্ক সক্রিয়+ সেহেতু এটি ক্রমাগত ভাবে বৈদ্যুতিক সংকেত পাঠাতে থাকবে+ মাথার খুলির ওপর যা ঘুরতে থাকা অবস্থায় টুপিতে লাগানো বিশেষ সেন্সর বা সংবেদক গুলো দিয়ে ধরে রাখবো] পর্দায় আমরা তাকে কিছু মুখাবয়ব এবং আকৃতির ছবি দেখাতে থাকবো এবং আপনার শিশুর প্রতিক্রিয়া লিপিবদ্ধ করবো]

এরপরে+ চোখের নড়াচড়া পরিমাপ এর কাজ করবো] আই ট্র্যাকিং যন্ত্রপাতি স্থাপন করার সময়ে আপনার শিশু আপনার কোলে বসে থাকবে এবং উজ্জ্বল কিছু বৃত্তের একটি ভিডিও দেখতে থাকবে] আই ট্র্যাকারটি একটি বিশেষ কম্পিউটারের পর্দা দিয়ে বানানো যাতে পর্দার ধার ঘেঁষে অনেকগুলো লাল আলোর ইনফ্রারেড ক্যামেরা আছে] এই ক্যামেরাগুলো চোখের নাড়াচাড়া অনুসরন করবে এবং পর্দায় দেখানো ছবিগুলো দেখার সময় আপনার শিশু পর্দার ঠিক কোন জায়গাটি দেখছে তা বুঝতে সাহায্য করবে] আপনার প্রথম ভিজিটে আমরা আপনার শিশুকে ছয় মিনিটের জন্য বিভিন্ন চলমান শিশু খেলনার ভিডিও দেখাবো এবং আমরা আপনার শিশুর মস্তিষ্কের কার্যকারিতা রেকর্ড করে রাখবো] তারপর+ কম্পিউটার স্ক্রিনে দেখানো বিভিন্ন ছবির প্রতি আপনার শিশুর চোখের নড়াচড়া লিপিবদ্ধ করবো]আপনার শিশু পর্দায় কিছু মুখাবয়ব এবং প্রাকৃতিক পটভূমির ছবি দেখবে এবং কার্টুন. জোকার. সূর্য.বেলুন দেখবে] একইসাথে+ পুরো সেশনটিতে+ কোন কিছুর দিকে শিশুর তাকানো এবং আচরন একটি ডিজিটাল ভিডিও দিয়ে রেকর্ড করে রাখা হবে] এটি গবেষকদের তথ্যসমুহ ভালভাবে বিশ্লেষণ করতে সাহায্য করবে] আপনার শিশুর গোপনীয়তা রক্ষার জন্য+ তার নাম ভিডিও রেকর্ডিং এর সাথে দেয়া হবে না এবং এই ফাইল শুধুমাত্র গবেষণার প্রধান গবেষক দেখতে পারবেন]

প্রাথমিক সব পরীক্ষা শেষে আপনার শিশুকে স্থানীয়ভাবে উৎপাদিত পরিপূরক খাবার গ্রহন করবে 'RUSF+ ৫০ গ্রাম.প্যাকেট সম্বলিত ২০৪ কিঃ ক্যালোরী শক্তি(9 RUSF এর দুই প্যাকেট প্রতিদিন ২ মাস পাবে]

বুদ্ধি পরীক্ষার মূল্যায়নের কোন কাজই ক্ষতিকারক নয়]

<u>আপনার 'মায়ের(নমুনা সংগ্রহ এবং প্রক্রিয়াঃ</u>

- মল সংগ্রহঃ তালিকাভুক্তির ২ সপ্তাহের মধ্যে একবার ১০ গ্রাম]
- রক্ত সংগ্রহঃ তালিকাভুক্তির ২ সপ্তাহের মধ্যে একবার ৫ মিলি]
- শারীরিক পরিমাপ (উচ্চতা এবং ওজন): তালিকাভুক্তির ২ সপ্তাহের মধ্যে একবার]
- মুখগহবর থেকে মিউকাসের নমুনাঃ তালিকাভুক্তির ২ সপ্তাহের মধ্যে একবার]

<u>ঝুঁকি এবং সুবিধাদিঃ</u>

এই গবেষণায় অংশগ্রহণের ঝুঁকিসমূহ কি কি>
কখনো কখনো গবেষণায় অংশগ্রহণকারীদের সাথে এমন কিছু ঘটে যা তাদের কোন ক্ষতি করতে পারে বা তাদের খারাপ অনুভূতি হতে পারে] এইগুলো কে ঝুঁকি বলা হয়] এই গবেষণায় অংশগ্রহণের ঝুঁকি সমূহের মধ্যে রয়েছে+ বুদ্ধি পরীক্ষা এবং নমুনা সংগ্রহ]

রক্ত সংগ্রহঃ হাল্কা ব্যাথা+ অস্বস্তি+ রক্তপাত অথবা জখম+ অথবা সুঁই বা লেন্সেট রয়েছে এমন কোন ইনজেকশন দেয়া 'যা অত্যন্ত বিরল( ] এই ঝুঁকি সমূহ কমানোর জন্য+ শুধুমাত্র প্রশিক্ষণ প্রাপ্ত+ অভিজ্ঞ কর্মী রক্ত সংগ্রহ করবে+ এবং দ্বিতীয়বার ব্যবহার অনুপযোগী উপকরণ ব্যবহার করা হবে] যদি রক্ত সংগ্রহ করার সময় কোন সংক্রমণ হয়+ প্রয়োজনীয় চিকিৎসা প্রদাণ করা হবে, যার জন্য আপনাকে কোন খরচ করতে হবে না]

মল সংগ্রহঃ মল সংগ্রহ করার সময় কোনরকম ঝুঁকি আছে বলে আমরা প্রত্যাশা করি না]

মুখগহবর থেকে মিউকাসের নমুনাঃ মুখগহবর থেকে মিউকাসের নিঃসৃত নমুনা সংগ্রহ করার সময় কোনরকম ঝুঁকি আছে বলে আমরা প্রত্যাশা করি না]

বুদ্ধি পরীক্ষার সময়9 এই গুলোতে তেমন কোন বড় ঝুঁকি নেই] প্রতিদিনের করা পরীক্ষার সংখ্যায় কিছুটা ক্লান্তির কারন হতে পারে+ তবে আপনার এবং আপনার শিশুকে আপনাদের প্রয়োজনমত বিশ্রাম দেয়া হবে]সমস্ত পরীক্ষা এবং পদ্ধতি শিশুর শরীরের বাইরে করা হবে+যা ব্যাথামুক্ত এবং কোন খারাপ প্রভাব মুক্ত] এই পরীক্ষাগুলো থেকে যদি এমন কিছু পাওয়া যায় যা অস্বাভাবিক বা উদ্বেগজনক 'ឃឃঃ কার্যক্রমে অস্বাভাবিক খিঁচুনি(+ তবে শিশুকে এই বিষয়ে অভিজ্ঞ বাংলাদেশ এর কোন বিশেষজ্ঞের কাছে রেফার করে দেয়া হবে+ এবং প্রয়োজনীয় তথ্যসহ পরবর্তীতে আপনাকে কি করতে হবে তা জানানো হবে] এই গবেষণায় আপনাকে

বিশেষজ্ঞের কাছে যাবার জন্য গাড়ির ব্যবস্থা করে দেয়া হবে+ পরামর্শ এবং রেফারেল প্রক্রিয়ায় সাহায্য করা হবে+ তবে আমরা প্রাথমিক চিকিৎসার বাইরে অন্য কোন চিকিৎসার ব্যয়ভার বহন করবো না]

সুবিধাঃ

আপনি এবং আপনার শিশু বিনাখরচে ভালমানের প্রাথমিক চিকিৎসা পাবেন এবং অন্যান্য অসুখে রেফার করে দেয়া হবে শুধুমাত্র গবেষণায় অংশগ্রহণকালীন সময়ের জন্য]

আর্থিক সুবিধাঃ

গবেষনায় অংশগ্রহণের জন্য সরাসরি কোন আর্থিক সুবিধা দেয়া হবে না+ তবে যদি উদ্বেগজনক কোন তথ্য পাওয়া যায় তখন বিনা খরচে বিশেষজ্ঞের কাছে রেফার করে দেয়া হবে] এছাড়াও নির্ধারিত সাক্ষাতের জন্য যাতায়াত খরচ এবং ক্লিনিকে অবস্থানকালীন সময়ে কিছু খাবার দেয়া হবে]

ব্যাক্তিগত+ নামহীনতা এবং গোপনীয়তা

আপনার এবং আপনার শিশুর কাছ থেকে সংগৃহীত সকল তথ্য গোপনীয় রাখা হবে এবং তা একটি সুরক্ষিত জায়গায় গবেষণার গবেষকবৃন্দের দায়িত্বে জমা রাখা হবে] তথ্যসমূহ আইসিডিডিআরবি এবং বোস্টন চিলড্রেন হসপিটালের সুরক্ষিত সার্ভারে জমা রাখা হবে] জৈবিক নমুনা কোনরকম চিহ্নিতকরণ তথ্য ছাড়া আইসিডিডিআরবি তে সুরক্ষিতভাবে রাখা হবে] নিউরো ইমেজিং পরীক্ষাগুলো পৃথক কক্ষে করানো হবে+ এবং অংশগ্রহণকারীকে একটি পুনঃ চিহ্নিতকরণ নাম্বার দিয়ে কোড করা হবে] উপরন্তু+ কর্মী এবং গবেষকবৃন্দের গবেষণায় অংশগ্রহণকারীদের স্বার্থ রক্ষা সম্পর্কিত কোর্স সম্পন্ন করা আছে]

সাধারনভাবে+ যারা এই গবেষণার সাথে যুক্ত আছেন+ এমনকি যারা আর্থিক সহায়তা দিয়েছেন এবং গবেষণার নীতিনির্ধারক তারাও তথ্যসমূহ দেখতে পাবেন+ যার মধ্যে রয়েছে আপনার এবং আপনার শিশুর সম্পর্কিত তথ্য+ স্থিরচিত্র এবং ভিডিও] উদাহরনস্বরূপ+ নিম্নলিখিত ব্যাক্তিবর্গ আপনার এবং আপনার শিশুর সম্পর্কিত তথ্য জানতে পারে

- আইসিডিডিআর,বির গবেষনাকর্মী
- বোস্টন চিলড্রেন হসপিটালের গবেষনাকর্মী
- অকল্যান্ড ইউনিভার্সিটির গবেষনাকর্মী
- ট্রপিকাল মেডিসিন রিসার্চ ইন্সটিটিউট, জ্যামাইকার গবেষনাকর্মী

এই পদ্ধতিগুলোর ফলাফল উপস্থাপন বা প্রকাশের সময় আপনার বা আপনার শিশুর নাম এবং পরিচয় কোথাও প্রকাশ করা হবে না] আপনি যদি এই ফর্মটিতে সাক্ষর করেন+ তবে আপনি তথ্য প্রকাশের অনুমতি দিতেছেন+ অনুমোদিত গবেষকবৃন্দ এবং নিরাপত্তা কমিটি+ আইসিডিডিআর+বি ইথিকাল রিভিউ কমিটি+ নীতিনির্ধারক কর্ত্রীপক্ষ 'বাংলাদেশ এবং আমেরিকা উভয়পক্ষ(+ গবেষণার অর্থ যোগানদাতা+ সিনপস ' যারা গবেষনার তথ্য সংরক্ষন ও বিশ্লেষনে গবেষকদের সহায়তা দিয়ে থাকে(এবং যারা দায়িত্বে আছেন+ এবং অন্যান্য গবেষণা প্রতিষ্ঠান] এই অনুমতির কোন মেয়াদউত্তীর্ণ তারিখ নেই] যদি আপনি নিজের অনুমতি প্রত্যাহার করেন

এবং এই চুক্তিটি শেষ করার সিদ্ধান্ত নেন+ তবে ডঃ রাশিদুল হকের সাথে নিম্নউল্লেখিত ঠিকানা.নাম্বারে যোগাযোগ করুন] তিনি বা তার কর্মীরা এই অনুমতি প্রত্যাহারের সিদ্ধান্ত লিখিতভাবে রাখতে সহায়তা করবেন] দয়া করে মনে রাখবেন যে+ গবেষণায় ইতোমধ্যে যেসব তথ্য নেয়া হয়েছে তা ব্যাবহার করা হতে পারে]

আপনার গবেষণায় অংশগ্রহণ করা ঐচ্ছিক] তবে+ এই ফর্মটিতে সাক্ষর না করলে আপনি এই গবেষণায় অংশগ্রহণ করতে পারবেন না]

<u>নমুনার ভবিষ্যৎ ব্যবহারঃ</u>

এই গবেষনা শেষে+ সব নমুনা আইসিডিডিআর+বিতে ৫ বছরের জন্য সংরক্ষন করা হবে] আপনি রাজী থাকলে+ এই নমুনা আমরা ভবিষ্যতে অন্য গবেষণার কাজে ব্যবহারের জন্য সংরক্ষন করবো] যদি আমরা বা আমাদের অন্য কোন সহযোগী প্রতিষ্ঠান গবেষণাটি করেন+ সেক্ষেত্রে তখন যথাযথ কত্রিপক্ষের কাছ থেকে অনুমতি নেয়া হবে] ভবিষ্যতে এই নমুনা ব্যবহার করা হলে+ আপনার শিশুর ব্যাক্তিগত গোপনীয়তা রক্ষা করা হবে] যদি আপনি আপনার শিশুর নমুনা ভবিষ্যতে গবেষণা কাজে ব্যাবহারের জন্য সংরক্ষনের অনুমতি প্রদান করেন কিন্তু পরবর্তীতে আপনার সিদ্ধান্ত পরিবর্তন করেন+ আপনি আমাদের সাথে যোগাযোগ করলে আমরা নমুনা নস্ট করে ফেলবো] যদি আপনি সিদ্ধান্ত পরিবর্তন না করেন+ তাহলে আমরা আপনার শিশুর নমুনা ৫ বছর পর্যন্ত সংরক্ষন করবো] দ্রষ্টব্য+ নমুনা বিশেষ পরীক্ষার জন্য বাংলাদেশের বাইরে সহযোগীদের কাছে পাঠানো হতে পারে]

<u>তথ্যের ভবিষ্যৎ ব্যবহারঃ</u>

আপনার এবং আপনার শিশুর সম্পর্কিত তথ্য শেয়ার করা হবে নীতিনির্ধারক কর্তৃপক্ষ সহ আইসিডিডিআর+বি ইথিকাল রিভিউ কমিটি 'שעק (+ ইন্সটিটিউশনাল রিভিউ বোর্ড 'צע' (অকল্যান্ড ইউনিভার্সিটি+ বোস্টন চিলড্রেন হসপিটাল+ ট্রপিকাল মেডিসিন রিসার্চ ইন্সটিটিউট+ জ্যামাইকা এবং গবেষণার অর্থ যোগানদাতা এবং যারা দায়িত্তে আছেন] গবেষকগণ ভবিষ্যতে গবেষণার উদ্দেশ্যে তাদের বিবেচনার ভিত্তিতে অন্যান্য গবেষকদের সাথে তথ্য এবং ডাটা ভাগ করে নিতে চাইতে পারেন]

<u>অংশগ্রহণ না করা এবং প্রত্যাহার করার অধিকারঃ</u>

গবেষণায় অংশগ্রহণ বিষয়টি ঐচ্ছিক এবং আপনি চাইলে অংশগ্রহণ নাও করতে পারেন অথবা যেকোন সময় নাম প্রত্যাহার করতে পারেন+ যার জন্য আপনাকে কোন ক্ষতিপূরণ দিতে হবে না বা আইসিডিডিআর+বি থেকে চিকিৎসা গ্রহণেও কোন সমস্যা হবে না]

মায়ের নিজের ও তার ৩ বছর বয়সী শিশুর সম্মতিপত্র
SID:

**Protocol No. PR-21084**	**Version No. 1.00**	**Date: 18 July 2021**

Protocol Title: Multidimensional evaluation of the early emergence of executive function and emotional regulation in young children in Bangladesh using nutritional and psychosocial intervention: A Pilot study

Investigator's name: Dr. Rashidul Haque

Organization: International Centre for Diarrhoeal Disease Research, Bangladesh (icddr,b)

আপনি যদি আপনার শিশুকে আমাদের গবেষণায় নাম লেখানোর জন্য আমাদের প্রস্তাবে সম্মত হন তবে দয়া করে নীচের বাক্সগুলোতে √ 'চিহ্ন(দিন এবং শেষে নির্দিষ্ট স্থানে স্বাক্ষর . বাম বৃদ্ধা আঙুলের ছাপ দিন9

**পয়েন্ট**	**স্ট্যাটাস**
আমি পড়েছি / গবেষণা কর্মীরা এই গবেষণার সমস্ত তথ্য ভার্সন ১.০, ১৮ জুলাই ২০২১ থেকে পড়ে শোনায়, তারা আরো প্রশ্ন জিজ্ঞাসা করার ও এই গবেষণা সম্পর্কে আলোচনা করার সুযোগ দেয় এবং তাদের কাছ থেকে সন্তোষজনক উত্তর পাই]	হ্যাঁ ☐ না ☐
আমি কোন কারণ ছাড়াই এই গবেষণা থেকে বের হয়ে আসতে পারি]	হ্যাঁ ☐ না ☐
আমি বুঝলাম যে আমার দেওয়া সকল তথ্য গোপন থাকবে]	হ্যাঁ ☐ না ☐
আমি গবেষণা কর্মীকে প্রতিটি নির্ধারিত ফলোআপ ভিজিট এবং হোম ভিজিটে আমার এবং আমার সন্তানের কাছ থেকে তথ্য+ উচ্চতা এবং ওজন সংগ্রহের অনুমতি দেওয়ার বিষয়ে সম্মত হই]	হ্যাঁ ☐ না ☐
আমি সম্মতি দিচ্ছি যে, এই গবেষণা সমীক্ষা থেকে প্রাপ্ত তথ্য আইসিডিডিআর, বি, বোস্টন চিলড্রেন হাসপাতাল, মার্কিন যুক্তরাষ্ট্র, অকল্যান্ড বিশ্ববিদ্যালয়, নিউজিল্যান্ড এবং ট্রপিকাল মেডিসিন রিসার্চ ইনস্টিটিউট, জামাইকা, সিনপস ' যারা গবেষনার তথ্য সংরক্ষন ও বিশ্লেষনে গবেষকদের সহায়তা দিয়ে থাকে(দ্বারা সংরক্ষণ করা হবে এবং ভবিষ্যতে অশনাক্তকারী অংশগ্রহণকারী হিসাবে পাবলিক ডেটাবেজে অন্তর্ভুক্ত থাকতে পারে] অশনাক্তকরণের অর্থ হলো আমি এবং আমার শিশুর নাম দ্বারা বা ডাটাবেসে থাকা অন্যান্য সনাক্তকারী চিহ্ন দ্বারা সনাক্ত করা যাবে না] আমার পুরো নাম বা কোনও ঠিকানার বিস্তারিত কোথাও অন্তর্ভুক্ত করা হবে না]প্রকাশিত তথ্যগুলি এই গবেষণায় আমার বা আমার সন্তানের অংশগ্রহণ সনাক্ত করবে না] আমি নির্দিষ্ট ব্যক্তিদের আমার তথ্য পাওয়ার অনুমতি দিচ্ছি]	হ্যাঁ ☐ না ☐
আমি আমার সন্তানের কাছ থেকে ২,৩ মিলি রক্ত+ মল+ মুখগহবর থেকে মিউকাসের নমুনা দিতে সম্মত হয়েছি]	হ্যাঁ ☐ না ☐
আমি আমার কাছ থেকে ৫ মিলি রক্ত+ মল+ মুখগহবর থেকে মিউকাসের নমুনা দিতে সম্মত হয়েছি]	হ্যাঁ ☐ না ☐

আমার আর আমার সন্তানের কাছ থেকে যে বেনামে রক্ত+ মল+ মুখগহবর থেকে মিউকাসের নমুনা সংগ্রহ করা হয়েছিলো সেই নমুনাগুলি বিশ্লেষণের জন্য বিদেশে পাঠানোর বাপারে সম্মত হয়েছি]	হ্যাঁ ☐ না ☐
আমি আমার সন্তানের বুদ্ধি বিকাশের পরীক্ষা 'এক্সিকিউটিভ ফাংশন+ ইমোশনাল রেগুলেশন+ এফএনআইআরএস+ ইইজি(করতে সম্মত আছি	হ্যাঁ ☐ না ☐
নৈতিকভাবে অনুমোদিত গবেষণা তে আমি এবং আমার সন্তানের তথ্য এবং নমুনাগুলি সংরক্ষণ এবং ভবিষ্যতে ব্যবহারে সম্মত আছি]	হ্যাঁ ☐ না ☐
আমার সন্তানকে পুষ্টির পরিপূরক খাবার দিতে সম্মত আছি	হ্যাঁ ☐ না ☐
আমি ভবিষ্যতে গবেষণা সম্পর্কিত তথ্যের জন্য যোগাযোগ করাতে সম্মত আছি]	হ্যাঁ ☐ না ☐
আমি অনুধাবন করতে পারলাম যে+ গবেষণাতে আমার এবং আমার সন্তানের চিকিৎসার তথ্য এবং সংগ্রহ করা তথ্য পৃষ্ঠপোষক ব্যক্তি এবং নিয়ন্ত্রক কর্তৃপক্ষ দ্বারা অনুসন্ধান করা হতে পারে+ যেখানে এটি আমার এবং আমার সন্তানের এই গবেষণায় অংশ নেওয়ার ক্ষেত্রে প্রাসঙ্গিক] আমি সেই নির্দিস্ট ব্যক্তিদের আমার তথ্য পাওয়ার অনুমতি দিয়েছি]	হ্যাঁ ☐ না ☐
আমি এই গবেষণায় অংশ নিতে সম্মত আছি]	হ্যাঁ ☐ না ☐

____ _____________________

অংশগ্রহণকারীদের স্বাক্ষর বা বাম বৃদ্ধা আঙুলের ছাপ তারিখ 'দিন.মাস.বছর(

____ _____________________

মাতা,পিতা . অভিভাবক . উপস্থিতির স্বাক্ষর বা
বাম বৃদ্ধা আঙুলের ছাপ তারিখ 'দিন.মাস.বছর(

____ _____________________

সাক্ষীর স্বাক্ষর তারিখ 'দিন.মাস.বছর(

____ _____________________

গবেষক বা তার প্রতিনিধি স্বাক্ষর তারিখ 'দিন.মাস.বছর(

যোগাযোগের জন্য:
আপনার যদি কোনও প্রশ্ন থাকে তবে আপনি এখন বা যে কোনও সময় নীচে উল্লিখিত কর্মীদের কাছে জিজ্ঞাসা করতে পারেন9

যোগাযোগের উদ্দেশ্য	নাম এবং ঠিকানা	যোগাযোগের ঠিকানা
স্টাডি সম্পর্কিত যে কোনও প্রশ্নের জন্য+ বা কোনও সমস্যার জন্য	ডা: মাসুদ আলম	ঠিকানা9 বাড়ি,২৮+ এভিনিউ,১+ কালসী রোড+ মিরপুর,১২+ ঢাকা,১২১৬+ মোবাইল নং: ০১৭১১৫৭০৫৫০ '৭.২৪ ঘন্টা খোলা থাকবে(
	ডা: রাশিদুল হক]	ঠিকানা9 প্যারাসাইটোলজি ল্যাবরেটরি+ আইসিডিডিআর+বি+ মহাখালী+ ঢাকা,১২১২- মোবাইল9 ০১৭১৩০৯৩৮৫৯ 'সকাল ৯৩০০ টা থেকে বিকাল ৫৩০০ টা পর্যন্ত(
অধিকার বা সুযোগ সুবিধা জানতে বা কোনও অভিযোগ বা অসন্তুষ্টি জানাতে	এম এ সালাম খান 'আইআরবি সমন্বয়কারী(	আইআরবি সচিবালয়+ গবেষণা প্রশাসন+ আইসিডিডিআর+ বি+ মহাখালী+ ঢাকা,১২১২<br>ফোন: '*৮৮,০২(৯৮২৭০৮৪ বা<br>মোবাইল9 ০১৭১১৪২৮৯৮৯

আপনার সহযোগিতার জন্য ধন্যবাদ]
স্বাক্ষরকৃত সম্মতি পত্রের একটি অনুলিপি আপনাকে দেওয়া হবে]
