# Supplementary material for: Multidimensional evaluation of the early emergence of executive function and development in Bangladeshi children using nutritional and psychosocial intervention: A randomized controlled trial protocol
Source: PLoS One. 2024 Mar 15;19(3):e0296529. doi: 10.1371/journal.pone.0296529 (PMC10942035; doi:10.1371/journal.pone.0296529)
Supplement: S4 File — (PDF) [file pone.0296529.s005.pdf]

Ethical Review Committee (ERC)
Continuation Approval

This is to certify that icddr,b research protocol # PR-21084 titled "Multidimensional evaluation of the early emergence of executive function and emotional regulation in young children in Bangladesh using nutritional and psychosocial intervention: A Pilot study": PI – Dr Rashidul Haque of the Infectious Diseases Division (IDD) had been approved by Ethical Review Committee (ERC) on 21 September 2021.

The ERC undertakes annual/periodic review of all ERC-approved protocols for reappraisal. The ERC approval for implementation of any research protocol is not, however, affected unless any unanticipated problems involving risks to the study participants or any serious or continuing noncompliance of the ERC Guidelines are detected in the implementation of the study, during the review period.

The review undertaken as of 25 August 2023 to oversee the implementation of the above protocol reveals no Adverse Event (AE) or Serious Adverse Event (SAE) or unanticipated problems involving risks to the study participants or any serious or continuing noncompliance of the ERC Guidelines. Therefore, the ERC is pleased to **approve** the protocol for continuation of its activity for next one year starting from **21 September 2023** to **20 September 2024**.

The continuing review application must be submitted to the IRB Secretariat for this study to continue beyond 20 September 2024. All necessary materials for continuing review must be reviewed with sufficient time for review and issuing continued approval before the expiration date. Failure to initiate a continuing review application in a timely fashion may result in discontinuation of study activities until approval can be renewed. Performing study activities, including data analysis, beyond the expiration date results in noncompliance of federal regulations.

Other terms and conditions for implementation of your research protocol, as contained in our memo dated 21 September 2021 according initial approval of the research protocol shall, however, remain unchanged.

____

Professor Ahmed Abu Saleh
Chairperson
Ethical Review Committee of icddr,b
Phone: +8801718378953
Email: aasaleh@gmail.com

27 August 2023
Cc: Manager, Grants, RA (GR-02289)
Senior Manager, Budget & Planning, Finance
(Budget Code # 22116420)

ETHICAL RESEARCH COMMITTEE
Continuation Approval

This is to certify that icddr,b research protocol # PR-21084 titled "Multidimensional evaluation of the early emergence of executive function and emotional regulation in young children in Bangladesh using nutritional and psychosocial intervention: A Pilot study": PI – Dr Rashidul Haque of the Infectious Diseases Division (IDD) had been approved by Ethical Review Committee (ERC) on 21 September 2021.

The ERC undertakes annual/periodic review of all ERC-approved protocols for reappraisal. The ERC approval for implementation of any research protocol is not, however, affected unless any unanticipated problems involving risks to the study participants or any serious or continuing noncompliance of the ERC Guidelines are detected in the implementation of the study, during the review period.

The review undertaken as of 30 August 2022 to oversee the implementation of the above protocol reveals no Adverse Event (AE) or Serious Adverse Event (SAE) or unanticipated problems involving risks to the study participants or any serious or continuing noncompliance of the ERC Guidelines. Therefore, the ERC is pleased to **approve** the protocol for continuation of its activity for next one year starting from **21 September 2022** to **20 September 2023**.

The continuing review application must be submitted to the IRB Secretariat for this study to continue beyond 20 September 2023. All necessary materials for continuing review must be reviewed with sufficient time for review and issuing continued approval before the expiration date. Failure to initiate a continuing review application in a timely fashion may result in discontinuation of study activities until approval can be renewed. Performing study activities, including data analysis, beyond the expiration date results in noncompliance of federal regulations.

Other terms and conditions for implementation of your research protocol, as contained in our memo dated 21 September 2021 according initial approval of the research protocol shall, however, remain unchanged.

Professor Ahmed Abu Saleh
Chairperson
Ethical Review Committee of icddr,b
01 September 2022
Cc: Manager, Grants, RA (GR-02135)
Senior Manager, Budget & Planning, Finance
(Budget Code # 22115994)

Research Review Committee
Approval Letter

21 August 2021

To: Dr Rashidul Haque
Principal Investigator of research protocol # PR-21084
Infectious Diseases Division (IDD)

From: Shafiqul Alam Sarker, MD, Ph.D, FRCP
Chairperson
Research Review Committee (RRC)

Sub: Approval of research protocol # PR-21084

Thank you for your memo dated 17 August 2021 attaching the modified version of your research protocol no. PR-21084 titled "Multidimensional evaluation of the early emergence of executive function and dysfunction in young children in Bangladesh using nutritional and psychosocial intervention: A Pilot study"; version No. 1.0, version date 18 July 2021; addressing the issues raised by the committee in its 22nd Virtual RRC meeting held on 05 August 2021 to the satisfaction of the Committee. Accordingly, the Committee approved the research protocol to proceed subject to the approval of the Ethical Review Committee (ERC).

Terms of approval

1. The research protocol is approved for 12-month period from the date of approval of the protocol by the Ethical Review Committee. Approval for further continuation of the research work, if needed, shall be obtained before expiration of the initial approval.

2. You should notify the IRB Secretariat of the start date of the protocol for updating in the integrated navision system. The protocol start date will not be updated in the navision system until receiving information from you. Therefore you will not be able to operate budget code and continue spending funds under the research protocol.

3. The RRC approval shall automatically be revoked after one year if the protocol is not started. After one year, you shall have to seek approval for revalidation of the protocol by the RRC & ERC before starting the protocol.

4. This approval is only valid whilst you hold a position at icddr,b; and in the event of your departure from the Centre, a new Principal Investigator will be designated for the research protocol.

5. You should notify the RRC and the ERC immediately of any serious or unexpected adverse effects on participants or unforeseen events that might affect continued acceptability of the protocol.

6. Any changes to the research protocol require the submission (in prescribed form) and approval of an amendment/addendum. Substantial variations may require a new protocol.

7. Continued approval of this protocol is dependent on your periodically updating the Centre's database for the protocol to show the progress; and a final report/completion report should be submitted at the conclusion of the protocol.

8. You shall submit a report for time extension of the protocol (in prescribed form) if you are unable to complete the protocol activities within the time mentioned in the protocol.

9. You are responsible for systematic storage and retention of the original data pertaining to the research protocol; and the ownership of data after certain period shall be determined as per Centre's rules and regulations.

10. The RRC should be notified if the protocol is discontinued before the expected date of completion.

I wish you all the success in conducting the research protocol.

Thank you.

Cc: Senior Director, IDD
Senior Manager, Budget & Planning, Finance

Approval Letter

21 September 2021

To: Dr Rashidul Haque
Principal Investigator of research protocol # PR-21084
Infectious Diseases Division (IDD)

From: Professor Ahmed Abu Saleh
Chairperson
Ethical Review Committee (ERC)

Sub: Approval of research protocol # PR-21084

Approval Date: 21 September 2021
Expiration Date: 20 September 2022
Review Type: Full Committee Review
Risk Level: No more than minimal
Project type: New Project

Thank you for your memo dated 18 September 2021 attaching the modified version of your research protocol # PR-21084, titled "Multidimensional evaluation of the early emergence of executive function and emotional regulation in young children in Bangladesh using nutritional and psychosocial intervention: A Pilot study"; version No. 1.0; version date 18 July 2021; addressing the issues raised by the ERC in its 27th Virtual ERC meeting held on 07 September 2021 to the satisfaction of the Committee. I am pleased to inform you that your protocol is approved. You will be required to observe the following terms and conditions in implementing the research protocol:

1. The research protocol is approved for 12-month period from the date of approval of the protocol by the Ethical Review Committee. The Federal regulations require review of an approved study not less than once per 12-month period. To comply with federal regulations, a continuing review application must be submitted to the IRB Secretariat for this study to continue beyond 20 September 2022.

   All necessary materials for continuing review must be reviewed with sufficient time for review and issuing continued approval before the expiration date. Failure to initiate a continuing review application in a timely fashion may result in discontinuation of study activities until approval can be renewed. Performing study activities, including data analysis, beyond the expiration date results in noncompliance of federal regulations.

2. The ERC approval shall automatically be revoked after one year if the protocol is not started. After one year, you shall have to seek approval for revalidation of the protocol by the ERC before starting.

Ethical Review Committee, IRB Secretariat, Research Administration, CMS
International Centre for Diarrhoeal Disease Research, Bangladesh
68, Shaheed Tajuddin Ahmed Sarani, Mohakhali, Dhaka 1212, Bangladesh
Mail: icddr,b, GPO Box 128, Dhaka 1000, Bangladesh
Phone: 880-2-9827084, Web: http://www.icddrb.org

3. You should notify the IRB Secretariat of the start date of the protocol for updating in the integrated Navision system. The protocol start date will not be updated in the Navision system until receiving information from you. Therefore, you will not be able to operate budget code and continue spending funds under the research protocol.

4. As Principal Investigator, the ultimate responsibility for scientific and ethical conduct including the protection of the rights and welfare of study participants vest upon you. You shall also be responsible for ensuring competence, integrity and ethical conduct of other investigators and staff directly involved in this research protocol.

5. You shall conduct the study in accordance with the ERC-approved protocol and shall fully comply with any subsequent determinations by the ERC.

6. You shall obtain prior approval from the Research Review Committee and the ERC for any modification in the approved research protocol and/or approved consent form(s), except in case of emergency to safeguard/ eliminate apparent immediate hazards to study participants. Such changes must immediately be reported to the ERC Chairman.

7. You shall recruit/enrol participants for this study strictly adhering to the criteria mentioned in the research protocol.

8. You shall obtain legally effective informed consent (i.e. consent should be free from coercion or undue influence) from the selected study participants or their legally responsible representative, as approved in the protocol, using the approved consent form prior to their enrolment in this study. Before obtaining consent, all prospective study participants must be adequately informed about the purpose(s) of the study, its methods and procedures, and also what would be done if they agree and also if they do not agree to participate in the study.

9. They must be informed that their participation in the study is voluntary and that they can withdraw their participation any time without any prejudice. Signed consent forms should be preserved for a period of at least five years following official termination of the study.

10. You shall promptly report the occurrence of any Serious Adverse Event or unanticipated problems of potential risk to study participants or others to the ERC in writing within 24 hours of such occurrences.

11. Any significant new findings, developing during the course of this study that might affect the risks and benefits and thus influence either participation in the study or continuation of participation should be reported in writing to the participants and the ERC.

12. You shall report progress of research to the ERC for continuing review of the implementation of the research protocol as stipulated in the ERC Guidelines. Relevant excerpt of ERC Guidelines and '*Annual/Completion* Report for Research Protocol involving Human Subjects' are attached for your information and guidance.

13. Data and/or samples should be collected and interviews should be conducted, as specified in the ERC-approved protocol, and confidentiality must be maintained. Data/samples must be protected by reasonable security, safeguarding against risks such as their loss or unauthorized access, destructions, used by others, and modification or disclosure of data. Data/samples should not be disclosed, made available to or use for purposes other than those specified in the protocol, and shall be preserved for a period, as specified under Centre's policies/practices.

14. You shall promptly and fully comply with the decision of the ERC to suspend or withdraw its approval for the research protocol.

15. The ERC should be immediately notified if the protocol is discontinued before the expected date of completion.

<u>Approved documents</u>:

a. Protocol version no 1.0 dated 18 July 2021
b. English and Bangla Information Sheet for mother with her 1-year-old child; version No 1.0 dated 18 July 2021
c. English and Bangla Consent Sheet for mother with her 1-year-old child SID; version No 1.0 dated 18 July 2021
d. English and Bangla Information Sheet for mother with her 3-year-old child; version No 1.0 dated 18 July 2021
e. English and Bangla Consent Sheet for mother with her 3-year-old child SID; version No 1.0 dated 18 July 2021

The IRB of icddr,b shall take into account the regulations of the Bangladesh Medical Research Council (BMRC), WHO, international guidelines for biomedical research as laid down by the Council of International Organization of Medical Sciences (CIOMS), the Declaration of Helsinki in relation to biomedical research involving human participants, ICH Guidelines on Good Clinical Practice (GCP), National Institutes of Health (NIH), National Institute of Allergy and Infectious Diseases (NIAID), and Division of Microbiology and Infectious Diseases (DMID). If there is any new declaration involving human participants, contents of such declaration should be appropriately adhered to and the applicable laws and policies of the local government.

I wish you success in running the above-mentioned study.

Cc: Senior Director, IDD
Senior Manager, Budget & Planning, Finance

গণপ্রজাতন্ত্রী বাংলাদেশ সরকার
ঔষধ প্রশাসন অধিদপ্তর
মহাখালী, ঢাকা-১২১২
www.dgda.gov.bd

স্মারক নং- ডিজিডিএ/ক্লিঃ ট্রাঃ প্রোঃ-০৪/২০১৬/২১০৯৯ তারিখ: ২৬/১০/২০২১

বরাবর
Dr. Rashidul Haque
Emeritus scientist
Head, Emerging Infections and Parasitology Lab
Infectious Disease Division
icddr,b, Mohakhali, Dhaka-1212

বিষয়ঃ "Multidimensional evaluation of the early emergence of executive function and emotional regulation in young children in Bangladesh using nutritional and psychosocial intervention: A Pilot study" নামীয় প্রোটকলটি অনুমোদন প্রসঙ্গে।

উপর্যুক্ত বিষয় ও আপনার আবেদনের পরিপ্রেক্ষিতে জানানো যাচ্ছে যে, গত ১৯/১০/২০২১ তারিখে অনুষ্ঠিত Clinical Trial Advisory Committee - এর সভায় প্রটোকল অনুমোদন বিষয়ে আলোচনা অনুষ্ঠিত হয়। আপনার দাখিলকৃত প্রটোকলটিতে কোন Therapeutic intervention না থাকায় উক্ত কমিটির সিদ্ধান্ত মোতাবেক প্রোটকলটি ঔষধ প্রশাসন অধিদপ্তরের অনুমোদন ব্যতিরেকে পরিচালনা করা যেতে পারে। একারনে প্রটোকলটি ঔষধ প্রশাসন অধিদপ্তর কর্তৃক অনুমোদনের প্রয়োজন নেই।

মহাপরিচালক
26 OCT 2021
ঔষধ প্রশাসন অধিদপ্তর
ফোনঃ ০২২২২২৮০৮০৩
E-mail: dgda.gov@gmail.com

AUCKLAND HEALTH RESEARCH ETHICS COMMITTEE (AHREC)

31/05/2022

Dr Justin O'Sullivan
Liggins Institute

Re: Application for Ethics Approval (Our Ref. AH23922): Approved

The Committee considered your application for ethics approval for the study entitled "**M4EFaD Bangladesh microbiome biosamples**".

We are pleased to inform you that ethics approval has been granted.

The expiry date for this approval is **31/05/2025**.

Restrictions to contact with participants in person due to the current COVID-19 protection framework setting may make the approved study methodology impractical. The Committee would like to remind researchers that they should check guidance updates and submit an amendment request if any changes need to be made to the approved ethics application to enable you to continue with your study.

Ministry of Health guidance:

https://covid19.govt.nz/

University of Auckland guidance:

https://www.auckland.ac.nz/en/news/notices/2022/covid-19.html

https://www.staff.auckland.ac.nz/en/covid-19/uoa-covid-protection-framework-plan.html

https://www.staff.auckland.ac.nz/en/covid-19/researcher-support-and-information/covid-19-research-continuity-guidance.html

If you have any questions about research continuity not answered by the pages linked above, please contact your Faculty/Institute Research Service Team representative, your Faculty/Institute Business Continuity Lead, or email researchcontinuity@auckland.ac.nz.

Researchers who are not members of the University of Auckland should also contact the DHB Research Office for further advice.

"Locality approval: Before starting your research, ensure that all the required locality approvals have been obtained. If one or more DHBs will be a locality, please contact their Research Office(s) to determine the locality approval requirements of the DHB(s)."

Auckland DHB and CM Health researcher should contact their DHB Research Office for further advice. University staff accessing DHB staff or patients as participants must likewise ensure all guidance from the DHBs are followed.

Locality approval: Before starting your research, ensure that all the required locality approvals have been obtained. If one or more DHBs will be a locality, please contact their Research Office(s) to determine the locality approval requirements of the DHB(s).

Final report: In order that up-to-date records are maintained, you must notify the Committee once your project is completed and submit a final report.

Amendments to the approved project: Should you need to make any changes to the approved project, please follow the steps below:

- Send a request to the AHREC Administrators to unlock the application form (using the Correspondence tab in Ethics RM).
- Make all changes to the relevant sections of the application form and attach revised documents (as appropriate).
- Change the Application Type to "Amendment request" in Section L.
- Add a summary of the changes requested in the text box.
- Submit the amendment request (PI/Supervisors only to submit the form).

If the project changes significantly, you are required to submit a new application.

Funded projects: If you received funding for this project, please provide this approval letter to your local Faculty Research Project Coordinator (RPC) or Research Project Manager (RPM) so that the approval can be notified via a Service Request to the Research

Operations Centre (ROC) for activation of the grant.

The Chair and the members of AHREC would be happy to discuss general matters relating to ethics approvals. If you wish to do so, please contact the AHREC Ethics Administrators at ahrec@auckland.ac.nz in the first instance.

Additional information:

- Do not forget to fill in the 'approval wording' on the PISs, CFs and/or advertisements, using the date of this approval and the reference number, before you use the documents or send them out to your participants.

All communications with the AHREC regarding this application should indicate this reference number: **AH23922**.

AHREC Administrators

Auckland Health Research Ethics Committee

AUCKLAND HEALTH RESEARCH ETHICS COMMITTEE (AHREC)

18/03/2022

Dr Justin O'Sullivan

Liggins Institute

Re: Application for Ethics Approval (Our Ref. AH23922): Conditional Approval

The Committee considered your application for ethics approval for the study entitled "**M4EFaD Bangladesh microbiome biosamples**".

Your application has been conditionally approved.

This means that you need to make the revisions required or provide further documentation or information in the application form as outlined below. Please prepare a **memo**, addressing each concern in this letter, and attach the memo and any amended documents (use **Track Changes** to highlight changes) to the application form.

Please note that until you submit the revisions and receive an approval letter, the requested changes have not been approved.

Issues to address:

1. Please note that AHREC ethics approval for this study covers only the analysis of tissue samples in New Zealand, and NOT the recruitment and interventions conducted in Bangladesh.

2. It is stated in the study protocol that unused/surplus tissue will not be returned to Bangladesh. However, in Sections C.6 and E.12 it is stated that tissue samples will be returned to current holder of existing stored human tissue. Please clarify for the Committee which is correct, and amend the application for consistency and accuracy.

3. The Committee noted from the study protocol that an MoU regarding collection, storage, use and ownership of specimen is being prepared. Please confirm this for the Committee and attach a copy of the MoU to the application.

When making changes to the attached documentation, please ensure you remove the previous version and upload the recent file version within the relevant section and question of the application form. Applications should only have one uploaded version of an attached document.

In order to make the required changes to your application:

- Click here https://apply.ethics.research.auckland.ac.nz/ProjectView/Index/23922 to open your ethics application.
- Make the changes listed under **Issues to address** and attach any revised documents.
- Attach the memo in **Section K** (Miscellaneous Documents).
- When all the required changes have been completed, go to **Section L** (Submission and Sign-off).
- Change the Application Type to “Conditional approval” in Section L
- Click the **Sign** button (Question **L4**)
  Note: This step must be done by the PI/supervisor only
- A confirmation notice that the form has been successfully received will be displayed.
- You will also receive a confirmatory email that your application has been received.

The Chair and the members of AHREC would be happy to discuss general matters relating to ethics approvals. If you wish to do so, please contact the AHREC Ethics Administrators at ahrec@auckland.ac.nz in the first instance.

Please note, if the requested revisions/clarifications are still outstanding after six months, the file will be closed and you will need to submit a new application for your research.

All communications with the AHREC regarding this application should indicate this reference number: **AH23922**.

AHREC Administrators

Auckland Health Research Ethics Committee

Institutional Review Board (IRB)
300 Longwood Avenue
Mailstop BCH 3164
Boston, MA 02115
Tel: (617) 355-7052
Fax: (617) 730-0226
www.bostonchildrens.org/research/irb

**Principal Investigator**	Charles Nelson, Ph.D
**Protocol Number**	IRB-P00039731
**Protocol Title**	The Longitudinal Accelerated Boston Study (LABS)
**Date:**	December 18, 2021

NOTICE OF EXPEDITED APPROVAL

IRB Approval Date: 11/17/2021
IRB Activation/Release Date: 12/17/2021

Please Note: The initiation or continuation of clinical research activities must follow the Boston Children's Hospital (BCH) advisories or policies. The IRB is continuing to review protocols; **however, BCH is currently restricting the conduct of clinical research in response to the COVID-19 crisis**. Details of these restrictions and an IRB Q&A document are available at https://www.childrenshospital.org/research/institutional-review-board/latest-resources-and-references and both will be updated as changes are implemented. Please contact the IRB Office with any questions you may have.

The Institutional Review Board (IRB) has approved the above referenced protocol through expedited review procedures in accordance with federal regulation 45 CFR 46.110 (b) (1), under Categories 6, 5, 7, 3, 2. We are now able to release this approval to you since you have adequately responded to the IRB's questions and concerns.

The following documents were included among the materials reviewed and approved by the IRB as part of this initial review:

- Protocol
- Consents A-E

RISK/BENEFIT ASSESSMENT

This study has been approved under HHS Regulations 45 CFR 46.404 for the inclusion of children. Risks were determined to be minimal with no potential for direct benefit

INFORMED CONSENT, ASSENT, PARENTAL PERMISSION AND HIPAA AUTHORIZATIONS

The IRB has determined that you have met the requirements to obtain method other consent through a waiver of informed consent documentation and waiver of HIPAA authorization. It is the IRB's understanding that this consent method is being utilized for the pre-screening.

The IRB has determined that only one parent/guardian is required to provide permission for their child to participate in this study and provide authorization for the use and/or disclosure of protected health information in the conduct of this research.

Assent is not required as subjects are too young to understand the research and its ramifications.

The approved consent document(s) are available on-line through the BCH the Informed Consent Library. To obtain the consent form, please go to the CHeRP IRB website and select "IC Library." Please note that the ICLibrary is accessible only through internal access and available only to those with a BCH web account. The IC Library should be accessed each time you need a consent form to ensure that the current version of the consent is always used. Do not store the consent forms on your computer or make copies for future use. Note that the activation/expiration date on the consent form can only be changed or modified by IRB Office staff. Please also note that subjects cannot be enrolled in a study if the consent form has expired. A copy of the signed consent should be kept in your files. It is our understanding that consent forms will be stored in the **research record.** The subject/family must also be given a signed copy.

Use of the short form is permitted for this protocol per the conditions and procedures outlined in the IRB's policy.

RENEWAL

In accordance with institutional policy you are required to submit an Administrative Update in advance of the anniversary of the approval date listed above.

The occurrence of unanticipated problems should promptly be reported to this office. Any revisions, amendments or changes to the protocol require prior IRB approval. The IRB has asked this office to notify investigators that clinical investigation protocol files are subject to audits at some future time.

Sincerely,

Matthew Stafford, MPH, Assistant IRB Director
For the Institutional Review Board

Tell us how we are doing! https://www.surveymonkey.com/s/irbsatisfactionsurvey

MONA CAMPUS RESEARCH ETHICS COMMITTEE
Kingston 7, Jamaica
Tel: (876) 970-4892 (876) 873-7431 (876) 927-1660-9 Ext 8192
Email: mcrec@uwimona.edu.jm

Chair, Helen Trotman-Edwards, MB, BS, DCH, DM (Paediatrics), MPH, MS (Bioethics)
Co-chair, Georgiana Gordon-Strachan, PHD, M.SC. (UCL, LSHTM/LSE)

December 2, 2021

Professor Terrence Forrester
UWI SODECO
25 West Road
Mona Campus, Kingston 7
Email: terrence.forrester@uwimona.edu.jm

Dear Professor Forrester,

Ref: CREC-MN.51, 21/22

Title: Multidimensional evaluation of the early emergence of executive function and dysfunction in young children in Bangladesh – A pilot study.

Thank you for submitting a summary of the above-mentioned proposal for review by the Mona Campus Research Ethics Committee.

The Committee is granting ethical approval for Professor Terrence Forrester to be the Co-Principal Investigator responsible for developing and executing this sub project based in Bangladesh as well as being the Head of the Management Unit for the project which will be sited at UWI.

The approval period commences on December 2, 2021 and will end on December 1, 2022.

Yours sincerely,

Professor Helen Trotman-Edwards
Chair, Mona Campus Research Ethics Committee
